# Rh(iii)-catalyzed double C–H activation of aldehyde hydrazones: a route for functionalized 1*H*-indazole synthesis†
†Electronic supplementary information (ESI) available. CCDC 1499990. For ESI and crystallographic data in CIF or other electronic format see DOI: 10.1039/c6sc03888c



**DOI:** 10.1039/c6sc03888c

**Published:** 2016-10-07

**Authors:** Pan Xu, Guoqiang Wang, Zhongkai Wu, Shuhua li, Chengjian Zhu

**Affiliations:** a State Key Laboratory of Coordination Chemistry , School of Chemistry and Chemical Engineering , Nanjing University , Nanjing 210093 , P. R. China . Email: cjzhu@nju.edu.cn; b Key Laboratory of Mesoscopic Chemistry of Ministry of Education , Institute of Theoretical and Computational Chemistry School of Chemistry and Chemical Engineering , Nanjing University , Nanjing , 210093 , P. R. China . Email: shuhua@nju.edu.cn; c State Key Laboratory of Organometallic Chemistry , Shanghai Institute of Organic Chemistry , Shanghai 200032 , P. R. China

## Abstract

A novel and straightforward strategy for functionalized 1*H*-indazoles is realized by the Rh(iii)-catalyzed double C–H activation and C–H/C–H cross coupling of readily available aldehyde phenylhydrazones. The reaction is scalable and various 1*H*-indazoles could be afforded in moderate to high yields with good functional-group compatibility. Mechanism experiments and DFT calculations suggest the distinctive Rh(iii)-catalyzed C–H/C–H cross coupling reaction underwent a cascade C(aryl)–H bond metalation, C(aldhyde)–H bond insertion and reductive elimination process.

## Abstract 2

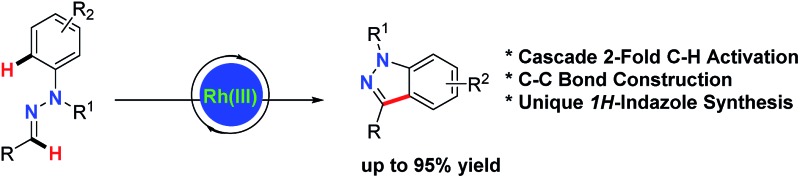
An unprecedented strategy for functionalized 1*H*-indazoles *via* C–C bond construction was realized by the Rh(iii)-catalyzed C–H/C–H cross coupling of aldehyde hydrazones.

## Introduction

**Scheme 1:**
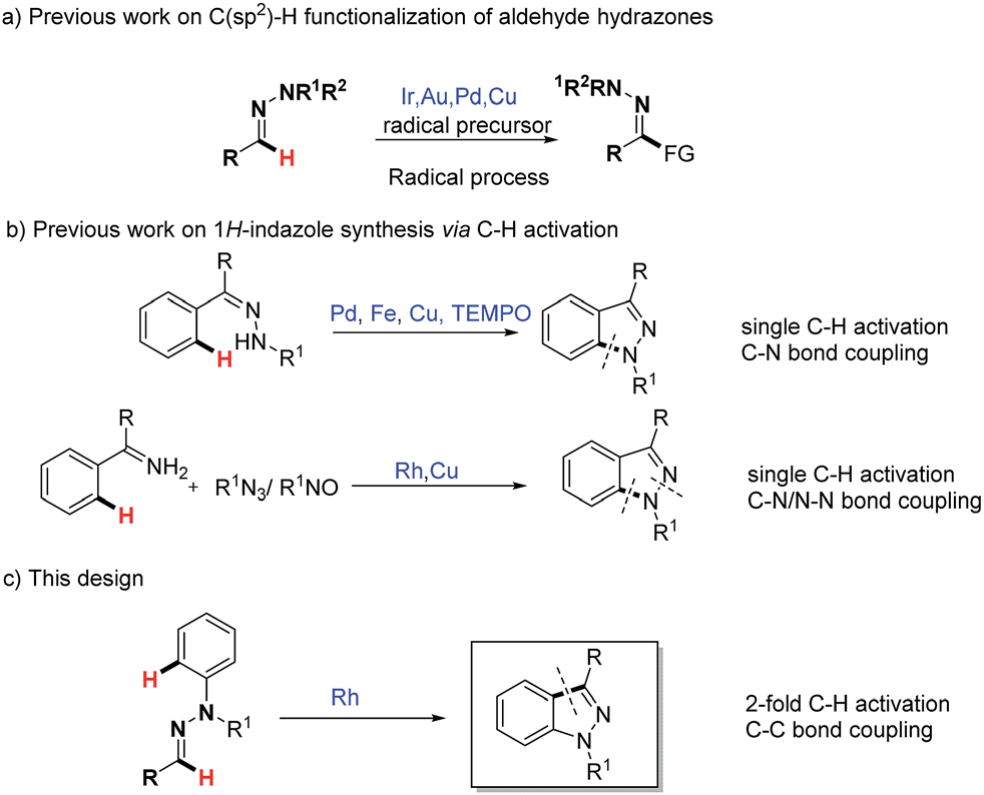
Strategy design for 1*H*-indazole synthesis from aldehyde hydrazones.

Hydrazones are particularly attractive as building blocks in synthetic organic chemistry because of their easy availability and versatile reactivity.^1^ As well as enjoying the features of carbonyl compounds, the most unique property of aldehyde hydrazone is its ability to react with active electrophiles as a neutral acyl anion equivalent, which represents an important method for the C(sp^2^)–H functionalization of aldehyde hydrazones.^2^ However, the demand of limited strong electrophilic reagents such as acyl chloride and the subsequent problem in terms of functional-group tolerance have restricted its widespread application. Hence the development of effective and general methodologies for the C(sp^2^)–H functionalization of aldehyde hydrazones is highly desired. A recent breakthrough in this area is the transition metal (Cu, Pd) or visible light catalytic strategies for the C(sp^2^)–H functionalization of aldehyde hydrazones (Scheme 1a).^3^ Mechanism experiments as well as theoretical calculations in our previous report^3*b*^ support an electron transfer induced aminyl radical-polar crossover (ARPC) process, in which the key activating unit, the N–N bond, plays a crucial role. From another point of view, the N–N bond, a Lewis basic functional unit, often appears as a directing group in transition metal-catalyzed C–H activation reactions. To complement these radical-type strategies, we have recently initiated a project to exploit the directing group strategy for the C(sp^2^)–H bond functionalization of aldehyde hydrazones.

1*H*-Indazole, a privileged pharmacophore in pharmaceuticals, is widely incorporated in multiple drugs such as anti-HIV, anti-inflammatory and anti-cancer drugs.^4^ The efficient synthesis of functionalized 1*H*-indazole has particularly attracted the increased attention of organic synthetic chemists.^5,6^ In the past few years, transition metal-catalyzed C–H activation has emerged with significant advantages toward the synthesis of a diverse array of heterocycles.^7^ Among them, Pd-,^6*a*^ Fe-,^6*b*^ Cu-^6c,e^ and Rh-^6d,f^ catalyzed C–H activation strategies have been applied to the synthesis of 1*H*-indazoles (Scheme 1b), which complement conventional approaches. Despite progress, it is noteworthy here that previous wisdom unanimously focused on the 1*H*-indazole synthesis *via* C–N bond formation. Given the importance of 1*H*-indazole synthesis and our recent ongoing interest in the C(sp^2^)–H bond functionalization of aldehyde hydrazones,^6b,c^ we wondered here whether functionalized 1*H*-indazole could be directly synthesized from readily available aldehyde phenylhydrazones through a Rh(iii)-catalyzed cascade 2-fold C–H activation and oxidative C–C bond formation (Scheme 1c). Importantly, different from previous 1*H*-indazole syntheses, such as the synthetic method, which is based on the challenging C–C bond construction, this method has its own particular advantages in the synthesis of certain distinctive and significant 1*H*-indazole scaffolds. In our strategy design, the N–N bond is initially used as a directing group in the Rh(iii)-catalyzed C(aryl)–H bond activation step. Then the resulting active metallacyclic intermediate could serve as a potential reactive linkage that is capable of the ultimate C(N)–H bond functionalization. Difficulties in this design need to be considered: (1) the regioselectivity in the presence of two different aromatic rings (C-aryl and N-aryl) is challenging; and (2) the stability of the N–N bond under the reaction condition, as in previous Rh(iii)-catalyzed hydrazine or other N–N group directed C–H activations, the N–N bond tends to cleave as an internal oxidant.^8^ To the best of our knowledge, the Rh(iii)-catalyzed C(sp^2^)–H functionalization of aldehyde hydrazones is unprecedented, thus representing an unexplored research topic.

## Results and discussion

We initially selected the readily accessible hydrazone **1a** as the model substrate to investigate our hypothesis about the Rh(iii)-catalyzed intramolecular oxidative C–H/C–H bond cross coupling. Various optimization studies revealed that the desired product 1*H*-indazole **2a** could be achieved in 80% yield with (RhCp*Cl_2_)_2_/AgOTf as the catalyst precursor, Cu(OAc)_2_ as the oxidant and K_2_CO_3_ as the base at 120 °C in 1,2-dichloroethane (Table 1 and ESI†). Control experiments were performed to test the role of each reactant. Hydrazone tends to decompose under pH-acidic reaction conditions. Thus, K_2_CO_3_ is added to neutralize the *in situ* generated acetic acid (entry 2). As expected, rhodium plays a core role in the transformation (entry 3). Catalytic Ag salt is not necessary, yet could promote the reaction with higher efficiency (entry 4). A lower loading of Cu(OAc)_2_ would induce a lower yield (entry 5) and an attempt to make the reaction greener with O_2_ as the sole oxidant failed (entry 6). The temperature effect was also examined and it was found that a higher temperature did not increase the reaction efficiency (entry 7), while lowering the reaction temperature decreased the yield (entry 8).

**Table 1:** Optimized reaction conditions^*a*^

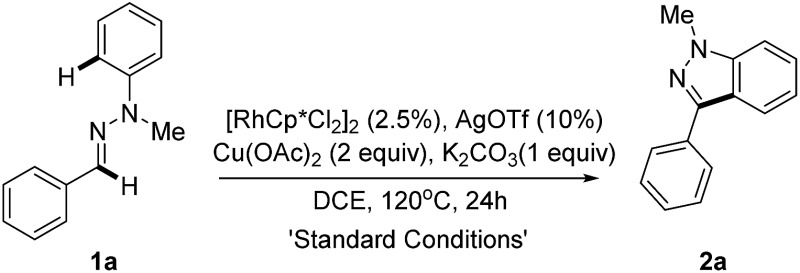			
Entry	Variation from the “standard conditions”	Conv. of **1a** ^*b*^ (%)	Yield^*b*^ (%)
1	None	84	81(80)^*c*^
2	Without K_2_CO_3_	5	Trace
3	Without [RhCp*Cl_2_]_2_	0	0
4	Without AgOTf	55	50
5	1.5 equiv. of Cu(OAc)_2_	50	42
6	O_2_ and without Cu(OAc)_2_	5	0
7	135 °C instead of 120 °C	85	80
8	100 °C instead of 120 °C	66	65

^*a*^The reactions were run on a 0.20 mmol scale in 0.5 mL of DCE.

^*b*^Yields determined by ^1^NMR spectroscopy using *N*-(4-methoxyphenyl)acetamide as the internal standard.

^*c*^Yield of isolated products. Cp* = 1,2,3,4,5-pentamethylcyclopentadiene, and DCE = 1,2-dichloroethane.

With the optimized reaction conditions in hand, the substrate scope of the reaction was next systematically explored. The representative examples are shown in Table 2. Substrates with different *N*-alkyl (Me, Et and Bn) or aryl groups all furnished the corresponding 1*H*-indazoles **2a–d** with good to excellent yields (78–95%), whereas N–H or N–Ac aldehyde hydrazones failed to give the desired product, which is probably a result of the operation of electronic effect. In addition, we found that *O*-aryl oxime derivatives cannot be transferred into benzisoxazoles under the reaction conditions. Various electron-donating (methoxy, methyl, dimethylamine) and electron-withdrawing (fluoro, chloro, bromo, trifluoromethyl, ester) groups substituted at the aldehyde phenyl rings were readily tolerated (**2e–p**). However, the position of the substituted group at the aryl group has an obvious effect on this reaction. Only *para* (**2e–k**) and *meta* (**2l–o**) substituted aldehyde hydrazones could proceed well, giving the corresponding products in good yields while *ortho* substituted aldehyde hydrazones proved unreactive with only *ortho*-fluoro hydrazone **1p** furnishing the desired product in a low yield (**2p**). This unforeseen steric effect prompted us to rethink the mechanism of this Rh(iii)-catalyzed oxidative C–H/C–H cross coupling reaction.^9^ Importantly, hetero-aldehyde derived aldehyde hydrazones such as furan and thiophene also exhibit good reactivity, providing the expected products in moderate to good yields (**2q–s**).

**Table 2:** Scope of the intramolecular C–H/C–H cross coupling of aldehyde hydrazones^*a*^ ^,^ ^*b*^

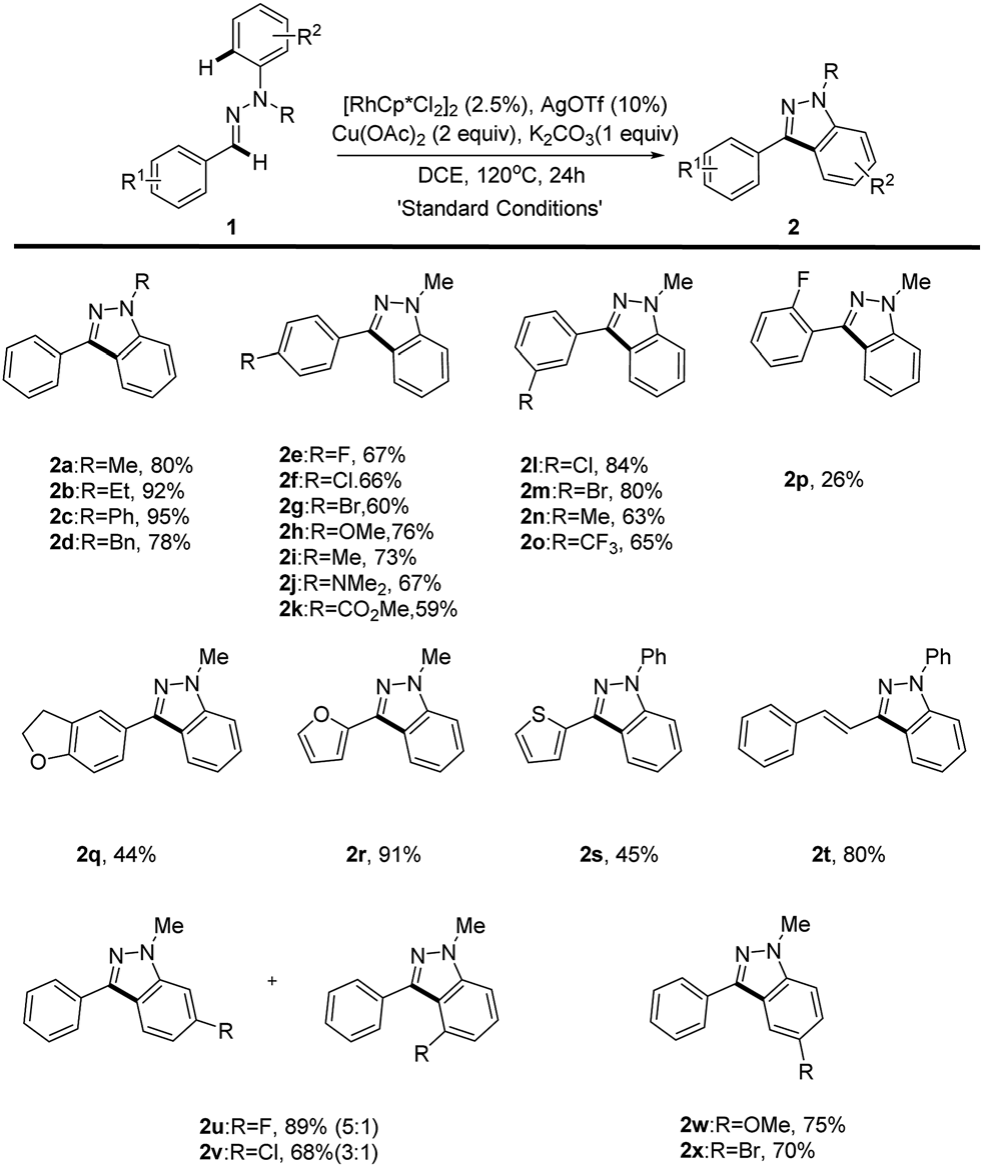

^*a*^Standard conditions.

^*b*^Yields of isolated products.

Unfortunately, the aliphatic aldehyde-derived or alkyl-substituted alkenyl aldehyde hydrazones decomposed and failed to give the desired product under our reaction conditions. The broad substrate scope of this reaction was further illustrated by altering the substitution patterns on the aryl-hydrazine unit (**2u–x**). When *meta*-substituted substrates underwent the oxidative coupling reaction, the C–H activation occurred at both positions with moderate regioselectivities (**2u–v**).

**Scheme 2:**
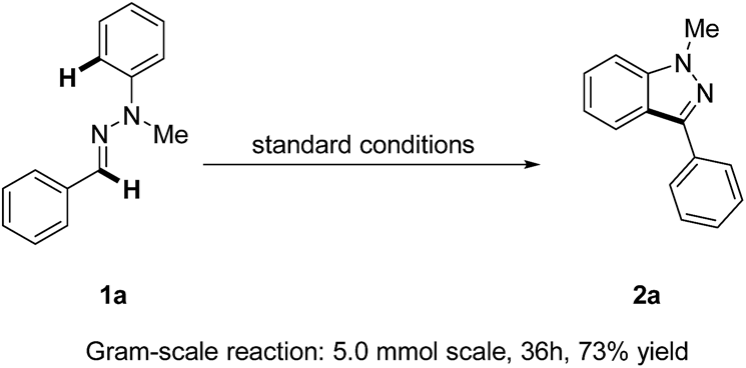
Gram-scale synthesis of 1*H*-indazole **2a**.

Remarkably, a gram-scale reaction of **1a** could still be transformed into the product **2a** in a good yield (73%) under standard reaction conditions, thus making our strategy for 1*H*-indazole synthesis more attractive and robust (Scheme 2).

To further demonstrate the unique advantages of our reaction, we applied our synthetic strategy to the rapid synthesis of certain significant and bioactive 1*H*-indazole scaffolds (Table 3), which show special 5-HT_4_/5-HT_3_ receptor antagonist activity.^10^ With readily available tetrahydroquinoline and benzo[*b*][1,4]oxazine as starting materials, a simple four-step sequence including *N*-nitrosation, reduction, condensation with aldehyde and Rh(iii)-catalyzed intramolecular oxidative C–H/C–H cross coupling could concisely furnish the desired 1*H*-indazole scaffolds in moderate overall yields. It is worth mentioning that this kind of 1*H*-indazole scaffold could not be accessed *via* previous reported synthetic strategies. The X-ray single-crystal structure analysis of the 1*H*-indazole **4f** is shown in Table 3.

**Table 3:** Representative synthesis of the bioactive fused polycyclic 1*H*-indazole skeleton^*a*^ ^,^ ^*b*^

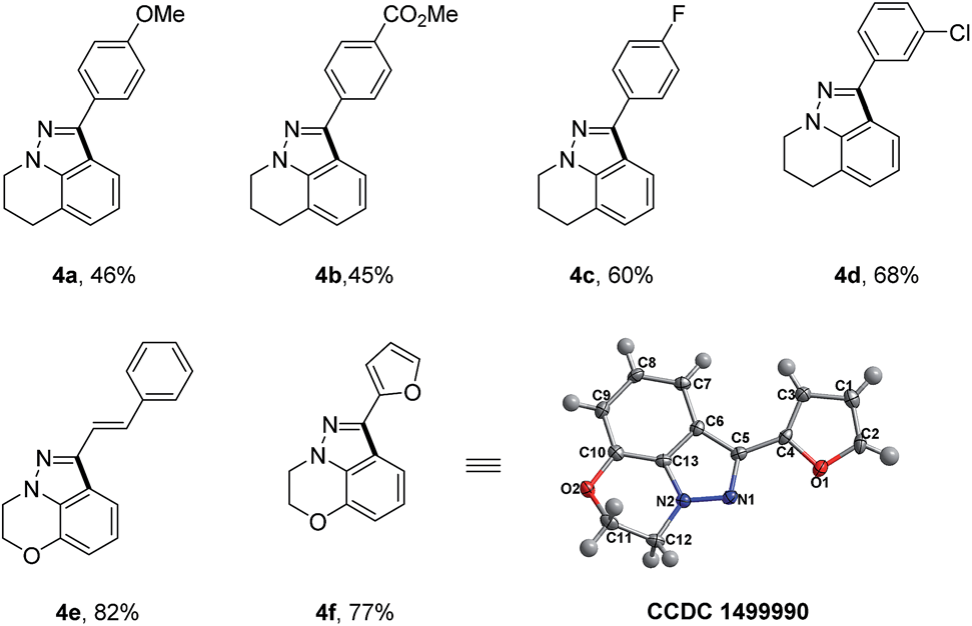

^*a*^Standard conditions.

^*b*^Yield of isolated products.

**Scheme 3:**
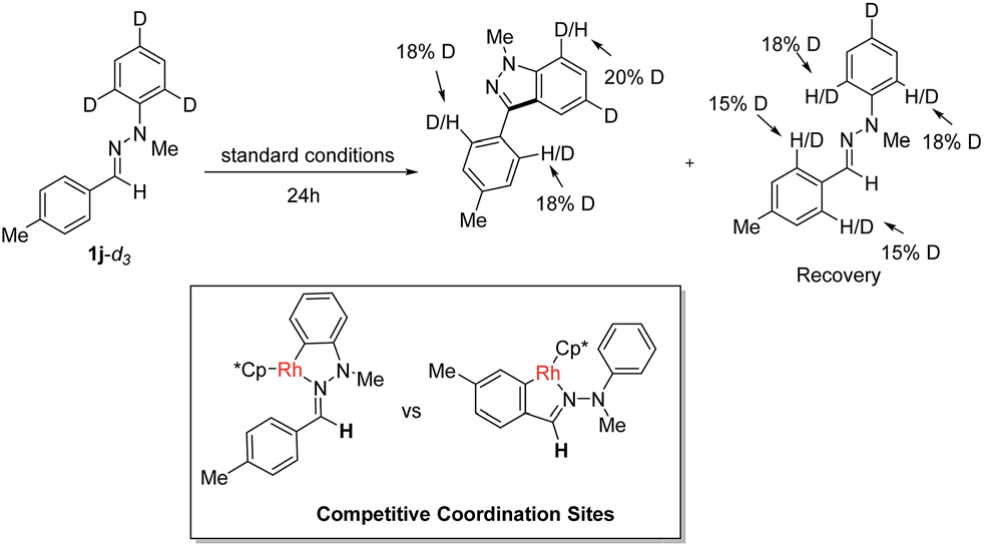
Deuterium labeling experiment.

To gain insight into this Rh(iii)-catalyzed oxidative C–H/C–H cross coupling reaction, a deuterium labeling experiment with **1j-d_3_** was carried out (Scheme 3). Deuterium losses both in the product and reclaimed substrate were observed, suggesting the process involved an initial reversible C–H bond metalation. In addition, the identification of C–H bond deuteration at the *ortho*-position of the C-aryl rings (both product and substrate) provides an evidence for the initial two competitive coordination sites with the C=N–N system as the directing group. However, valuable and convincible KIE data for this intramolecular double C–H activation process can be obtained with difficultly *via* experiments to study the rate-limiting process.

**Scheme 4:**
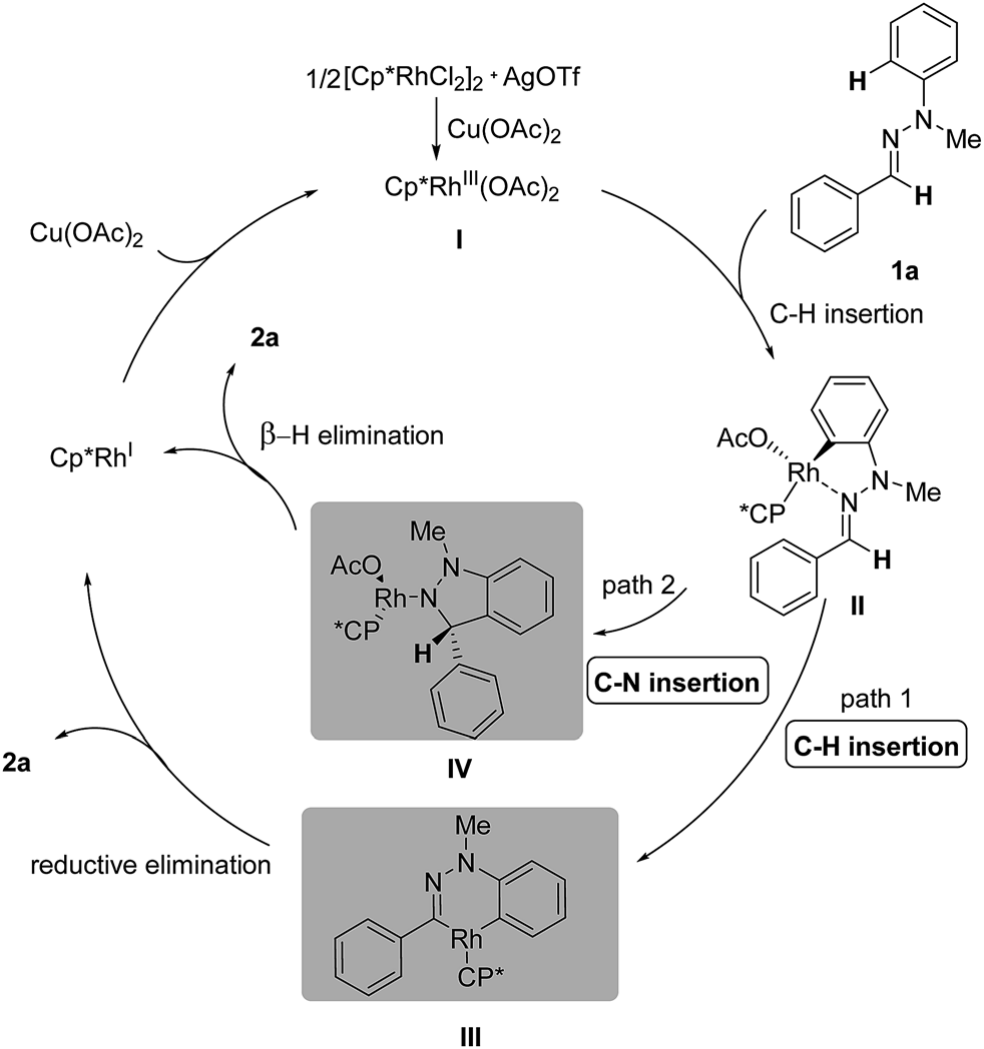
Proposed mechanism.

Based on the preliminary studies, the mechanism of this Rh(iii)-catalyzed oxidative C–H/C–H cross coupling is proposed in Scheme 4. In the presence of catalytic AgOTf and an equivalent of Cu(OAc)_2_, Rh(iii)Cp*(OAc)_2_ **I** is generated *in situ* as an active catalyst. The transformation is initiated through the coordination of the nitrogen atom of the C=N bond to the cationic Rh center and subsequent C(aryl)–H activation, generating the five-membered rhodacyclic complex **II**.^8^ Then the active intermediate serves as a reactive linkage for the second C(sp^2^)–H bond insertion,^11^ resulting in the six-membered rhodacyclic complex **III** (path 1). Final reductive elimination will give the desired 1*H*-indazole **2a** and Rh(i), which can be oxidized by Cu(ii) to regenerate the active Rh(iii) catalyst. However, another mechanism, which involves C=N bond insertion^12^ rather than C–H bond insertion, is also possible at this stage (path 2). The resulting polar C(aryl)–Rh bond in the first step can serve as a nucleophilic aryl source and undergo nucleophilic addition to the C=N bond, forming the N–Rh species **IV**. Under the basic conditions, the N–Rh species then undergoes β-H elimination to furnish the desired product **2a**.

**Fig. 1:**
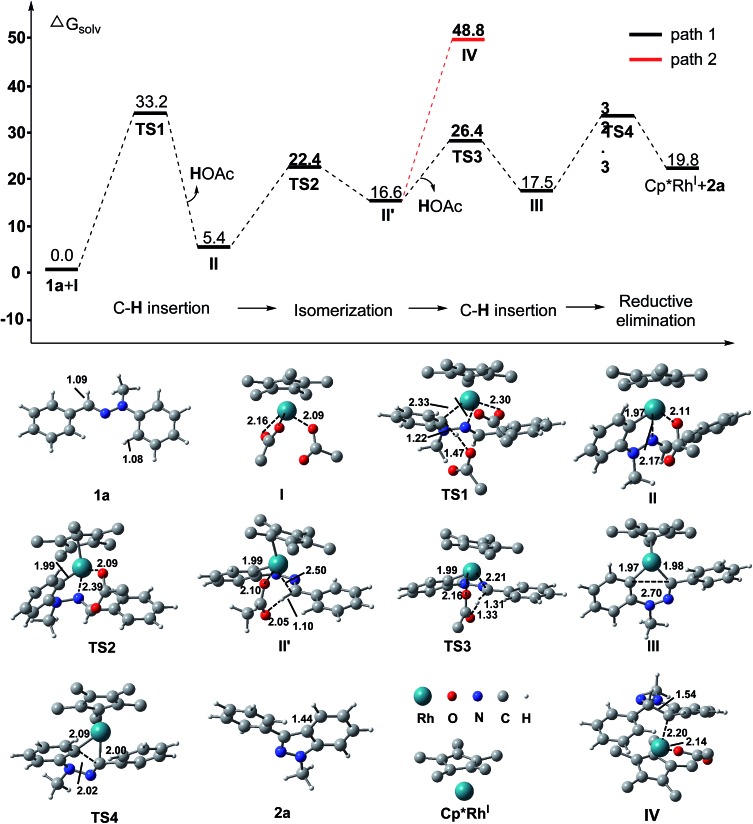
Computed Gibbs free energy (in kcal mol^–1^) profile for the reaction between the hydrazone **1a** and active catalyst **I** in the solvent (1,2-dichloroethane). Distances are in Å.

Furthermore, density functional theory (DFT) calculations were performed to investigate the detailed mechanism of this Rh(iii)-catalyzed oxidative C–H/C–H cross coupling reaction; two possible reaction pathways are proposed in Scheme 4, and the free energy profiles are shown in Fig. 1 (see ESI† for details). The active catalyst **I** and hydrazone **1a** were chosen as the model system. First, the coordination of the nitrogen atom of the C=N bond in **1a** to the Rh center and subsequent C(aryl)–H activation^8^ generates the five-membered rhodacyclic intermediate **II** *via* **TS1**, with a barrier of 33.3 kcal mol^–1^. The formation of intermediate **II** is endothermic by 5.4 kcal mol^–1^, which is consistent with the deuterium labeling experiment (a reversible C–H bond metalation). Then, the N–N bond rotation of intermediate **II** forms the rotation isomer **II′** *via* **TS2**, with a barrier of 22.4 kcal mol^–1^. Subsequently, the second C(aldehyde)–H bond activation of the **II′** intermediate assisted by an acetate yields the 16-electron six-membered rhodacyclic complex **III** *via* **TS3** (with a barrier of 26.4 kcal mol^–1^), releasing an acetic acid molecule. Finally, the reductive elimination of the intermediate **III** gives the desired 1*H* indazole (**2a**) and Rh(i) intermediate (Cp*Rh(i)) *via* **TS4**, with a barrier of 32.3 kcal mol^–1^ (path 1, black lines). The generation of Cp*Rh(i) and **2a** is endothermic by 19.8 kcal mol^–1^ (with respect to the active catalyst **I** and reactant **1a**). This result is consistent with the observed fact that the stoichiometric amount of oxidant (Cu(OAc)_2_) and base (K_2_CO_3_) were required for promoting this reaction. In addition to path 1, path 2 which involves the nucleophilic addition of the C–Rh bond to the C=N bond of species **II** ^11^ is also possible at this stage (as shown in Scheme 4, path 2). However, all attempts that tried to locate the transition state of the intramolecular nucleophilic addition (or C=N insertion) failed. Nevertheless, we performed a relaxed potential energy scan by fixing the C–C distance at a series of values to estimate the approximate barrier (as shown in Fig. S1†). The generation of the C=N bond insertion intermediate **IV** is endothermic by 48.8 kcal mol^–1^ (path 2, red lines), which suggests that path 2 can be excluded. Therefore, path 1 which involves the C(aryl)–H metalation/C(aldehyde)–H insertion/reductive elimination sequence is more responsible for the Rh(iii)-catalyzed C–H/C–H cross coupling reaction.

## Conclusions

In conclusion, an intramolecule directing group strategy has been successfully applied for the C(aldehyde)–H functionalization of aldehyde hydrazones for the first time. Highly functionalized 1*H*-indazoles could be directly accessed from easily available aldehyde phenylhydrazones *via* Rh(iii)-catalyzed oxidative C–H/C–H cross coupling. The reaction is scalable and various 1*H*-indazoles can be afforded in moderate to high yields with good functional-group compatibility. A mechanism study indicates this distinctive C–H/C–H cross coupling was enabled by a C(aryl)–H metalation/C(aldhyde)–H insertion/reductive elimination sequence. We believe this new strategy for the C(sp^2^)–H functionalization of aldehyde hydrazones will be extended to the concise synthesis of other important heterocyclic skeletons.
