# Supplementary material for: Rh(iii)-catalyzed double C–H activation of aldehyde hydrazones: a route for functionalized 1H-indazole synthesis
Source: Chem Sci. 2016 Oct 7;8(2):1303–8. doi: 10.1039/c6sc03888c (PMC5460599; doi:10.1039/c6sc03888c)

Electronic Supplementary Material (ESI) for Chemical Science.
This journal is © The Royal Society of Chemistry 2016

Rh(III)-catalyzed double C-H activation of aldehyde hydrazones: en route to functionalized 1*H*-Indazole synthesis

Pan Xu, Guoqiang Wang, Zhongkai Wu, Shuhua li*, Chengjian Zhu*

Table of Contents

1 General Information **2**

2 General Procedure for the Aldehyde Hydrazones **2**

3 General Procedure for the 1*H*-indazoles **12**

4 X-ray Structure Characterization **24**

5 Optimization of the Reaction Conditions **26**

6 Deuterium Labeling Experiment **27**

7 Computational Details of Mechanistic Studies **30**

8 Copies of ^{1}H NMR, ^{13}C NMR **62**

1. General Information

All reactions were carried out under an atmosphere of argon atmosphere in flame-dried glassware unless otherwise noted. Reactions were monitored by TLC on silica gel plates (GF254), and the analytical thin-layer chromatography (TLC) was performed on precoated, glass-backed silica gel plates. ^{1}H NMR, ^{13}C NMR spectra were recorded on on a Bruker AVANCE III–400 spectrometer at room temperature. Chemical shifts (δ) are reported in ppm downfield from tetramethylsilane. Abbreviations for signal couplings are: s, singlet; d, doublet; t, triplet; m, multiplet. The high and low resolution mass spectra were recorded in a positive and negative ion mode on a hybrid quadrupole time-off light mass spectrometer with an Electrospray Ionization (ESI) ion source. Hydrazine was obtained from commercial sources or prepared following the previous literature.[1] Solvents and all other reagents were obtained from commercial sources and used as received.

2. General Procedure for the Aldehyde Hydrazones

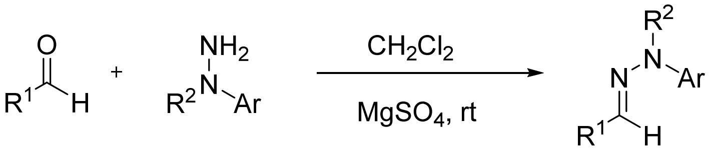

1

General procedure **A**: A mixture of hydrazine (2.4 mmol), aldehyde (2.0 mmol) and anhydrous $MgSO_4$ (0.5 g) in CH_2Cl_2 (10 mL) was stirred overnight at room temperature. After filtration of $MgSO_4$, CH_2Cl_2 was removed under reduced pressure and the residue was subjected to column chromatography to give the desired product **1**.

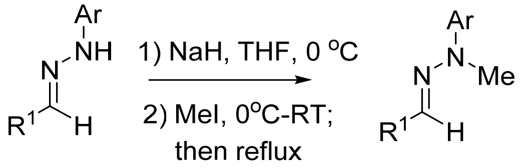

1

General procedure **B**[2]: To a solution of aldehyde hydrazone (5 mmol) in dry THF (20 mL) was added NaH (60%, 50 mmol) at 0 °C. The mixture was stirred at 0 °C for 15 min, and then MeI (7.5 mmol) was added dropwise. The reaction mixture was stirring at room temperature for 3 h then refluxed for another 2 h. The reaction mixture was cooled to the room temperature, and the solvent was removed under reduced pressure with a rotary evaporator. The residue was diluted with water, extracted with ethyl acetate, and dried over $MgSO_4$. After removal of the solvent, the residue was purified

[1] a) N. Giuseppone, J. L. Schmitt, E. Schwartz, J. M. Lehn, *J. Am. Chem. Soc.* **2005**, *127*, 5528; b) R. F. Smith, L. A. Dennis, W. J. Ryan, G. Rodriguez, K. A. Brophy, *J. Heterocycl. Chem.* **1992**, *29*, 181; c) S. Zhou, J. Wang, F. Zhang, C. Song, J. Zhu, *Org. Lett.* **2016**, *18*, 2427.

[2] S. D. Sharma, S. B. Pandhi, *J. Org. Chem.* **1990**, *55*, 2196.

by silica gel column chromatography to give the product **1**.

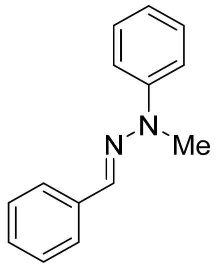

1a

The title compound **1a** was prepared according to the general procedure **A** as slightly yellow solid, m.p. 106-107 °C. ^{1}H NMR (400 MHz, $CDCl_3$): δ = 7.69 (d, *J* = 7.6 Hz, 2H), 7.48 (s, 1H), 7.39-7.30 (m, 6H), 7.27-7.24 (m, 1H), 6.93 (t, *J* = 7.2 Hz, 1H), 3.41 (s, 3H); ^{13}C NMR (100 MHz, CDCl3): δ 147.9, 136.8, 131.9, 129.1, 128.6, 127.7, 126.1, 120.6, 115.3, 33.1 ppm. MS (ESI, *m/z*): 211.05([M+H$^+$]).

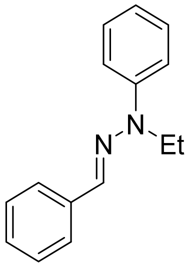

1b

The title compound **1b** was prepared according to the general procedure **A** as yellow solid, m.p. 50-51 °C. ^{1}H NMR (400 MHz, $CDCl_3$): δ = 7.70-7.68 (m, 2H), 7.52(s, 1H), 7.37-7.27 (m, 6H), 7.26-7.19 (m, 1H), 6.93-6.90 (m, 1H), 3.97 (q, *J* = 7.2 Hz, 2H), 3.41 (t, *J* = 7.2 Hz, 3H); ^{13}C NMR (100 MHz, CDCl3): δ 146.9, 137.0, 131.0, 129.2, 128.7, 127.8, 126.1, 120.4, 114.7, 39.5, 10.2 ppm. MS (ESI, *m/z*): 225.05([M+H$^+$]).

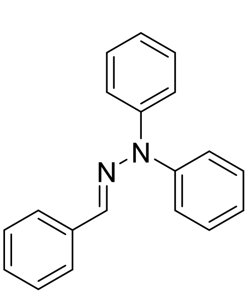

1c

The title compound **1c** was prepared according to the general procedure **A** as white solid, m.p. 126-127 °C. ^{1}H NMR (400 MHz, $CDCl_3$): δ = 7.60 (d, *J* = 7.6 Hz, 2H), 7.42 (s, *J* = 7.6 Hz, 4H), 7.33 (t, *J* = 7.6 Hz, 2H), 7.27-7.24 (m, 1H), 7.21-7.15 (m, 6H), 7.15 (s, 1H); ^{13}C NMR (100 MHz, CDCl3): δ 143.7, 136.2, 135.5, 129.8, 128.6, 128.1, 126.3, 124.5, 122.6 ppm. MS (ESI, *m/z*): 273.05([M+H$^+$]).

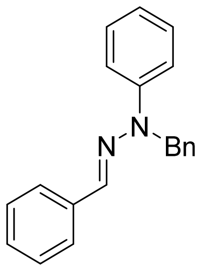

1d

The title compound **1d** was prepared according to the general procedure **A** as white solid, m.p. 111-112 ^{o}C. ^{1}H NMR (400 MHz, $CDCl_3$): δ = 7.62 (d, *J* = 8.0 Hz, 2H), 7.31 (d, *J* = 8.0 Hz, 2H),7.39 (s, 1H), 7.36-7.30 (m, 6H), 7.28-7.25 (m, 3H), 7.23-7.22 (m, 1H), 6.95 (t, *J* = 7.2 Hz, 1H), 5.19 (s, 2H); ^{13}C NMR (100 MHz, CDCl3): δ 147.9, 136.6, 135.7, 132.5, 129.2, 129.0, 128.6, 127.9, 127.3, 126.2, 126.0, 120.8, 114.8, 50.5 ppm. MS (ESI, *m/z*): 287.05([M+H^{+}]).

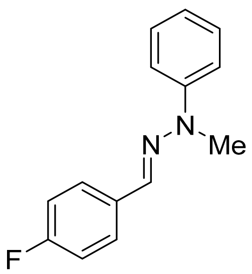

1e

The title compound **1e** was prepared according to the general procedure **A** as white solid, m.p. 108-109 ^{o}C. ^{1}H NMR (400 MHz, $CDCl_3$): δ = 7.68-7.65 (m, 2H), 7.46 (s, 1H), 7.36 (d, *J* = 8.0 Hz, 2H), 7.34-7.30 (m, 2H), 7.06 (t, *J* = 8.4 Hz, 2H), 6.94 (t, *J* = 7.2 Hz, 1H); ^{13}C NMR (100 MHz, CDCl3): δ 162.5 (d, *J* = 245.5 Hz), 147.8, 133.0 (d, *J* = 3.2 Hz), 130.7, 129.1, 127.6 (d, *J* = 6.8 Hz), 120.7, 115.5 (d, *J* = 21.6 Hz), 115.3, 33.1 ppm. MS (ESI, *m/z*): 229.05([M+H^{+}]).

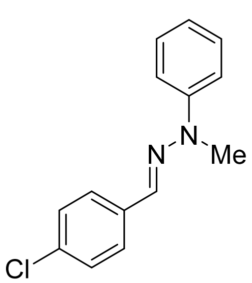

1f

The title compound **1f** was prepared according to the general procedure **A** as yellow solid, m.p. 110-111 ^{o}C. ^{1}H NMR (400 MHz, $CDCl_3$): δ = 7.63-7.61 (m, 2H), 7.43 (s, 1H), 7.38-7.30 (m, 6H), 6.95 (t, *J* = 7.2 Hz, 1H), 7.21-7.19 (m, 1H), 6.97-6.95 (m, 1H), 3.42 (s, 3H); ^{13}C NMR (100 MHz, $CDCl_3$): δ 147.7, 135.4, 133.2, 130.4, 129.1, 128.8, 127.1, 120.9, 115.4, 33.2 ppm. MS (ESI, *m/z*): 245.00([M+H^{+}]).

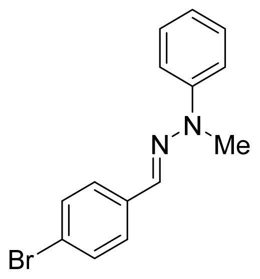

1g

The title compound **1g** was prepared according to the general procedure **A** as white solid, m.p. 127-128 °C. ^{1}H NMR (400 MHz, $CDCl_3$): δ = 7.57-7.54 (m, 2H), 7.49-7.47 (m, 2H), 7.41 (s, 1H), 7.38-7.31 (m, 4H), 6.95 (d, *J* = 7.2 Hz, 1H), 3.42 (s, 3H); ^{13}C NMR (100 MHz, $CDCl_3$): δ 147.7, 135.8, 131.7, 130.4, 129.1, 127.5, 121.3, 120.9, 115.4, 33.2 ppm. MS (ESI, *m/z*): 288.95([M+H$^+$]).

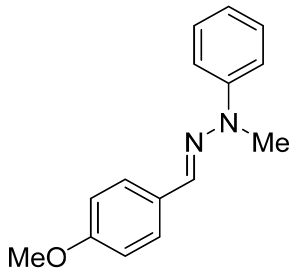

1h

The title compound **1h** was prepared according to the general procedure **A** as white solid, m.p. 116-117 °C. ^{1}H NMR (400 MHz, $CDCl_3$): δ = 7.64 (d, *J* = 8.8 Hz, 2H), 7.48 (s, 1H), 7.36 (d, *J* = 8.0 Hz, 2H), 7.31 (t, *J* = 8.0 Hz, 2H), 6.92-6.89 (m, 3H), 3.83 (s, 3H), 3.40 (s, 3H); ^{13}C NMR (100 MHz, $CDCl_3$): δ 159.5, 148.1, 131.9, 129.7, 129.0, 127.4, 120.2, 115.1, 114.1, 55.4, 33.0 ppm. MS (ESI, *m/z*): 241.05([M+H$^+$]).

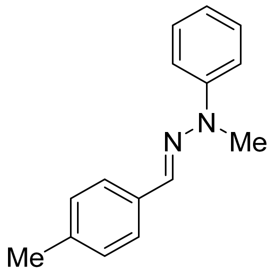

1i

The title compound **1i** was prepared according to the general procedure **A** as white solid, m.p. 121-122 °C. ^{1}H NMR (400 MHz, $CDCl_3$): *δ* = 7.59 (d, *J* = 8.0 Hz, 2H), 7.49 (s, 1H), 7.39-7.37 (m, 2H), 7.34-7.30 (m, 2H), 7.17 (d, *J* = 8.0 Hz, 2H), 6.92 (t, *J* = 7.2 Hz, 1H), 3.41 (s, 3H), 2.36 (s, 3H) ppm; ^{13}C NMR (100 MHz, $CDCl_3$): δ 148.0, 137.7, 134.1, 132.1, 129.3, 129.0, 126.0, 120.4, 115.2, 33.0, 21.4 ppm. MS (ESI, *m/z*): 225.05([M+H$^+$]).

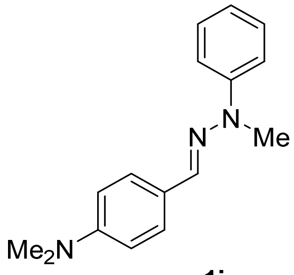

1j

The title compound **1j** was prepared according to the general procedure **A** as yellow solid, m.p. 149-150 °C. ^{1}H NMR (400 MHz, $CDCl_3$): δ = 7.61-7.58 (m, 2H), 7.49 (s, 1H), 7.37-7.35 (m, 2H), 7.32-7.28 (m, 2H), 6.89-6.86 (m, 1H), 6.74-6.72 (m, 2H), 3.39 (s, 3H), 2.99 (s, 6H) ppm; ^{13}C NMR (100 MHz, $CDCl_3$): δ 150.4, 148.3, 133.2, 129.0, 127.3, 125.3, 119.7, 114.8, 112.4, 40.5, 33.0 ppm. MS (ESI, *m/z*): 254.05([M+H$^+$]).

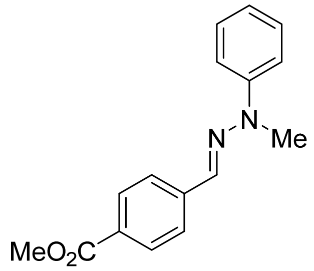

1k

The title compound **1k** was prepared according to the general procedure **A** as yellow solid, m.p. 105-106 °C. ^{1}H NMR (400 MHz, $CDCl_3$): δ = 8.03 (d, J = 7.6 Hz, 2H), 7.73 (d, J = 8.0 Hz, 2H), 7.48 (s, 1H), 7.41-7.39 (m, 2H), 7.36-7.32 (m, 2H), 7.00-6.98 (m, 1H), 6.74-6.72 (m, 2H), 3.92 (s, 3H), 3.45 (s, 3H) ppm; ^{13}C NMR (100 MHz, $CDCl_3$): δ 167.1, 147.6, 141.2, 130.3, 130.0, 129.1, 128.7, 125.7, 121.3, 115.6, 52.1, 33.3 ppm. MS (ESI, *m/z*): 269.05([M+H$^+$]).

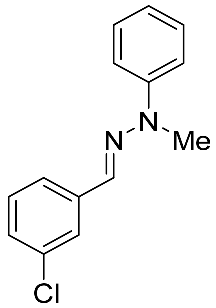

1l

The title compound **1l** was prepared according to the general procedure **A** as slightly yellow solid, m.p. 69-70 °C. ^{1}H NMR (400 MHz, $CDCl_3$): δ = 7.70 (t, J = 2.0 Hz, 1H), 7.52 (d, J = 7.6 Hz, 2H), 7.40-7.30 (m, 5H), 7.28-7.25 (m, 1H), 7.23-7.20 (m, 1H), 3.42 (s, 3H) ppm; ^{13}C NMR (100 MHz, $CDCl_3$): δ 147.6, 138.7, 134.6, 130.1, 129.8, 129.1, 127.5, 125.7, 124.3, 121.1, 115.5, 33.3 ppm. MS (ESI, *m/z*): 245.00([M+H$^+$]).

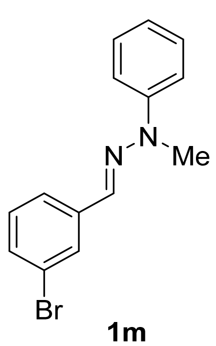

1m

The title compound **1m** was prepared according to the general procedure **A** as brown solid, m.p. 87-88 °C. ^{1}H NMR (400 MHz, $CDCl_3$): δ = 7.83 (s, 1H), 7.53 (d, J = 8.0 Hz, 1H), 7.36-7.30 (m, 6H), 7.19 (t, J = 8.0 Hz, 1H), 6.96-6.94 (m, 1H), 3.36 (s, 3H) ppm; ^{13}C NMR (100 MHz, $CDCl_3$): δ 147.6, 139.0, 130.4, 130.1, 129.9, 129.1, 128.6, 124.8, 122.9, 121.1, 115.5, 33.3 ppm. MS (ESI, *m/z*): 288.95([M+H^{+}]).

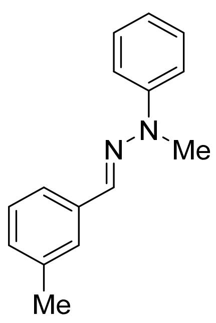

1n

The title compound **1n** was prepared according to the general procedure **A** as yellow solid, m.p. 87-88 °C. ^{1}H NMR (400 MHz, $CDCl_3$): δ = 7.53 (s, 1H), 7.48 (d, J = 8.4 Hz, 2H), 7.38 (d, J = 8.0 Hz, 2H), 7.34-7.30 (m, 2H), 7.25 (d, J = 6.4 Hz, 1H), 7.08 (d, J = 7.6 Hz, 1H), 6.93 (t, J = 7.2 Hz, 1H), 3.41 (s, 3H), 3.39 (s, 3H) ppm; ^{13}C NMR (100 MHz, $CDCl_3$): δ 148.0, 138.2, 136.7, 132.2, 129.1, 128.6, 128.5, 126.6, 123.5, 120.5, 115.3, 33.1, 21.5 ppm. MS (ESI, *m/z*): 225.05([M+H^{+}]).

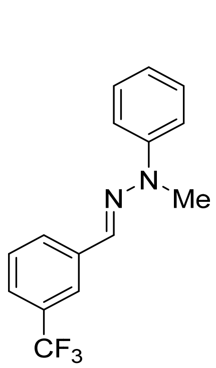

1o

The title compound **1o** was prepared according to the general procedure **A** as slightly yellow solid, m.p. 88-89 °C. ^{1}H NMR (400 MHz, $CDCl_3$): δ = 7.91 (s, 1H), 7.86 (d, J = 7.2 Hz, 1H), 7.50-7.46 (m, 3H), 7.40-7.33 (m, 4H), 7.00-6.98 (m, 1H), 3.44 (s, 3H) ppm; ^{13}C NMR (100 MHz, $CDCl_3$): δ 147.6, 137.7, 131.0 (q, J = 31.2 Hz), 130.0,

129.2, 129.0, 128.9, 124.2 (q, *J* = 270.8 Hz), 123.9 (q, *J* = 3.9 Hz), 123.9 (q, *J* = 4.0 Hz), 121.2, 115.6, 33.3 ppm. MS (ESI, *m/z*): 279.00([M+H$^+$]).

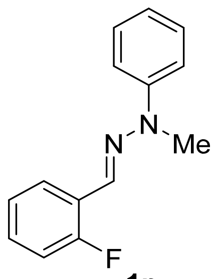

1p

The title compound **1p** was prepared according to the general procedure **A** as white solid, m.p. 81-82 ^{o}C. ^{1}H NMR (400 MHz, $CDCl_3$): δ = 8.04-8.00 (m, 1H), 7.66 (s, 1H), 7.39-7.37 (m, 2H), 7.37-7.30 (m, 2H), 7.23-7.18 (m, 1H), 7.14 (t, *J* = 7.2 Hz), 7.07-7.02 (m, 1H), 6.96-6.92 (m, 1H), 3.42 (s, 3H) ppm; ^{13}C NMR (100 MHz, $CDCl_3$): δ 160.6 (d, *J* = 246.6 Hz), 147.8, 129.1, 128.8 (d, *J* = 8.2 Hz), 125.8 (d, *J* = 4.2 Hz), 124.6 (d, *J* = 4.9 Hz), 124.5, 124.2 (d, *J* = 2.5 Hz), 120.9, 115.6, 115.4, 33.2 ppm. MS (ESI, *m/z*): 229.05([M+H$^+$]).

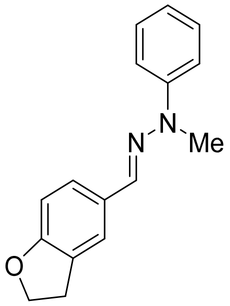

1q

The title compound **1q** was prepared according to the general procedure **A** as brown solid, m.p. 120-121 ^{o}C. ^{1}H NMR (400 MHz, $CDCl_3$): δ = 7.64 (s, 1H), 7.45 (s, 1H), 7.34 (d, *J* = 7.6 Hz, 3H), 7.32-7.28 (m, 2H), 6.89 (t, *J* = 7.2 Hz, 1H), 6.76 (d, *J* = 8.0 Hz, 1H), 4.57 (t, *J* = 8.8 Hz, 2H), 3.35 (s, 3H), 3.21 (t, *J* = 8.8 Hz, 2H) ppm; ^{13}C NMR (100 MHz, $CDCl_3$): δ 160.2, 148.1, 132.5, 129.8, 129.1, 127.8, 127.1, 122.1, 120.1, 115.0, 109.2, 71.6, 33.0, 29.6 ppm. MS (ESI, *m/z*): 253.05([M+H$^+$]).

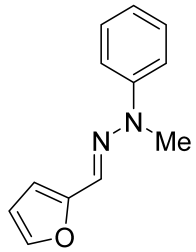

1r

The title compound **1r** was prepared according to the general procedure **A** as slightly yellow solid, m.p. 54-55 ^{o}C. ^{1}H NMR (400 MHz, $CDCl_3$): δ = 7.43-7.24 (m, 6H), 6.93 (t, *J* = 6.4 Hz, 1H), 6.53 (s, 1H), 6.45-6.44 (m, 1H), 3.36 (s, 3H) ppm; ^{13}C NMR (100 MHz, $CDCl_3$): δ 152.4, 147.6, 142.2, 129.1, 122.8, 120.9, 115.6, 111.5, 107.6, 33.3

ppm. MS (ESI, *m/z*): 201.00([M+H$^+$]).

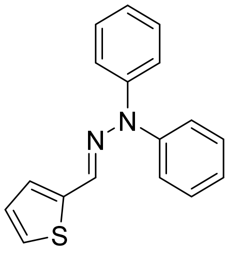

1s

The title compound **1s** was prepared according to the general procedure **A** as slightly green solid, m.p. 129-130 ^{o}C. ^{1}H NMR (400 MHz, CDCl$_3$): δ = 7.43-7.39 (m, 4H), 7.30 (s, 1H), 7.21-7.17 (m, 7H), 6.95-6.94 (m, 1H), 6.91-6.90 (m, 1H) ppm; ^{13}C NMR (100 MHz, CDCl$_3$): δ 143.4, 141.9, 130.5, 129.9, 127.2, 126.4, 125.5, 124.7, 122.5 ppm. MS (ESI, *m/z*): 278.95([M+H$^+$]).

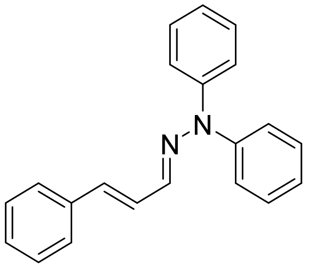

1t

The title compound **1t** was prepared according to the general procedure **A** as slightly green solid, m.p. 130-131 ^{o}C. ^{1}H NMR (400 MHz, CDCl$_3$): δ = 7.43-7.38 (m, 6H), 7.31 (t, J = 7.6 Hz, 2H), 7.25-7.14 (m, 7H), 7.09-7.04 (m, 2H), 6.53-6.45 (m, 1H) ppm; ^{13}C NMR (100 MHz, CDCl$_3$): δ 143.5, 138.3, 137.0, 134.1, 129.8, 128.7, 127.8, 126.9, 126.4, 124.6, 122.5 ppm. MS (ESI, *m/z*): 299.05([M+H$^+$]).

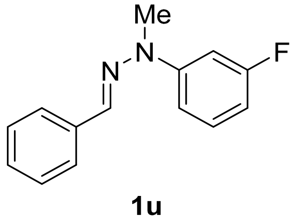

1u

The title compound **1u** was prepared according to the general procedure **B** as pink solid, m.p. 124-125 ^{o}C. ^{1}H NMR (400 MHz, CDCl$_3$): δ = 7.68 (d, J = 7.6 Hz, 2H), 7.50 (s, 1H), 7.37 (t, J = 7.6 Hz, 2H), 7.28 (d, J = 7.2 Hz, 1H), 7.24-7.15 (m, 2H), 7.05 (dd, J = 2.0 Hz, 8.4 Hz, 1H), 6.60 (dt, J = 2.4 Hz, 8.0 Hz, 1H), 3.37 (s, 3H) ppm; ^{13}C NMR (100 MHz, CDCl$_3$): δ 163.8 (d, J = 241.2 Hz), 139.5 (d, J = 11.1 Hz), 136.4, 132.9, 130.0 (d, J = 9.7 Hz), 128.7, 128.2, 126.3, 110.0 (d, J = 2.3 Hz), 106.8 (d, J = 21.4 Hz), 102.4 (d, J = 26.9 Hz), 32.8 ppm. MS (ESI, *m/z*): 229.05([M+H$^+$]).

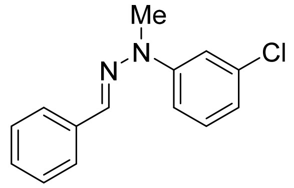

1v

The title compound **1v** was prepared according to the general procedure **B** as white solid, m.p. 100-101 °C. ^{1}H NMR (400 MHz, $CDCl_3$): δ = 7.69 (d, J = 8.4 Hz, 2H), 7.51 (s, 1H), 7.40-7.36 (m, 3H), 7.28 (t, J = 7.6 Hz, 1H), 7.21-7.20 (m, 2H), 6.89-6.86 (m, 1H), 6.60 (dt, J = 2.4 Hz, 8.0 Hz, 1H), 3.38 (s, 3H) ppm; ^{13}C NMR (100 MHz, $CDCl_3$): δ 148.9, 136.3, 134.9, 133.1, 130.0, 128.7, 128.2, 126.3, 120.2, 115.2, 112.9, 32.8 ppm. MS (ESI, *m/z*): 245.00([M+H$^+$]).

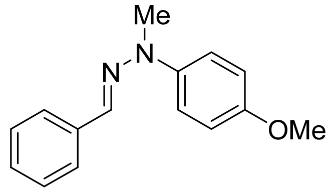

1w

The title compound **1w** was prepared according to the general procedure **B** as yellow solid, m.p. 131-132 °C. ^{1}H NMR (400 MHz, $CDCl_3$): δ = 7.67 (d, J = 7.6 Hz, 2H), 7.42 (s, 1H), 7.35 (t, J = 8.0 Hz, 2H), 7.30-7.24 (m, 3H), 6.91-6.88 (m, 2H), 3.80(s, 3H), 3.39 (s, 3H) ppm; ^{13}C NMR (100 MHz, $CDCl_3$): δ 154.3, 142.4, 137.0, 131.2, 128.6, 127.4, 125.9, 117.2, 114.4, 55.7, 34.3 ppm. MS (ESI, *m/z*): 241.05([M+H$^+$]).

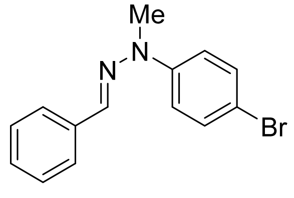

1x

The title compound **1x** was prepared according to the general procedure **B** as yellow solid, m.p. 104-105 °C. ^{1}H NMR (400 MHz, $CDCl_3$): δ = 7.68 (d, J = 7.2 Hz, 2H), 7.50 (s, 1H), 7.41-7.35 (m, 4H), 7.30-7.28 (m, 1H), 7.26-7.24 (m, 2H), 3.38 (s, 3H) ppm; ^{13}C NMR (100 MHz, $CDCl_3$): δ 146.9, 136.4, 132.7, 131.8, 128.7, 128.1, 126.2, 116.7, 112.8, 32.9 ppm. MS (ESI, *m/z*): 290.95([M+H$^+$]).

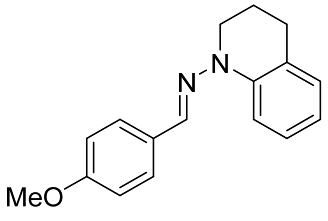

3a

The title compound **3a** was prepared according to the general procedure **A** as yellow solid, m.p. 101-102 °C. ^{1}H NMR (400 MHz, $CDCl_3$): δ = 7.75 (d, J = 7.6 Hz, 1H), 7.64-7.61 (m, 2H), 7.47 (s, 1H), 7.20-7.17 (m, 1H), 6.99 (d, J = 6.4 Hz, 1H),

6.91-6.88 (m, 2H), 6.76 (dt, J = 1.2 Hz, 7.6 Hz, 1H), 3.81 (s, 3H), 3.61 (t, J = 6.4 Hz, 2H), 2.73 (t, J = 6.4 Hz, 2H), 2.16-2.10 (m, 2H) ppm; ^{13}C NMR (100 MHz, $CDCl_3$): δ 159.4, 143.2, 130.5, 130.0, 128.2, 127.4, 127.3, 123.4, 119.2, 114.2, 114.1, 55.4, 44.7, 27.2, 22.0 ppm. MS (ESI, *m/z*): 267.05 ([M+H$^+$]).

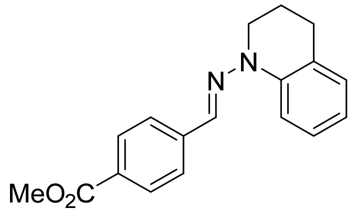

3b

The title compound **3b** was prepared according to the general procedure **A** as yellow solid, m.p. 129-130 °C. ^{1}H NMR (400 MHz, $CDCl_3$): δ = 8.03-8.00 (m, 2H), 7.77 (d, J = 8.0 Hz, 1H), 7.73-7.71 (m, 2H), 7.48 (s, 1H), 7.24-7.20 (m, 1H), 7.02 (d, J = 7.6 Hz, 1H), 7.02 (dt, J = 1.2 Hz, 7.6 Hz, 1H), 3.91 (s, 3H), 3.65 (t, J = 6.4 Hz, 2H), 2.75 (t, J = 6.4 Hz, 2H), 2.19-2.13 (m, 2H) ppm; ^{13}C NMR (100 MHz, $CDCl_3$): δ 167.1, 142.6, 141.5, 130.0, 129.0, 128.6, 128.3, 127.5, 125.6, 124.1, 120.3, 114.7, 52.1, 45.1, 27.0, 21.9 ppm. MS (ESI, *m/z*): 295.05([M+H$^+$]).

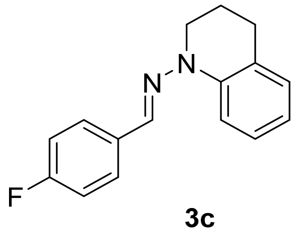

3c

The title compound **3c** was prepared according to the general procedure **A** as white solid, m.p. 113-114 °C. ^{1}H NMR (400 MHz, $CDCl_3$): δ = 7.73 (d, J = 8.0 Hz, 1H), 7.66-7.63 (m, 2H), 7.44 (s, 1H), 7.22-7.19 (m, 1H), 7.06-7.00 (m, 3H), 6.79 (t, J = 7.6 Hz, 1H), 3.60 (t, J = 6.0 Hz, 2H), 2.73 (t, J = 6.0 Hz, 2H), 2.16-2.12 (m, 2H) ppm; ^{13}C NMR (100 MHz, $CDCl_3$): δ 162.4 (d, J = 244.5 Hz), 142.9, 133.2 (d, J = 2.6 Hz), 129.3, 128.2, 127.5, 127.4 (d, J = 1.9 Hz), 123.6, 119.6, 115.6, 115.4, 114.3, 44.8, 27.0, 21.9 ppm. MS (ESI, *m/z*): 255.05([M+H$^+$]).

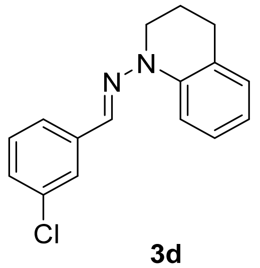

3d

The title compound **3d** was prepared according to the general procedure **A** as yellow solid, m.p. 56-57 °C. ^{1}H NMR (400 MHz, $CDCl_3$): δ = 7.74 (d, J = 8.4 Hz, 1H), 7.68 (t, J = 2.0 Hz, 1H), 7.49 (dt, J = 1.2 Hz, 7.6 Hz, 1H), 7.37 (s, 1H), 7.27-7.17 (m, 3H), 7.00 (d, J = 7.2 Hz, 1H), 6.81 (dt, J = 1.2 Hz, 7.2 Hz, 1H), 3.59 (t, J = 6.4 Hz, 2H), 2.72 (t, J = 6.4 Hz, 2H), 2.16-2.09 (m, 2H) ppm; ^{13}C NMR (100 MHz, $CDCl_3$): δ

142.7, 139.0, 134.6, 129.8, 128.7, 128.4, 127.5, 1274, 125.6, 124.3, 123.9, 120.0, 114.6, 45.0, 27.0, 21.9 ppm. MS (ESI, *m/z*): 271.00([M+H$^+$]).

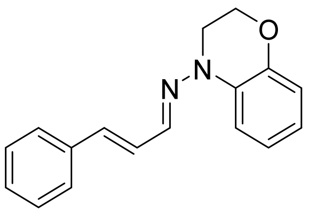

3e

The title compound **3e** was prepared according to the general procedure **A** as slightly green solid, m.p. 106-107 °C. ^{1}H NMR (400 MHz, $CDCl_3$): δ = 7.64 (d, J = 8.4 Hz, 1H), 7.45 (d, J = 8.0 Hz, 2H), 7.39 (d, J = 8.8 Hz, 1H), 7.34 (t, J = 7.2 Hz, 2H), 7.25 (t, J = 7.2 Hz, 1H), 7.12-7.05 (m, 1H), 6.98-6.94 (m, 1H), 6.86 (d, J = 8.0 Hz, 1H), 6.81-6.71 (m, 2H), 4.37 (t, J = 4.4 Hz, 2H), 3.71 (t, J = 4.4 Hz, 2H) ppm; ^{13}C NMR (100 MHz, $CDCl_3$): δ 143.5, 137.0, 134.9, 133.8, 132.6, 128.8, 127.9, 127.1, 126.5, 122.5, 120.5, 116.8, 114.4, 63.5, 43.0 ppm. MS (ESI, *m/z*): 265.05

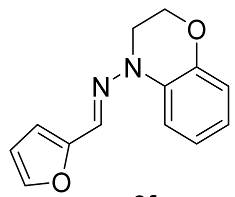

3f

The title compound **3f** was prepared according to the general procedure **A** as yellow solid, m.p. 75-76 °C. ^{1}H NMR (400 MHz, $CDCl_3$): δ = 7.69 (dd, J = 1.6 Hz, 8.0 Hz, 1H), 7.46-7.45 (m, 1H), 7.39 (s, 1H), 6.99-6.94 (m, 1H), 6.87-6.85 (m, 1H), 6.81-6.77 (m, 1H), 6.57 (d, J = 3.2 Hz, 1H), 6.46-6.45 (m, 1H), 4.38 (t, J = 4.8 Hz, 2H), 3.70 (t, J = 4.8 Hz, 2H) ppm; ^{13}C NMR (100 MHz, $CDCl_3$): δ 151.8, 143.5, 142.6, 132.6, 123.0, 122.5, 120.6, 116.7, 114.7, 111.6, 108.3, 63.5, 42.9 ppm. MS (ESI, *m/z*): 229.05([M+H$^+$]).

3. General procedure for Rh(III)-Catalyzed C-H/C-H bonds cross coupling of aldehyde hydrazone

An oven-dried Schlenk tube (10 mL) was equipped with a magnetic stir bar, **1** or **3** (0.2 mmol), $[RhCp^*Cl_2]_2$ (0.025 equiv, 0.005 mmol, 3.1 mg), AgOTf (0.10 equiv, 0.02 mmol, 5.1 mg), $Cu(OAc)_2$ (2.0 equiv, 0.40 mmol, 72 mg), K_2CO_3 (1.0 equiv, 0.20 mmol, 27 mg). The flask was evacuated and backfilled with Argon for 3 times. 0.5 mL DCE was added with syringe under Argon. The resulting solution was stirred at 120°C for 24 h. Then the mixture was concentrated under vacuum to remove DCE, and the residue was purified by chromatography on silica gel to afford the **2** or **4**.

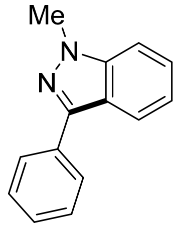

2a

Following general procedure, the crude product was purified by flash column chromatography (15:1 petroleum ether: ethyl acetate) to afford **2a** (33.3 mg, 80% yield) as yellow solid. m.p. 69-70 °C. Reaction time: 24h. ^{1}H NMR (400 MHz, $CDCl_3$): δ = 7.92 (d, J = 8.0 Hz, 1H), 7.87 (d, J = 8.0 Hz, 2H), 7.41 (t, J = 7.6 Hz, 2H), 7.33-7.28 (m, 3H), 7.15-7.10 (m, 1H), 4.03 (s, 3H) ppm; ^{13}C NMR (100 MHz, $CDCl_3$): δ 143.8, 141.5, 133.7, 128.8, 127.9, 127.4, 126.3, 121.6, 121.4, 121.0, 109.2, 35.6 ppm. HRMS (ESI) m/z calcd for $C_{14}H_{13}N_2$ $[M+H]^+$: 209.1073, found: 209.1073.

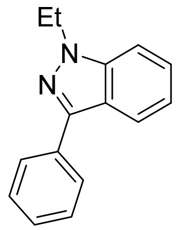

2b

Following general procedure, the crude product was purified by flash column chromatography (20:1 petroleum ether: ethyl acetate) to afford **2b** (41.4 mg, 92% yield) as slight yellow oil. Reaction time: 24h. ^{1}H NMR (400 MHz, $CDCl_3$): δ = 8.00 (d, J = 8.0 Hz, 1H), 7.97-7.95 (m, 2H), 7.49 (t, J = 8.0 Hz, 2H), 7.42-7.35 (m, 3H), 7.20-7.16 (m, 1H), 4.46 (q, J = 7.6 Hz, 2H), 1.53 (t, J = 7.6 Hz, 3H); ^{13}C NMR (100 MHz, $CDCl_3$): δ 143.7, 140.5, 133.9, 128.9, 127.8, 127.5, 126.2, 121.8, 121.5, 120.9, 109.3, 43.9, 15.1 ppm. HRMS (ESI) m/z calcd for $C_{15}H_{15}N_2$ $[M+H]^+$: 223.1230, found: 223.1231.

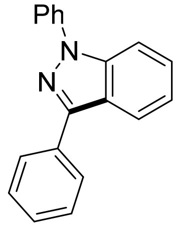

2c

Following general procedure, the crude product was purified by flash column chromatography (15:1 petroleum ether: ethyl acetate) to afford **2c** (51.2 mg, 95% yield) as slight yellow solid. m.p. 110-111 °C. Reaction time: 24h. ^{1}H NMR (400 MHz, $CDCl_3$): δ = 8.08-8.03 (m, 3H), 7.80-7.75 (m, 3H), 7.55-7.50 (m, 4H), 7.45-7.40 (m, 2H), 7.37-7.32 (m, 1H), 7.28-7.24 (m, 1H) ppm; ^{13}C NMR (100 MHz, $CDCl_3$): δ 146.2, 140.4, 140.2, 133.3, 129.5, 128.9, 128.3, 127.8, 127.2, 126.7, 123.1,

122.0, 121.7, 117.9, 110.7 ppm. HRMS (ESI) m/z calcd for $C_{19}H_{15}N_2$ $[M+H]^+$: 271.1230, found: 271.1231.

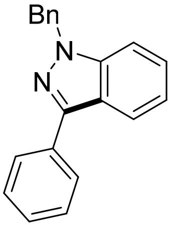

2d

Following general procedure, the crude product was purified by flash column chromatography (15:1 petroleum ether: ethyl acetate) to afford **2d** (44.5 mg, 78% yield) as slight yellow solid. m.p. 65-66 ^{o}C. Reaction time: 24h. ^{1}H NMR (400 MHz, $CDCl_3$): δ = 7.94-7.90 (m, 3H), 7.42-7.39 (m, 2H), 7.32-7.27 (m, 1H), 7.24-7.23 (m, 2H), 7.20-7.07 (m, 6H), 5.54 (s, 2H) ppm; ^{13}C NMR (100 MHz, $CDCl_3$): δ 144.2, 141.1, 137.0, 133.7, 128.9, 128.8, 128.0, 127.8, 127.6, 127.2, 126.5, 122.2, 121.5, 121.2, 109.7, 53.1 ppm. HRMS (ESI) m/z calcd for $C_{20}H_{17}N_2$ $[M+H]^+$: 285.1386, found: 285.1390.

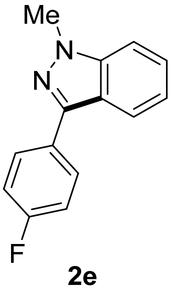

2e

Following general procedure, the crude product was purified by flash column chromatography (15:1 petroleum ether: ethyl acetate) to afford **2e** (30.1 mg, 67% yield) as white solid. m.p. 97-98 ^{o}C. Reaction time: 24h. ^{1}H NMR (400 MHz, $CDCl_3$): δ = 7.87-7.83 (m, 2H), 7.82-7.81 (m, 1H), 7.33-7.32 (m, 2H), 7.12-7.07 (m, 3H), 4.01 (s, 3H) ppm; ^{13}C NMR (100 MHz, $CDCl_3$): δ 162.6 (d, J = 245.4 Hz), 142.8, 141.4, 129.9 (d, J = 2.7 Hz), 129.0 (d, J = 8.3 Hz), 126.4, 121.5, 121.0 (d, J = 4.3 Hz), 115.9, 115.7, 109.3, 35.6 ppm. HRMS (ESI) m/z calcd for $C_{14}H_{12}FN_2$ $[M+H]^+$: 227.0979, found: 227.0979.

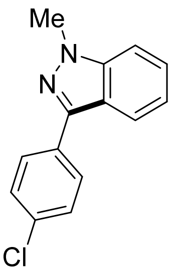

2f

Following general procedure, the crude product was purified by flash column chromatography (15:1 petroleum ether: ethyl acetate) to afford **2f** (32.0 mg, 66% yield) as yellow solid, m.p. 105-106 ^{o}C. Reaction time: 24h. ^{1}H NMR (400 MHz, $CDCl_3$): δ

= 7.95 (d, *J* = 8.0 Hz, 1H), 7.90-7.88 (m, 2H), 7.47-7.45 (m, 2H), 7.43-7.41 (m, 2H), 7.24-7.20 (m, 1H), 4.11 (s, 3H) ppm; ^{13}C NMR (100 MHz, $CDCl_3$): δ 142.5, 141.5, 133.6, 132.2, 129.0, 128.5, 126.4, 121.5, 121.2, 121.1, 109.4, 35.6 ppm. HRMS (ESI) m/z calcd for $C_{14}H_{12}ClN_2$ $[M+H]^+$: 243.0684, found: 243.0684.

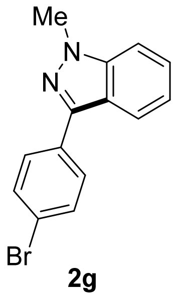

2g

Following general procedure, the crude product was purified by flash column chromatography (20:1 petroleum ether: ethyl acetate) to afford **2g** (34.3 mg, 60% yield) as slight yellow solid. m.p. 89-90 °C. Reaction time: 24h. ^{1}H NMR (400 MHz, $CDCl_3$): δ = 7.86 (d, *J* = 8.4 Hz, 1H), 7.75 (d, *J* = 8.4 Hz, 2H), 7.52 (d, *J* = 8.8 Hz, 2H), 7.34-7.32 (m, 2H), 7.15-7.12 (m, 1H), 4.02 (s, 3H) ppm; ^{13}C NMR (100 MHz, $CDCl_3$): δ 142.2, 141.5, 135.5, 134.8, 130.1, 127.8, 127.3, 126.5, 125.4, 121.5, 121.3, 121.0, 109.4, 35.6 ppm. HRMS (ESI) m/z calcd for $C_{14}H_{12}BrN_2$ $[M+H]^+$: 287.0179, found: 297.0177.

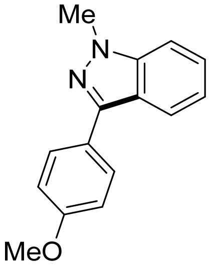

2h

Following general procedure, the crude product was purified by flash column chromatography (10:1 petroleum ether: ethyl acetate) to afford **2h** (35.9 mg, 76% yield) as slight yellow oil. Reaction time: 24h. ^{1}H NMR (400 MHz, $CDCl_3$): δ = 7.86 (d, *J* = 8.0 Hz, 1H), 7.78 (d, *J* = 8.0 Hz, 2H), 7.29-7.27 (m, 2H), 7.09-7.06 (m, 1H), 6.93 (d, *J* = 8.8 Hz, 2H), 3.98 (s, 3H), 3.75 (s, 3H) ppm; ^{13}C NMR (100 MHz, $CDCl_3$): δ 159.5, 143.6, 141.4, 128.6, 126.4, 126.2, 121.5, 121.4, 120.7, 114.3, 109.2, 55.4, 35.5 ppm. HRMS (ESI) m/z calcd for $C_{15}H_{15}N_2O$ $[M+H]^+$: 239.1179, found: 239.1181.

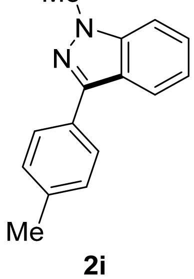

2i

Following general procedure, the crude product was purified by flash column chromatography (20:1 petroleum ether: ethyl acetate) to afford **2i** (32.5 mg, 80% yield) as white solid. m.p. 61-62 °C. Reaction time: 24h. ^{1}H NMR (400 MHz, $CDCl_3$): δ = 7.99 (d, J = 8.4 Hz, 1H), 7.84 (d, J = 8.0 Hz, 2H), 7.42-7.37 (m, 2H), 7.29 (d, J = 8.0 Hz, 2H), 7.20-7.16 (m, J = 8.8 Hz, 1H), 4.09 (s, 3H), 2.41 (s, 3H) ppm; ^{13}C NMR (100 MHz, $CDCl_3$): δ 143.8, 141.4, 137.6, 130.9, 129.5, 127.3, 126.2, 121.6, 121.5, 120.8, 109.2, 35.5, 21.4 ppm. HRMS (ESI) m/z calcd for $C_{15}H_{15}N_2$ $[M+H]^+$: 223.1230, found: 223.1232.

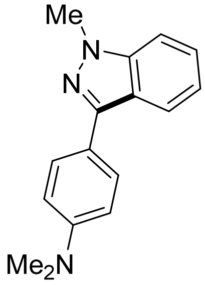

2j

Following general procedure, the crude product was purified by flash column chromatography (5:1 petroleum ether: ethyl acetate) to afford **2j** (42.0 mg, 67% yield) as slight yellow solid. m.p. 80-81 °C. Reaction time: 24h. ^{1}H NMR (400 MHz, $CDCl_3$): δ = 7.92 (d, J = 8.0 Hz, 1H), 7.76 (d, J = 8.4 Hz, 2H), 7.31-7.28 (m, 2H), 7.10-7.07 (m, J = 8.8 Hz, 1H), 6.77 (d, J = 8.8 Hz, 2H), 4.00 (s, 3H), 2.93 (s, 6H) ppm; ^{13}C NMR (100 MHz, $CDCl_3$): δ 150.3, 144.3, 141.4, 128.2, 126.1, 122.0, 121.7, 121.6, 120.3, 112.7, 109.0, 40.6, 35.4 ppm. HRMS (ESI) m/z calcd for $C_{16}H_{18}N_3$ $[M+H]^+$: 252.1495, found: 252.1498.

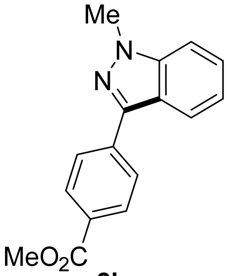

2k

Following general procedure, the crude product was purified by flash column chromatography (20:1 petroleum ether: ethyl acetate) to afford **2k** (31.1 mg, 59% yield) as slight yellow solid. m.p. 125-126 °C. Reaction time: 24h. ^{1}H NMR (400 MHz, $CDCl_3$): δ = 8.16 (d, J = 6.8 Hz, 2H), 8.05 (d, J = 7.2 Hz, 2H), 8.01 (d, J = 8.0 Hz, 1H), 7.43-7.42 (m, 2H), 7.26-7.22 (m, 1H), 4.13 (s, 3H), 3.94 (s, 3H) ppm; ^{13}C NMR (100 MHz, $CDCl_3$): δ 167.0, 142.4, 141.5, 138.2, 130.1, 129.1, 127.0, 126.5, 121.7, 121.5, 121.1, 109.5, 52.2, 35.7 ppm. HRMS (ESI) m/z calcd for $C_{16}H_{15}N_2O_2$ $[M+H]^+$: 267.1128, found: 267.1124.

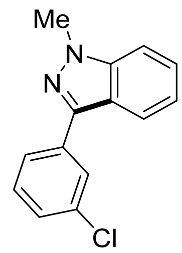

2l

Following general procedure, the crude product was purified by flash column chromatography (20:1 petroleum ether: ethyl acetate) to afford **2l** (40.7 mg, 84% yield) as colourless oil. Reaction time: 24h. ^{1}H NMR (400 MHz, $CDCl_3$): δ = 7.89-7.87 (m, 2H), 7.75 (dt, J = 1.6, 7.6 Hz, 1H), 7.34-7.30 (m, 4H), 7.15-7.11 (m, 1H), 4.01(s, 3H) ppm; ^{13}C NMR (100 MHz, $CDCl_3$): δ 141.1, 140.4, 134.5, 133.7, 129.0, 126.7, 126.2, 125.4, 124.3, 120.4, 120.2, 120.0, 108.3, 34.6 ppm. HRMS (ESI) m/z calcd for $C_{14}H_{12}ClN_2$ $[M+H]^+$: 243.0684, found: 243.0683.

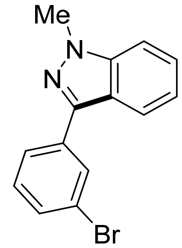

2m

Following general procedure, the crude product was purified by flash column chromatography (20:1 petroleum ether: ethyl acetate) to afford **2m** (45.4 mg, 80% yield) as white solid. m.p. 71-72 °C. Reaction time: 24h. ^{1}H NMR (400 MHz, $CDCl_3$): δ = 8.12 (t, J = 1.6 Hz, 1H), 7.97 (d, J = 8.0 Hz, 1H), 7.89 (dt, J = 1.2 Hz, 7.6 Hz, 1H), 7.52-7.49 (m, 1H), 7.43-7.42 (m, 2H), 7.35 (t, J = 8.0 Hz, 1H), 7.24-7.21 (m, 1H), 4.11 (s, 3H) ppm; ^{13}C NMR (100 MHz, $CDCl_3$): δ 142.1, 141.5, 135.8, 130.7, 130.3, 130.2, 126.5, 125.8, 123.0, 121.5, 121.3, 121.0, 109.4, 35.7 ppm. HRMS (ESI) m/z calcd for $C_{14}H_{12}BrN_2$ $[M+H]^+$: 287.0179, found: 287.0177.

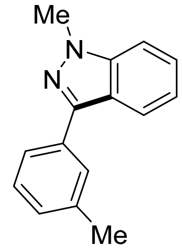

2n

Following general procedure, the crude product was purified by flash column chromatography (20:1 petroleum ether: ethyl acetate) to afford **2n** (27.5 mg, 63% yield) as slight yellow oil. Reaction time: 24h. ^{1}H NMR (400 MHz, $CDCl_3$): δ = 8.01 (d, J = 8.0 Hz, 1H), 7.78 (s, 1H), 7.75 (d, J = 7.2 Hz, 2H), 7.41-7.37 (m, 3H), 7.22-7.19 (m, 2H), 4.11 (s, 3H), 2.45 (s, 3H) ppm; ^{13}C NMR (100 MHz, $CDCl_3$): δ

143.9, 141.5, 138.5, 128.7(2C), 128.0, 126.3, 124.6, 121.7, 121.5, 120.9, 109.2, 35.5, 21.6 ppm. HRMS (ESI) m/z calcd for $C_{15}H_{15}N_2$ $[M+H]^+$: 223.1230, found: 223.1230.

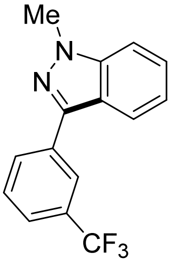

2o

Following general procedure, the crude product was purified by flash column chromatography (20:1 petroleum ether: ethyl acetate) to afford **2o** (36.0 mg, 65% yield) as slight yellow oil. Reaction time: 24h. ^{1}H NMR (400 MHz, $CDCl_3$): δ = 8.24 (s, 1H), 8.15-8.13 (m, 1H), 7.99-7.97 (m, 1H), 7.64-7.58 (m, 2H), 7.45-7.43 (m, 2H), 7.27-7.23 (m, 1H), 4.13 (s, 3H); ^{13}C NMR (100 MHz, $CDCl_3$): δ 142.1, 141.5, 134.6, 131.3 (q, J = 31.0 Hz), 130.4, 129.3, 126.5, 124.3 (q, J = 3.4 Hz), 124.2 (q, J = 270.9 Hz), 124.0 (q, J = 3.7 Hz), 121.5, 121.4, 120.9, 109.5, 35.7 ppm. HRMS (ESI) m/z calcd for $C_{15}H_{12}F_3N_2$ $[M+H]^+$: 277.0947, found: 277.0945.

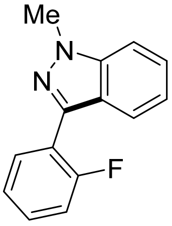

2p

Following general procedure, the crude product was purified by flash column chromatography (10:1 petroleum ether: ethyl acetate) to afford **2p** (15.0 mg, 26% yield) as slight yellow solid. m.p. 70-71 °C. Reaction time: 24h. ^{1}H NMR (400 MHz, $CDCl_3$): δ = 7.78-7.75 (m, 1H), 7.72 (dt, J = 1.6, 7.2 Hz, 1H), 7.36-7.34 (m, 2H), 7.33-7.31 (m, 1H), 7.21-7.11 (m, 3H), 4.07 (s, 3H) ppm; ^{13}C NMR (100 MHz, $CDCl_3$): δ 159.0 (d, J = 247.4 Hz), 140.0, 138.4, 130.0 (d, J = 3.4 Hz), 128.7 (d, J = 8.1 Hz), 125.4, 123.4 (d, J = 4.0 Hz), 121.6, 120.8 (d, J = 7.5 Hz), 120.2 (d, J = 4.6 Hz), 119.8, 115.1 (d, J = 22.3 Hz), 108.0, 34.6 ppm. HRMS (ESI) m/z calcd for $C_{14}H_{12}FN_2$ $[M+H]^+$: 227.0979, found: 227.0979.

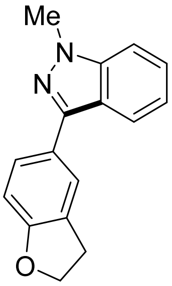

2q

Following general procedure, the crude product was purified by flash column

chromatography (20:1 petroleum ether: ethyl acetate) to afford **2q** (22.0 mg, 44% yield) as slight yellow solid. m.p. 71-72 °C. Reaction time: 24h. ^{1}H NMR (400 MHz, $CDCl_3$): δ = 7.96 (d, J = 8.0 Hz, 1H), 7.78 (s, 1H), 7.71-7.9 (m, 1H), 7.40-7.39 (m, 2H), 7.20-7.18 (m, 1H), 6.91 (d, J = 8.0 Hz, 1H), 4.62 (t, J = 8.8 Hz, 2H), 4.01(s, 3H), 3.29 (t, J = 8.8 Hz, 2H) ppm; ^{13}C NMR (100 MHz, $CDCl_3$): δ 160.1, 144.1, 141.4, 127.8, 127.5, 126.2, 124.1, 121.5, 121.4, 120.6, 109.5, 109.1, 71.5, 35.5, 29.7 ppm. HRMS (ESI) m/z calcd for $C_{16}H_{15}N_2O$ $[M+H]^+$: 251.1179, found: 251.1179.

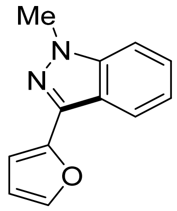

2r

Following general procedure, the crude product was purified by flash column chromatography (15:1 petroleum ether: ethyl acetate) to afford **2r** (36.0 mg, 91% yield) as slight yellow oil. Reaction time: 24h. ^{1}H NMR (400 MHz, $CDCl_3$): δ = 8.06 (dt, J = 1.2, 8.4 Hz, 1H), 7.57 (dd, J = 0.8, 2.0 Hz, 1H), 7.44-7.36 (m, 2H), 7.24-7.20 (m, 1H), 6.89 (dd, J = 0.8, 3.2 Hz, 1H), 6.55 (dd, J = 2.0, 3.2 Hz, 1H), 4.09 (s, 3H) ppm; ^{13}C NMR (100 MHz, $CDCl_3$): δ 148.9, 142.1, 140.9, 136.0, 126.7, 121.6, 121.2, 120.9, 111.4, 109.1, 106.6, 35.7 ppm. HRMS (ESI) m/z calcd for $C_{12}H_{11}N_2O$ $[M+H]^+$: 199.0866, found: 199.0867.

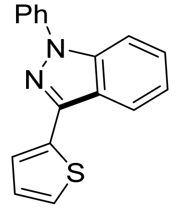

2s

Following general procedure, the crude product was purified by flash column chromatography (20:1 petroleum ether: ethyl acetate) to afford **2s** (24.8 mg, 45% yield) as slight yellow solid. m.p. 88-89 °C. Reaction time: 24h. ^{1}H NMR (400 MHz, $CDCl_3$): δ = 8.09 (d, J = 8.0 Hz, 1H), 7.78-7.72 (m, 4H), 7.53 (t, J = 8.0 Hz, 2H), 7.44 (t, J = 7.6 Hz, 1H), 7.40-7.36 (m, 2H), 7.29 (t, J = 7.2 Hz, 1H), 7.20-7.18 (m, 1H) ppm; ^{13}C NMR (100 MHz, $CDCl_3$): δ 141.1, 140.2, 140.0, 135.5, 129.5, 127.7, 127.4, 126.8, 125.5, 125.2, 123.1, 122.6, 122.2, 121.4, 110.8 ppm. HRMS (ESI) m/z calcd for $C_{17}H_{12}N_2S$ $[M+H]^+$: 277.0794, found: 277.0795.

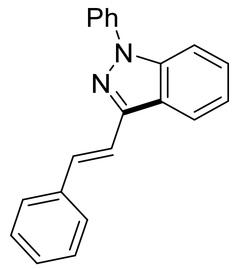

2t

Following general procedure, the crude product was purified by flash column chromatography (20:1 petroleum ether: ethyl acetate) to afford **2t** (47.5 mg, 80% yield) as slight yellow oil. Reaction time: 24h. ^{1}H NMR (400 MHz, $CDCl_3$): δ = 8.06 (d, J = 8.0 Hz, 1H), 7.76-7.72 (m, 3H), 7.63-7.59 (m, 3H), 7.54-7.51 (m, 3H), 7.45-7.35 (m, 4H), 7.30-7.26 (m, 2H) ppm; ^{13}C NMR (100 MHz, $CDCl_3$): 144.3, 140.2, 140.0, 137.3, 131.7, 129.5, 128.8, 128.0, 127.3, 126.8, 126.7, 123.5, 123.0, 122.0, 121.2, 119.7, 110.8 δppm. HRMS (ESI) m/z calcd for $C_{21}H_{17}N_2$ $[M+H]^+$: 297.1386, found: 297.1388.

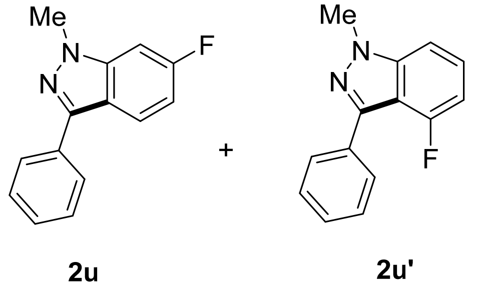

2u **2u'**

Following general procedure, the crude product was purified by flash column chromatography (20:1 petroleum ether: ethyl acetate) to afford **2u** and **2u'** (**2u**: **2u**'=5:1, 40.0 mg, 89% yield) as slight yellow oil. Reaction time: 24h. Mixed spectrum: ^{1}H NMR (400 MHz, $CDCl_3$): δ = 7.94-7.91 (m, 2.22H), 7.48-7.45 (m, 2H), 7.40-7.36 (m, 1H), 7.33-7.28 (m, 0.89H), 7.13 (d, J = 8.8 Hz, 0.85H), 7.00 (dd, J = 2.4, 8.8 Hz, 0.20H), 6.95 (dd, J = 2.0, 8.8 Hz, 0.21H), 6.83-6.78 (m, 0.84H), 4.07(s, 2.53H), 4.03 (s, 0.54H) ppm; ^{13}C NMR (100 MHz, $CDCl_3$): δ 162.2 (d, J = 243.6 Hz), 156.2 (d, J = 251.5 Hz), 144.2 (d, J = 8.9 Hz), 144.1, 142.9 (d, J = 4.0 Hz), 141.8 (d, J = 12.1 Hz), 133.2, 133.0, 128.9, 128.6 (d, J = 4.9 Hz), 128.4, 128.1, 128.0, 127.4 (d, J = 2.7 Hz), 127.3, 122.8 (d, J = 10.9 Hz), 118.6, 111.4 (d, J = 20.5 Hz), 110.8(d, J = 26.9 Hz), 105.5 (d, J = 20.6 Hz), 105.2 (d, J = 3.9 Hz), 94.8 (d, J = 26.4 Hz), 35.9, 35.6 ppm. HRMS (ESI) m/z calcd for $C_{15}H_{12}FN_2$ $[M+H]^+$: 227.0979, found: 227.0980.

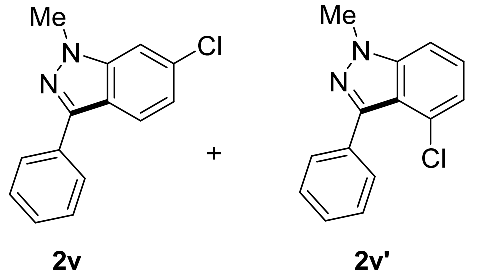

2v **2v'**

Following general procedure, the crude product was purified by flash column chromatography (20:1 petroleum ether: ethyl acetate) to afford **2v** and **2v'** (**2v**: **2v**'=3:1, 30.0 mg, 68% yield) as slight yellow oil. Reaction time: 24h. Mixed spectrum: ^{1}H NMR (400 MHz, $CDCl_3$): δ = 8.02-8.00 (m, 0.25H), 7.97-7.94 (m, 0.48H), 7.91-7.90 (m, 0.73H), 7.89-7.87 (m, 1.48H), 7.51-7.47 (m, 2H), 7.41-7.37 (m, 2H), 7.22-7.18 (m, 0.23H), 7.14 (dd, J = 2.0, 8.4 Hz, 0.71H), 4.11(s, 0.71H), 4.05 (s, 2.23H) ppm; ^{13}C NMR (100 MHz, $CDCl_3$): δ 144.0, 143.7, 141.9, 141.5, 133.7, 133.1, 132.8, 128.9, 128.8, 128.2, 127.8, 127.4, 127.3, 126.3, 122.4, 122.0, 121.6, 121.4, 120.9, 120.2, 109.2, 109.0, 35.7, 35.6 ppm. HRMS (ESI) m/z calcd for $C_{14}H_{12}ClN_2$ $[M+H]^+$: 243.0684, found: 243.0684.

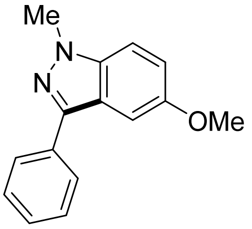

2w

Following general procedure, the crude product was purified by flash column chromatography (15:1 petroleum ether: ethyl acetate) to afford **2w** (35.7 mg, 75% yield) as slight yellow solid. m.p. 62-63 °C. Reaction time: 24h. ^{1}H NMR (400 MHz, $CDCl_3$): δ = 7.92-7.90 (m, 2H), 7.51-7.48 (m, 2H), 7.39-7.35 (m, 1H), 7.32-7.28 (m, 2H), 7.09 (dd, J = 2.0, 8.8 Hz, 1H), 4.07 (s, 3H), 3.86 (s, 3H) ppm; ^{13}C NMR (100 MHz, $CDCl_3$): δ 155.1, 142.9, 137.5, 134.0, 128.9, 127.6, 127.2, 121.7, 118.5, 110.3, 100.5, 55.9, 35.7 ppm. HRMS (ESI) m/z calcd for $C_{15}H_{15}N_2O$ $[M+H]^+$: 239.1179, found: 239.1181.

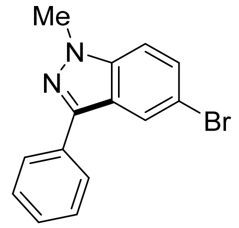

2x

Following general procedure, the crude product was purified by flash column chromatography (20:1 petroleum ether: ethyl acetate) to afford **2x** (40.0 mg, 70% yield) as white solid. m.p. 106-107 °C. Reaction time: 24h. ^{1}H NMR (400 MHz, $CDCl_3$): δ = 8.12 (s, 1H), 7.88 (d, J = 7.2 Hz, 2H), 7.51-7.45 (m, 3H), 7.40-7.39 (m, 1H), 7.25 (d, J = 8.8 Hz, 1H), 4.07 (s, 3H) ppm; ^{13}C NMR (100 MHz, $CDCl_3$): δ 143.2, 140.1, 133.0, 129.4, 128.9, 128.2, 127.3, 123.8, 123.1, 114.2, 110.7, 35.8 ppm. HRMS (ESI) m/z calcd for $C_{14}H_{12}BrN_2$ $[M+H]^+$: 287.0179, found: 287.0179.

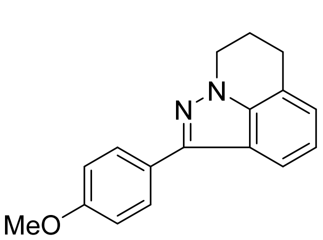

4a

Following general procedure, the crude product was purified by flash column chromatography (15:1 petroleum ether: ethyl acetate) to afford **4a** (24.4 mg, 46% yield) as white solid. m.p. 113-114 °C. Reaction time: 24h. ^{1}H NMR (400 MHz, $CDCl_3$): *δ* = 7.94 (d, *J* = 8.8 Hz, 2H), 7.78 (d, *J* = 7.2 Hz, 1H), 7.12-7.07 (m, 2H), 7.02 (d, *J* = 8.8 Hz, 2H), 4.44 (t, *J* = 6.4 Hz, 2H), 3.03 (t, *J* = 6.4 Hz, 2H), 2.37-2.34 (m, 2H) ppm; ^{13}C NMR (100 MHz, $CDCl_3$): δ 159.3, 143.0, 139.6, 128.1, 127.0, 122.2, 122.0, 121.5, 118.9, 118.8, 114.3, 55.4, 46.3, 24.3, 23.1 ppm. HRMS (ESI) m/z calcd for $C_{17}H_{17}N_2O$ $[M+H]^+$: 265.1336 found: 265.1336.

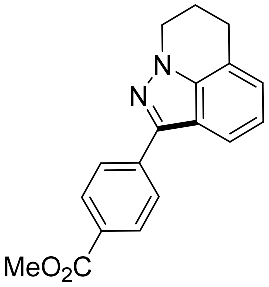

4b

Following general procedure, the crude product was purified by flash column chromatography (15:1 petroleum ether: ethyl acetate) to afford **4b** (26.0 mg, 45% yield) as yellow solid. m.p. 156-157 °C. Reaction time: 24h. ^{1}H NMR (400 MHz, $CDCl_3$): *δ* = 8.16-8.09 (m, 4H), 7.84-7.82 (m, 1H), 7.18-7.14 (m, 1H), 7.12-7.10 (m, 1H), 4.47 (t, *J* = 6.0 Hz, 2H), 3.94 (s, 3H), 3.05 (t, *J* = 6.4 Hz, 2H), 2.37-2.34 (m, 2H) ppm; ^{13}C NMR (100 MHz, $CDCl_3$): δ 167.0, 141.9, 139.7, 138.8, 130.1, 128.8, 126.5, 122.5, 122.4, 122.3, 119.3, 118.6, 52.1, 46.6, 24.2, 23.0 ppm. HRMS (ESI) m/z calcd for $C_{14}H_{13}N_2$ $[M+H]^+$: 293.1285, found: 293.1286.

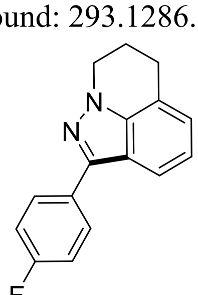

4c

Following general procedure, the crude product was purified by flash column chromatography (20:1 petroleum ether: ethyl acetate) to afford **4c** (30.1 mg, 60% yield) as slight yellow oil. Reaction time: 24h. 1H NMR (400 MHz, CDCl3): δ = 7.90-7.87 (m, 2H), 7.66 (d, J = 8.0 Hz, 1H), 7.10-7.06 (m, 2H), 7.03-6.99 (m, 2H), 4.36 (t, *J* = 6.0 Hz, 2H), 2.94 (t, *J* = 6.4 Hz, 2H), 2.28-2.24 (m, 2H) ppm; ^{13}C NMR (100 MHz, $CDCl_3$): δ 162.5 (d, *J* = 246.3 Hz), 142.2, 139.6, 130.4 (d, *J* = 3.8 Hz),

128.5 (d, J = 7.8 Hz), 122.2, 122.1 (d, J = 43.4 Hz), 118.9, 118.5, 115.9, 115.6, 46.4, 24.2, 23.0 ppm. HRMS (ESI) m/z calcd for $C_{14}H_{13}N_2$ $[M+H]^+$: 253.1136, found: 253.1137.

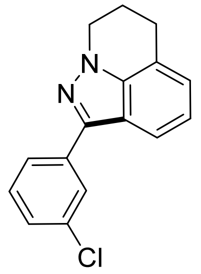

4d

Following general procedure, the crude product was purified by flash column chromatography (20:1 petroleum ether: ethyl acetate) to afford **4d** (36.6 mg, 68% yield) as slight yellow solid. m.p. 96-97 °C. Reaction time: 24h. ^{1}H NMR (400 MHz, $CDCl_3$): δ = 8.01 (t, J = 1.6 Hz, 1H), 7.90-7.88 (m, 1H), 7.77 (d, J = 8.0 Hz, 1H), 7.39 (t, J = 7.6 Hz, 1H), 7.32-7.30 (m, 1H), 7.15-7.07 (m, 2H), 4.44 (t, J = 6.0 Hz, 2H), 3.02 (t, J = 6.4 Hz, 2H), 2.37-2.31 (m, 2H) ppm; ^{13}C NMR (100 MHz, $CDCl_3$): δ 141.6, 139.6, 136.1, 134.7, 130.0, 127.5, 126.8, 124.9, 122.5, 122.3, 122.2, 119.0, 118.4, 46.5, 24.2, 23.0 ppm. HRMS (ESI) m/z calcd for $C_{14}H_{13}N_2$ $[M+H]^+$: 269.0840, found: 269.0840.

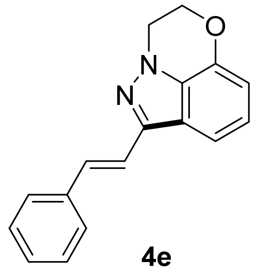

4e

Following general procedure, the crude product was purified by flash column chromatography (8:1 petroleum ether: ethyl acetate) to afford **4e** (43.0 mg, 80% yield) as white solid. m.p. 82-83 °C. Reaction time: 24h. ^{1}H NMR (400 MHz, $CDCl_3$): δ = 7.56 (d, J = 7.6 Hz, 2H), 7.53 (d, J = 8.8 Hz, 1H), 7.44 (s, 2H), 7.37 (t, J = 7.2 Hz, 2H), 7.27 (t, J = 7.6 Hz, 1H), 7.08 (t, J = 8.0 Hz, 1H), 6.77 (d, J = 7.2 Hz, 1H), 4.58 (t, J = 4.4 Hz, 2H), 4.56 (t, J = 4.8 Hz, 2H); ^{13}C NMR (100 MHz, $CDCl_3$): δ 143.8, 142.8, 137.3, 131.9, 131.1, 128.8, 127.9, 126.5, 123.0, 121.3, 120.8, 113.8, 108.3, 66.3, 46.1 ppm. HRMS (ESI) m/z calcd for $C_{14}H_{13}N_2$ $[M+H]^+$: 263.1179, found: 263.1180.

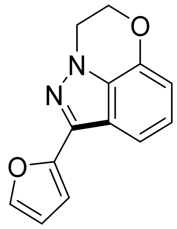

4f

Following general procedure, the crude product was purified by flash column chromatography (8:1 petroleum ether: ethyl acetate) to afford **4f** (35.0 mg, 77% yield)

as white solid. m.p. 129-130 °C. Reaction time: 24h. ^{1}H NMR (400 MHz, $CDCl_3$): δ = 7.56 (d, J = 1.6 Hz, 1H), 7.53 (d, J = 8.4 Hz, 1H), 7.08 (t, J = 8.0 Hz, 1H), 6.88 (d, J = 3.6 Hz, 1H), 6.77 (d, J = 7.2 Hz, 1H), 6.55 (m, 1H), 4.62 (t, J = 4.4 Hz, 2H), 4.51 (t, J = 4.8 Hz, 2H); ^{13}C NMR (100 MHz, $CDCl_3$): δ 148.9, 142.7, 142.3, 137.0, 131.6, 123.1, 120.3, 113.8, 111.5, 108.3, 106.9, 66.3, 46.2 ppm. HRMS (ESI) m/z calcd for $C_{14}H_{13}N_2$ $[M+H]^+$: 227.0815, found: 227.0814.

4. X-ray structure of 4f (CCDC 1499990)

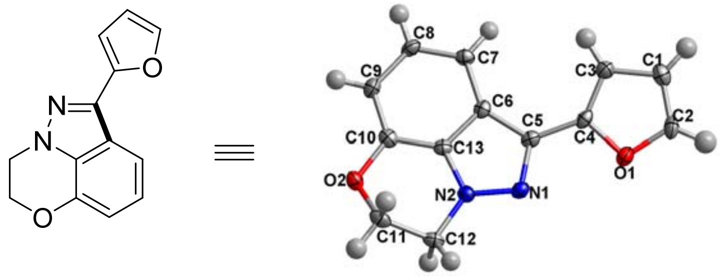

4f

Bond lengths (Å) and angles (deg):

O1	-C2	1.3753	C7	-C8	1.3777
O1	-C4	1.3807	C8	-C9	1.4275
O2	-C10	1.3823	C9	-C10	1.3652
O2	-C11	1.4602	C10	-C13	1.3969
N1	-N2	1.3592	C11	-C12	1.5252
N1	-C5	1.3465	C1	-H1	0.9300
N2	-C12	1.4525	C2	-H2	0.9300
N2	-C13	1.3495	C3	-H3	0.9300
C1	-C2	1.3377	C7	-H7	0.9300
C1	-C3	1.4250	C8	-H8	0.9300
C3	-C4	1.3507	C9	-H9	0.9300
C4	-C5	1.4485	C11	-H11A	0.9700
C5	-C6	1.4431	C11	-H11B	0.9700

C6	-C7	1.4152	C12	-H12A	0.9700
C6	-C13	1.3946	C12	-H12B	0.9700

C2	-O1	-C4	105.44	N2	-C13	-C6	109.23
C10	-O2	-C11	111.87	N2	-C13	-C10	125.14
N2	-N1	-C5	105.44	C6	-C13	-C10	125.60
N1	-N2	-C12	128.68	C2	-C1	-H1	127.00
N1	-N2	-C13	111.45	C3	-C1	-H1	127.00
C12	-N2	-C13	119.87	O1	-C2	-H2	124.00
C2	-C1	-C3	106.09	C1	-C2	-H2	124.00
O1	-C2	-C1	111.42	C1	-C3	-H3	126.00
C1	-C3	-C4	107.10	C4	-C3	-H3	126.00
O1	-C4	-C3	109.94	C6	-C7	-H7	121.00
O1	-C4	-C5	117.10	C8	-C7	-H7	121.00
C3	-C4	-C5	132.93	C7	-C8	-H8	118.00
N1	-C5	-C4	121.52	C9	-C8	-H8	118.00
N1	-C5	-C6	111.49	C8	-C9	-H9	120.00
C4	-C5	-C6	126.94	C10	-C9	-H9	120.00
C5	-C6	-C7	140.47	O2	-C11	-H11A	109.00
C5	-C6	-C13	102.38	O2	-C11	-H11B	109.00
C7	-C6	-C13	117.14	C12	-C11	-H11A	109.00
C6	-C7	-C8	117.89	C12	-C11	-H11B	109.00
C7	-C8	-C9	123.14	H11A	-C11	-H11B	108.00

C8	-C9	-C10	119.64	N2	-C12	-H12A	110.00
O2	-C10	-C9	125.70	N2	-C12	-H12B	110.00
O2	-C10	-C13	117.72	C11	-C12	-H12A	110.00
C9	-C10	-C13	116.57	C11	-C12	-H12B	110.00
O2	-C11	-C12	114.25	H12A	-C12	-H12B	109.00
N2	-C12	-C11	107.01				

5. Optimization of the Reaction Conditions

1. Initial optimization of the Ag salts：

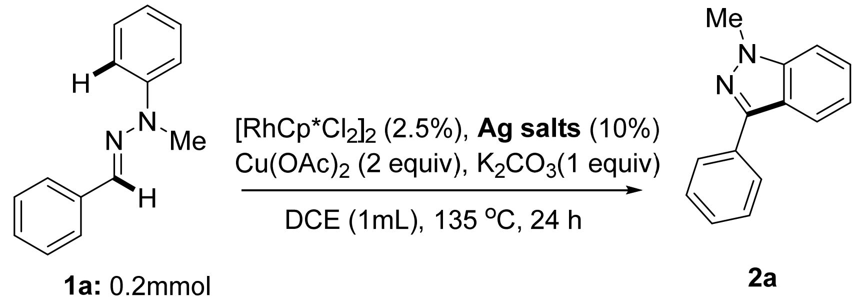

Entry	Ag salts	Yield (%)[a]
**1**	**AgOTf**	**75**
2	$AgSbF_6$	59
3	$AgBF_4$	50
4	AgOAc	10
5	-	50

[a] Yields determined by ^{1}NMR spectroscopy using *N*-(4-methoxyphenyl)acetamide as the internal standard.

2. Initial optimization of the solvents：

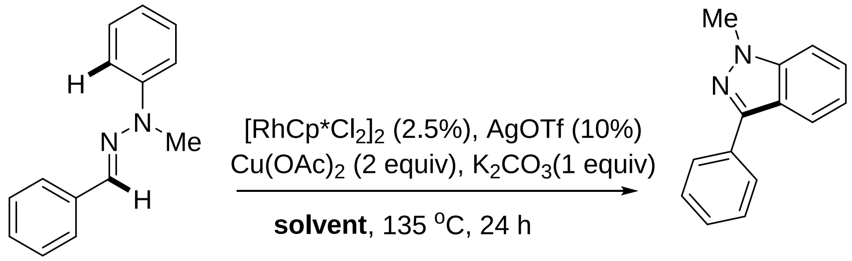

1a: 0.2 mmol **2a**

Entry	Solvent	Yield(%)[a]
1	DCE (1mL)	75
2	THF (1ml)	11
3	1,4-Dioxane (1ml)	22
**4**	**DCE (0.5 mL)**	**80**

[a] Yields determined by ^{1}NMR spectroscopy using *N*-(4-methoxyphenyl)acetamide as the internal standard.

6. Deuterium labeling experiment

1. Synthesis of deuterated *N*-nitrosoaniline substrate **1j**-d_3:

1j-d_3 was prepared from *p*-tolualdehyde and deuterated hydrazine[1c] according to the general procedure **A.**

2. Deuterium labeling experiment

An oven-dried Schlenk tube (10 mL) was equipped with a magnetic stir bar, **1j**-d_3 (0.2 mmol, 45.4 mg), $[RhCp^*Cl_2]_2$ (0.025 equiv, 0.005 mmol, 3.1 mg), AgOTf (0.10 equiv, 0.02 mmol, 5.1 mg), $Cu(OAc)_2$ (2.0 equiv, 0.40 mmol, 72 mg), K_2CO_3 (1.0 equiv, 0.20 mmol, 27 mg). The flask was evacuated and backfilled with Argon for 3 times. 0.5 mL DCE was added with syringe under Argon. The resulting solution was stirred at 120°C for 24 h. Then the mixture was concentrated under vacuum to remove DCE, and the residue was subjected to column chromatography on silica gel to afford the product and remaining substrate.

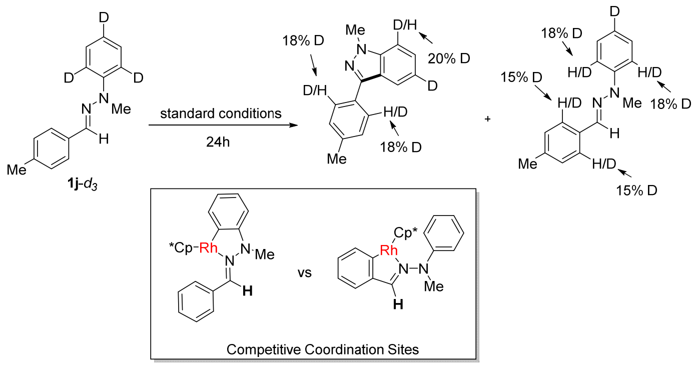

^{1}H NMR (400M,CH_3CN)

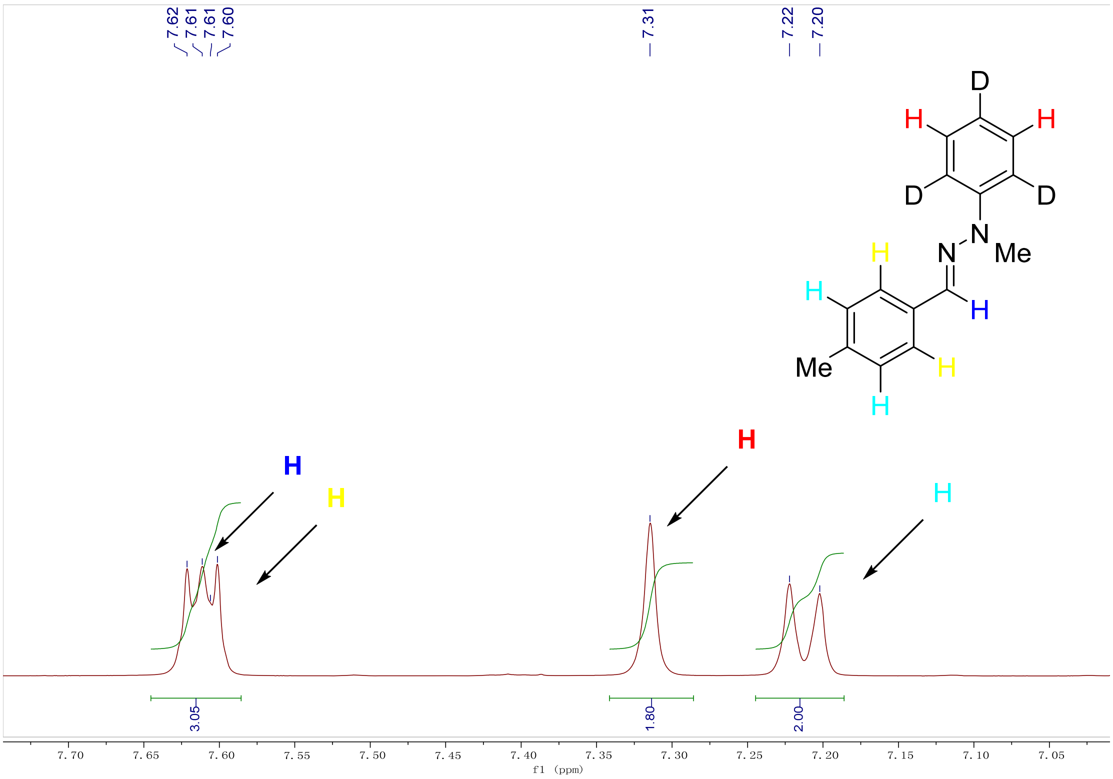

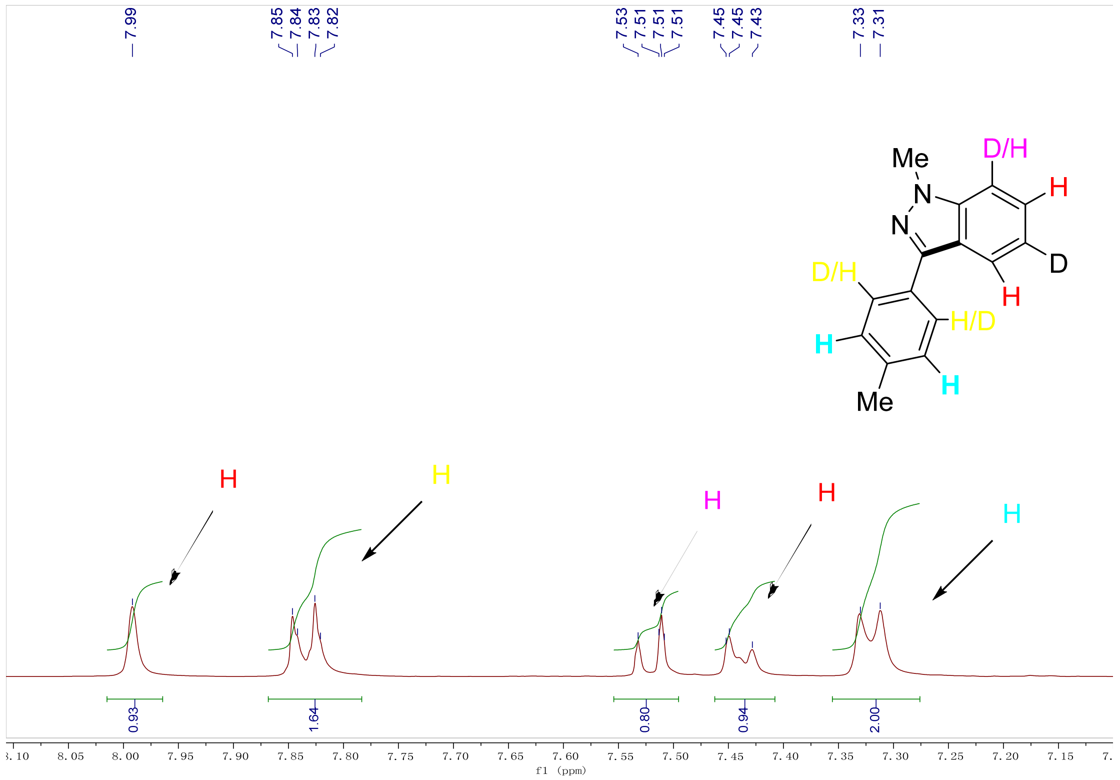
7.99
7.85
7.84
7.83
7.82
7.53
7.51
7.51
7.51
7.45
7.45
7.43
7.33
7.31
Me
N
N
D/H
H
D
H
D/H
H/D
H
H
Me
H
H
H
H
H
0.93
1.64
0.80
0.94
2.00
f1 (ppm)

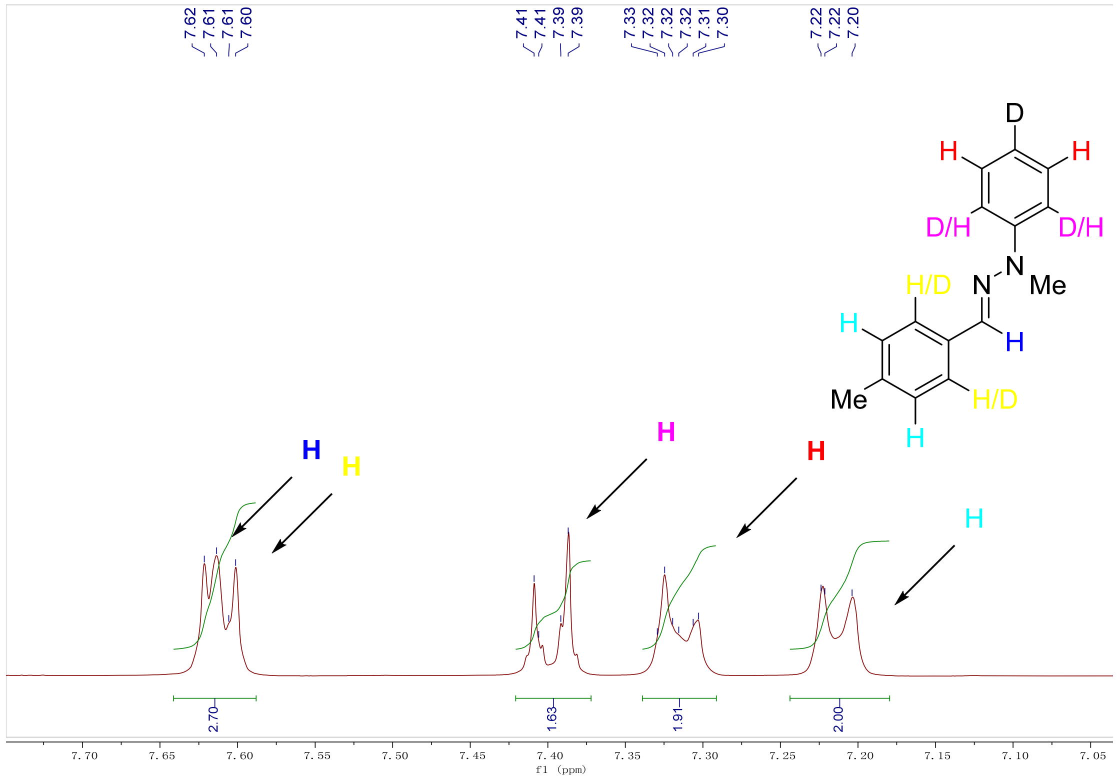
7.62
7.61
7.61
7.60
7.41
7.41
7.39
7.39
7.33
7.32
7.32
7.32
7.31
7.30
7.22
7.22
7.20
D
H
H
D/H
D/H
N
N
Me
H/D
H
H
Me
H/D
H
H
H
H
H
H
2.70
1.63
1.91
2.00
f1 (ppm)

7. Computational Details of Mechanistic Studies

Computational Details

All calculations were performed with the Gaussian 09 package.[3] All of the geometries were optimized by the M06-2X functional[4] with the basis set BS1. In BS1, for Rh atom, the effective core potential (ECP)[5] was employed for Rh, and the basis set for Rh is a modified LANL2DZ plus a set of f-type functions,[6] in which the two 5p and 6p functions of the standard LANL2DZ are replaced by the optimized 5p and 6p functions from Couty and Hall,[7] respectively. For other atoms, the 6-31G(d,p) basis set was used. To get more accurate energies, we performed single-point energy calculations for all the species at the M062x/BS2 level. In BS2, we employed the same basis set for Rh atom as in BS1, and the cc-pVTZ basis set for other atoms. The calculated Gibbs free energies refer to 298.15 K and 1 atm. For each transition state, the intrinsic reaction coordinate (IRC)[8] analysis was performed to verify whether the transition state truly connects the reactant and the product. The solvent effect was treated with the polarizable continuum model (PCM).[9] In calculating the free energies for species in the 1,2-dichloroethane solvent, we have used the method developed by Whitesides *et al.*[10] to calculate the entropic contributions. This method was designed to better describe the suppression of the translational entropy upon moving from gas phase to a solvent for each species.[11]

Activation free energy barriers here are defined as the free energy difference between the transition state and the lowest-energy stationary point before it in the reaction pathways.

1. The alternative pathway for C=N double insertion (path 2)

Direct formation of intermediate **IV** from **II** via C=N insertion reaction is

---

[3] M. J. Frisch, G. W. Trucks, H. B. Schlegel, G. E. Scuseria, M. A. Robb, J. R. Cheeseman, G. Scalmani, V. Barone, B. Mennucci, G. A. Petersson, H. Nakatsuji, M. Caricato, X. Li, H. P. Hratchian, A. F. Izmaylov, J. Bloino, G. Zheng, J. L. Sonnenberg, M. Hada, M. Ehara, K. Toyota, R. Fukuda, J. Hasegawa, M. Ishida, T. Nakajima, Y. Honda, O. Kitao, H. Nakai, T. Vreven, J. A. Montgomery, Jr., J. E. Peralta, F. Ogliaro, M. Bearpark, J. J. Heyd, E. Brothers, K. N. Kudin, V. N. Staroverov, T. Keith, R. Kobayashi, J. Normand, K. Raghavachari, A. Rendell, J. C. Burant, S. S. Iyengar, J. Tomasi, M. Cossi, N. Rega, J. M. Millam, M. Klene, J. E. Knox, J. B. Cross, V. Bakken, C. Adamo, J. Jaramillo, R. Gomperts, R. E. Stratmann, O. Yazyev, A. J. Austin, R. Cammi, C. Pomelli, J. W. Ochterski, R. L. Martin, K. Morokuma, V. G. Zakrzewski, G. A. Voth, P. Salvador, J. J. Dannenberg, S. Dapprich, A. D. Daniels, O. Farkas, J. B. Foresman, J. V. Ortiz, J. Cioslowski, and D. J. Fox, Gaussian, Inc., Wallingford CT, 2013.

[4] a) Y. Zhao, N. E Schultz, D. G.Truhlar, *J. Chem. Theory. Comput.* **2006**, *2*, 364; b) Y. Zhao, D. G.Truhlar, *J. Chem. Phys.* **2006**, *125*, 194101; c) Y. Zhao, D. G.Truhlar, *J. Phys. Chem. A* **2006**, *110*, 13126.

[5] a) P. J. Hay and W. R. Wadt, *J. Chem. Phys.*, 1985, **82**, 299; b) W. R. Wadt and P. J. Hay, *J. Chem. Phys.*, 1985, **82**, 284.

[6] A. W. Ehlers, M. Böhme, S. Dapprich, A. Gobbi, A. Höllwarth, V. Jonas, K. F. Köhler, R. Stegman, A. Veldkamp and G. Frenking, *Chem. Phys. Lett.*, **1993**, *208*, 111.

[7] M. Couty and M. B. Hall, *J. Comput. Chem.*, **1996**, *17*, 1359.

[8] C. Gonzalez, H. B. Schlegel, *J. Chem. Phys.***1989**, *90*, 2154.

[9] J. Tomasi, M. Persico, *Chem. Rev.* **1994**, *94*, 2027.

[10] M. Mammen, E. I. Shakhnovich, J. M. Deutch, G. M. Whitesides, *J. Org. Chem.* **1998**, *63*, 3821.

[11] a) G.Jindal, R. B. Sunoj, *J. Am. Chem. Soc.* **2014**, *136*, 15998; b) G. Zeng, S. Li, *Inorg. Chem.* **2011**, *50*, 10572.

possible. We have tried our best to locate the transition state of C=N insertion **II** or its rotation isomer (**II**). However, all attempts failed. We performed a relaxed potential energy scan by fixing the C-C distance at a series of values to estimate the approximate barrier. As shown in Figure S1, the generation of the C=N bond insertion intermediate (**IV**) is endothermic by about 40 kcal mol^{-1} in electronic energy (the relative free energy is 48.8 kcal mol^{-1} above above the active catalyst **I** and reactant **1a**), which suggests that the path 2 could be excluded.

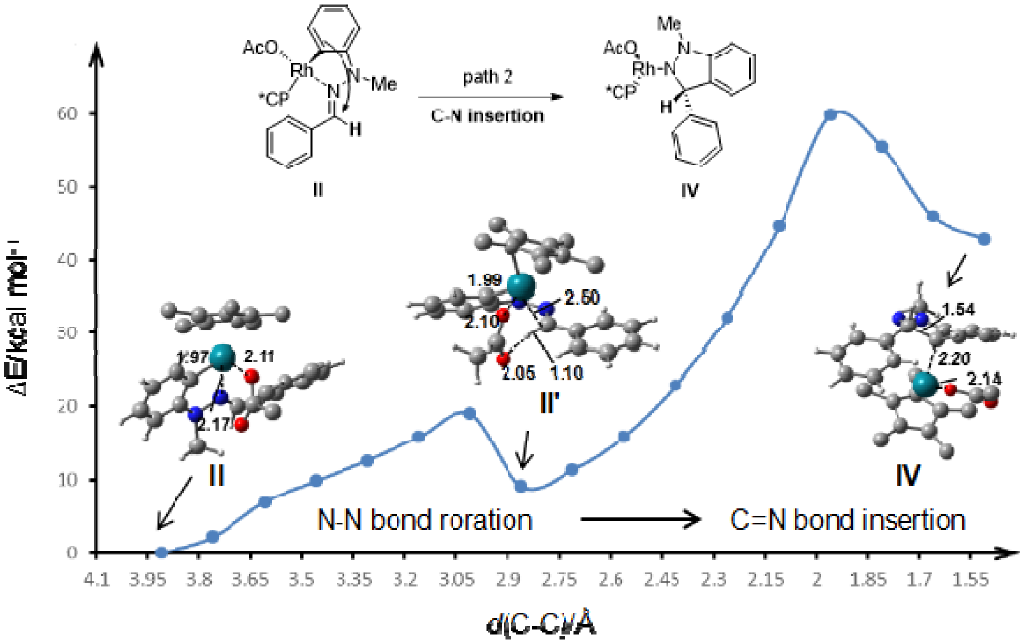

Figure S1. The relaxed potential energy scan for the C=N bond insertion step from **II** to **IV**.

2. Computational investigations of the steric effects on the proposed C-H/C-H cross coupling protocol

To investigate the influence of steric effects on this reaction, we calculated the corresponding Gibbs Free Energy Barriers of substrate **1A** and **1B**. The Second C-H bond activation step and reductive elimination step, are shown in Table S2. For substrates **5** and **6**, the corresponding reductive elimination barriers are 37.6 and 38.9 kcal mol^{-1}, respectively, which are much higher than that of substrate **1a**. These results can account for the experimental observations that no desired product was observed for substrate **1A** and **1B**.

Table S1 Comparison of the Gibbs Free Energy Barriers ($G^{\neq}$, in kcal/mol) of other substrates.

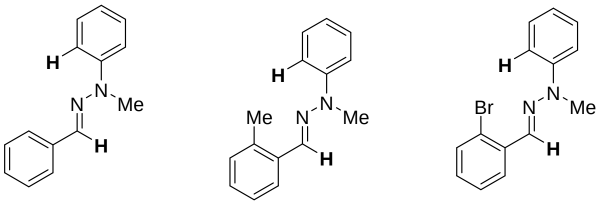

1a **1A** **1B**

Substrate	$G^{\neq}_{TS1}$	$G^{\neq}_{TS3}$	$G^{\neq}_{TS4}$	yield
**1a**	33.2	26.5	**32.3**	80%
**1A**	32.7	31.7	**37.6**	n.d.
**1B**	31.3	30.4	**38.9**	n.d.

Cartesian Coordinates and Energies of the Optimized Structures

Table S2. Calculated electronic energies in 1,2-dichloroethane (Esol, in a.u., at the M06-2X/BS2 level), thermal correction energies (Etherm, in a.u.), corrected translational entropy (St-W, in cal $mol^{-1}\cdot K^{-1}$), rotational entropy (Sr, in cal $mol^{-1}\cdot K^{-1}$), vibrational entropy (Sv, in cal $mol^{-1}\cdot K^{-1}$), corrected entropy contribution (TSW, in a.u.) and corrected free energies (G, in a.u.) for all stationary points in Figure 1 and Table S1. TSW stands for the corrected entropy using the Whitesides' method.

**Species**	E_{sol}	E_{therm}	$S_{t\text{-}W}$	S_r	S_v	TS_W	**G**
**1a**	-651.34439	0.26407	32.2	32.9	43.0	0.05135	-651.13167
**I**	-956.49032	0.35310	33.1	33.3	87.7	0.07323	-956.21045
**TS1**	-1607.80639	0.61481	34.8	36.3	134.6	0.09769	-1607.28926
**II**	-1378.74249	0.55066	34.4	35.7	120.0	0.09035	-1378.28219
**AcOH**	-229.09174	0.06650	28.5	23.8	2.5	0.02605	-229.05129
**TS2**	-1378.71211	0.54703	34.4	35.7	119.6	0.09013	-1378.25520

**II´**	-1378.72246	0.54971	34.4	35.8	122.6	0.09156	-1378.26430
**TS3**	-1378.70435	0.54454	34.4	35.7	116.9	0.08889	-1378.24869
**III**	-1149.61227	0.48007	33.9	35.3	98.1	0.07947	-1149.21166
**TS4**	-1149.58759	0.47795	33.9	34.9	96.3	0.07847	-1149.18811
**Cp*Rh(I)**	-499.40626	0.23626	31.5	30.8	43.7	0.05035	-499.22035
**2a**	-650.18142	0.24214	32.2	32.4	37.4	0.04841	-649.98769
**IV**	-1471.75614	0.62339	34.6	36.7	125.1	0.09334	-1471.22609
**1A**	-690.65386	0.29355	33.1	33.3	51.7	0.05613	-690.41645
**1B**	-3224.99111	0.25560	32.4	34.3	49.7	0.05526	-3224.79077
**1A-TS1**	-1647.11755	0.64437	35.2	36.5	142.1	0.10158	-1646.57475
**1B-TS1**	-4181.45374	0.60675	34.8	37.0	147.8	0.10433	-4180.95132
**1A-TS3**	-1418.00999	0.57443	34.5	35.9	118.1	0.08956	-1417.52512
**1B-TS3**	-3952.34713	0.53654	34.9	36.2	120.1	0.09083	-3951.90142
**1A-TS4**	-1188.89175	0.50802	34.0	35.0	100.6	0.08061	-1188.46433
**1B-TS4**	-3723.22387	0.46944	34.5	35.6	103.1	0.08226	-3722.83669

1a

Center Number	Atomic Number	Atomic Type	Coordinates (Angstroms) X	Y	Z
1	6	0	-1.390622	0.688688	-0.902686
2	6	0	-0.989431	-0.061972	-2.015928
3	6	0	-1.427473	0.282633	-3.286433
4	6	0	-2.271680	1.378475	-3.470009
5	6	0	-2.675731	2.127874	-2.369693
6	6	0	-2.237506	1.783909	-1.094614
7	1	0	-0.337163	-0.915316	-1.862283

8	1	0	-1.112513	-0.306602	-4.142064
9	1	0	-3.333649	2.980907	-2.502625
10	1	0	-2.553313	2.370610	-0.235395
11	6	0	-0.948945	0.353366	0.458016
12	7	0	-0.151263	-0.632999	0.652662
13	6	0	-0.100823	-0.156087	3.029386
14	1	0	0.094875	0.899590	2.806536
15	1	0	-1.161073	-0.265192	3.291334
16	7	0	0.261291	-0.961734	1.885377
17	1	0	0.514142	-0.440077	3.879810
18	1	0	-2.612176	1.643873	-4.465613
19	1	0	-1.345869	0.974526	1.263556
20	6	0	1.111296	-2.080051	1.986104
21	6	0	1.830886	-2.519463	0.866625
22	6	0	1.243817	-2.773216	3.194717
23	6	0	2.671953	-3.618188	0.969558
24	1	0	1.720618	-1.987075	-0.069260
25	6	0	2.100449	-3.866291	3.283982
26	1	0	0.658445	-2.487654	4.061475
27	6	0	2.821532	-4.297927	2.177073
28	1	0	3.226168	-3.940104	0.093246
29	1	0	2.187544	-4.390538	4.230544
30	1	0	3.485118	-5.152535	2.249974

I

Center Number	Atomic Number	Atomic Type	Coordinates (Angstroms) X	Y	Z
1	45	0	-0.142451	-0.077354	0.003466
2	6	0	-1.577094	1.368482	0.698290
3	6	0	-1.580895	1.328280	-0.749676
4	6	0	-1.951320	0.071790	1.172748
5	6	0	-1.965668	0.007090	-1.150036
6	6	0	-2.181983	-0.782674	0.033904
7	6	0	1.484327	-1.984410	0.001687
8	8	0	1.004647	-1.542599	-1.089258
9	8	0	0.998238	-1.555540	1.093998
10	6	0	2.618172	-2.959180	-0.001802
11	1	0	3.528241	-2.354360	0.000230
12	1	0	2.592146	-3.577406	0.895539
13	1	0	2.592162	-3.571182	-0.903441
14	6	0	-1.152501	2.562903	1.489892

15	1	0	-1.858933	3.386734	1.349341
16	1	0	-1.094561	2.336358	2.555214
17	1	0	-0.160406	2.879979	1.151958
18	6	0	-1.167074	2.479847	-1.608268
19	1	0	-1.882694	3.302943	-1.518431
20	1	0	-0.180379	2.827878	-1.286223
21	1	0	-1.101970	2.193111	-2.658598
22	6	0	-2.012831	-0.536259	-2.542505
23	1	0	-3.038951	-0.807335	-2.808603
24	1	0	-1.647452	0.192451	-3.266259
25	1	0	-1.383768	-1.427848	-2.615555
26	6	0	-2.588593	-2.221056	0.082505
27	1	0	-3.675234	-2.318492	0.177093
28	1	0	-2.275516	-2.741409	-0.824908
29	1	0	-2.122670	-2.723417	0.933291
30	6	0	-1.992869	-0.396875	2.591984
31	1	0	-1.618827	0.365956	3.275069
32	1	0	-3.019579	-0.646692	2.876658
33	1	0	-1.371655	-1.289267	2.709297
34	8	0	1.384894	1.352942	-0.001178
35	6	0	2.669544	1.134618	0.004100
36	8	0	3.225494	0.048352	0.017410
37	6	0	3.475714	2.428684	-0.006535
38	1	0	3.216958	3.016264	-0.890912
39	1	0	3.221586	3.026670	0.872284
40	1	0	4.541021	2.202295	-0.007525

TS1

Center Number	Atomic Number	Atomic Type	Coordinates (Angstroms) X	Y	Z
1	6	0	-0.800474	-1.035310	-2.231276
2	6	0	0.170071	-1.908806	-2.731923
3	6	0	0.260326	-2.113202	-4.106436
4	6	0	-0.619851	-1.474013	-4.976603
5	6	0	-1.614350	-0.634897	-4.473042
6	6	0	-1.711052	-0.423164	-3.102225
7	1	0	0.842765	-2.415516	-2.043629
8	1	0	1.019581	-2.786001	-4.492948
9	1	0	-2.314265	-0.152258	-5.147737
10	1	0	-2.485987	0.227329	-2.702554
11	6	0	-0.889730	-0.776582	-0.787415

12	7	0	0.164583	-0.569820	-0.082325
13	6	0	-1.219415	-0.558861	1.908098
14	1	0	-1.938919	0.226030	1.647957
15	1	0	-1.583353	-1.550807	1.623219
16	7	0	0.058389	-0.323495	1.254663
17	1	0	-1.061727	-0.558477	2.983037
18	1	0	-0.546293	-1.642608	-6.046567
19	1	0	-1.879679	-0.758339	-0.336665
20	6	0	1.264859	-0.419277	1.967997
21	6	0	2.385814	-0.992152	1.327412
22	6	0	1.365598	0.106380	3.261210
23	6	0	3.619123	-0.961904	2.000677
24	6	0	2.596487	0.088468	3.905203
25	1	0	0.509968	0.561091	3.749476
26	6	0	3.734880	-0.426439	3.277649
27	1	0	4.475098	-1.399491	1.493803
28	1	0	2.671624	0.502113	4.906853
29	1	0	4.688717	-0.427618	3.795171
30	45	0	2.277845	0.044067	-0.751836
31	6	0	2.590170	2.116841	-0.074852
32	6	0	3.818437	1.555200	-0.611270
33	6	0	1.618299	2.094882	-1.113390
34	6	0	3.593733	1.230459	-1.986305
35	6	0	2.222245	1.526156	-2.303022
36	6	0	2.413184	2.650714	1.309235
37	1	0	2.742787	3.694250	1.354111
38	1	0	1.366416	2.605488	1.617577
39	1	0	2.995523	2.070013	2.028721
40	6	0	5.113716	1.372000	0.111078
41	1	0	5.837497	2.121548	-0.226926
42	1	0	4.979606	1.475090	1.189232
43	1	0	5.502196	0.370066	-0.094069
44	6	0	4.602219	0.636151	-2.912199
45	1	0	5.144029	1.436643	-3.427377
46	1	0	5.306866	0.018227	-2.352770
47	1	0	4.114443	0.005017	-3.656658
48	6	0	1.573041	1.308227	-3.632601
49	1	0	1.828024	2.119333	-4.323084
50	1	0	1.907393	0.362007	-4.064758
51	1	0	0.485828	1.254802	-3.544502
52	6	0	0.190705	2.523838	-0.991238
53	1	0	-0.182477	2.346147	0.020421
54	1	0	0.098602	3.591607	-1.214770
55	1	0	-0.440467	1.969974	-1.690230

56	8	0	1.714915	-3.245277	0.047869
57	6	0	0.624717	-3.711629	0.533530
58	8	0	-0.117772	-3.140455	1.342620
59	6	0	0.237641	-5.089918	-0.001483
60	1	0	0.007233	-5.007144	-1.068373
61	1	0	1.080901	-5.777568	0.096433
62	1	0	-0.631221	-5.479040	0.529243
63	6	0	4.087094	-3.628628	-2.028157
64	1	0	5.092342	-4.014228	-1.862380
65	1	0	3.348241	-4.233665	-1.494717
66	1	0	3.838613	-3.641968	-3.091781
67	6	0	3.952826	-2.228132	-1.471344
68	8	0	4.808639	-1.737698	-0.731067
69	1	0	2.124160	-1.950436	0.621991
70	8	0	2.860225	-1.635572	-1.839546

II

Center Number	Atomic Number	Atomic Type	Coordinates (Angstroms) X	Y	Z
1	6	0	-1.328009	-0.070639	-1.008871
2	6	0	-0.756833	-0.552777	-2.193554
3	6	0	-1.309145	-0.197836	-3.421490
4	6	0	-2.419879	0.640680	-3.486233
5	6	0	-2.998489	1.112993	-2.309165
6	6	0	-2.465504	0.744831	-1.080395
7	1	0	0.101726	-1.215765	-2.149334
8	1	0	-0.870588	-0.589678	-4.334377
9	1	0	-3.870564	1.757603	-2.348833
10	1	0	-2.924552	1.099947	-0.160927
11	6	0	-0.841901	-0.469143	0.319297
12	7	0	0.393920	-0.671655	0.619568
13	6	0	-0.309998	-2.053531	2.449310
14	1	0	-1.052600	-1.483591	3.016097
15	1	0	-0.795467	-2.629490	1.652411
16	7	0	0.697605	-1.194205	1.843532
17	1	0	0.182331	-2.751137	3.121599
18	1	0	-2.843773	0.912174	-4.447872
19	1	0	-1.608315	-0.622161	1.076136
20	6	0	2.064818	-1.512864	2.010187
21	6	0	2.940833	-1.240890	0.950867
22	6	0	2.546149	-2.068156	3.196932

23	6	0	4.257755	-1.669989	1.045953
24	6	0	3.882194	-2.452168	3.289012
25	1	0	1.891823	-2.199403	4.052294
26	6	0	4.736408	-2.281080	2.207553
27	1	0	4.923656	-1.529986	0.198034
28	1	0	4.245678	-2.892345	4.211926
29	1	0	5.769272	-2.609737	2.264355
30	45	0	2.158693	-0.236180	-0.557543
31	6	0	2.474698	1.960685	-0.316751
32	6	0	3.747394	1.347621	-0.502054
33	6	0	1.743429	1.884036	-1.581963
34	6	0	3.716451	0.690089	-1.770889
35	6	0	2.488934	1.100702	-2.458527
36	6	0	2.041608	2.757278	0.875806
37	1	0	2.201155	3.829698	0.715243
38	1	0	0.978694	2.603485	1.081791
39	1	0	2.600260	2.458379	1.765644
40	6	0	4.878334	1.394846	0.478200
41	1	0	5.294328	2.407615	0.506504
42	1	0	4.552288	1.124709	1.485471
43	1	0	5.681492	0.711405	0.198828
44	6	0	4.783190	-0.139757	-2.416137
45	1	0	5.211729	0.373767	-3.283689
46	1	0	5.596256	-0.355172	-1.719044
47	1	0	4.358520	-1.092879	-2.748032
48	6	0	2.123060	0.617464	-3.825463
49	1	0	2.883869	0.905531	-4.558536
50	1	0	2.054975	-0.477735	-3.820683
51	1	0	1.162950	1.025055	-4.147191
52	6	0	0.443704	2.582260	-1.830208
53	1	0	-0.213046	2.514297	-0.959313
54	1	0	0.624427	3.645601	-2.025448
55	1	0	-0.093462	2.160458	-2.682122
56	8	0	2.126733	-2.029551	-1.669821
57	6	0	1.569365	-3.118093	-1.227361
58	8	0	0.865126	-3.214452	-0.228149
59	6	0	1.871455	-4.334762	-2.092179
60	1	0	1.697552	-4.101021	-3.144740
61	1	0	2.928412	-4.592185	-1.982347
62	1	0	1.256331	-5.178637	-1.782220

AcOH

Center Number	Atomic Number	Atomic Type	Coordinates (Angstroms) X	Y	Z
1	6	0	-3.959574	1.410072	-1.475917
2	1	0	-4.216356	0.857588	-2.377757
3	1	0	-4.451183	0.964395	-0.609195
4	1	0	-4.305230	2.442359	-1.556635
5	6	0	-2.473846	1.420767	-1.230297
6	8	0	-1.790491	0.753982	-2.180515
7	1	0	-0.853889	0.817667	-1.937324
8	8	0	-1.930621	1.957759	-0.298632

TS2

Center Number	Atomic Number	Atomic Type	Coordinates (Angstroms) X	Y	Z
1	6	0	-1.194643	-0.671511	-0.878504
2	6	0	-1.489488	0.608944	-1.362206
3	6	0	-2.305667	0.763689	-2.473800
4	6	0	-2.842519	-0.358668	-3.105871
5	6	0	-2.564549	-1.632639	-2.618376
6	6	0	-1.744773	-1.793499	-1.505115
7	1	0	-1.076492	1.469041	-0.845408
8	1	0	-2.534125	1.758701	-2.843596
9	1	0	-2.985834	-2.505321	-3.106901
10	1	0	-1.488025	-2.781686	-1.136405
11	6	0	-0.345314	-0.843183	0.314040
12	7	0	0.313386	0.161954	0.793761
13	6	0	0.106690	0.006775	3.159213
14	1	0	-0.512678	0.895459	3.021505
15	1	0	-0.549329	-0.873499	3.264213
16	7	0	1.010348	-0.085832	2.032514
17	1	0	0.687067	0.125001	4.076894
18	1	0	-3.483559	-0.237537	-3.973619
19	1	0	-0.312828	-1.827617	0.785198
20	6	0	1.943407	-1.148549	1.978122
21	6	0	2.656137	-1.316484	0.776566
22	6	0	2.219215	-1.971272	3.071758
23	6	0	3.619019	-2.306331	0.687385
24	6	0	3.219528	-2.940340	2.975352

25	1	0	1.660731	-1.868335	3.996467
26	6	0	3.917970	-3.115173	1.789296
27	1	0	4.126691	-2.467906	-0.261001
28	1	0	3.432887	-3.569795	3.833585
29	1	0	4.679392	-3.884549	1.708186
30	45	0	2.160579	-0.071859	-0.697346
31	6	0	2.705993	2.056846	-0.315650
32	6	0	3.875356	1.256107	-0.269575
33	6	0	2.206032	2.078997	-1.699080
34	6	0	3.979263	0.602766	-1.552006
35	6	0	2.982995	1.207197	-2.448468
36	6	0	2.126367	2.864433	0.802661
37	1	0	2.382300	3.923637	0.689813
38	1	0	1.037849	2.764239	0.814557
39	1	0	2.493318	2.512499	1.767993
40	6	0	4.780172	1.054470	0.905336
41	1	0	5.583348	1.798800	0.899119
42	1	0	4.228069	1.145983	1.843359
43	1	0	5.226159	0.057851	0.891004
44	6	0	5.038965	-0.346762	-2.013458
45	1	0	5.771349	0.163049	-2.649290
46	1	0	5.569848	-0.786743	-1.166683
47	1	0	4.581406	-1.156685	-2.590148
48	6	0	2.795146	0.779794	-3.867594
49	1	0	3.714741	0.926434	-4.443133
50	1	0	2.537308	-0.285734	-3.888748
51	1	0	1.993348	1.342897	-4.348943
52	6	0	1.042882	2.891957	-2.180071
53	1	0	0.414339	3.205813	-1.343417
54	1	0	1.389111	3.798521	-2.688264
55	1	0	0.419550	2.325686	-2.877070
56	8	0	1.896998	-1.677804	-2.024469
57	6	0	1.311766	-2.803659	-1.778942
58	8	0	0.795551	-3.139473	-0.715178
59	6	0	1.238289	-3.726862	-2.987661
60	1	0	0.394205	-3.406803	-3.607501
61	1	0	2.144890	-3.657879	-3.590422
62	1	0	1.067324	-4.752984	-2.662887

II′

Center Number	Atomic Number	Atomic Type	Coordinates (Angstroms) X	Y	Z
1	6	0	-1.186381	-0.165262	-0.823237
2	6	0	-1.902903	1.017243	-0.593294
3	6	0	-2.770503	1.512631	-1.558896
4	6	0	-2.938951	0.838244	-2.767943
5	6	0	-2.240494	-0.345617	-2.997128
6	6	0	-1.369604	-0.847578	-2.033400
7	1	0	-1.777840	1.530629	0.355117
8	1	0	-3.321190	2.428811	-1.367440
9	1	0	-2.376397	-0.883980	-3.930115
10	1	0	-0.828844	-1.777216	-2.194320
11	6	0	-0.218674	-0.701011	0.151876
12	7	0	-0.025348	-0.040487	1.257579
13	6	0	0.765440	0.263892	3.437304
14	1	0	0.241380	1.204434	3.265492
15	1	0	0.197649	-0.320657	4.171330
16	7	0	0.868153	-0.418159	2.159806
17	1	0	1.765018	0.453761	3.838194
18	1	0	-3.616312	1.228603	-3.520724
19	1	0	0.185275	-1.707319	-0.036431
20	6	0	1.867389	-1.410351	1.966031
21	6	0	2.549026	-1.527093	0.738901
22	6	0	2.232742	-2.205651	3.057365
23	6	0	3.580652	-2.458994	0.650703
24	6	0	3.282707	-3.110471	2.950460
25	1	0	1.682662	-2.122619	3.989859
26	6	0	3.959598	-3.239483	1.743475
27	1	0	4.083210	-2.585348	-0.305378
28	1	0	3.556321	-3.719320	3.805786
29	1	0	4.772524	-3.952282	1.644699
30	45	0	2.057805	-0.396164	-0.838084
31	6	0	2.229925	1.810199	-0.531927
32	6	0	3.491024	1.193446	-0.322570
33	6	0	1.876161	1.683672	-1.953686
34	6	0	3.830653	0.513107	-1.548336
35	6	0	2.852150	0.910952	-2.569363
36	6	0	1.468786	2.630041	0.462901
37	1	0	1.727885	3.690079	0.361710
38	1	0	0.392307	2.519324	0.317005
39	1	0	1.699907	2.315054	1.483036
40	6	0	4.284296	1.181915	0.946889
41	1	0	4.972621	2.032804	0.977222

42	1	0	3.626896	1.238677	1.818325
43	1	0	4.863888	0.260584	1.037411
44	6	0	5.052373	-0.296012	-1.841847
45	1	0	5.765039	0.278825	-2.443355
46	1	0	5.551437	-0.606257	-0.921409
47	1	0	4.768034	-1.193627	-2.400237
48	6	0	2.872766	0.405466	-3.975080
49	1	0	3.786178	0.719630	-4.490587
50	1	0	2.838626	-0.690072	-3.966661
51	1	0	2.012215	0.775079	-4.536402
52	6	0	0.646643	2.249368	-2.601666
53	1	0	0.082177	2.868249	-1.902428
54	1	0	0.923307	2.872817	-3.457422
55	1	0	-0.026069	1.460971	-2.955401
56	8	0	2.280223	-2.102482	-2.049792
57	6	0	1.604436	-3.197494	-1.908309
58	8	0	0.653812	-3.361452	-1.146758
59	6	0	2.107221	-4.338059	-2.780150
60	1	0	2.250983	-3.996472	-3.807318
61	1	0	3.079969	-4.665026	-2.401501
62	1	0	1.407472	-5.171928	-2.748788

TS3

Center Number	Atomic Number	Atomic Type	Coordinates (Angstroms) X	Y	Z
1	6	0	-1.024236	0.081244	-0.944213
2	6	0	-1.943907	1.062462	-0.546993
3	6	0	-2.851295	1.593813	-1.456825
4	6	0	-2.867003	1.151001	-2.779022
5	6	0	-1.968705	0.165913	-3.180348
6	6	0	-1.055512	-0.362818	-2.272914
7	1	0	-1.930753	1.401641	0.483908
8	1	0	-3.550310	2.359490	-1.133240
9	1	0	-1.975404	-0.190453	-4.206210
10	1	0	-0.337390	-1.112912	-2.597228
11	6	0	0.001212	-0.477784	-0.024293
12	7	0	-0.100048	-0.083415	1.217894
13	6	0	0.368758	-0.023473	3.494941
14	1	0	-0.298942	0.828199	3.380059

15	1	0	-0. 158126	-0. 815699	4. 040130
16	7	0	0. 753744	-0. 447718	2. 158694
17	1	0	1. 255957	0. 268711	4. 062150
18	1	0	-3. 575238	1. 568221	-3. 487687
19	1	0	0. 279156	-1. 728792	-0. 293740
20	6	0	1. 844011	-1. 340761	1. 992808
21	6	0	2. 525798	-1. 454996	0. 771043
22	6	0	2. 263581	-2. 091084	3. 102006
23	6	0	3. 598522	-2. 343676	0. 692790
24	6	0	3. 352912	-2. 947435	3. 005903
25	1	0	1. 724321	-2. 027111	4. 040416
26	6	0	4. 026032	-3. 079656	1. 796656
27	1	0	4. 105101	-2. 464528	-0. 262276
28	1	0	3. 659808	-3. 520240	3. 874891
29	1	0	4. 872677	-3. 753626	1. 708985
30	45	0	2. 044104	-0. 400614	-0. 851166
31	6	0	2. 334643	1. 764290	-0. 588434
32	6	0	3. 606146	1. 124057	-0. 412680
33	6	0	1. 971224	1. 678459	-2. 000116
34	6	0	3. 926018	0. 476307	-1. 639089
35	6	0	2. 914444	0. 858684	-2. 623307
36	6	0	1. 605602	2. 584636	0. 432171
37	1	0	1. 954773	3. 623272	0. 421261
38	1	0	0. 530999	2. 578802	0. 231057
39	1	0	1. 756417	2. 179854	1. 436377
40	6	0	4. 414510	1. 109919	0. 847625
41	1	0	5. 083767	1. 976234	0. 878318
42	1	0	3. 767922	1. 144899	1. 727929
43	1	0	5. 018240	0. 202893	0. 923133
44	6	0	5. 150173	-0. 326414	-1. 953012
45	1	0	5. 882876	0. 272014	-2. 506478
46	1	0	5. 630568	-0. 685344	-1. 039799
47	1	0	4. 895929	-1. 196876	-2. 565223
48	6	0	2. 917283	0. 364481	-4. 036239
49	1	0	3. 789439	0. 743071	-4. 579967
50	1	0	2. 956595	-0. 729525	-4. 054818
51	1	0	2. 018620	0. 679611	-4. 569145
52	6	0	0. 803870	2. 374814	-2. 626434
53	1	0	-0. 075720	2. 354517	-1. 978332
54	1	0	1. 055003	3. 423629	-2. 822183
55	1	0	0. 518999	1. 910873	-3. 572280
56	8	0	1. 975103	-2. 277345	-1. 911068
57	6	0	1. 177374	-3. 180099	-1. 525191
58	8	0	0. 285531	-3. 014489	-0. 649543

59	6	0	1.328450	-4.556016	-2.130162
60	1	0	0.356016	-5.041962	-2.205631
61	1	0	1.813630	-4.493348	-3.103637
62	1	0	1.957542	-5.149257	-1.460143

III

Center Number	Atomic Number	Atomic Type	Coordinates (Angstroms) X	Y	Z
1	6	0	-0.706839	-0.134172	-1.093545
2	6	0	-1.824639	0.687760	-0.900733
3	6	0	-2.683991	0.981266	-1.953504
4	6	0	-2.452164	0.447847	-3.220496
5	6	0	-1.359824	-0.392710	-3.420231
6	6	0	-0.495430	-0.677971	-2.366044
7	1	0	-1.997104	1.099993	0.088608
8	1	0	-3.536170	1.633596	-1.787420
9	1	0	-1.182161	-0.832359	-4.397623
10	1	0	0.356170	-1.336630	-2.523145
11	6	0	0.260099	-0.394241	0.015804
12	7	0	-0.308093	-0.511235	1.167434
13	6	0	-0.484482	-0.843949	3.466803
14	1	0	-1.448675	-0.432663	3.179177
15	1	0	-0.616286	-1.884606	3.787966
16	7	0	0.377126	-0.748271	2.301419
17	1	0	-0.073545	-0.266267	4.299659
18	1	0	-3.122526	0.678233	-4.042441
19	6	0	1.692095	-1.194566	2.366727
20	6	0	2.552222	-1.123143	1.251960
21	6	0	2.163318	-1.738811	3.585327
22	6	0	3.849540	-1.654494	1.398014
23	6	0	3.443423	-2.250819	3.680450
24	1	0	1.522945	-1.775486	4.457322
25	6	0	4.298430	-2.229185	2.576262
26	1	0	4.525580	-1.612573	0.545661
27	1	0	3.775872	-2.676401	4.622380
28	1	0	5.298559	-2.645125	2.642766
29	45	0	2.170556	-0.267373	-0.485933
30	6	0	2.792094	1.747449	-0.505971
31	6	0	4.078971	1.067423	-0.653903

32	6	0	2.103569	1.679539	-1.794718
33	6	0	4.039479	0.390458	-1.865447
34	6	0	2.803689	0.770552	-2.576844
35	6	0	2.447247	2.681837	0.612086
36	1	0	2.863767	3.679107	0.425308
37	1	0	1.364650	2.777339	0.720699
38	1	0	2.848016	2.313456	1.559150
39	6	0	5.170840	1.108876	0.368905
40	1	0	5.634372	2.101672	0.381107
41	1	0	4.784878	0.900523	1.370188
42	1	0	5.953249	0.377115	0.156951
43	6	0	5.069327	-0.530873	-2.442171
44	1	0	5.543660	-0.082427	-3.322407
45	1	0	5.852915	-0.759309	-1.717003
46	1	0	4.618670	-1.476174	-2.760062
47	6	0	2.459658	0.290742	-3.952299
48	1	0	3.089460	0.779542	-4.704565
49	1	0	2.620439	-0.788225	-4.043534
50	1	0	1.414550	0.498082	-4.191637
51	6	0	0.864608	2.442712	-2.141418
52	1	0	0.208813	2.546282	-1.274148
53	1	0	1.130594	3.448736	-2.485277
54	1	0	0.286937	1.949599	-2.927209

TS4

Center Number	Atomic Number	Atomic Type	Coordinates (Angstroms) X	Y	Z
1	6	0	-0.248982	-1.432604	-0.980689
2	6	0	-1.199485	-0.742965	-1.782959
3	6	0	-1.308473	-1.033006	-3.121431
4	6	0	-0.460162	-1.991086	-3.722218
5	6	0	0.455402	-2.680650	-2.957671
6	6	0	0.575506	-2.423646	-1.573833
7	1	0	-1.817255	0.015862	-1.312789
8	1	0	-2.043557	-0.512293	-3.727569
9	1	0	1.078747	-3.446384	-3.409070
10	1	0	1.167241	-3.079193	-0.944496
11	6	0	0.164514	-0.954262	0.323675
12	7	0	-0.464100	-0.513640	1.342810

13	6	0	-0.243867	0.218404	3.585586
14	1	0	-1.211157	0.657833	3.348968
15	1	0	-0.390176	-0.671092	4.212513
16	7	0	0.405012	-0.107939	2.330545
17	1	0	0.353393	0.950072	4.134334
18	1	0	-0.551803	-2.199629	-4.783279
19	6	0	1.611619	-0.800066	2.321083
20	6	0	1.971976	-1.405499	1.097288
21	6	0	2.378770	-0.993603	3.481613
22	6	0	3.030102	-2.331901	1.118185
23	6	0	3.460818	-1.856616	3.443758
24	1	0	2.112442	-0.496047	4.407885
25	6	0	3.774733	-2.551462	2.269533
26	1	0	3.312447	-2.839185	0.198398
27	1	0	4.047715	-2.015891	4.342920
28	1	0	4.610148	-3.244223	2.252345
29	45	0	1.772699	-0.425112	-0.742194
30	6	0	3.174822	1.196963	-0.239768
31	6	0	3.860596	0.435439	-1.255552
32	6	0	2.059273	1.896191	-0.853189
33	6	0	3.063064	0.510166	-2.414275
34	6	0	1.968079	1.447326	-2.177611
35	6	0	3.690972	1.429664	1.147783
36	1	0	4.460658	2.210242	1.143573
37	1	0	2.887796	1.746934	1.818146
38	1	0	4.127931	0.516632	1.563703
39	6	0	5.142058	-0.313785	-1.053989
40	1	0	6.006512	0.359403	-1.090687
41	1	0	5.155224	-0.817372	-0.082030
42	1	0	5.282387	-1.076019	-1.824684
43	6	0	3.296730	-0.165043	-3.731422
44	1	0	3.676278	0.546209	-4.473950
45	1	0	4.020818	-0.977554	-3.640455
46	1	0	2.364269	-0.585991	-4.122903
47	6	0	0.950261	1.818316	-3.211206
48	1	0	1.375491	2.487540	-3.967938
49	1	0	0.573292	0.928960	-3.727518
50	1	0	0.092032	2.322614	-2.760713
51	6	0	1.146817	2.843819	-0.134702
52	1	0	0.895631	2.461373	0.859107
53	1	0	1.609042	3.829646	-0.011350
54	1	0	0.209749	2.978796	-0.679971

Cp*Rh(I)

Center Number	Atomic Number	Atomic Type	Coordinates (Angstroms) X	Y	Z
1	45	0	-4.598555	-0.323288	-0.522850
2	6	0	-4.184355	-2.667563	0.477089
3	6	0	-3.306824	-2.056590	-0.557360
4	6	0	-4.210921	-1.818388	1.544227
5	6	0	-2.601210	-0.956930	0.026211
6	6	0	-3.349144	-0.652743	1.208370
7	6	0	-4.949589	-3.933384	0.254434
8	1	0	-4.275907	-4.775832	0.058947
9	1	0	-5.563736	-4.188813	1.120502
10	1	0	-5.614807	-3.849296	-0.612492
11	6	0	-2.940499	-2.766311	-1.826825
12	1	0	-2.249683	-3.596075	-1.630466
13	1	0	-3.827567	-3.187110	-2.310232
14	1	0	-2.459725	-2.088396	-2.536216
15	6	0	-1.353399	-0.292538	-0.470168
16	1	0	-0.457936	-0.765869	-0.047086
17	1	0	-1.277526	-0.352619	-1.558713
18	1	0	-1.330531	0.765265	-0.196662
19	6	0	-3.030972	0.429249	2.197014
20	1	0	-2.358638	0.060718	2.982040
21	1	0	-2.546596	1.279830	1.711591
22	1	0	-3.938977	0.792432	2.688167
23	6	0	-5.015067	-1.902972	2.802654
24	1	0	-5.616535	-2.813896	2.834401
25	1	0	-4.370438	-1.893548	3.689140
26	1	0	-5.697158	-1.050182	2.894474

2a

Center Number	Atomic Number	Atomic Type	Coordinates (Angstroms) X	Y	Z
1	6	0	-12.373749	3.924353	-3.059502
2	6	0	-11.342879	4.925126	-3.020281
3	6	0	-11.627328	5.665953	-1.855228

4	1	0	-9.937838	4.676455	-4.658504
5	6	0	-10.207711	5.255645	-3.781904
6	6	0	-10.845026	6.754983	-1.439449
7	6	0	-9.752335	7.068680	-2.221285
8	6	0	-9.429796	6.321988	-3.377590
9	1	0	-11.086539	7.319560	-0.544945
10	1	0	-9.119204	7.903579	-1.938719
11	1	0	-8.550157	6.593122	-3.951365
12	7	0	-12.729101	5.095068	-1.294498
13	7	0	-13.177915	4.052539	-2.019550
14	6	0	-13.429524	5.518508	-0.105864
15	1	0	-12.743525	5.561048	0.744903
16	1	0	-13.886857	6.502110	-0.249722
17	1	0	-14.206529	4.781543	0.089608
18	6	0	-12.591623	2.859083	-4.050393
19	6	0	-12.224304	3.039114	-5.387401
20	6	0	-13.187859	1.652926	-3.665945
21	6	0	-12.433627	2.026454	-6.318465
22	1	0	-11.804116	3.988425	-5.704761
23	6	0	-13.402315	0.646377	-4.599024
24	1	0	-13.475276	1.521246	-2.627946
25	6	0	-13.020408	0.826669	-5.927304
26	1	0	-12.147525	2.180574	-7.354134
27	1	0	-13.863787	-0.285768	-4.288187
28	1	0	-13.183137	0.037509	-6.654277

IV

Center Number	Atomic Number	Atomic Type	Coordinates (Angstroms) X	Y	Z
1	45	0	-4.598555	-0.323288	-0.522850
2	6	0	-4.184355	-2.667563	0.477089
3	6	0	-3.306824	-2.056590	-0.557360
4	6	0	-4.210921	-1.818388	1.544227
5	6	0	-2.601210	-0.956930	0.026211
6	6	0	-3.349144	-0.652743	1.208370
7	6	0	-4.949589	-3.933384	0.254434
8	1	0	-4.275907	-4.775832	0.058947
9	1	0	-5.563736	-4.188813	1.120502
10	1	0	-5.614807	-3.849296	-0.612492

11	6	0	-2.940499	-2.766311	-1.826825
12	1	0	-2.249683	-3.596075	-1.630466
13	1	0	-3.827567	-3.187110	-2.310232
14	1	0	-2.459725	-2.088396	-2.536216
15	6	0	-1.353399	-0.292538	-0.470168
16	1	0	-0.457936	-0.765869	-0.047086
17	1	0	-1.277526	-0.352619	-1.558713
18	1	0	-1.330531	0.765265	-0.196662
19	6	0	-3.030972	0.429249	2.197014
20	1	0	-2.358638	0.060718	2.982040
21	1	0	-2.546596	1.279830	1.711591
22	1	0	-3.938977	0.792432	2.688167
23	6	0	-5.015067	-1.902972	2.802654
24	1	0	-5.616535	-2.813896	2.834401
25	1	0	-4.370438	-1.893548	3.689140
26	1	0	-5.697158	-1.050182	2.894474

1A

Center Number	Atomic Number	Atomic Type	Coordinates (Angstroms) X	Y	Z
1	6	0	-1.425589	0.687305	-0.868850
2	6	0	-1.051790	-0.108104	-1.959983
3	6	0	-1.460051	0.209266	-3.246733
4	6	0	-2.254759	1.332638	-3.463034
5	6	0	-2.630977	2.125103	-2.384395
6	6	0	-2.229924	1.822807	-1.082081
7	1	0	-0.439310	-0.983599	-1.771452
8	1	0	-1.162425	-0.419029	-4.080135
9	1	0	-3.250305	3.002575	-2.551322
10	6	0	-0.983610	0.337067	0.490619
11	7	0	-0.169854	-0.641511	0.668162
12	6	0	-0.140141	-0.207489	3.053660
13	1	0	0.044470	0.854822	2.852315
14	1	0	-1.201135	-0.333265	3.305120
15	7	0	0.239114	-0.986918	1.897188
16	1	0	0.471566	-0.500805	3.903184
17	1	0	-2.581054	1.590346	-4.465497
18	1	0	-1.383538	0.924458	1.315872
19	6	0	1.113543	-2.087900	1.981937

20	6	0	1.855184	-2.485333	0.861065
21	6	0	1.247592	-2.805411	3.175942
22	6	0	2.718864	-3.567888	0.949013
23	1	0	1.744046	-1.933639	-0.063561
24	6	0	2.126632	-3.881562	3.250574
25	1	0	0.646408	-2.551721	4.041827
26	6	0	2.869467	-4.271739	2.142438
27	1	0	3.289986	-3.857786	0.072422
28	1	0	2.214231	-4.425734	4.185760
29	1	0	3.550499	-5.113419	2.203899
30	6	0	-2.659829	2.715556	0.056939
31	1	0	-3.273056	2.176135	0.786726
32	1	0	-1.799798	3.129770	0.593493
33	1	0	-3.252230	3.552751	-0.317396

1B

Center Number	Atomic Number	Atomic Type	Coordinates (Angstroms) X	Y	Z
1	6	0	-1.411913	0.709680	-0.856734
2	6	0	-1.049939	-0.091435	-1.951613
3	6	0	-1.458932	0.214568	-3.239145
4	6	0	-2.250279	1.338747	-3.473037
5	6	0	-2.626323	2.149213	-2.409844
6	6	0	-2.207710	1.829921	-1.122500
7	1	0	-0.440840	-0.966737	-1.752719
8	1	0	-1.164230	-0.424808	-4.064624
9	1	0	-3.240508	3.028437	-2.567293
10	6	0	-0.969040	0.368095	0.502063
11	7	0	-0.167735	-0.622634	0.669895
12	6	0	-0.118851	-0.177969	3.050368
13	1	0	0.078949	0.880835	2.845441
14	1	0	-1.180767	-0.290289	3.302511
15	7	0	0.247248	-0.966190	1.893685
16	1	0	0.491139	-0.478438	3.898488
17	1	0	-2.576158	1.585604	-4.477832
18	1	0	-1.356666	0.972407	1.318133
19	6	0	1.110778	-2.077260	1.977942
20	6	0	1.853536	-2.476960	0.859321
21	6	0	1.231350	-2.799054	3.170297

22	6	0	2.705243	-3.569120	0.946882
23	1	0	1.754631	-1.919195	-0.063175
24	6	0	2.098650	-3.884623	3.245110
25	1	0	0.629226	-2.540534	4.034101
26	6	0	2.842052	-4.278521	2.138574
27	1	0	3.278188	-3.861598	0.072416
28	1	0	2.176739	-4.432931	4.178641
29	1	0	3.513975	-5.127471	2.200151
30	35	0	-2.758715	2.988278	0.282268

1A-TS1

Center Number	Atomic Number	Atomic Type	Coordinates (Angstroms) X	Y	Z
1	6	0	-0.822002	-1.011318	-2.224936
2	6	0	0.171852	-1.849150	-2.737980
3	6	0	0.276462	-2.038727	-4.113132
4	6	0	-0.621569	-1.411711	-4.970677
5	6	0	-1.639862	-0.612089	-4.451952
6	6	0	-1.766907	-0.407922	-3.078756
7	1	0	0.853616	-2.348026	-2.053267
8	1	0	1.056643	-2.684206	-4.504061
9	1	0	-2.352928	-0.142084	-5.124180
10	6	0	-0.896066	-0.785649	-0.774288
11	7	0	0.162529	-0.561040	-0.079405
12	6	0	-1.213538	-0.553183	1.918435
13	1	0	-1.935029	0.222227	1.634872
14	1	0	-1.573726	-1.553428	1.659271
15	7	0	0.062993	-0.327310	1.260489
16	1	0	-1.058020	-0.521234	2.993080
17	1	0	-0.546393	-1.556420	-6.044158
18	1	0	-1.874924	-0.813156	-0.301896
19	6	0	1.271137	-0.416819	1.969433
20	6	0	2.390260	-0.991778	1.327671
21	6	0	1.376251	0.114431	3.260547
22	6	0	3.625597	-0.959194	1.997676
23	6	0	2.608958	0.099878	3.900469
24	1	0	0.522524	0.571016	3.750236
25	6	0	3.745394	-0.418264	3.271720
26	1	0	4.479961	-1.399192	1.490393

27	1	0	2.687238	0.518250	4.899928
28	1	0	4.700815	-0.417597	3.786344
29	45	0	2.286433	0.040304	-0.755649
30	6	0	2.599975	2.114208	-0.086019
31	6	0	3.831058	1.547436	-0.609872
32	6	0	1.637446	2.089211	-1.133036
33	6	0	3.617148	1.215764	-1.985304
34	6	0	2.249289	1.513783	-2.314997
35	6	0	2.410226	2.654985	1.293589
36	1	0	2.734033	3.700458	1.335363
37	1	0	1.361074	2.605824	1.593552
38	1	0	2.989710	2.081051	2.020699
39	6	0	5.120898	1.366033	0.122280
40	1	0	5.848714	2.110776	-0.217710
41	1	0	4.980803	1.478107	1.198708
42	1	0	5.507779	0.361555	-0.073353
43	6	0	4.632927	0.615134	-2.899145
44	1	0	5.168952	1.411676	-3.426294
45	1	0	5.340990	0.011791	-2.328531
46	1	0	4.152254	-0.032911	-3.633620
47	6	0	1.607731	1.297009	-3.648262
48	1	0	1.815076	2.139927	-4.316354
49	1	0	1.990782	0.383439	-4.107818
50	1	0	0.524901	1.179596	-3.559661
51	6	0	0.209273	2.518857	-1.030191
52	1	0	-0.184493	2.329703	-0.028215
53	1	0	0.120571	3.588704	-1.244969
54	1	0	-0.404176	1.971255	-1.749466
55	8	0	1.710017	-3.244024	0.050814
56	6	0	0.616822	-3.710720	0.528289
57	8	0	-0.130041	-3.142422	1.335689
58	6	0	0.232487	-5.087415	-0.013003
59	1	0	0.022505	-5.004383	-1.083951
60	1	0	1.070836	-5.778707	0.100929
61	1	0	-0.647077	-5.472614	0.502754
62	6	0	4.094516	-3.643947	-2.007796
63	1	0	5.098368	-4.031278	-1.837608
64	1	0	3.352364	-4.243317	-1.472574
65	1	0	3.847868	-3.663145	-3.071748
66	6	0	3.962762	-2.239726	-1.459986
67	8	0	4.818914	-1.746525	-0.721673
68	1	0	2.124465	-1.950542	0.625998
69	8	0	2.872070	-1.647196	-1.833417
70	6	0	-2.898921	0.426604	-2.530721

71	1	0	-3.620005	-0.197107	-1.990260
72	1	0	-2.549201	1.191298	-1.829272
73	1	0	-3.436767	0.928258	-3.337686

1B-TS1

Center Number	Atomic Number	Atomic Type	Coordinates (Angstroms) X	Y	Z
1	6	0	-0.670714	-1.127254	-2.315459
2	6	0	0.199752	-2.151364	-2.702254
3	6	0	0.345723	-2.471413	-4.049230
4	6	0	-0.389153	-1.795186	-5.018641
5	6	0	-1.271872	-0.780820	-4.651627
6	6	0	-1.398488	-0.465778	-3.307820
7	1	0	0.773100	-2.662452	-1.932774
8	1	0	1.033757	-3.259541	-4.336177
9	1	0	-1.843787	-0.234713	-5.392834
10	6	0	-0.849272	-0.806855	-0.887833
11	7	0	0.164771	-0.546917	-0.146105
12	6	0	-1.296211	-0.489508	1.782515
13	1	0	-2.001596	0.268091	1.423246
14	1	0	-1.641576	-1.502195	1.551836
15	7	0	0.010890	-0.284642	1.181826
16	1	0	-1.198655	-0.402651	2.861109
17	1	0	-0.281211	-2.051536	-6.067754
18	1	0	-1.859454	-0.825445	-0.487158
19	6	0	1.195724	-0.396089	1.932862
20	6	0	2.329752	-0.962537	1.307604
21	6	0	1.269483	0.102327	3.237819
22	6	0	3.548794	-0.954031	2.006315
23	6	0	2.485240	0.054642	3.910465
24	1	0	0.407691	0.551243	3.720137
25	6	0	3.635899	-0.454966	3.300503
26	1	0	4.413653	-1.381808	1.504645
27	1	0	2.538607	0.442211	4.923838
28	1	0	4.575442	-0.476659	3.843060
29	45	0	2.247970	0.106540	-0.736522
30	6	0	2.690877	2.147904	-0.002196
31	6	0	3.847617	1.559408	-0.658014
32	6	0	1.636527	2.207019	-0.949017

33	6	0	3.499315	1.309761	-2.022686
34	6	0	2.119777	1.666497	-2.209207
35	6	0	2.666392	2.624613	1.413355
36	1	0	3.171765	3.593645	1.486136
37	1	0	1.644664	2.741933	1.777648
38	1	0	3.177339	1.918532	2.073278
39	6	0	5.185807	1.323327	-0.036156
40	1	0	5.876189	2.126388	-0.316530
41	1	0	5.104815	1.297233	1.052575
42	1	0	5.581693	0.361273	-0.367533
43	6	0	4.393406	0.703602	-3.053131
44	1	0	4.934964	1.493736	-3.584057
45	1	0	5.110168	0.030905	-2.577858
46	1	0	3.811116	0.127394	-3.774184
47	6	0	1.355419	1.541614	-3.490589
48	1	0	1.730180	2.258884	-4.228452
49	1	0	1.458134	0.532980	-3.905269
50	1	0	0.290876	1.733519	-3.340257
51	6	0	0.247533	2.694153	-0.684592
52	1	0	-0.021200	2.542568	0.363500
53	1	0	0.174390	3.763004	-0.910979
54	1	0	-0.486874	2.166755	-1.296635
55	8	0	1.701336	-3.231916	0.070446
56	6	0	0.628513	-3.708812	0.591284
57	8	0	-0.148685	-3.102308	1.337103
58	6	0	0.322576	-5.149214	0.185841
59	1	0	0.141465	-5.190017	-0.892926
60	1	0	1.187979	-5.782765	0.393871
61	1	0	-0.553678	-5.519929	0.717297
62	6	0	3.957940	-3.571457	-2.127791
63	1	0	4.964773	-3.970491	-2.010013
64	1	0	3.236599	-4.190692	-1.586282
65	1	0	3.673569	-3.547635	-3.182373
66	6	0	3.858327	-2.188354	-1.519703
67	8	0	4.735659	-1.739603	-0.780869
68	1	0	2.078063	-1.935251	0.598205
69	8	0	2.767893	-1.566474	-1.846579
70	35	0	-2.523826	0.982704	-2.815347

1A-TS3

Center Number	Atomic Number	Atomic Type	Coordinates (Angstroms) X	Y	Z
1	6	0	-1.026669	0.175491	-1.006020
2	6	0	-1.726510	1.270673	-0.469216
3	6	0	-2.651885	1.993880	-1.209350
4	6	0	-2.910651	1.632945	-2.527956
5	6	0	-2.240035	0.543592	-3.069239
6	6	0	-1.301958	-0.194518	-2.339964
7	1	0	-1.528365	1.544851	0.561125
8	1	0	-3.165233	2.836793	-0.756589
9	1	0	-2.443840	0.247113	-4.095283
10	6	0	-0.009877	-0.528643	-0.153612
11	7	0	-0.188312	-0.272449	1.120869
12	6	0	0.071796	-0.404288	3.419890
13	1	0	-0.676424	0.376766	3.305578
14	1	0	-0.401052	-1.286454	3.866595
15	7	0	0.595415	-0.688982	2.091529
16	1	0	0.877435	-0.062780	4.074711
17	1	0	-3.626385	2.188092	-3.125893
18	1	0	0.309570	-1.789771	-0.414464
19	6	0	1.781503	-1.452664	1.963318
20	6	0	2.556801	-1.428227	0.794691
21	6	0	2.193709	-2.217229	3.067291
22	6	0	3.719931	-2.201784	0.767584
23	6	0	3.369727	-2.952512	3.021806
24	1	0	1.581781	-2.261444	3.960488
25	6	0	4.141271	-2.949445	1.865191
26	1	0	4.304443	-2.230818	-0.148940
27	1	0	3.667815	-3.538008	3.885382
28	1	0	5.058044	-3.528608	1.811857
29	45	0	2.086547	-0.419775	-0.867912
30	6	0	2.333619	1.754372	-0.681825
31	6	0	3.615284	1.149082	-0.452361
32	6	0	2.007172	1.602658	-2.098203
33	6	0	3.979241	0.463083	-1.643014
34	6	0	2.982514	0.778140	-2.663773
35	6	0	1.577386	2.605369	0.292066
36	1	0	1.941190	3.638874	0.274235
37	1	0	0.512809	2.612648	0.047136
38	1	0	1.683744	2.220278	1.309887
39	6	0	4.392775	1.207106	0.825646
40	1	0	5.032522	2.095896	0.839075
41	1	0	3.723702	1.253399	1.688137

42	1	0	5.023140	0.323891	0.949888
43	6	0	5.227362	-0.321626	-1.905683
44	1	0	5.911195	0.240303	-2.551864
45	1	0	5.755256	-0.548563	-0.976970
46	1	0	4.994867	-1.267775	-2.404963
47	6	0	3.036055	0.234087	-4.056981
48	1	0	3.907134	0.624589	-4.594185
49	1	0	3.117170	-0.857646	-4.033052
50	1	0	2.140627	0.497809	-4.622563
51	6	0	0.858257	2.251312	-2.805918
52	1	0	0.007443	2.415362	-2.141410
53	1	0	1.170038	3.223899	-3.203625
54	1	0	0.504903	1.640897	-3.639942
55	8	0	2.197107	-2.375011	-1.802557
56	6	0	1.440727	-3.287874	-1.369051
57	8	0	0.456896	-3.090113	-0.599459
58	6	0	1.753733	-4.711151	-1.761640
59	1	0	0.846114	-5.313568	-1.765578
60	1	0	2.248998	-4.737294	-2.731999
61	1	0	2.438209	-5.116810	-1.010322
62	6	0	-0.627198	-1.357104	-3.021218
63	1	0	0.459292	-1.229270	-3.066997
64	1	0	-0.818446	-2.292864	-2.489450
65	1	0	-1.000741	-1.463010	-4.042622

1B-TS3

Center Number	Atomic Number	Atomic Type	Coordinates (Angstroms) X	Y	Z
1	6	0	-1.070784	0.170171	-1.004161
2	6	0	-1.846054	1.194942	-0.421655
3	6	0	-2.803769	1.908463	-1.124052
4	6	0	-3.043286	1.619492	-2.466028
5	6	0	-2.303146	0.619416	-3.076989
6	6	0	-1.332532	-0.079290	-2.358623
7	1	0	-1.669433	1.414904	0.624486
8	1	0	-3.364366	2.690920	-0.622436
9	1	0	-2.458640	0.374746	-4.121564
10	6	0	-0.039344	-0.537756	-0.174944
11	7	0	-0.186437	-0.250118	1.096822

12	6	0	0.109093	-0.320680	3.394004
13	1	0	-0.609765	0.486651	3.271683
14	1	0	-0.388945	-1.167615	3.879155
15	7	0	0.591790	-0.669576	2.064836
16	1	0	0.943099	0.007037	4.019600
17	1	0	-3.791030	2.165174	-3.031514
18	1	0	0.284650	-1.771004	-0.458114
19	6	0	1.766784	-1.454147	1.941734
20	6	0	2.549585	-1.437868	0.778826
21	6	0	2.161957	-2.219212	3.050840
22	6	0	3.709233	-2.216623	0.764066
23	6	0	3.333806	-2.961662	3.017646
24	1	0	1.540066	-2.256329	3.937754
25	6	0	4.116193	-2.962699	1.867985
26	1	0	4.298961	-2.253591	-0.148412
27	1	0	3.620999	-3.547992	3.884247
28	1	0	5.030200	-3.546941	1.824292
29	45	0	2.082958	-0.436455	-0.889341
30	6	0	2.318666	1.741196	-0.701614
31	6	0	3.597334	1.137560	-0.471375
32	6	0	1.993158	1.590100	-2.120016
33	6	0	3.956289	0.443113	-1.662447
34	6	0	2.966073	0.767686	-2.688507
35	6	0	1.553744	2.584903	0.272526
36	1	0	1.901696	3.623797	0.251330
37	1	0	0.487233	2.576862	0.033429
38	1	0	1.669313	2.203771	1.290831
39	6	0	4.377783	1.197084	0.804588
40	1	0	5.020648	2.083700	0.813124
41	1	0	3.711223	1.248664	1.668855
42	1	0	5.005452	0.312264	0.930551
43	6	0	5.200845	-0.346767	-1.924810
44	1	0	5.883436	0.209690	-2.576913
45	1	0	5.731560	-0.570681	-0.996992
46	1	0	4.960334	-1.294135	-2.417836
47	6	0	3.011977	0.212441	-4.076935
48	1	0	3.901722	0.565269	-4.609591
49	1	0	3.048374	-0.881718	-4.039158
50	1	0	2.128135	0.498322	-4.648837
51	6	0	0.844393	2.246958	-2.820793
52	1	0	-0.009772	2.396179	-2.155868
53	1	0	1.153600	3.230104	-3.193656
54	1	0	0.502394	1.652732	-3.671382
55	8	0	2.234674	-2.390234	-1.788150

56	6	0	1.430648	-3.288799	-1.406884
57	8	0	0.441153	-3.088445	-0.651653
58	6	0	1.694561	-4.705037	-1.860620
59	1	0	0.750759	-5.225218	-2.023797
60	1	0	2.306646	-4.710677	-2.761804
61	1	0	2.235999	-5.218665	-1.060664
62	35	0	-0.338410	-1.342664	-3.368074

1A-TS4

Center Number	Atomic Number	Atomic Type	Coordinates (Angstroms) X	Y	Z
1	6	0	-0.198450	-1.526452	-0.975372
2	6	0	-1.105653	-0.694998	-1.691461
3	6	0	-1.296322	-0.856468	-3.042128
4	6	0	-0.548848	-1.824516	-3.743666
5	6	0	0.316427	-2.650237	-3.061241
6	6	0	0.497298	-2.558601	-1.659332
7	1	0	-1.625508	0.079391	-1.135369
8	1	0	-1.999241	-0.221763	-3.572617
9	1	0	0.849908	-3.433770	-3.594170
10	6	0	0.246732	-1.094131	0.346488
11	7	0	-0.436243	-0.645675	1.332060
12	6	0	-0.329920	0.148007	3.565805
13	1	0	-1.329135	0.481323	3.291954
14	1	0	-0.403922	-0.717435	4.236967
15	7	0	0.367242	-0.183172	2.338946
16	1	0	0.187738	0.959211	4.082869
17	1	0	-0.675703	-1.936434	-4.815967
18	6	0	1.623462	-0.767941	2.365599
19	6	0	2.041851	-1.384197	1.166036
20	6	0	2.388464	-0.855133	3.542458
21	6	0	3.177110	-2.216834	1.236231
22	6	0	3.540386	-1.619755	3.546900
23	1	0	2.066256	-0.348757	4.446056
24	6	0	3.925414	-2.325985	2.398811
25	1	0	3.508069	-2.740056	0.343243
26	1	0	4.129578	-1.693960	4.455448
27	1	0	4.817784	-2.943894	2.411828
28	45	0	1.830666	-0.482041	-0.718394

29	6	0	3.178808	1.187537	-0.224235
30	6	0	3.889930	0.437158	-1.230520
31	6	0	2.063506	1.877019	-0.854882
32	6	0	3.095715	0.487105	-2.391597
33	6	0	1.989696	1.421577	-2.171818
34	6	0	3.683263	1.467038	1.158934
35	1	0	4.418102	2.280552	1.137251
36	1	0	2.866247	1.765735	1.821139
37	1	0	4.160441	0.585225	1.596648
38	6	0	5.188281	-0.280131	-1.020897
39	1	0	6.039727	0.405583	-1.104451
40	1	0	5.228370	-0.738179	-0.027607
41	1	0	5.328495	-1.073830	-1.759579
42	6	0	3.349565	-0.192568	-3.702897
43	1	0	3.727580	0.517866	-4.447232
44	1	0	4.082873	-0.995460	-3.599502
45	1	0	2.426414	-0.627858	-4.100790
46	6	0	0.988449	1.785367	-3.223421
47	1	0	1.415884	2.480434	-3.955398
48	1	0	0.651257	0.896704	-3.766371
49	1	0	0.103015	2.255348	-2.788251
50	6	0	1.143278	2.822118	-0.143445
51	1	0	0.776353	2.381911	0.789337
52	1	0	1.651433	3.760608	0.105976
53	1	0	0.274919	3.066783	-0.759217
54	6	0	1.181450	-3.713865	-0.964765
55	1	0	2.206570	-3.846459	-1.323434
56	1	0	1.205159	-3.583935	0.115752
57	1	0	0.633821	-4.636504	-1.186002

1B-TS4

Center Number	Atomic Number	Atomic Type	Coordinates (Angstroms) X	Y	Z
1	6	0	-0.221115	-1.521679	-0.994161
2	6	0	-1.142680	-0.693073	-1.695051
3	6	0	-1.341168	-0.831217	-3.047627
4	6	0	-0.595371	-1.778052	-3.778513
5	6	0	0.284654	-2.614926	-3.128109
6	6	0	0.466866	-2.511664	-1.734210

7	1	0	-1.669749	0.060608	-1.118138
8	1	0	-2.056943	-0.194292	-3.557338
9	1	0	0.822188	-3.386911	-3.667182
10	6	0	0.230568	-1.124212	0.333232
11	7	0	-0.461194	-0.698085	1.319995
12	6	0	-0.384612	0.057944	3.565127
13	1	0	-1.391102	0.368857	3.291712
14	1	0	-0.438318	-0.818730	4.223165
15	7	0	0.325940	-0.237892	2.336350
16	1	0	0.110091	0.874865	4.095314
17	1	0	-0.732818	-1.871222	-4.850833
18	6	0	1.597321	-0.795035	2.361082
19	6	0	2.041744	-1.389440	1.162166
20	6	0	2.354178	-0.873995	3.544530
21	6	0	3.202242	-2.185305	1.230758
22	6	0	3.527868	-1.603717	3.551690
23	1	0	2.008251	-0.388966	4.450871
24	6	0	3.943781	-2.282506	2.398212
25	1	0	3.555510	-2.687330	0.335993
26	1	0	4.110791	-1.671059	4.464795
27	1	0	4.855236	-2.872046	2.410417
28	45	0	1.816819	-0.507945	-0.733685
29	6	0	3.198914	1.140020	-0.249743
30	6	0	3.880142	0.371994	-1.260224
31	6	0	2.086928	1.849559	-0.869992
32	6	0	3.068106	0.433002	-2.411296
33	6	0	1.990797	1.398813	-2.186246
34	6	0	3.717960	1.414127	1.128901
35	1	0	4.473371	2.208092	1.096241
36	1	0	2.913270	1.739471	1.793277
37	1	0	4.173657	0.523339	1.570699
38	6	0	5.157476	-0.387658	-1.070485
39	1	0	6.031311	0.236901	-1.289263
40	1	0	5.249718	-0.741661	-0.039523
41	1	0	5.201224	-1.263307	-1.724410
42	6	0	3.290913	-0.272073	-3.714849
43	1	0	3.709307	0.409271	-4.464546
44	1	0	3.979625	-1.112004	-3.598117
45	1	0	2.348174	-0.662883	-4.112878
46	6	0	0.987132	1.787301	-3.226665
47	1	0	1.417814	2.486888	-3.952319
48	1	0	0.636963	0.910301	-3.780099
49	1	0	0.109977	2.262070	-2.779941
50	6	0	1.200176	2.819649	-0.149970

51	1	0	0.826106	2.388605	0.784121
52	1	0	1.739448	3.740704	0.098995
53	1	0	0.336378	3.093803	-0.759636
54	35	0	1.340685	-3.971732	-0.891828

8. Copies of NMR spectra

NMR spectra of **1a**

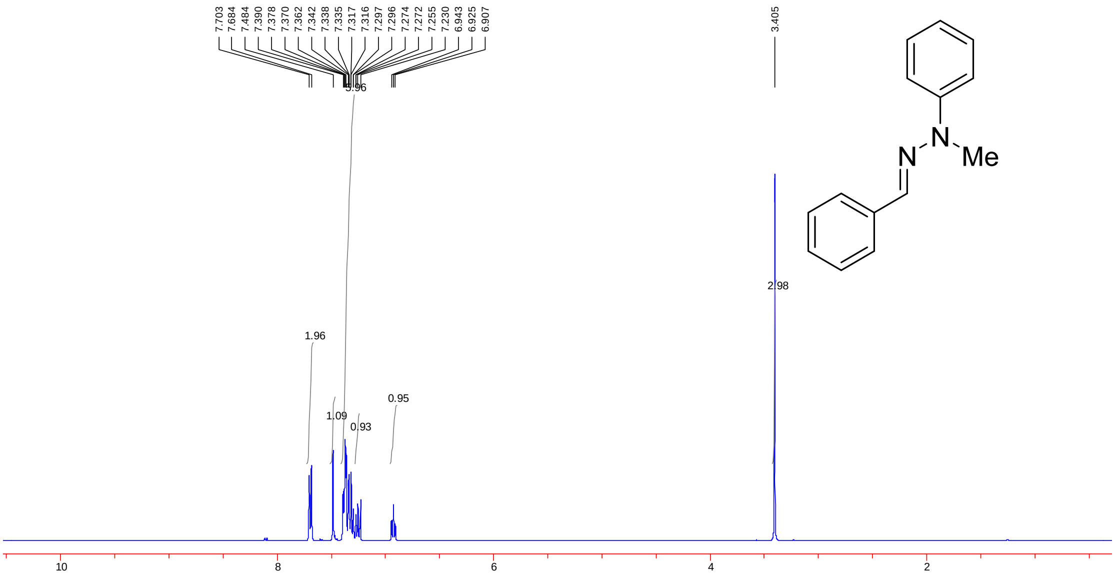

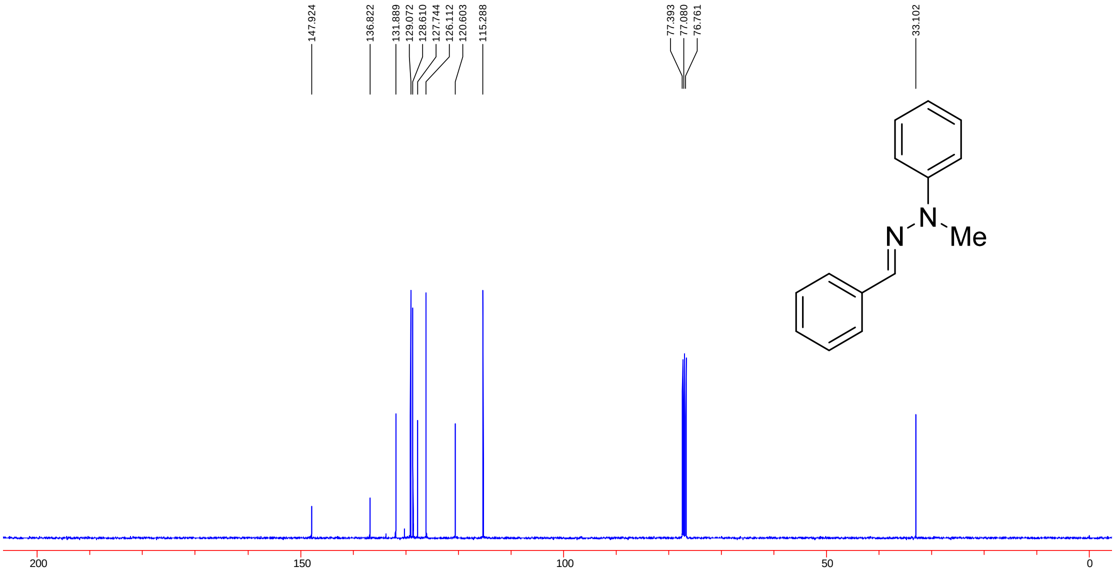

NMR spectra of **1b**

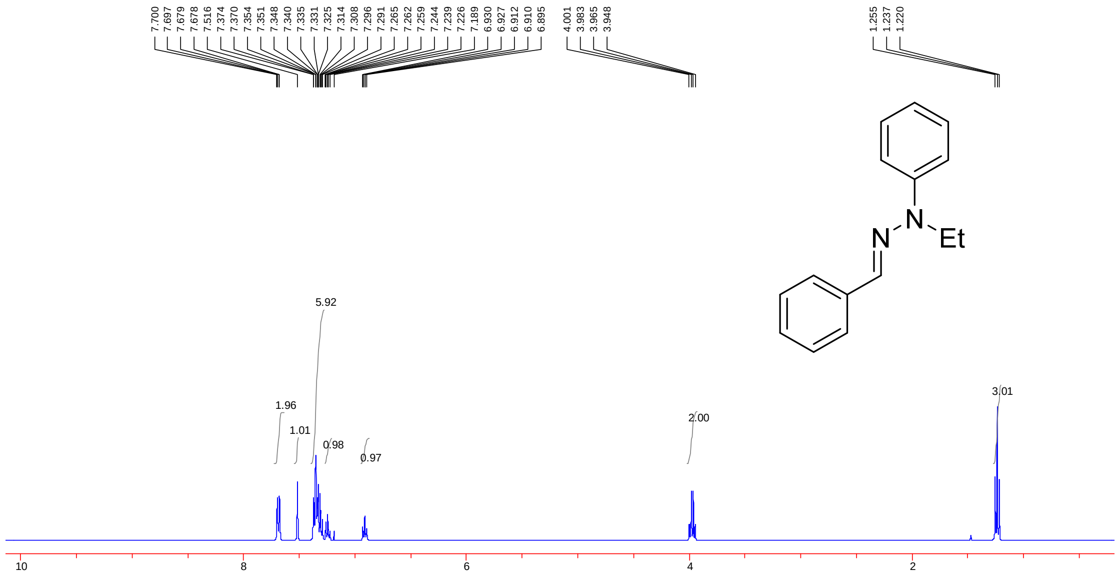

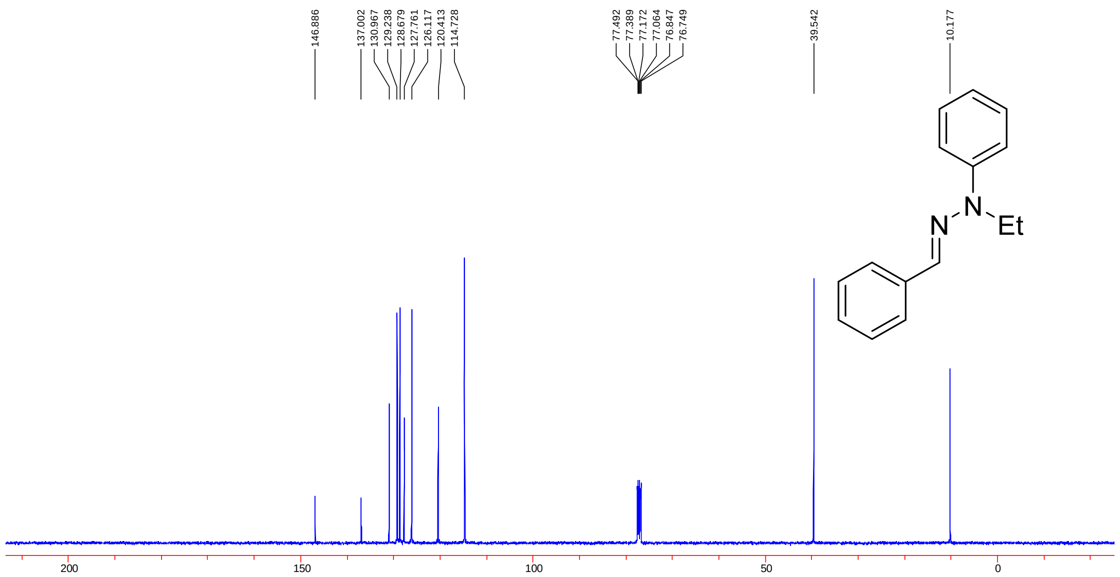

NMR spectra of **1c**

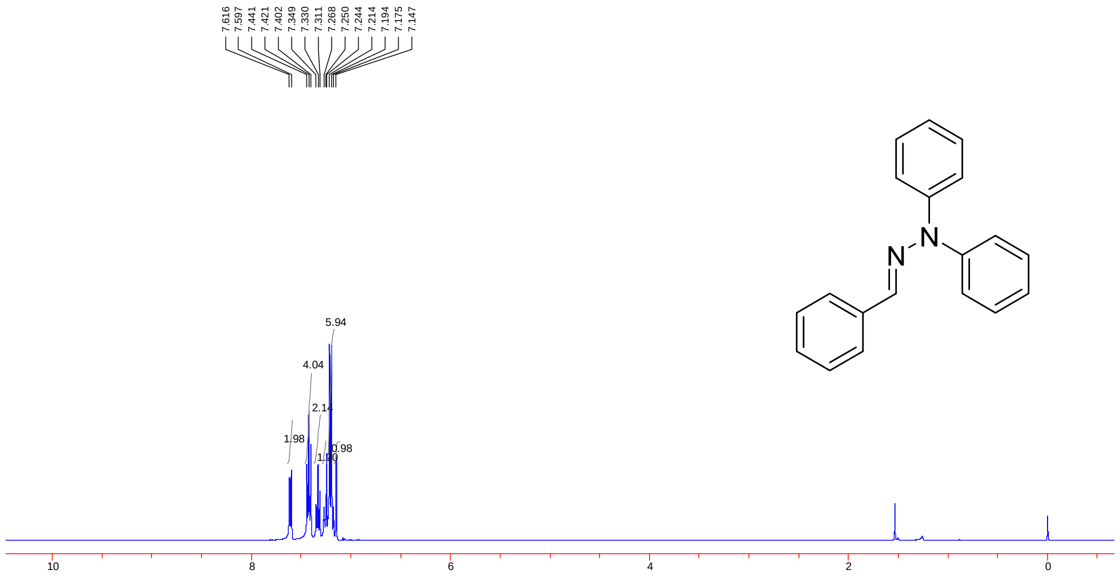

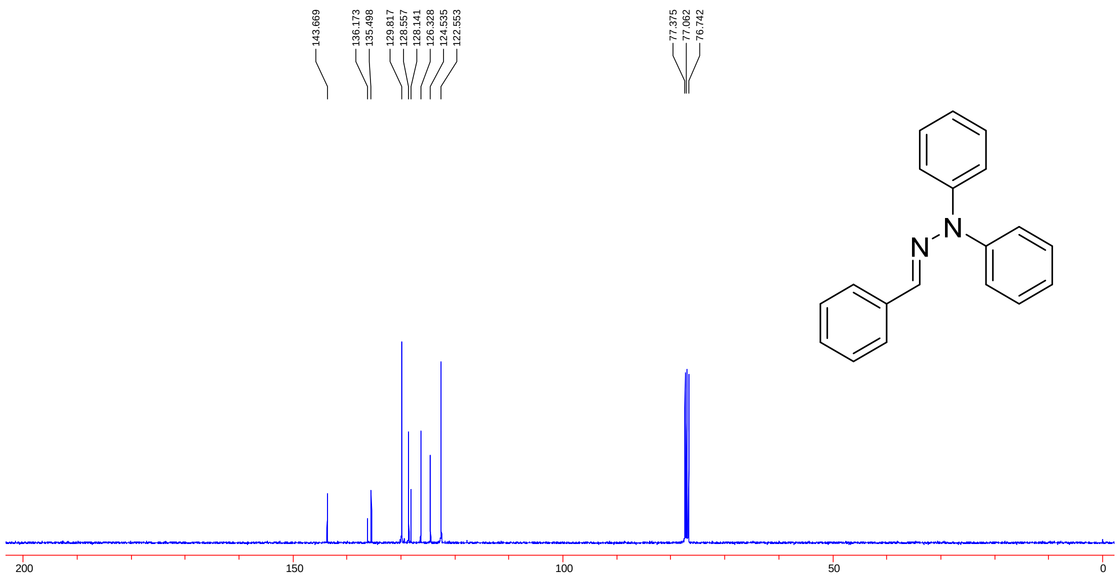

NMR spectra of **1d**

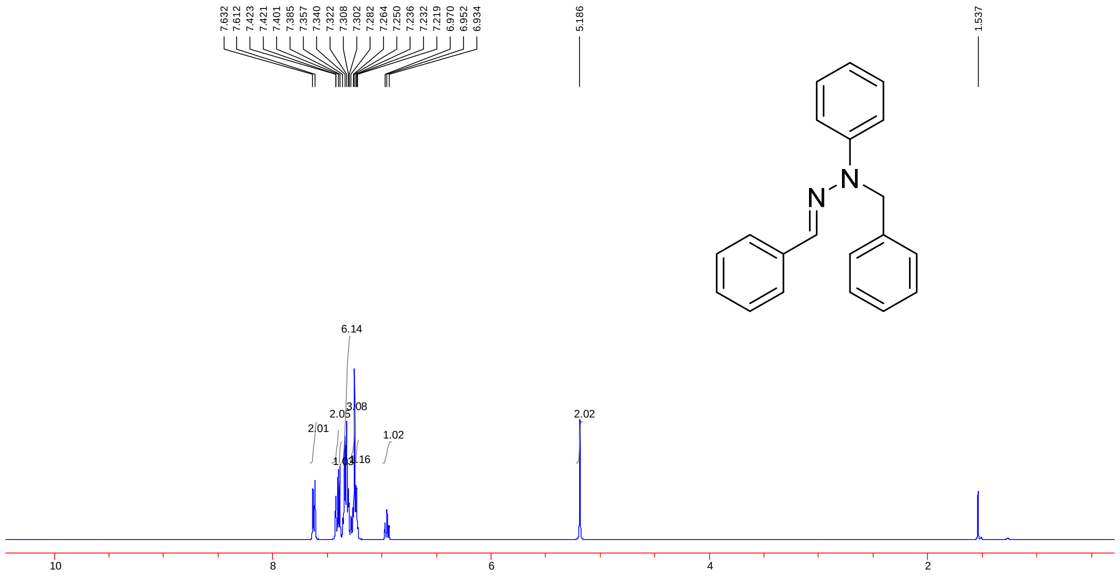

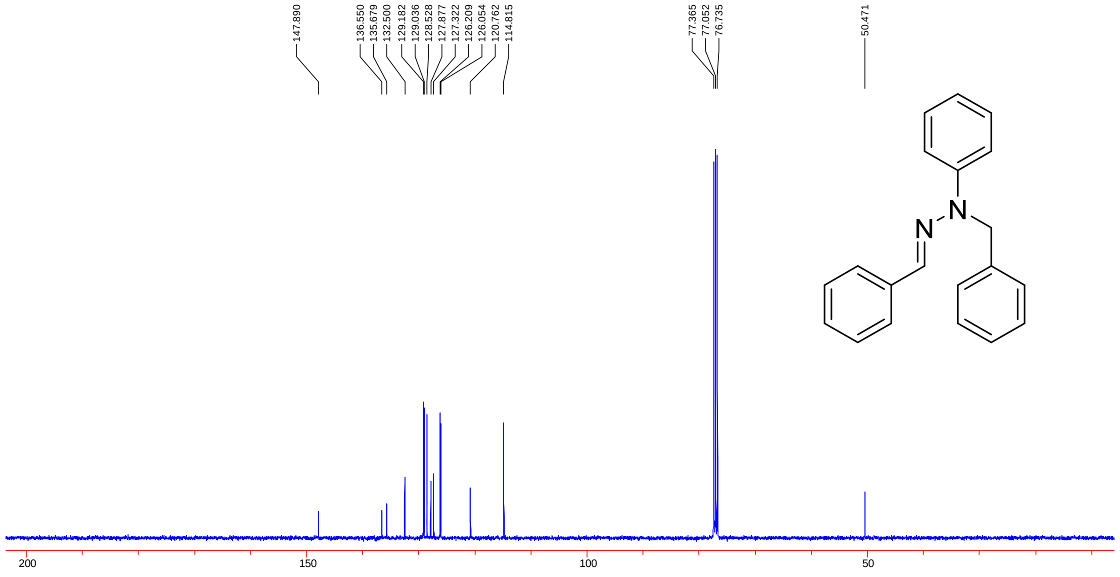

NMR spectra of **1e**

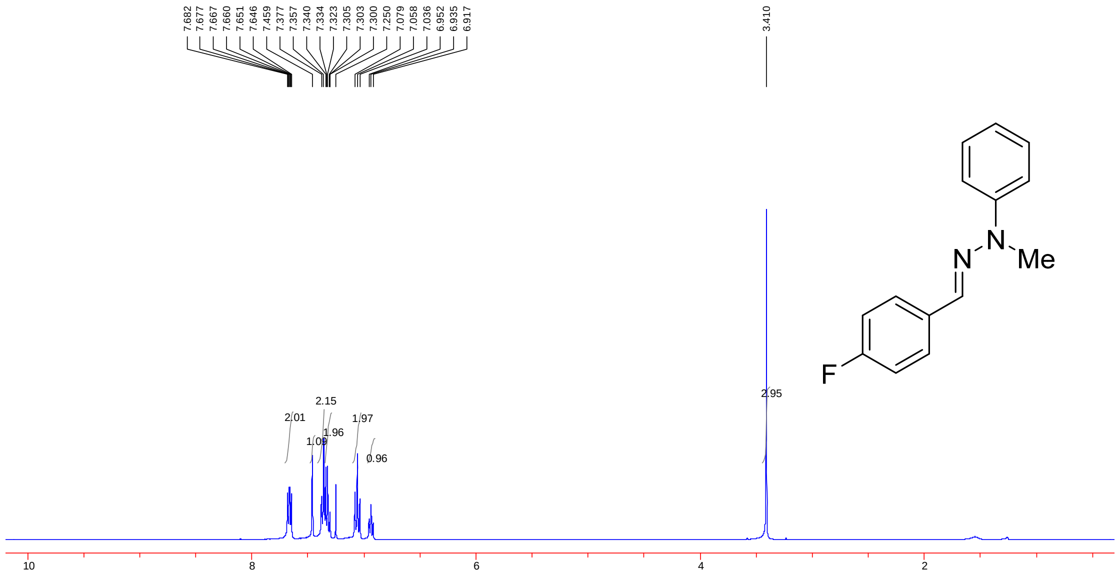

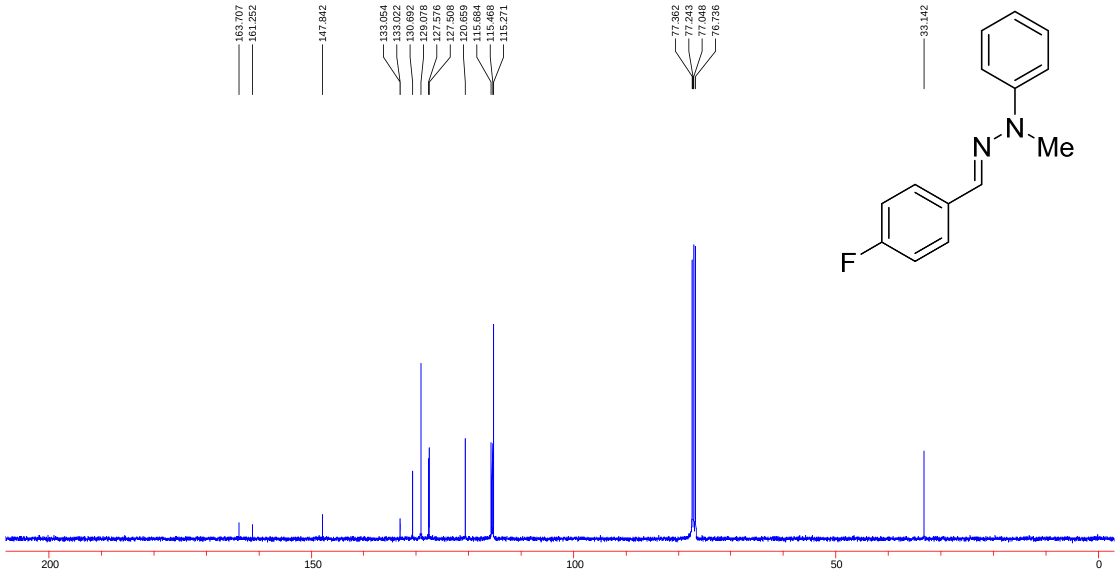

NMR spectra of **1f**

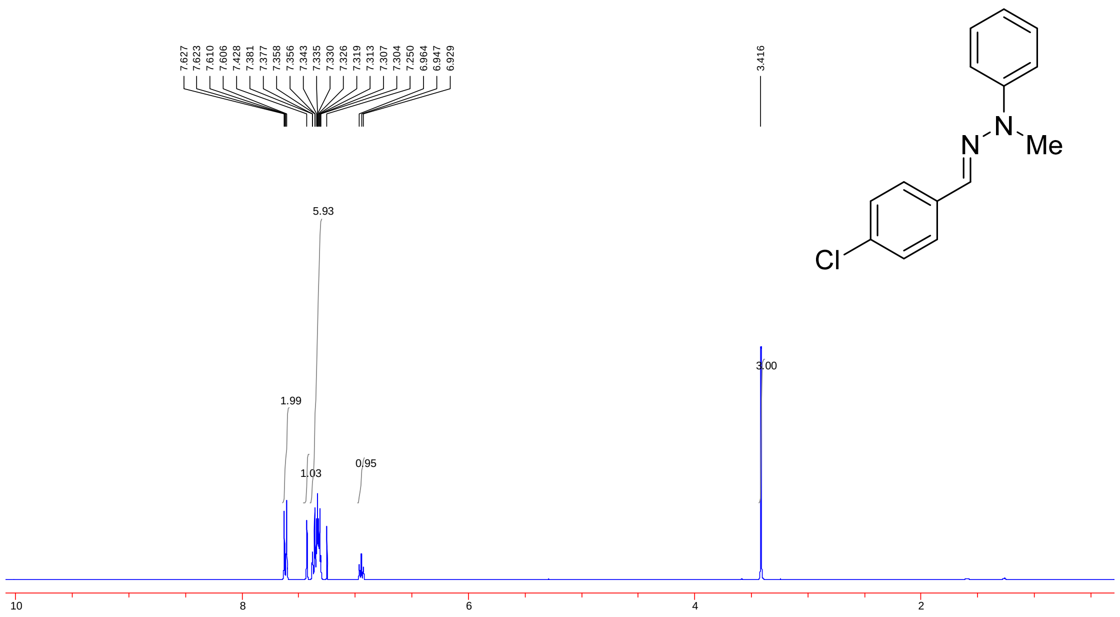

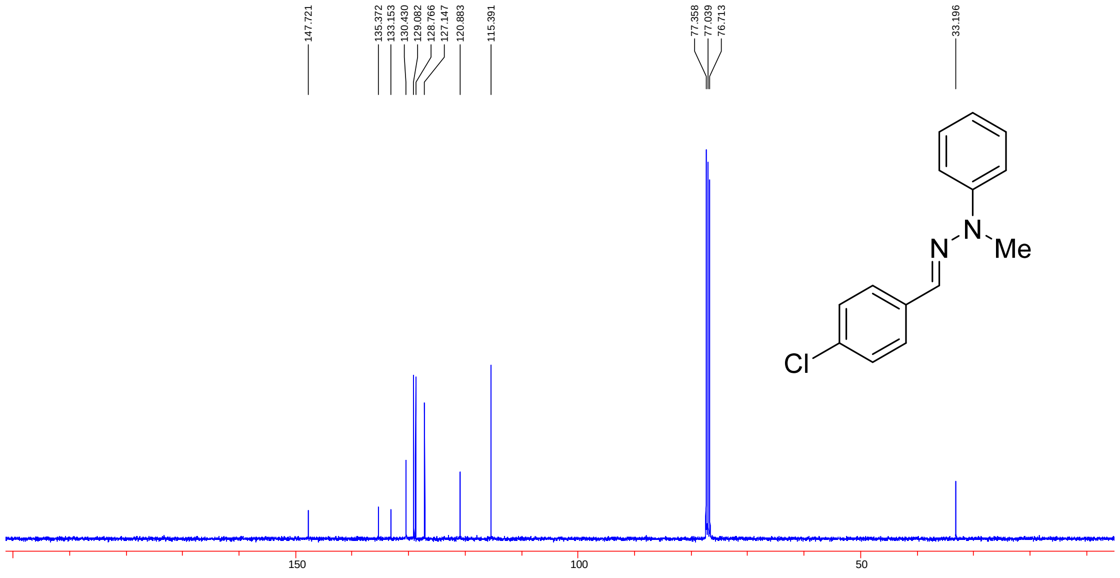

NMR spectra of **1g**

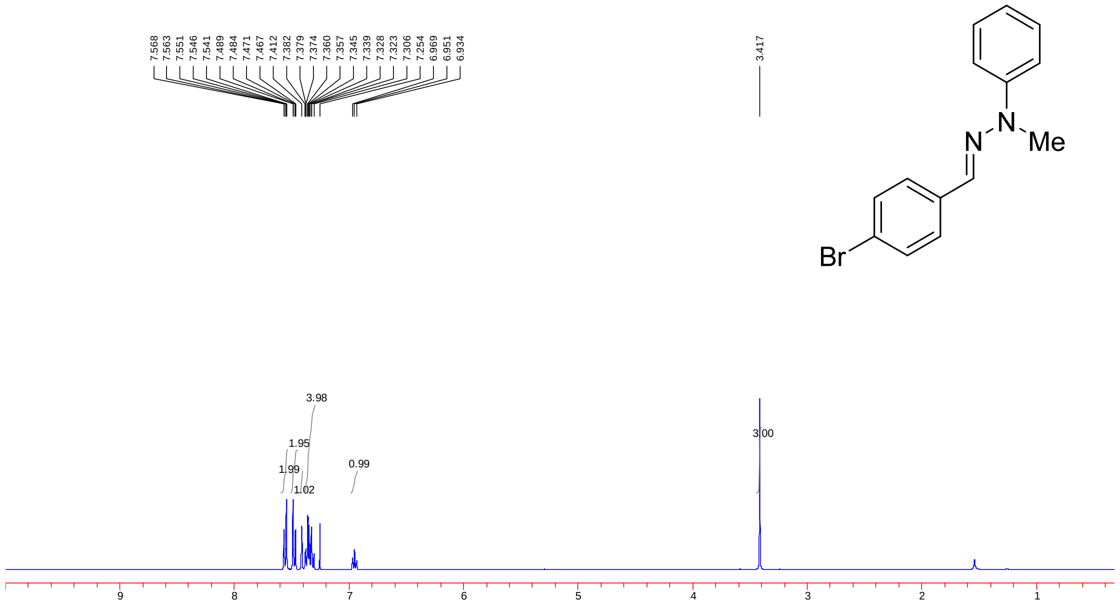

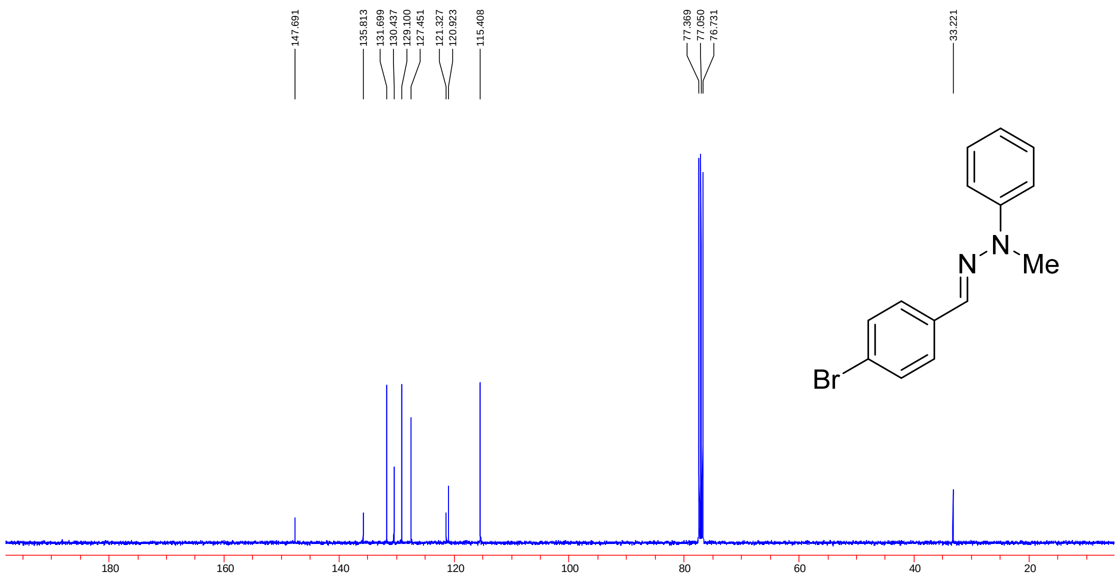

NMR spectra of **1h**

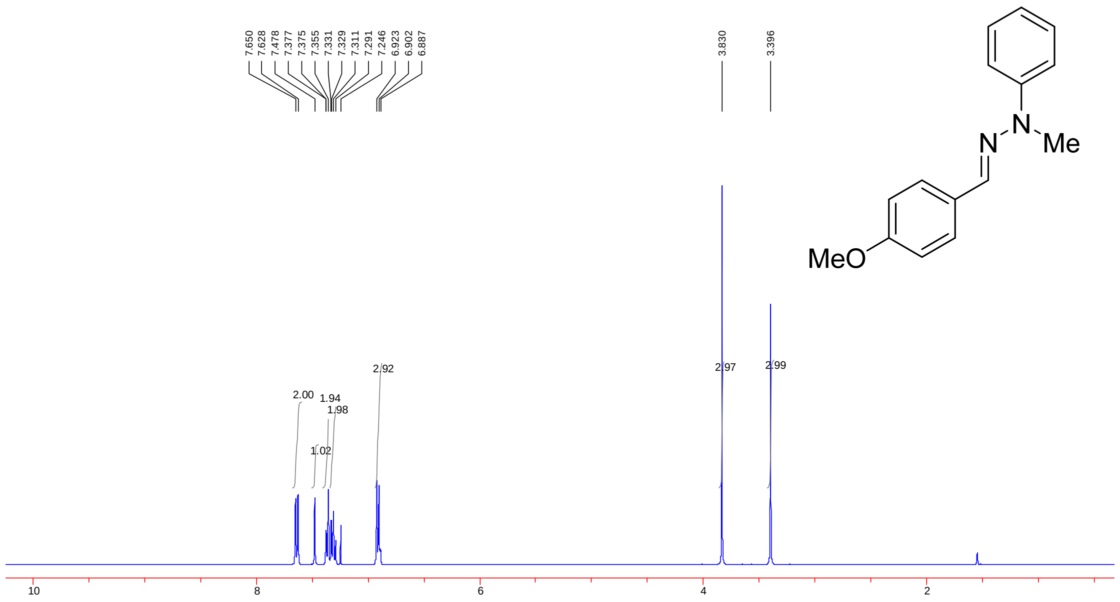

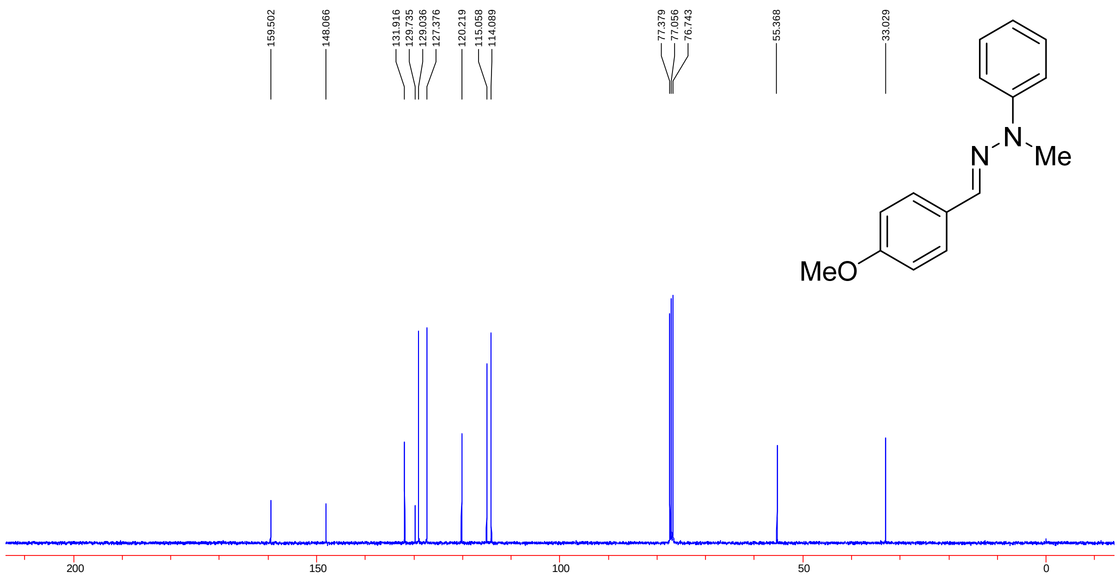

NMR spectra of **1i**

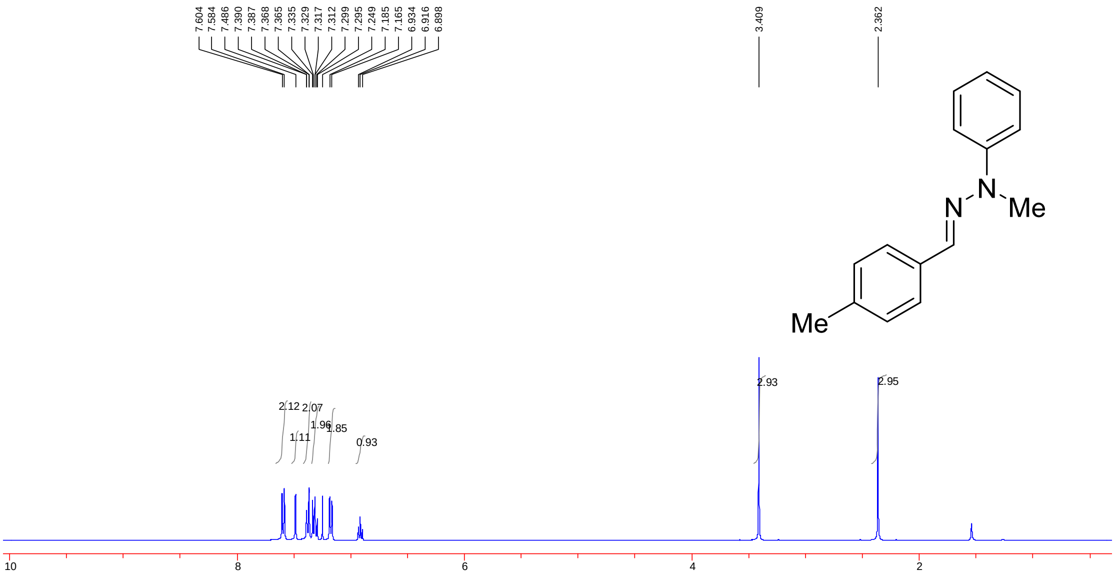

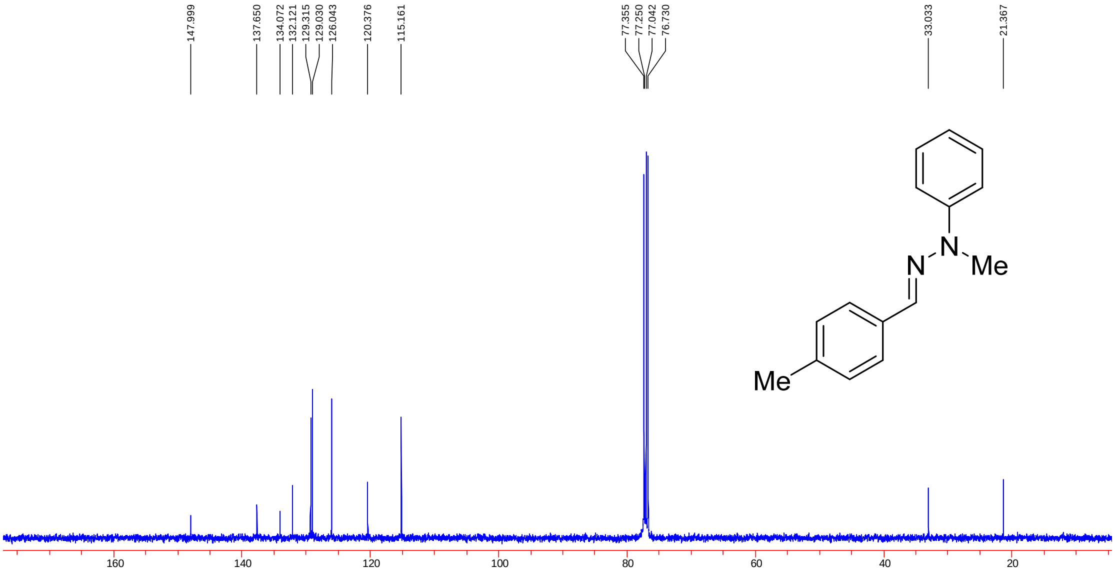

NMR spectra of **1j**

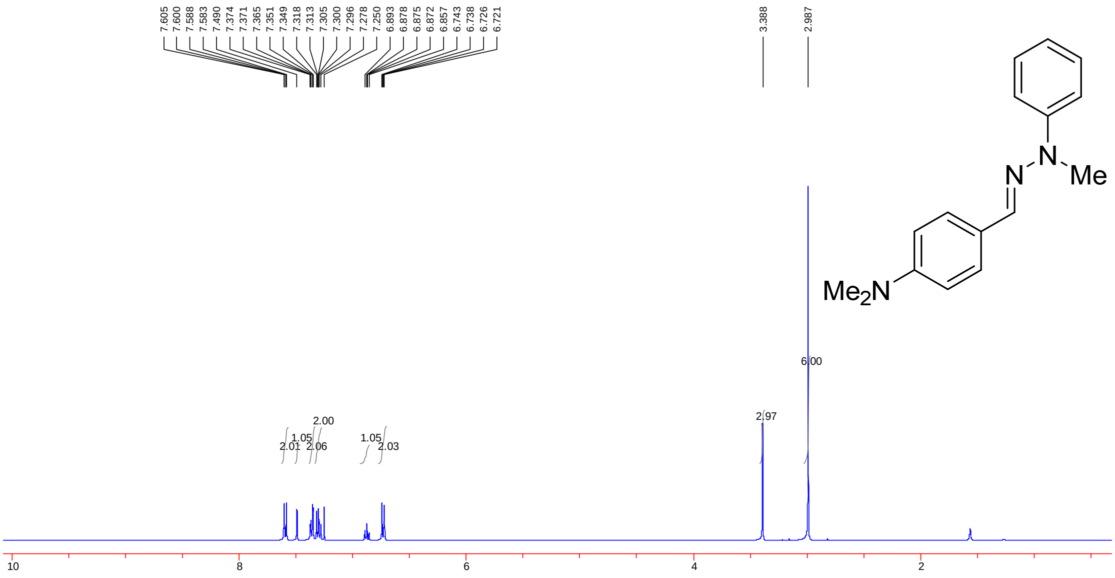

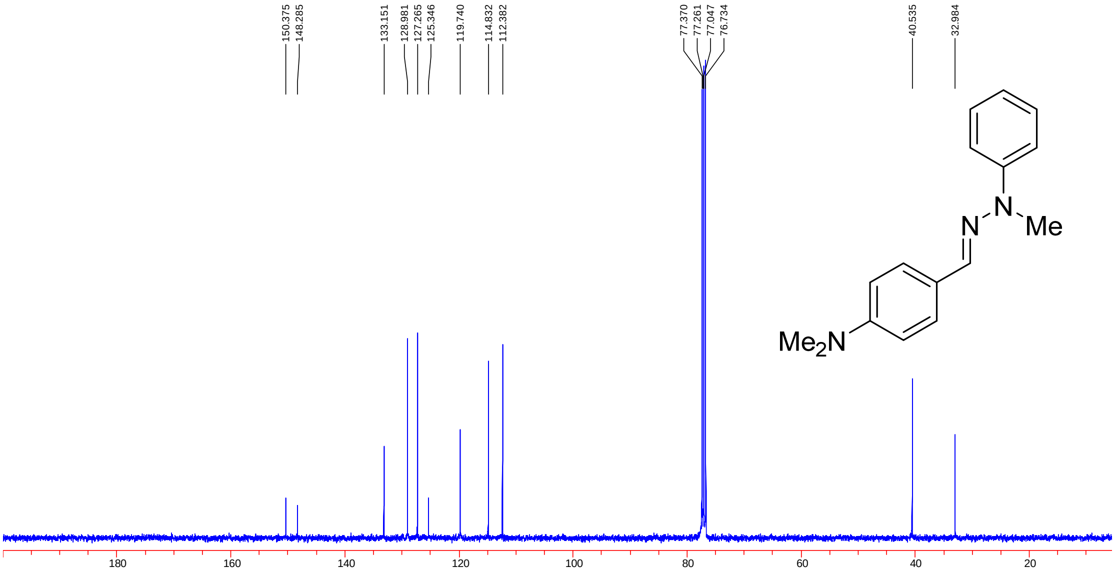

NMR spectra of **1k**

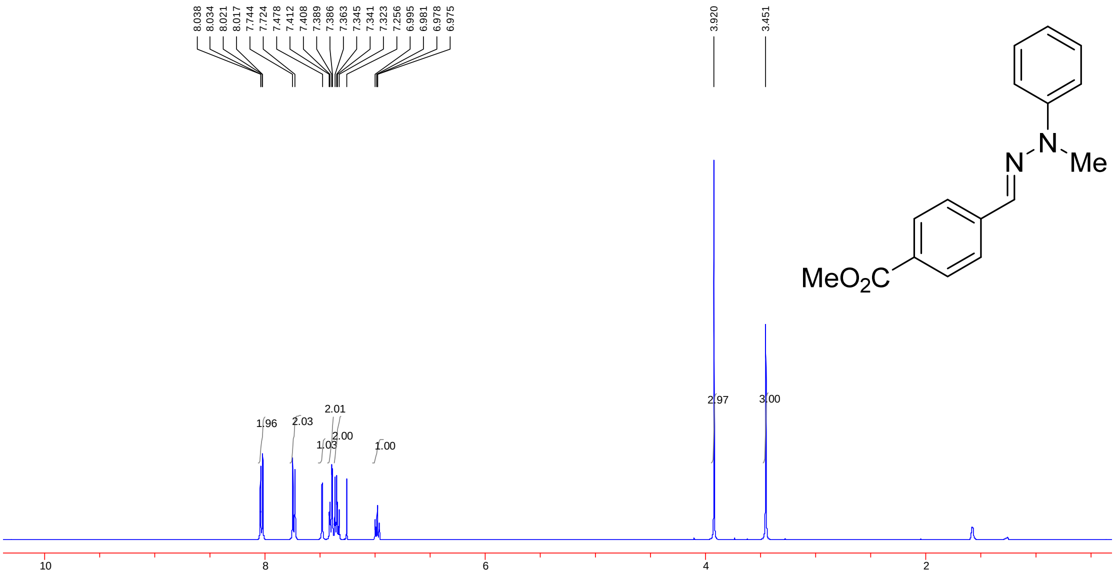

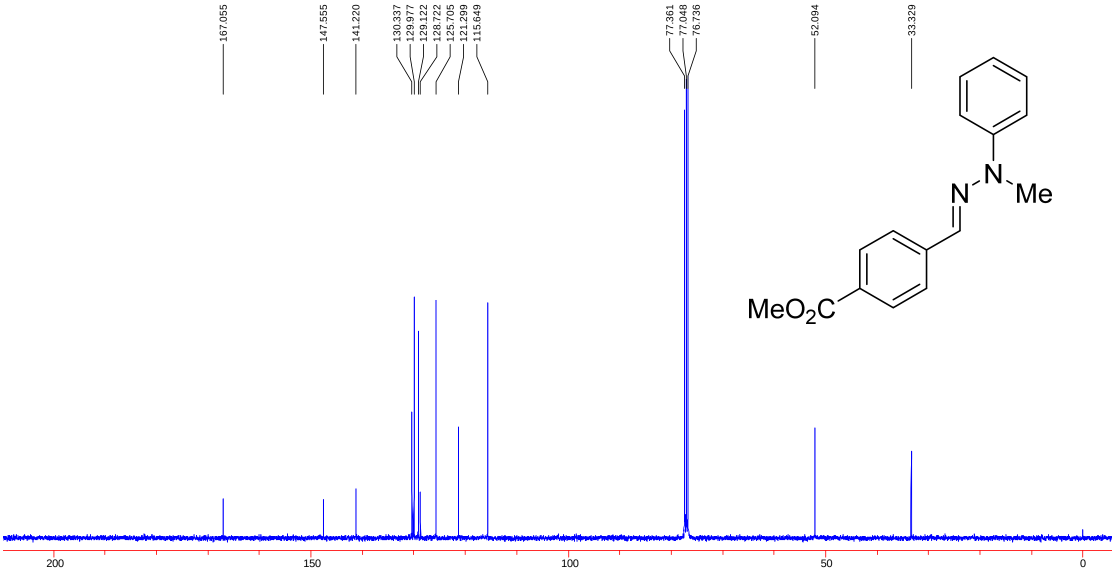

NMR spectra of **1l**

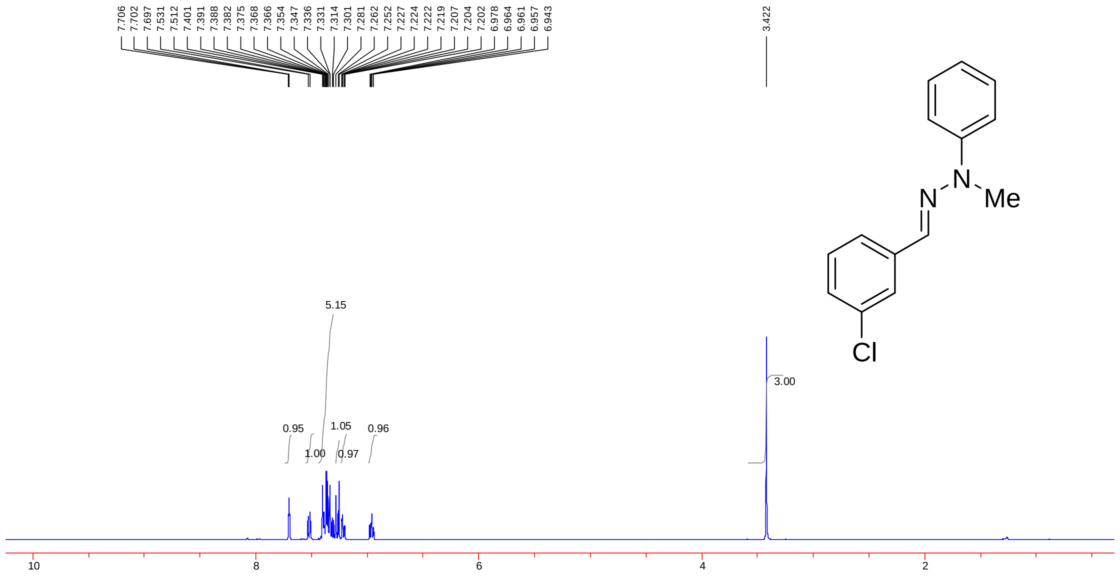

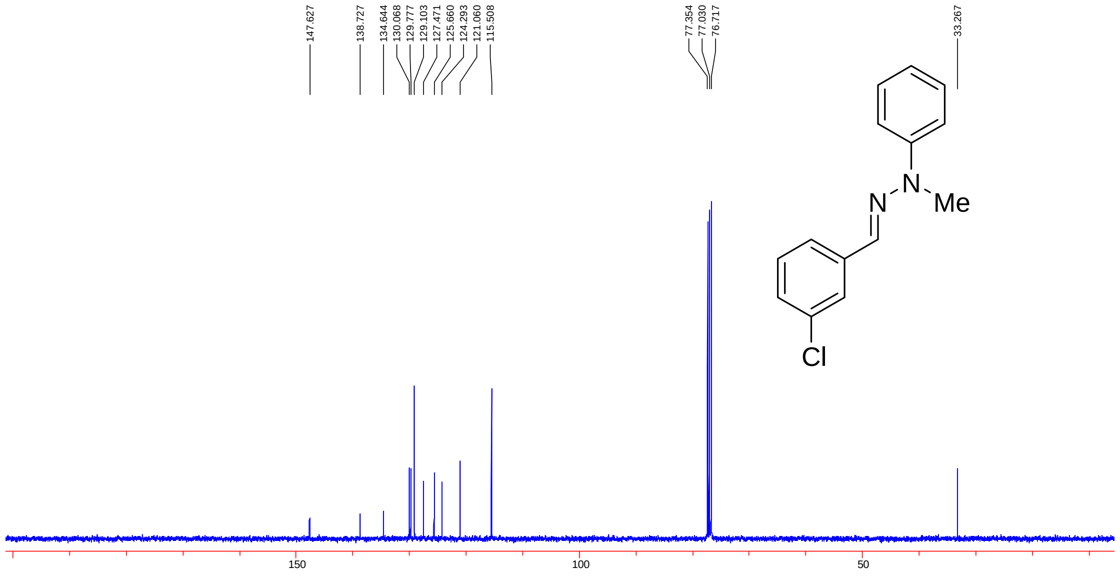

NMR spectra of **1m**

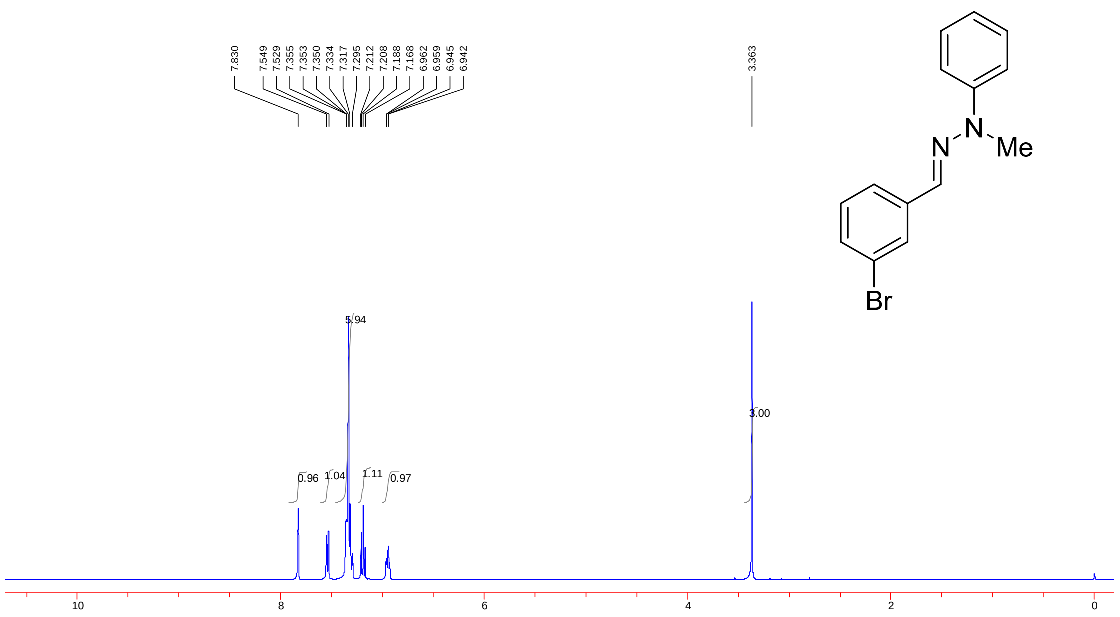

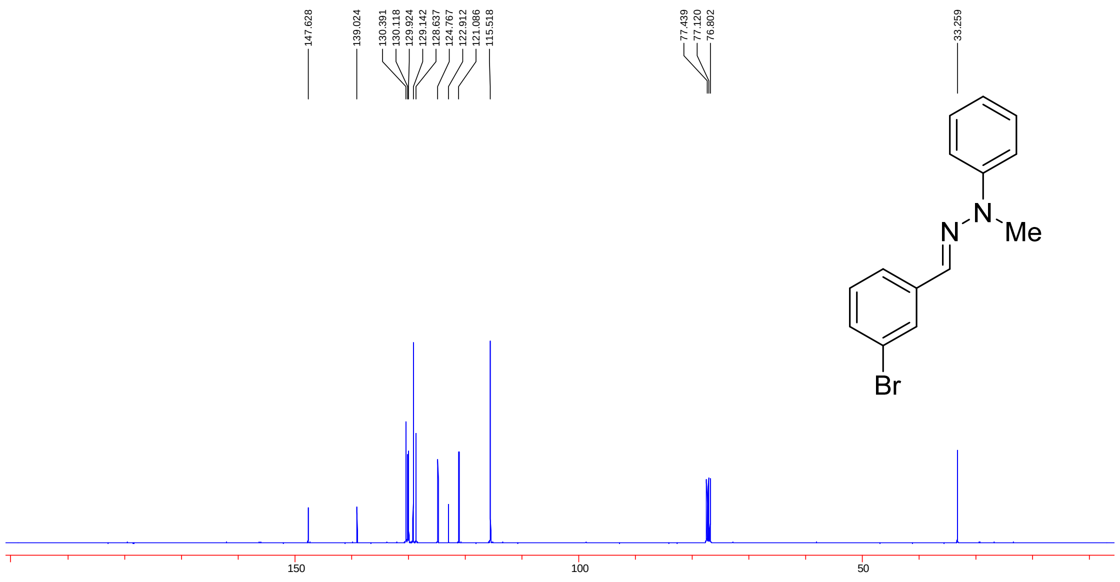

NMR spectra of **1n**

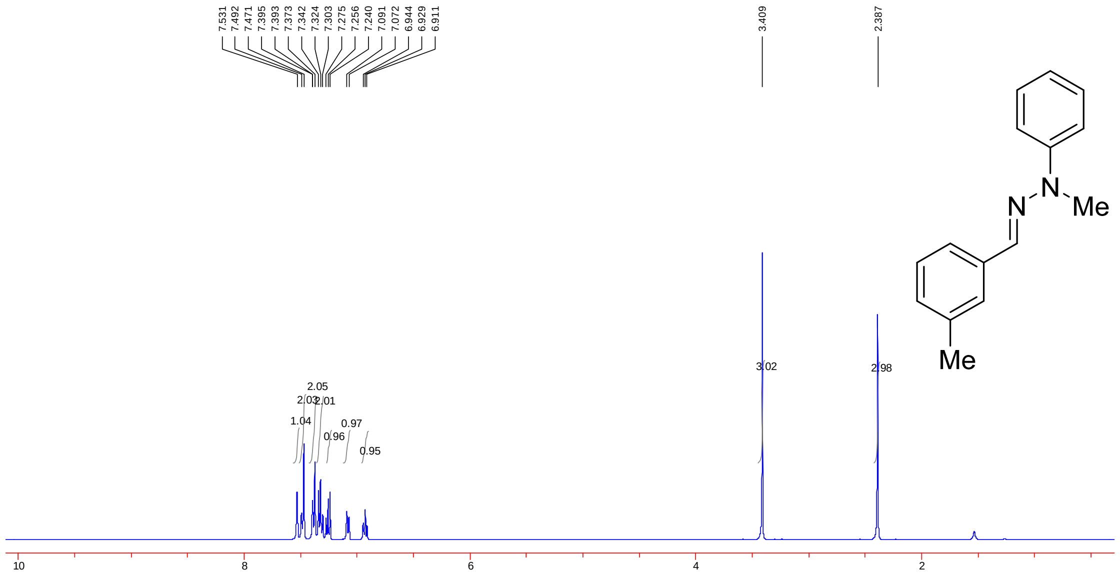

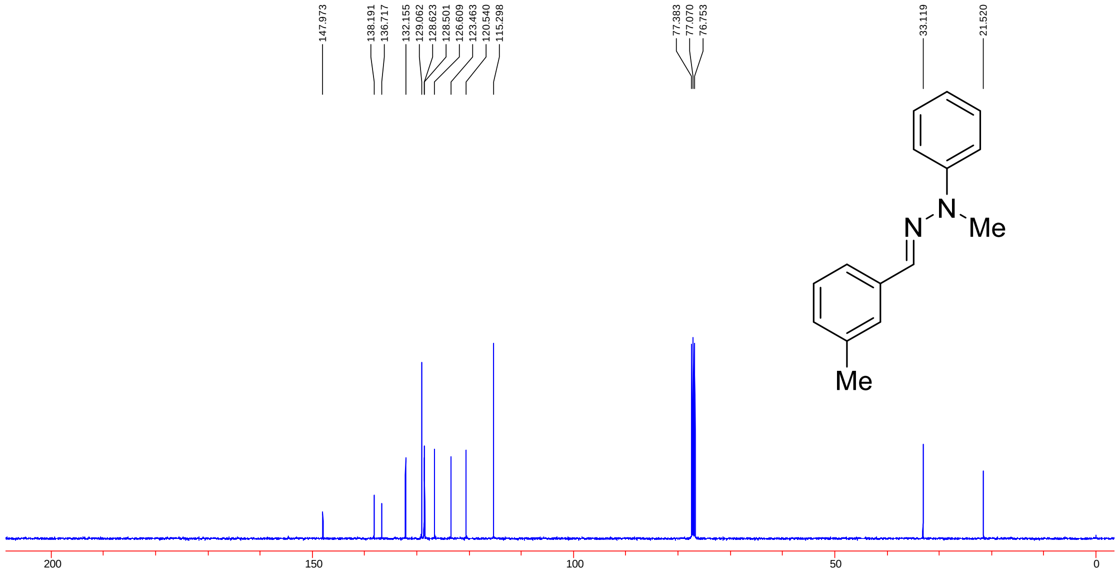

NMR spectra of **1o**

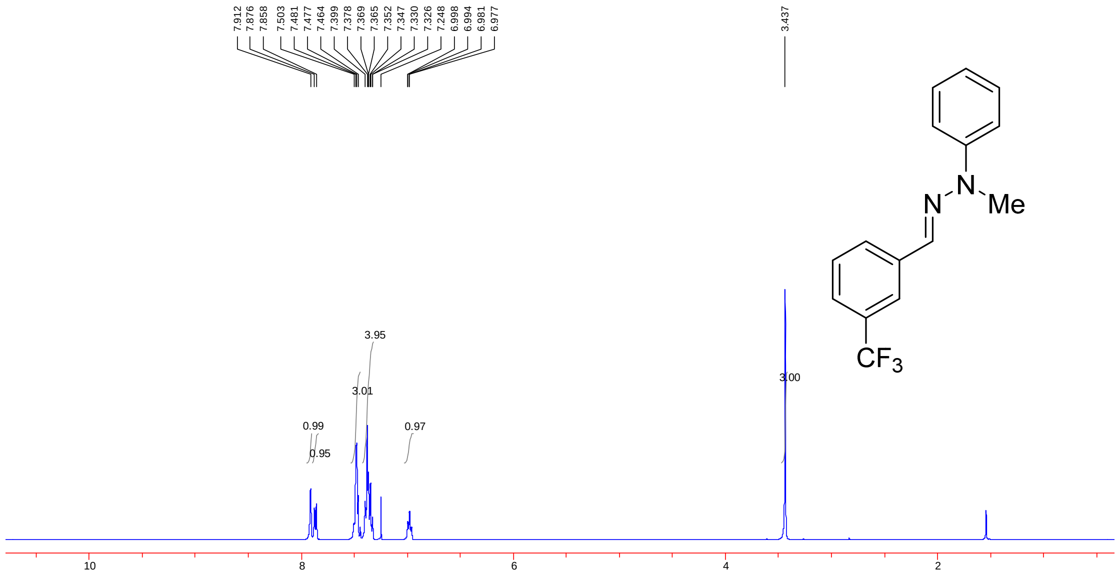

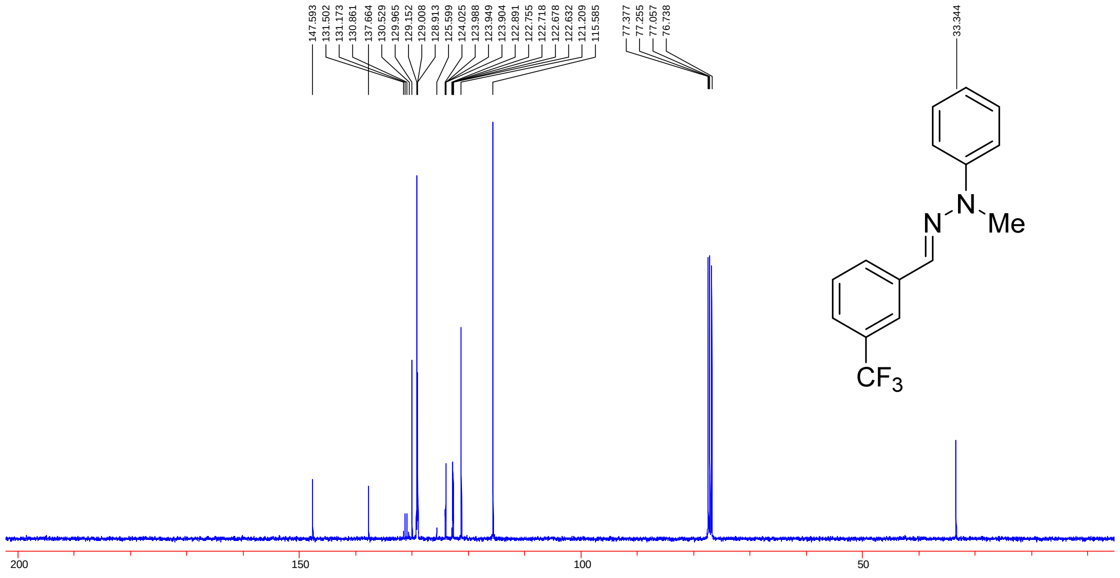

NMR spectra of **1p**

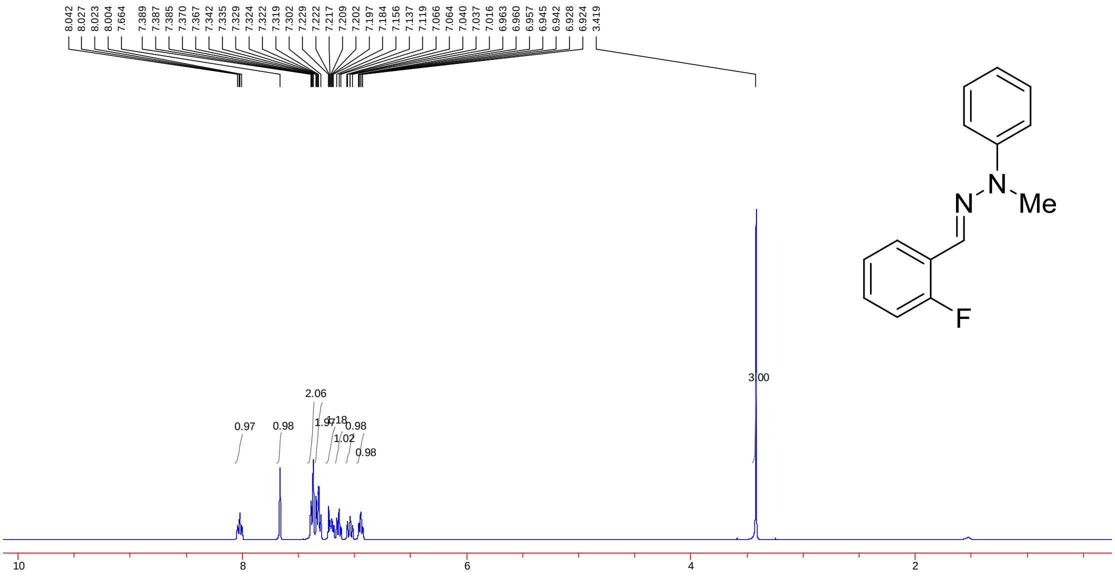

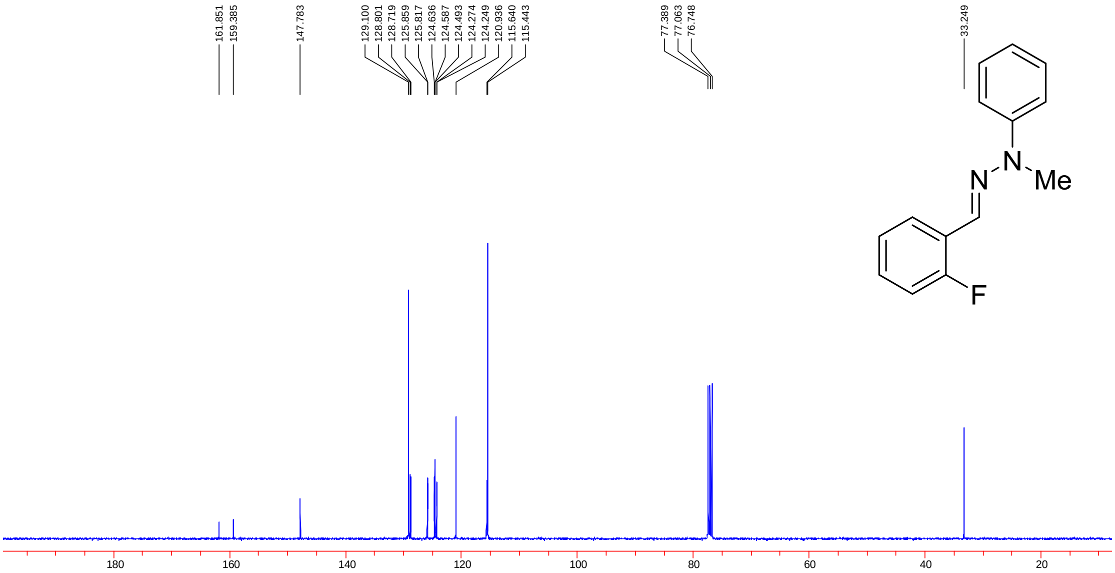

NMR spectra of **1q**

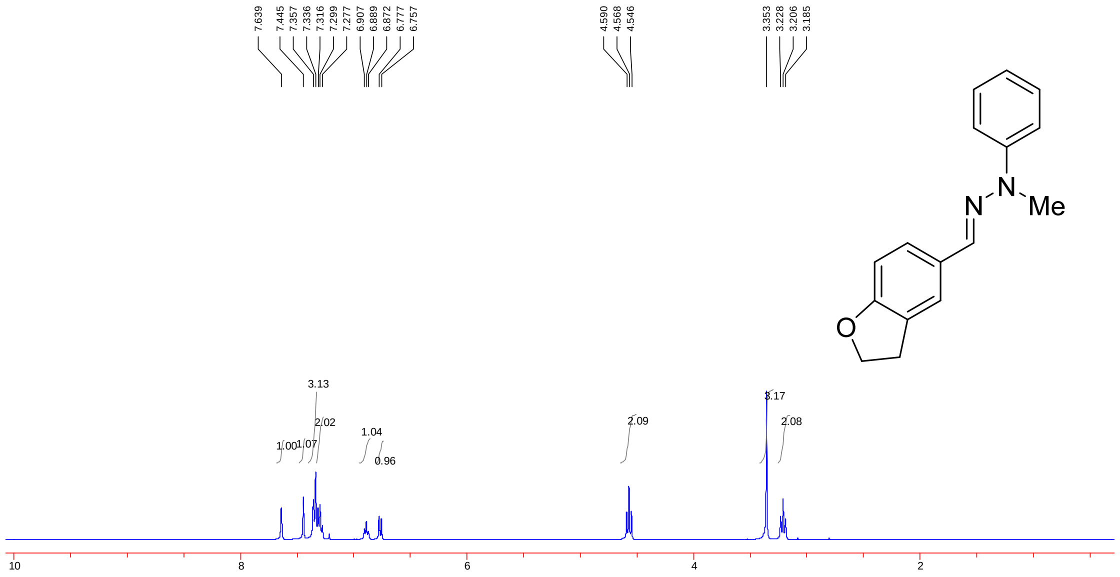

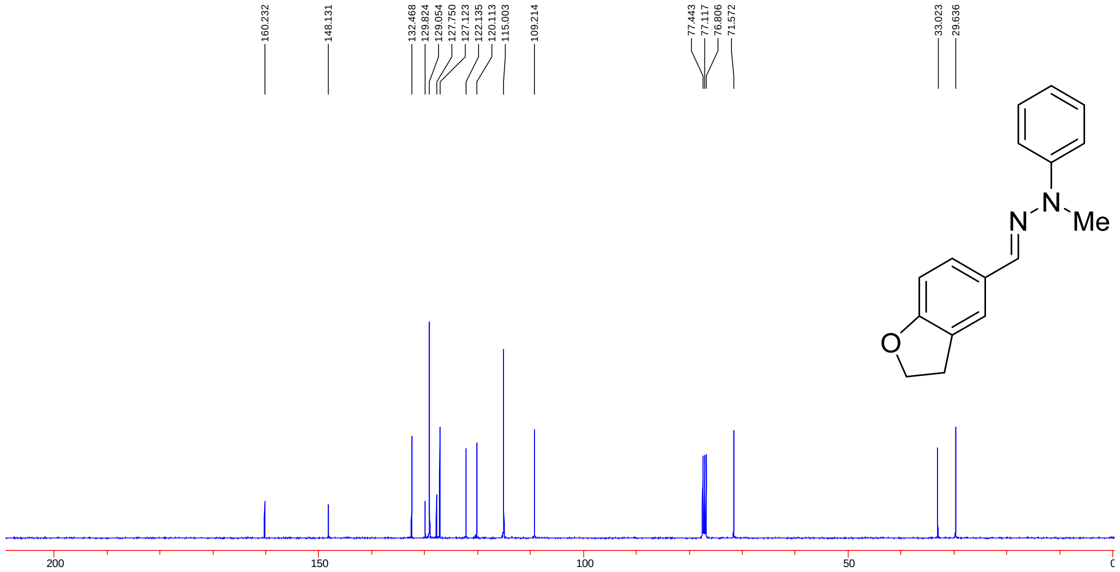

NMR spectra of **1r**

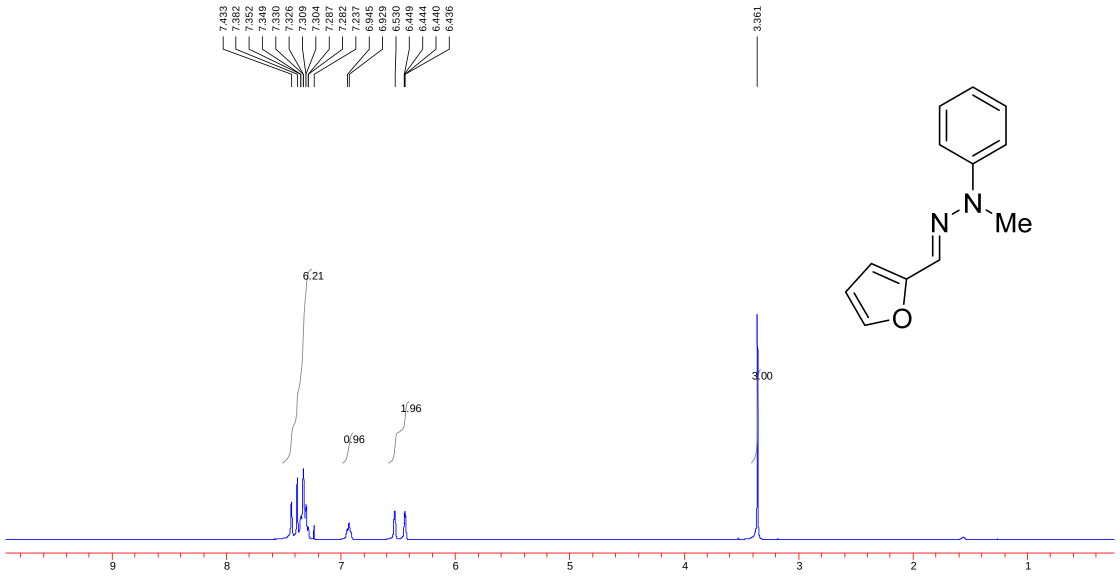

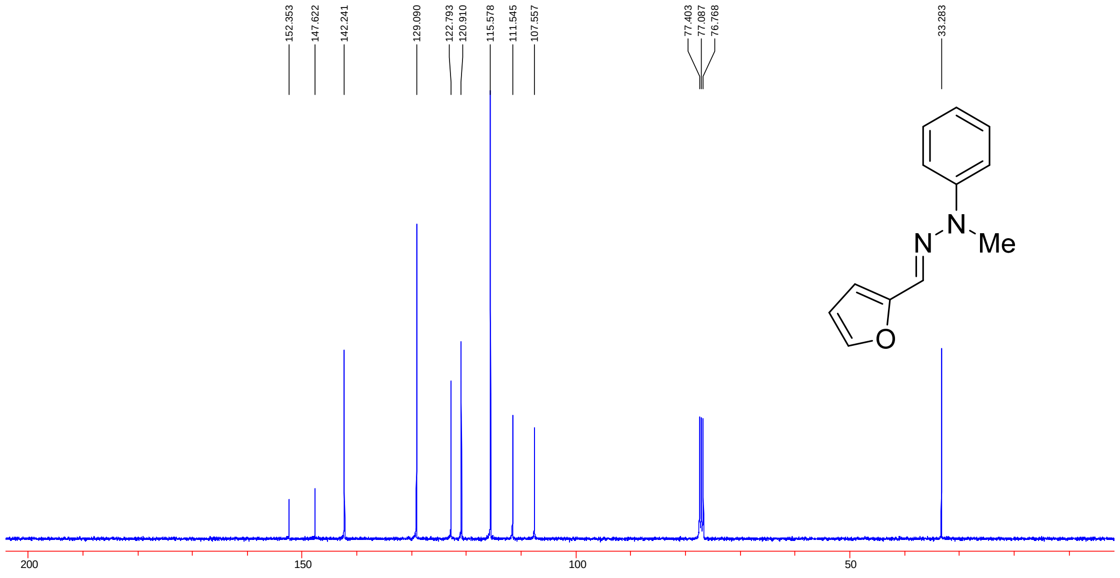

NMR spectra of **1s**

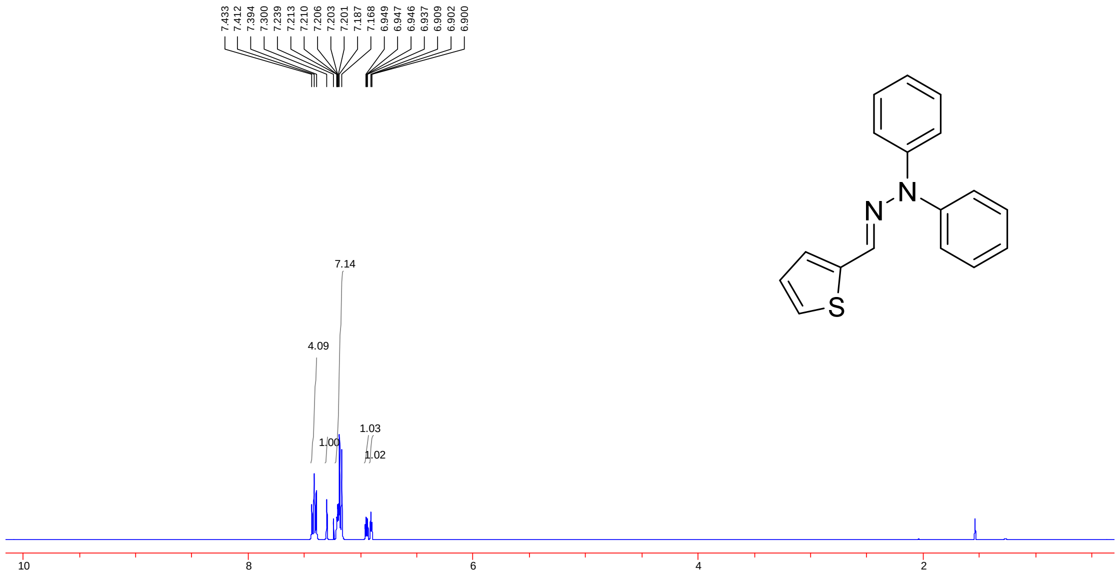

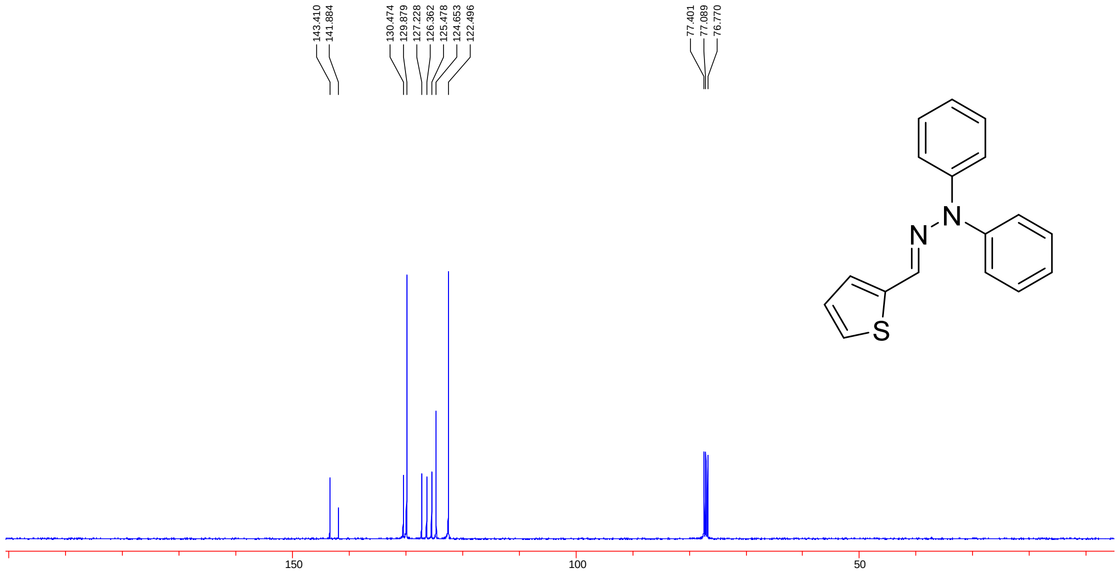

NMR spectra of **1t**

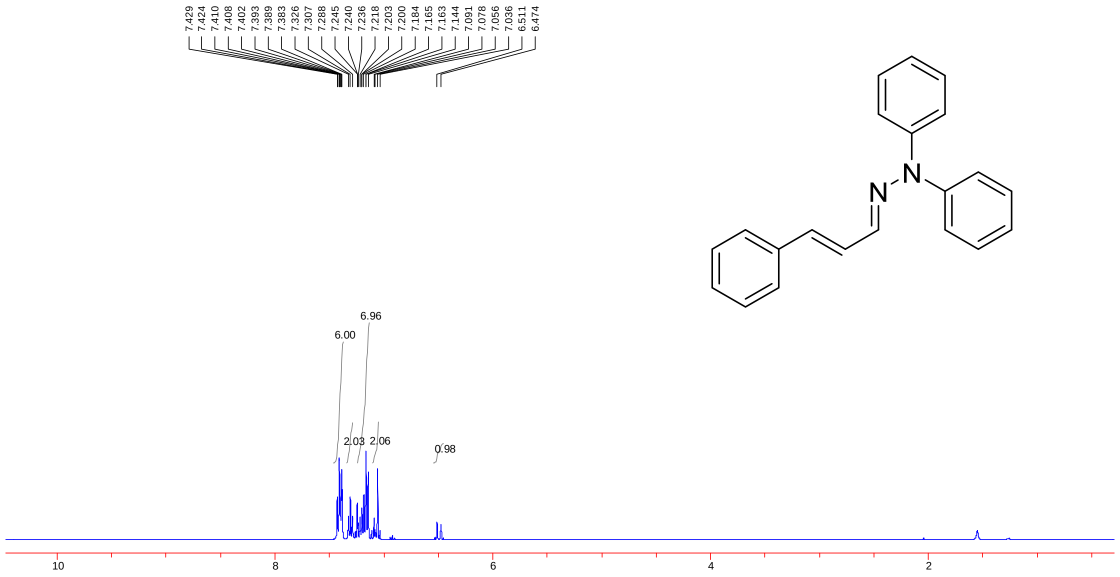

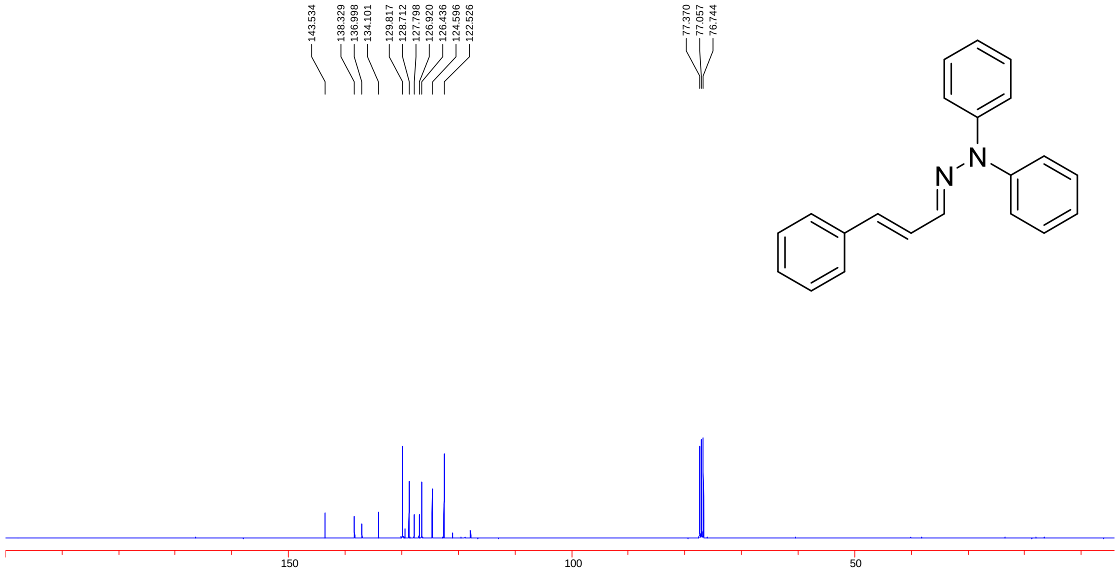

NMR spectra of **1u**

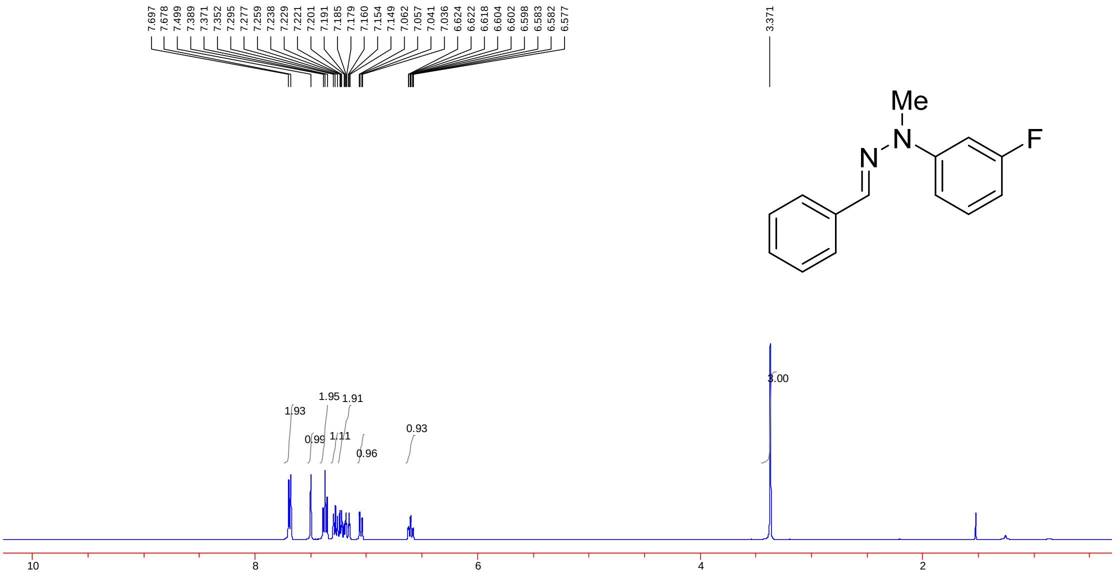

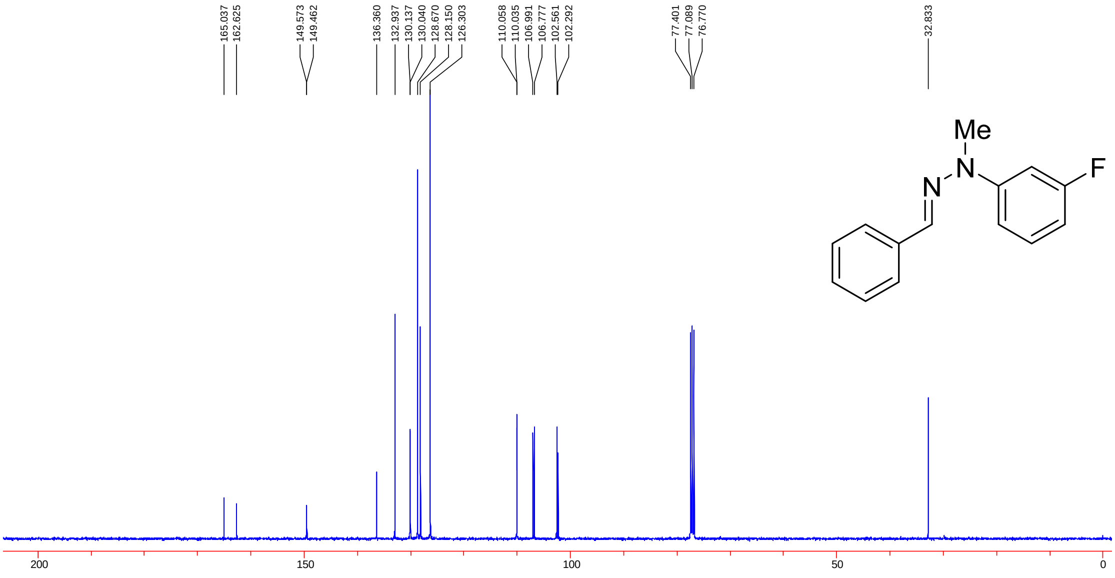

NMR spectra of **1v**

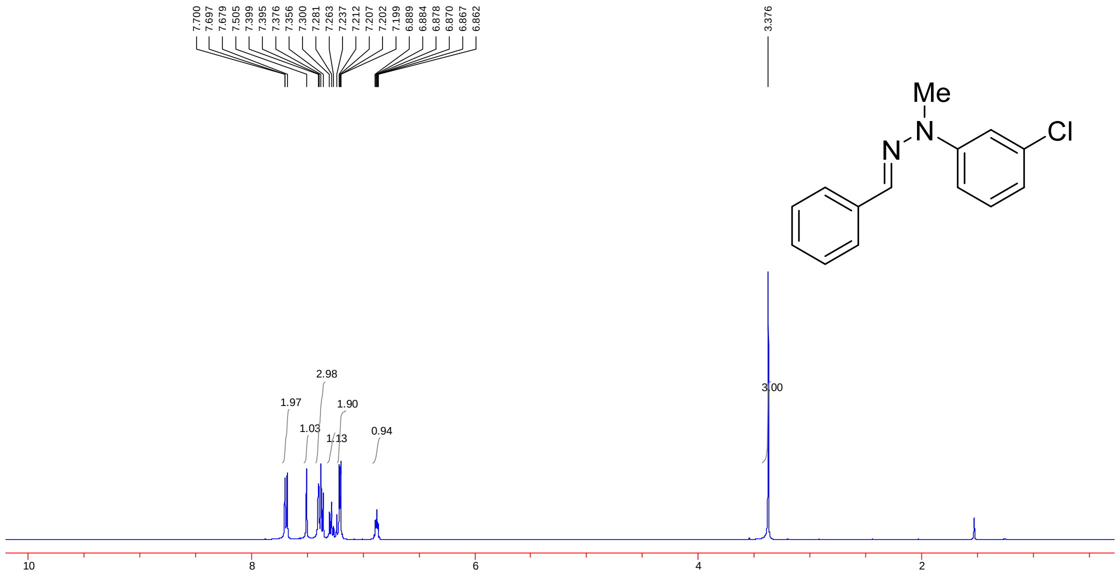

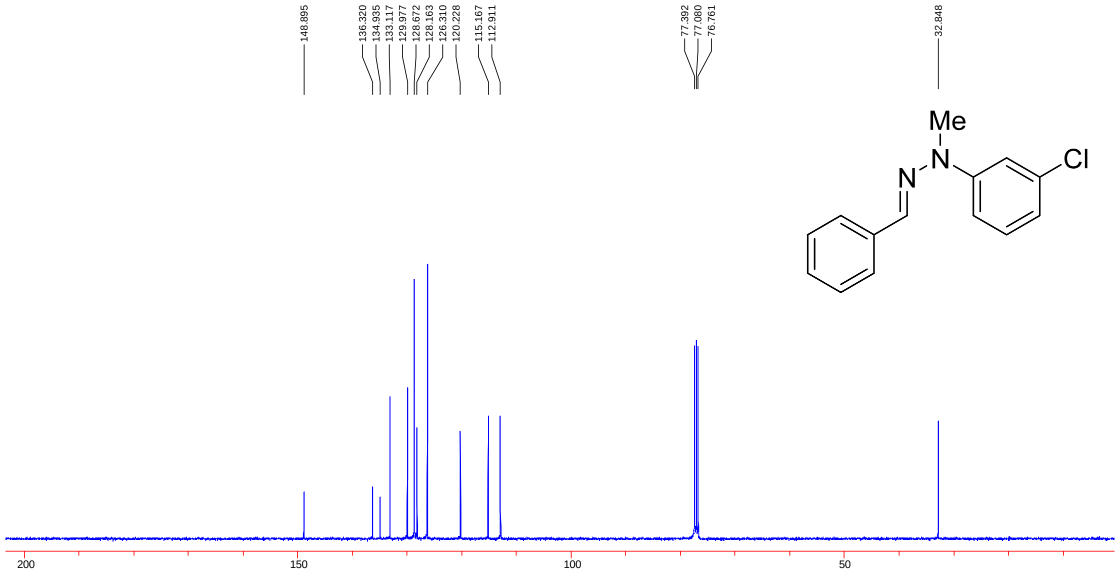

NMR spectra of **1w**

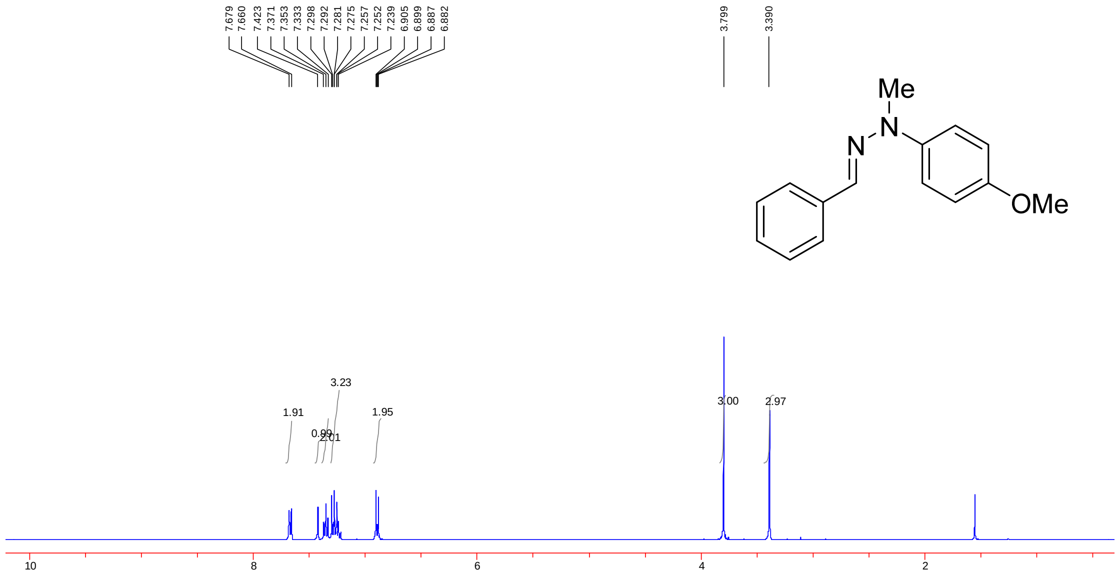

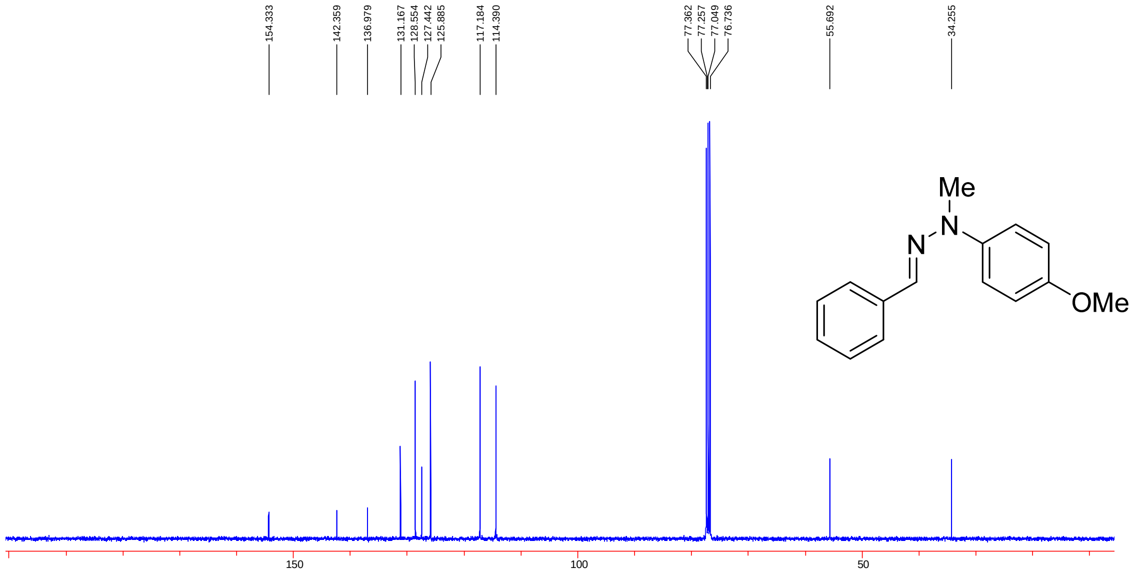

NMR spectra of **1x**

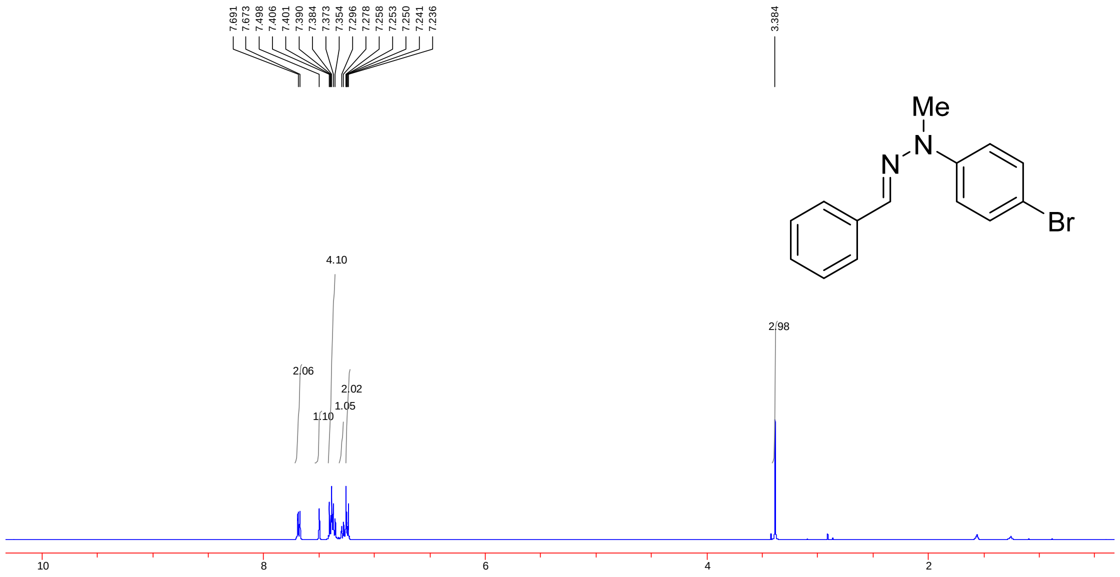

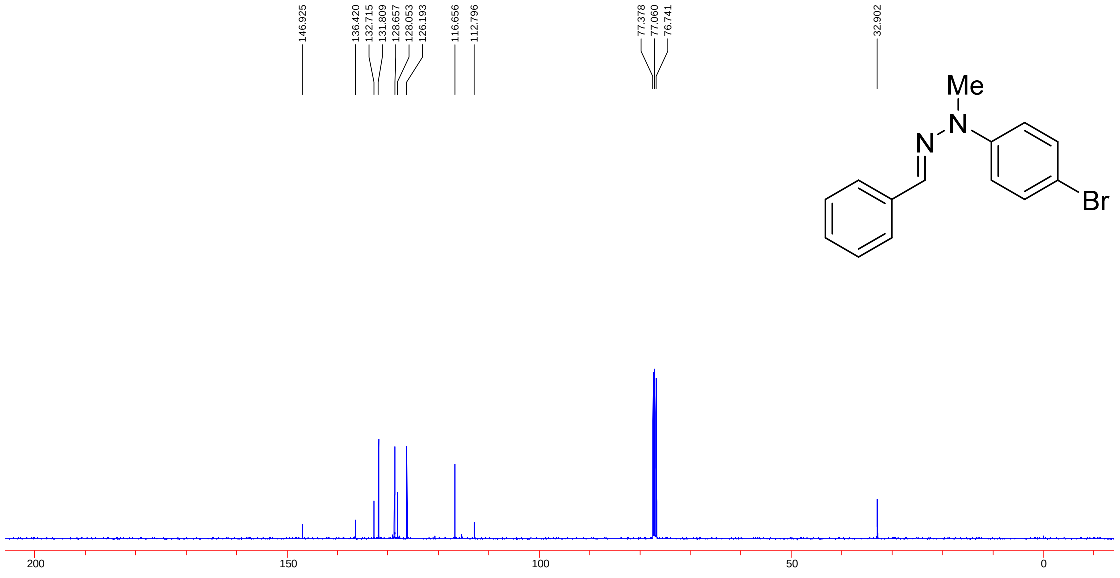

NMR spectra of **3a**

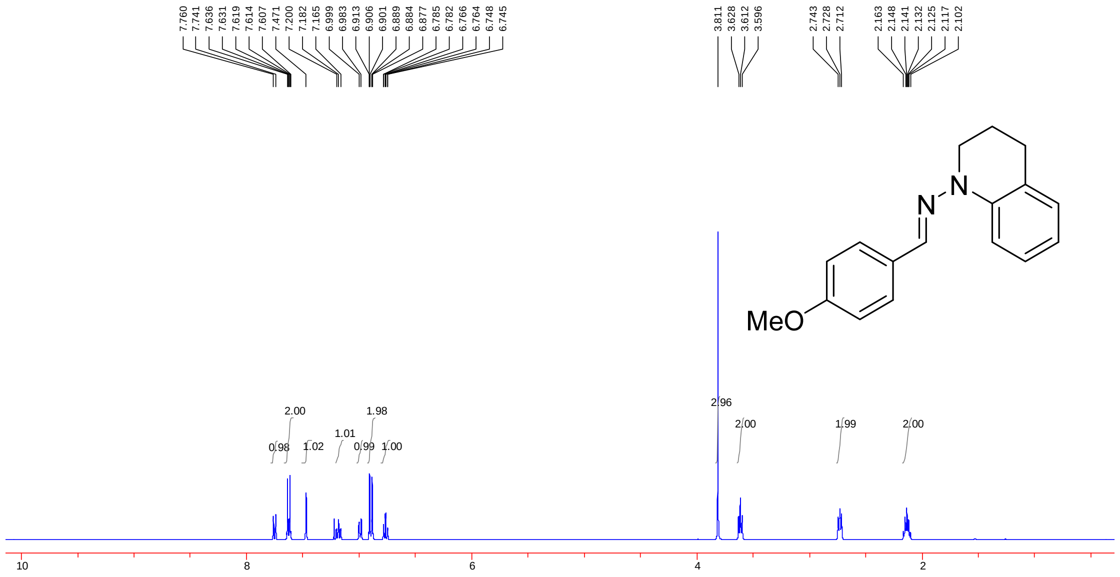

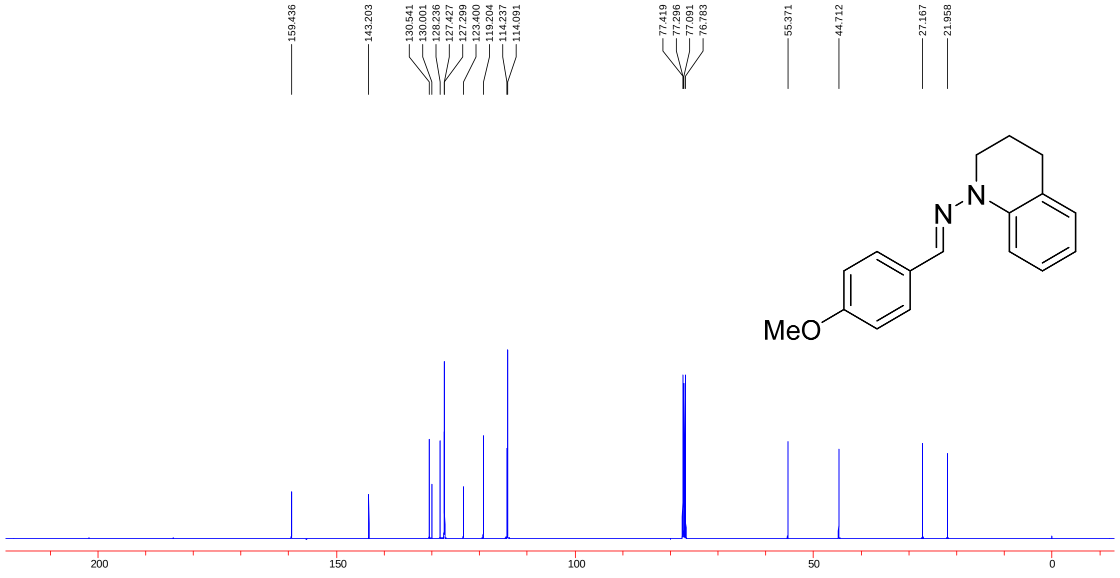

NMR spectra of **3b**

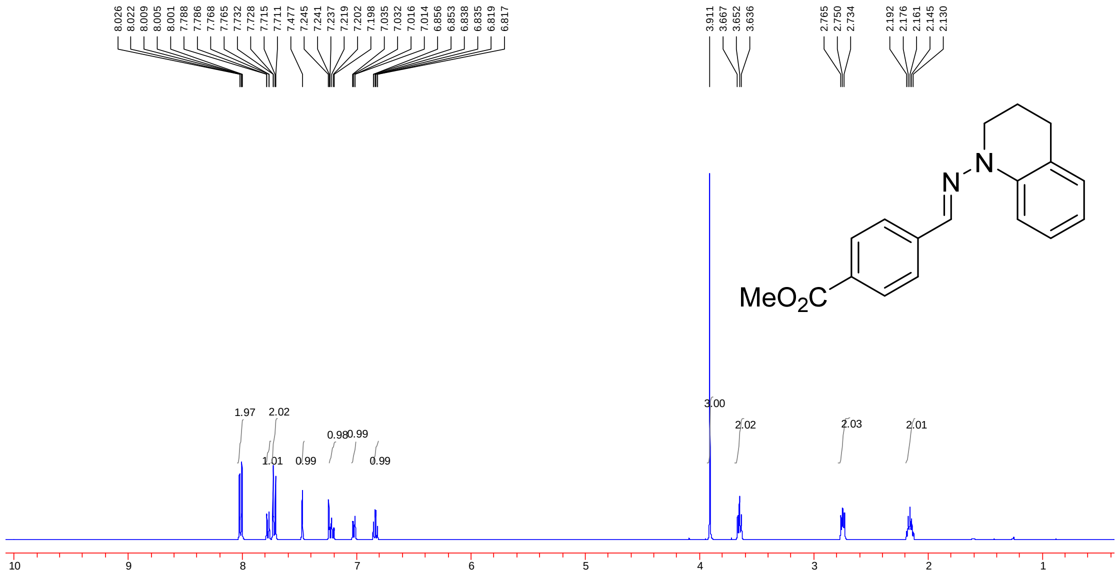

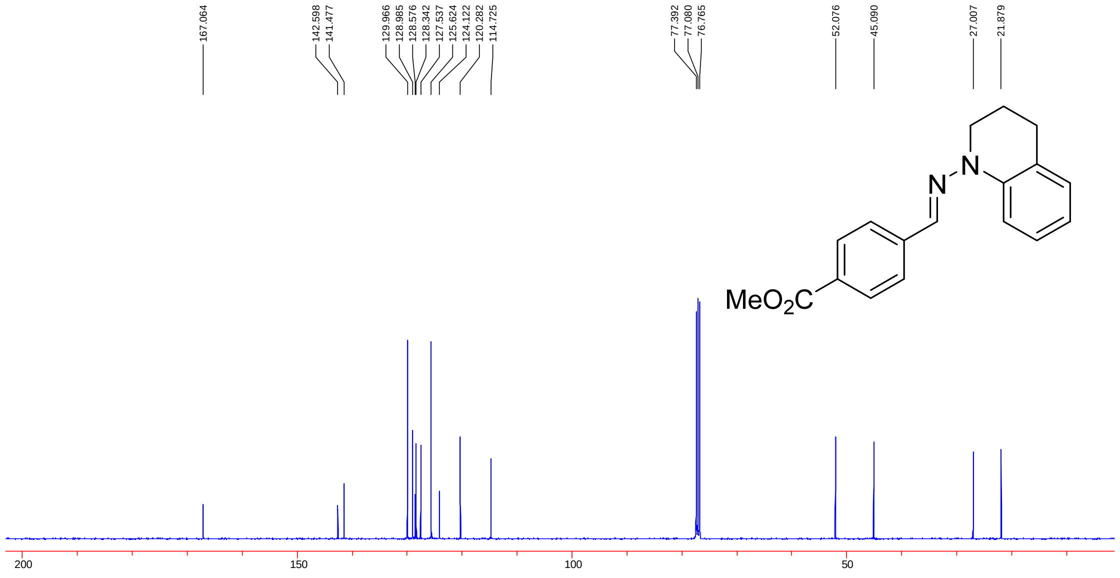

NMR spectra of **3c**

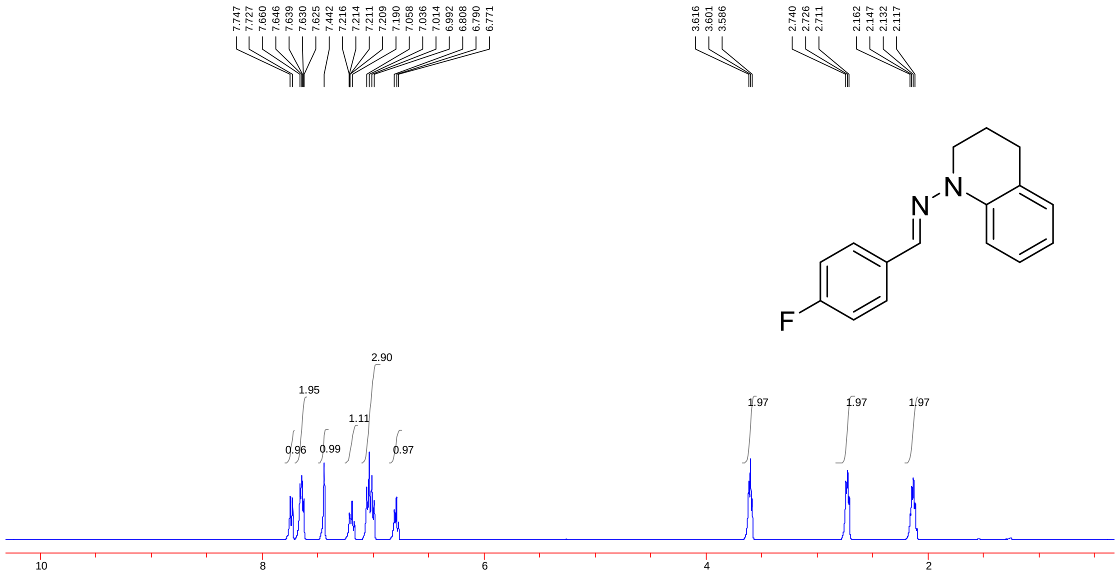

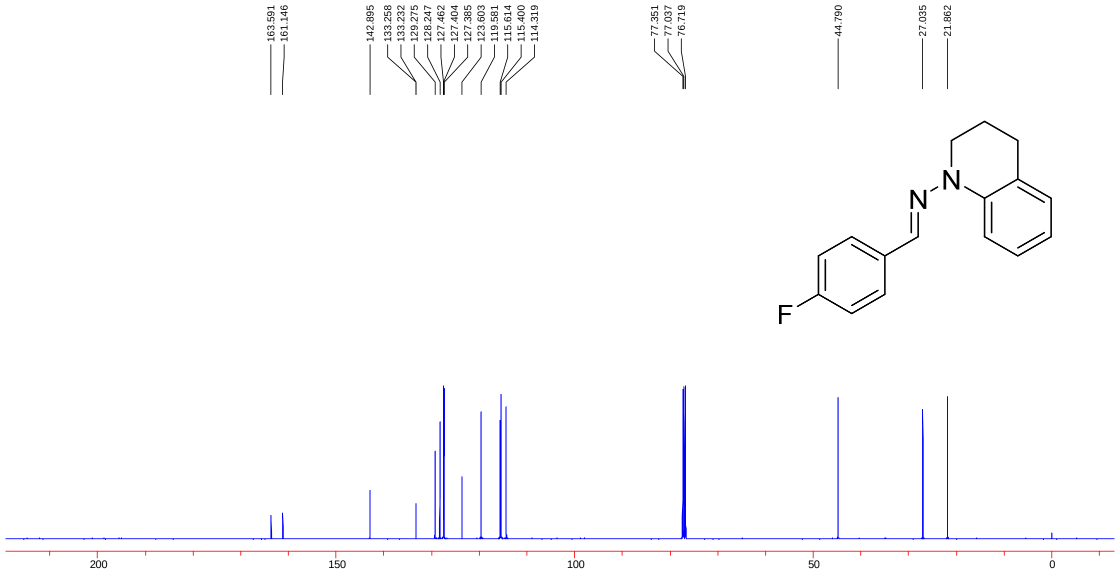

NMR spectra of **3d**

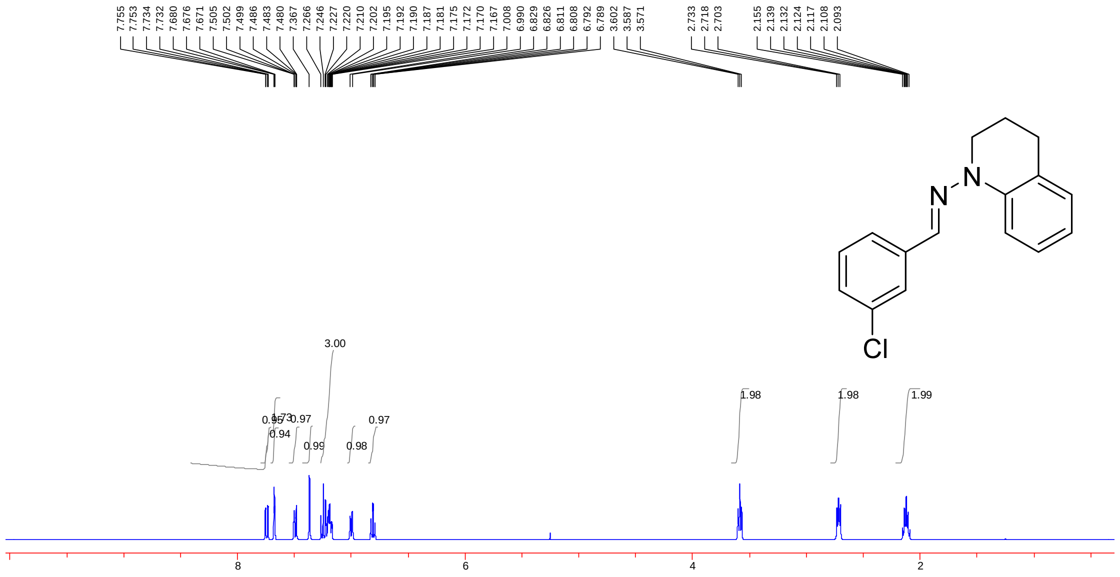

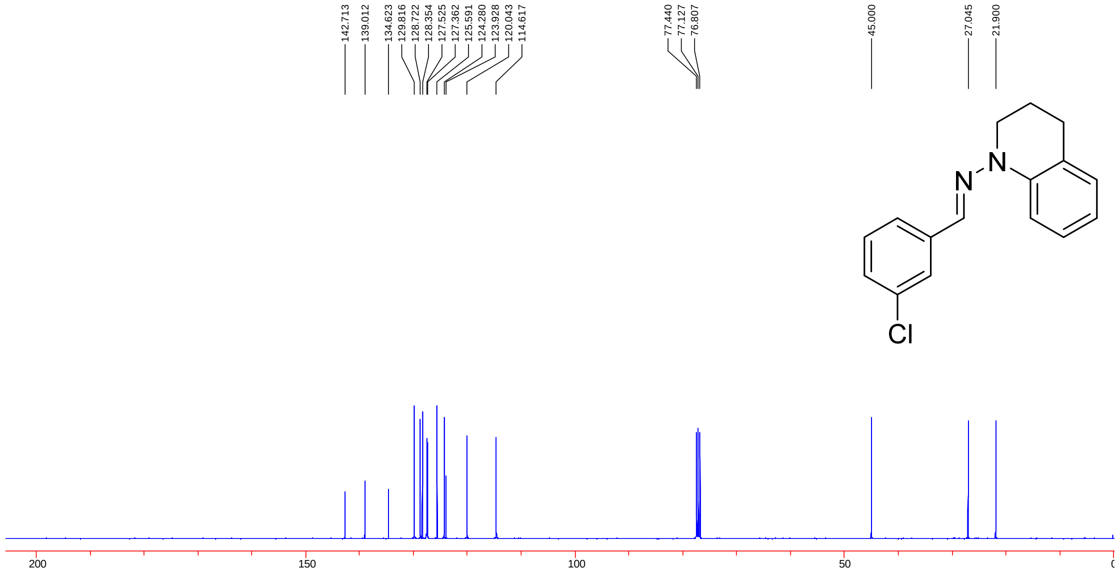

NMR spectra of **3e**

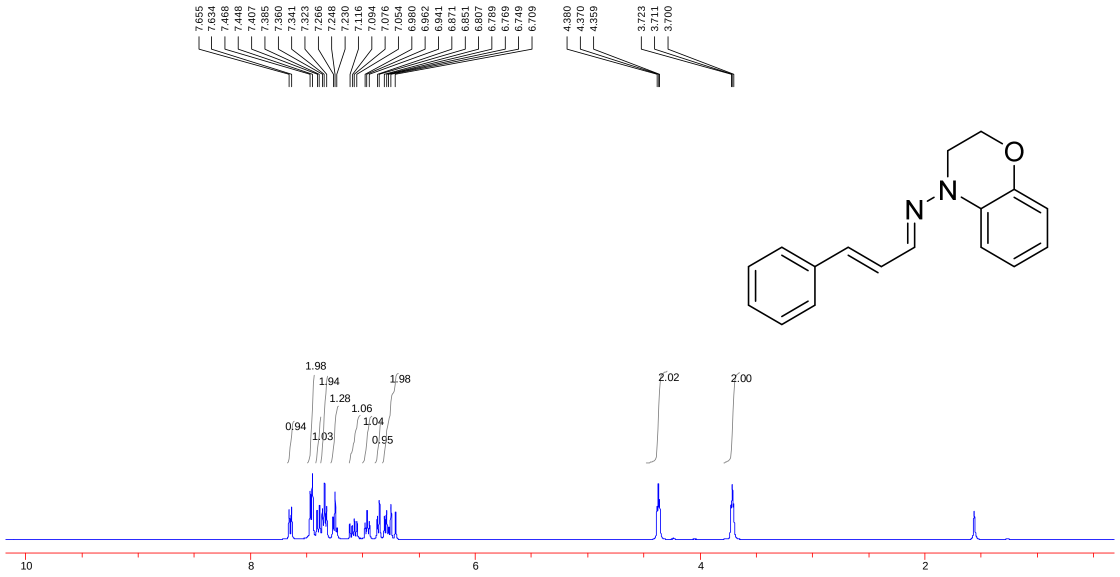

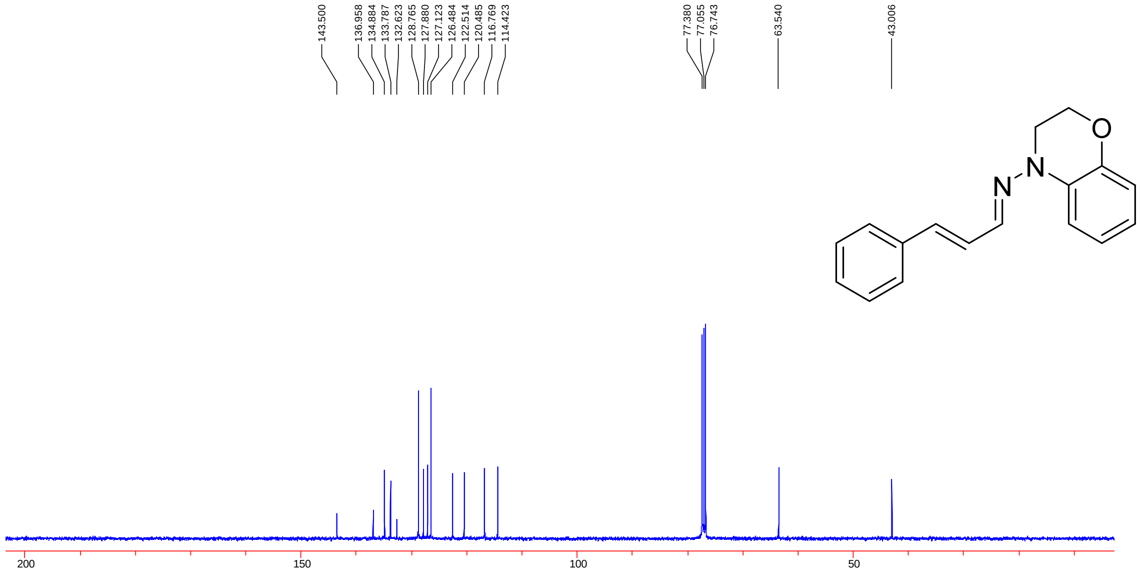

NMR spectra of **3f**

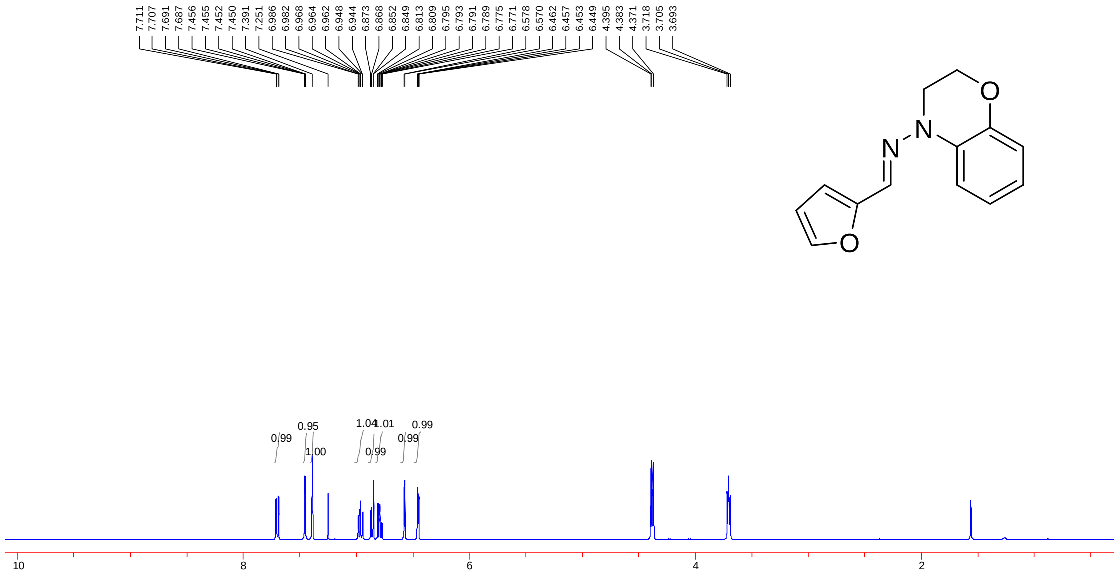

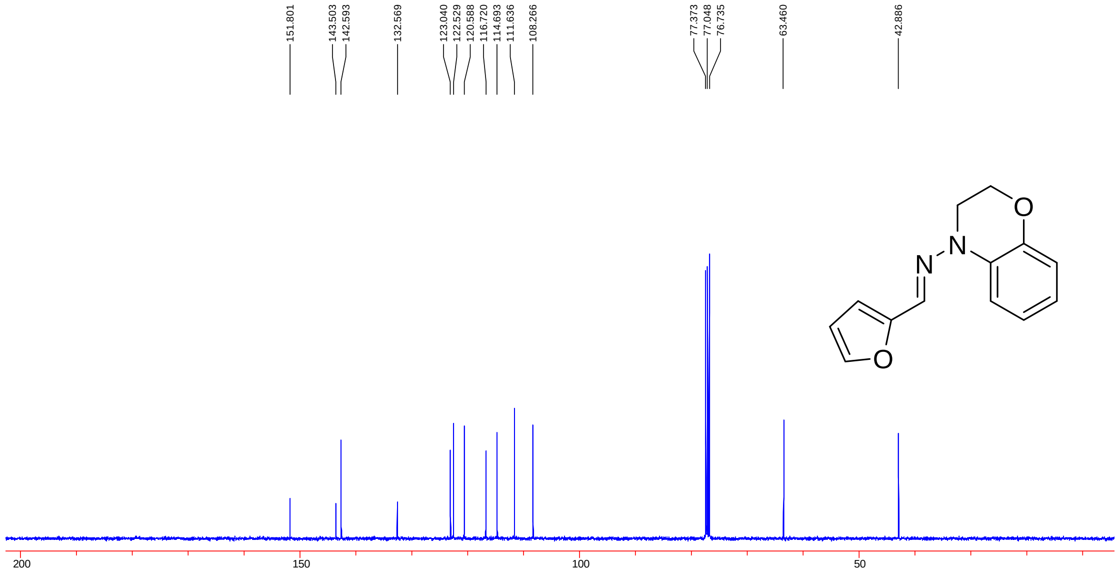

NMR spectra of **2a**

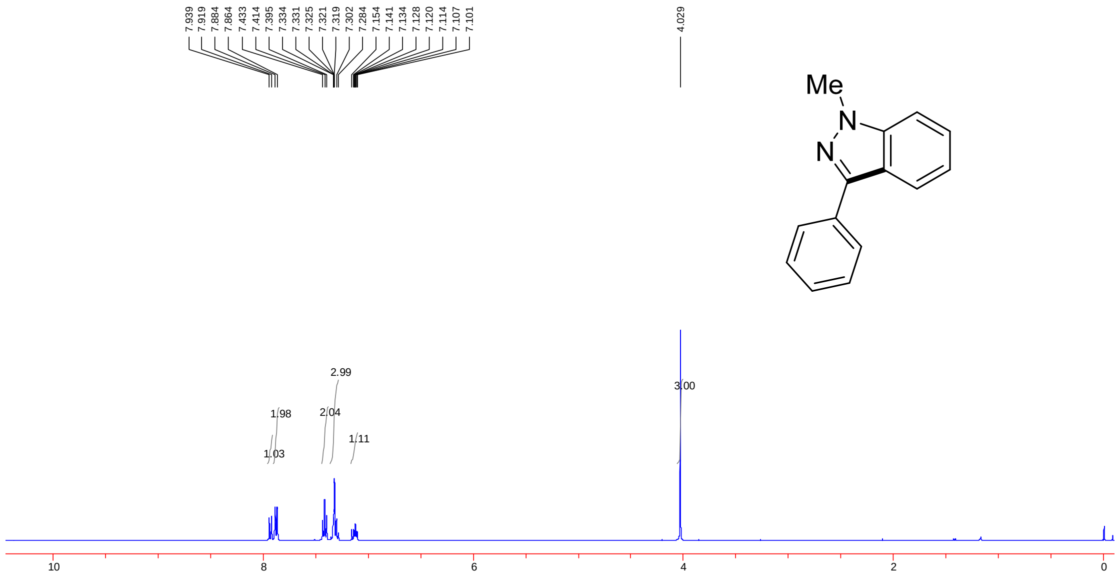

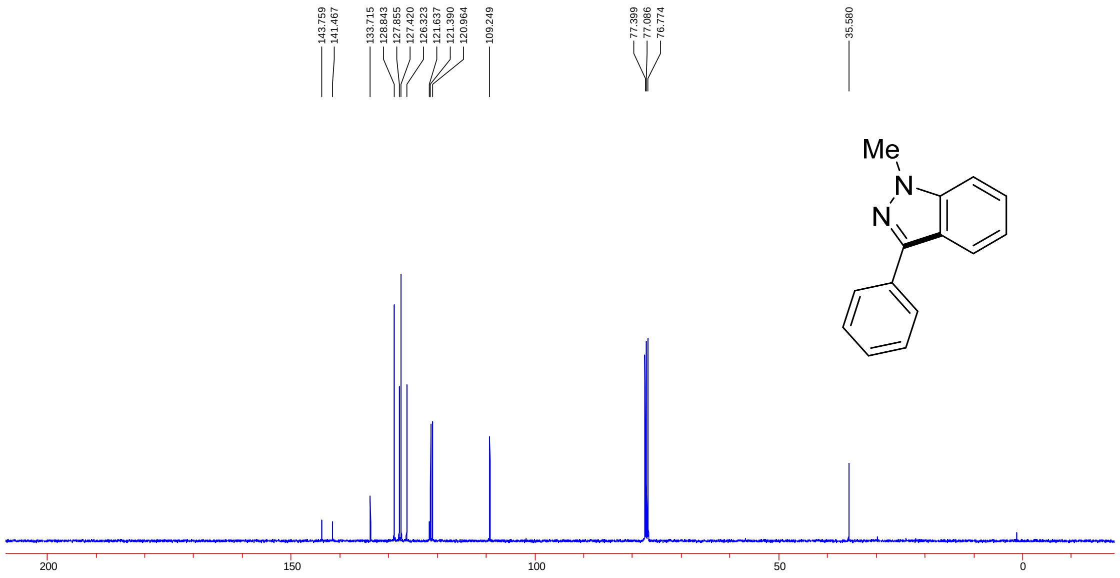

NMR spectra of **2b**

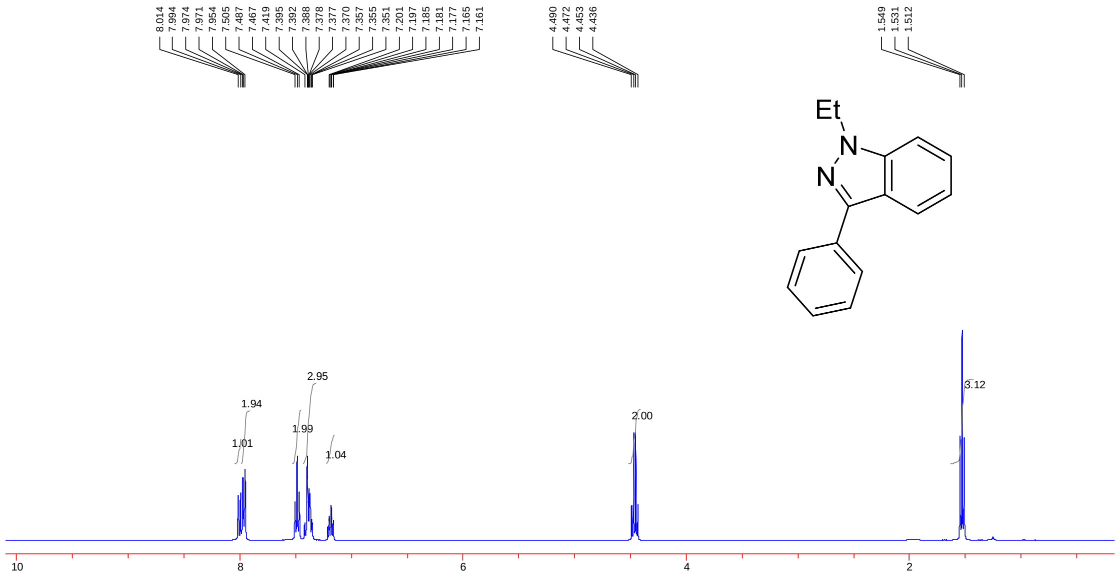

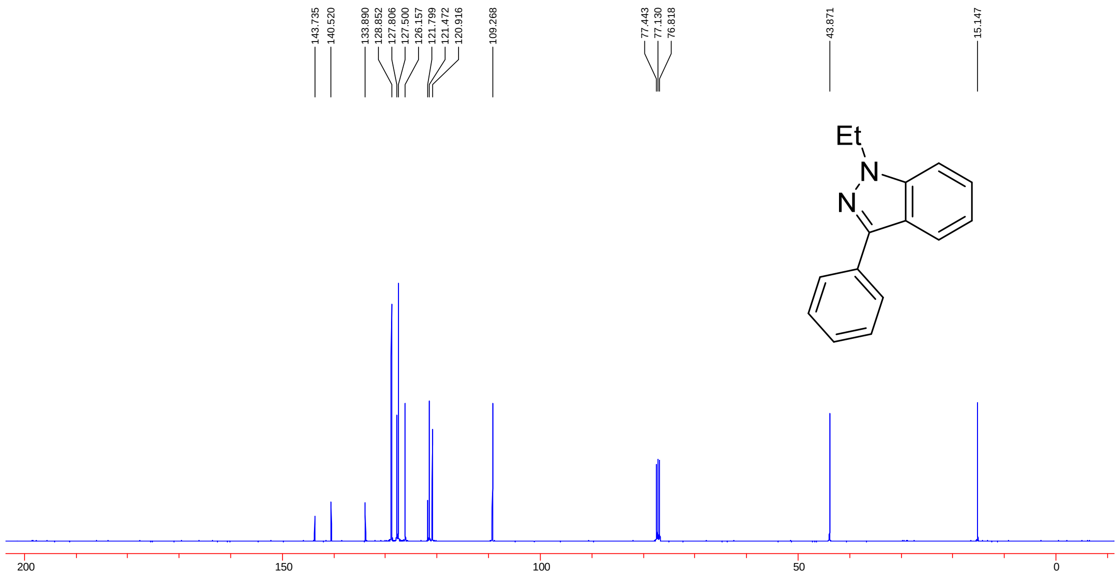

NMR spectra of **2c**

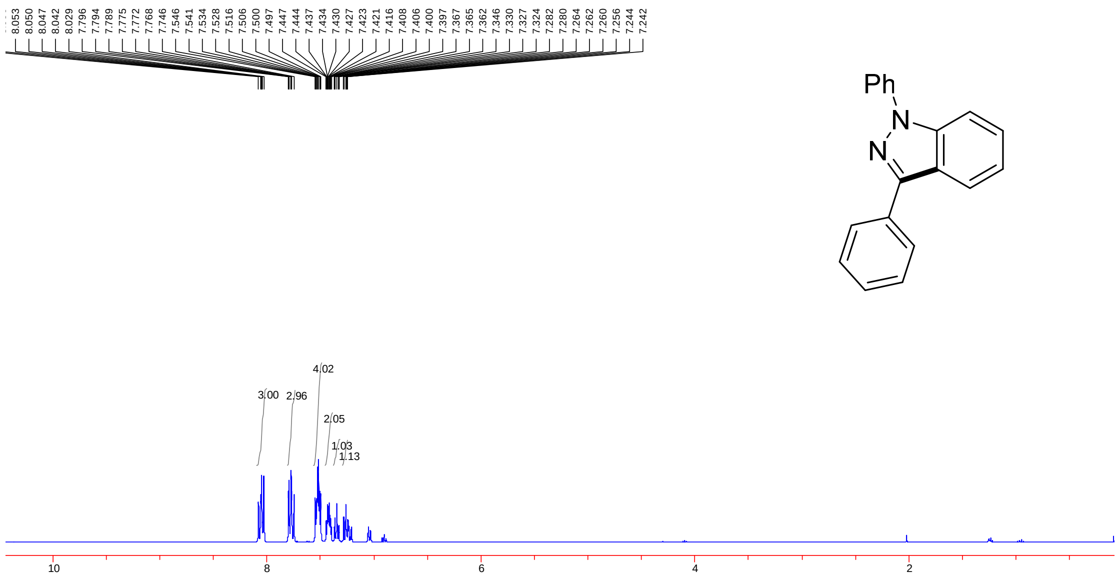

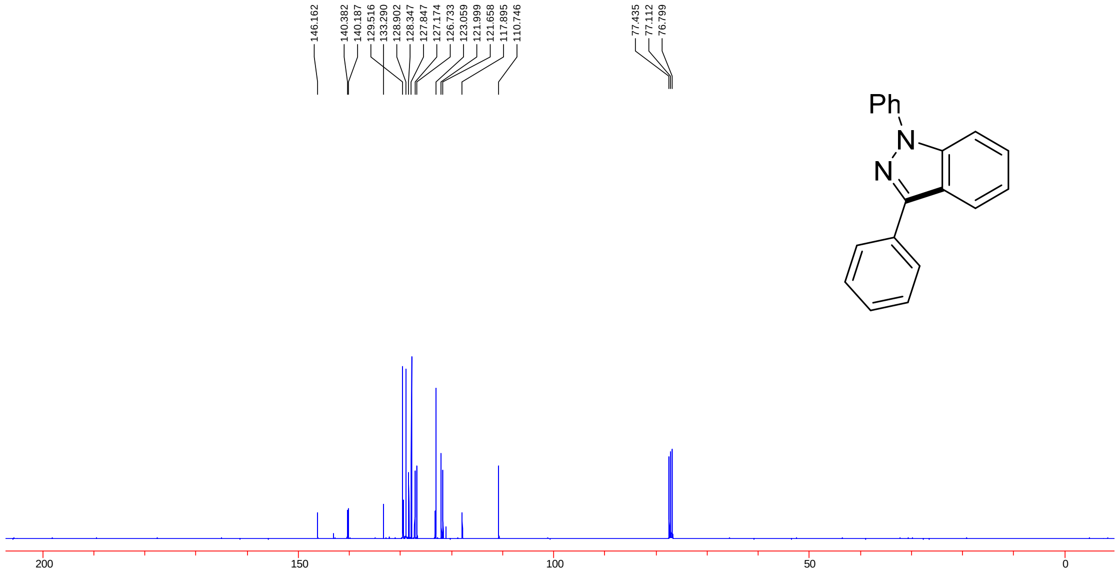

NMR spectra of **2d**

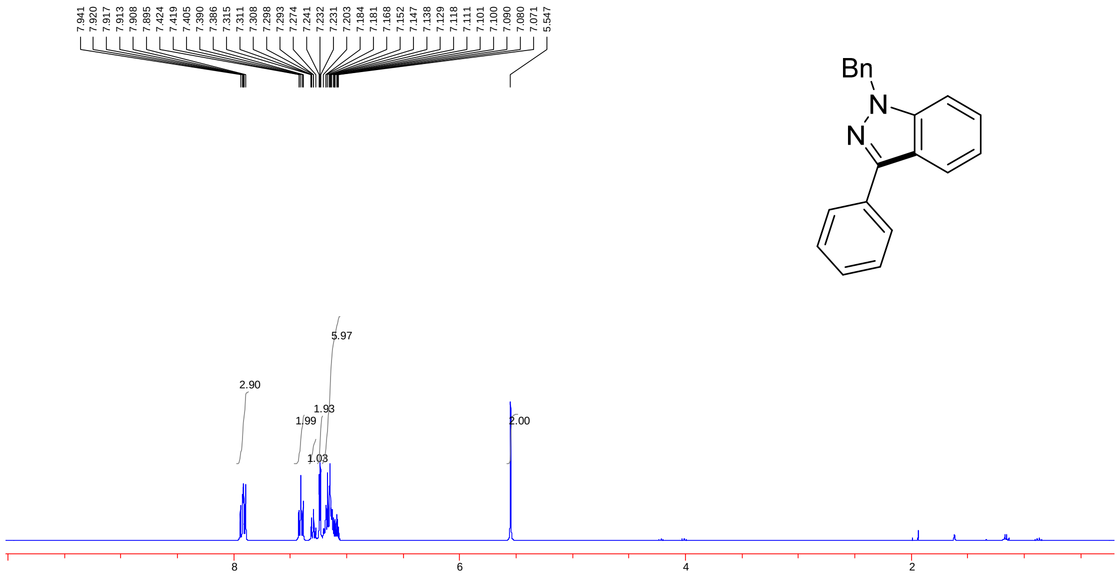

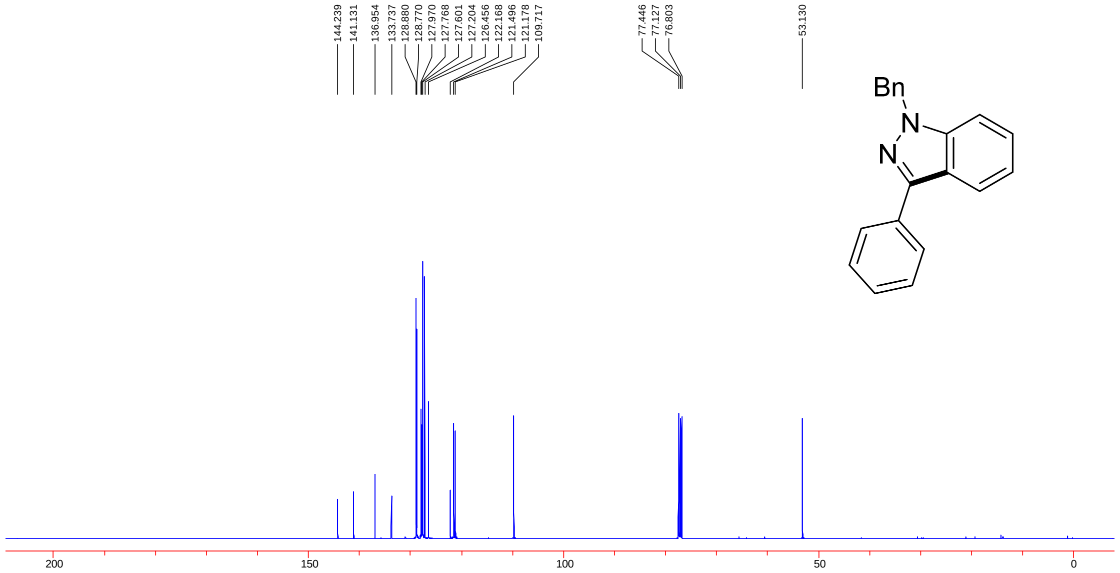

NMR spectra of **2e**

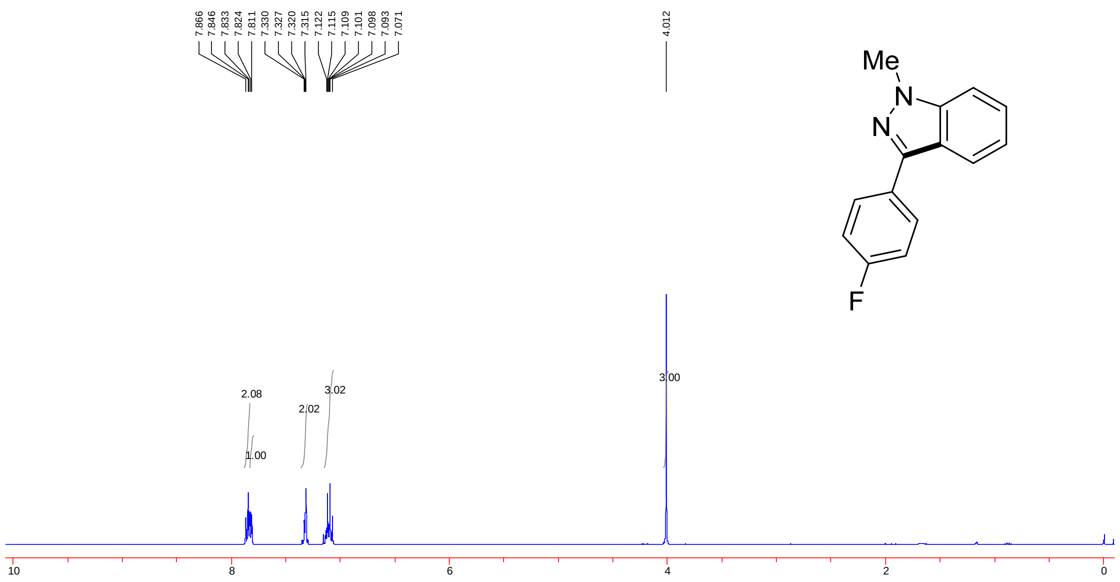

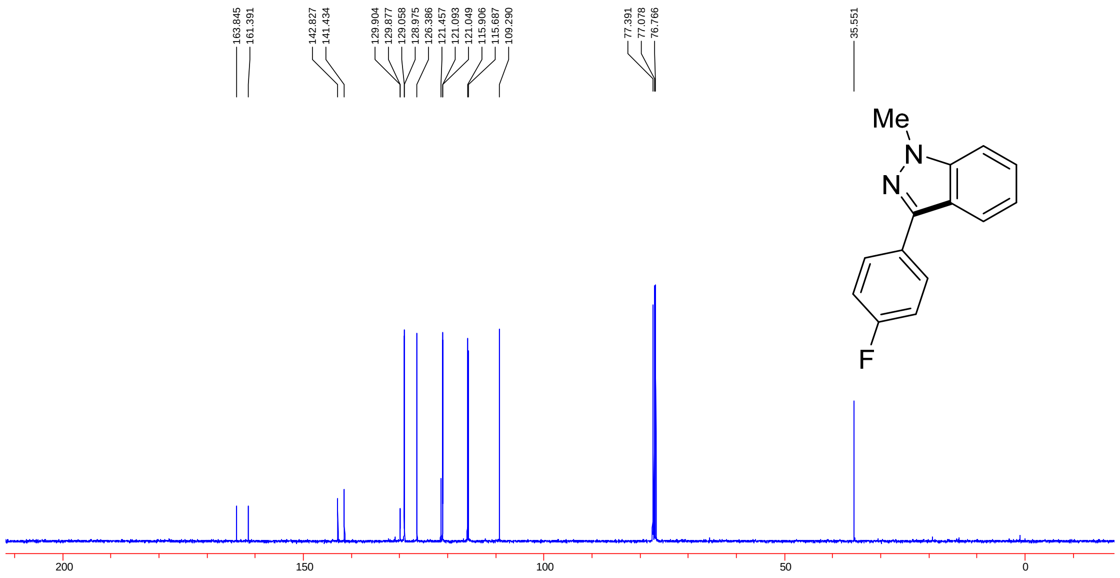

NMR spectra of **2f**

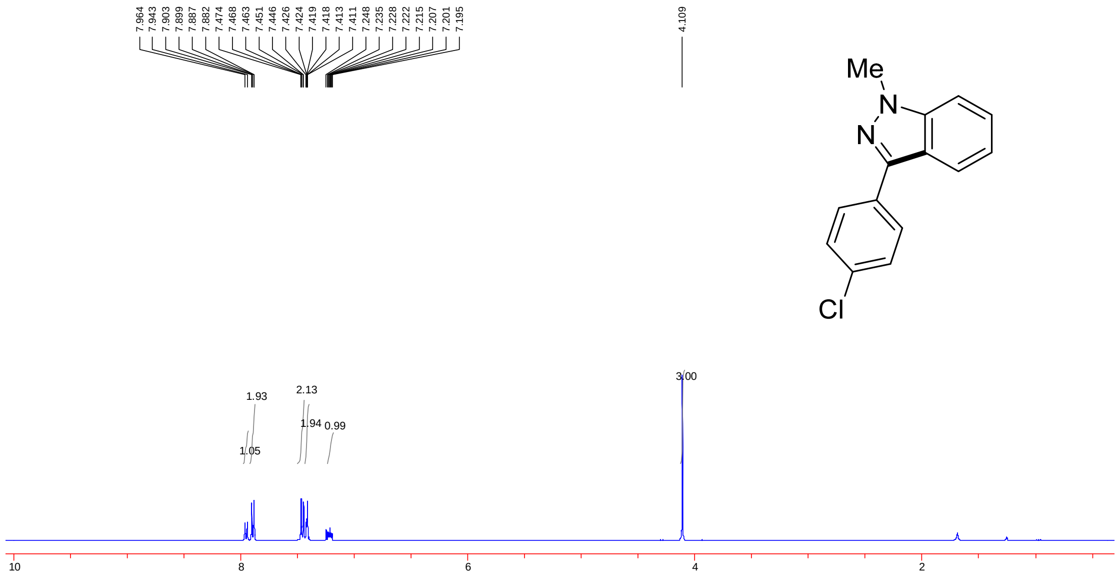

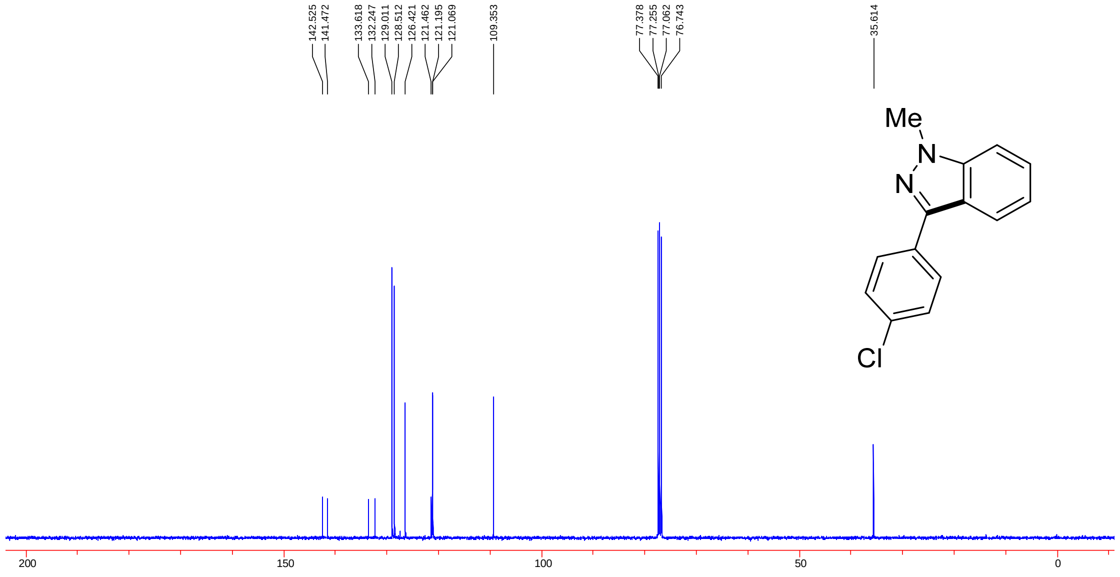

NMR spectra of **2g**

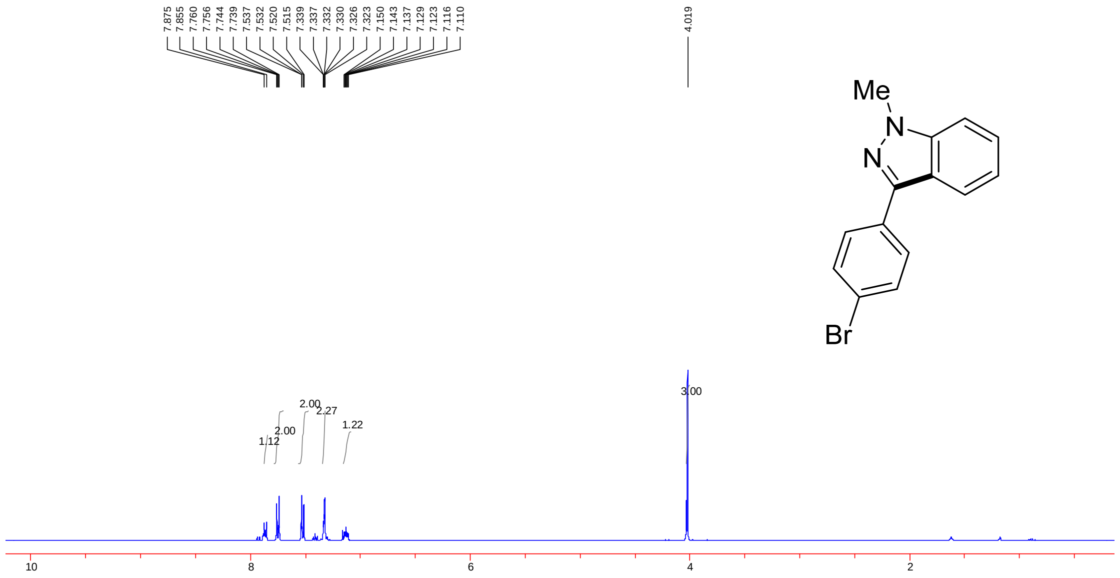

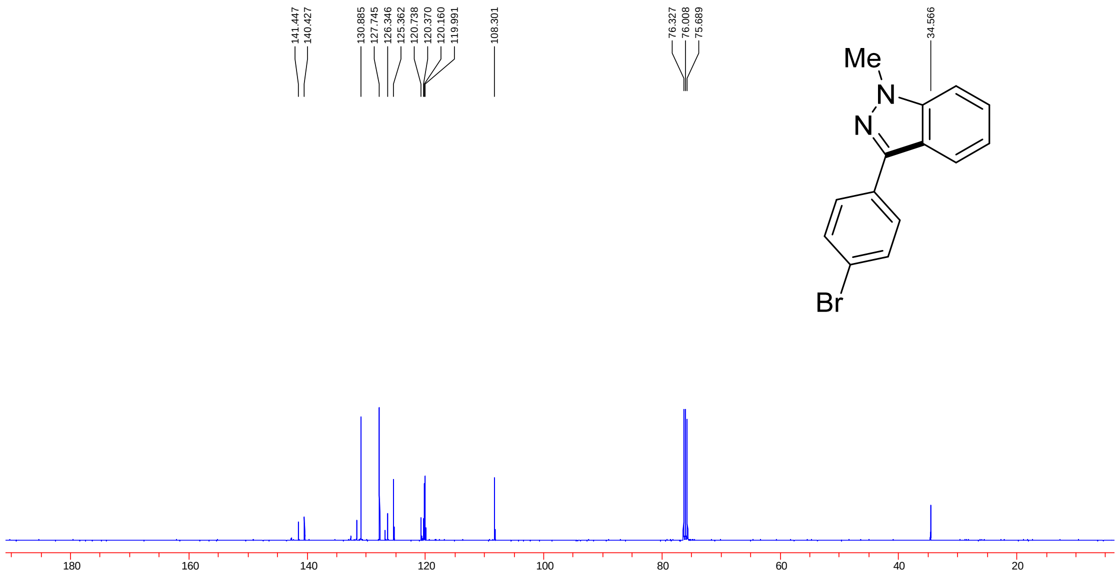

NMR spectra of **2h**

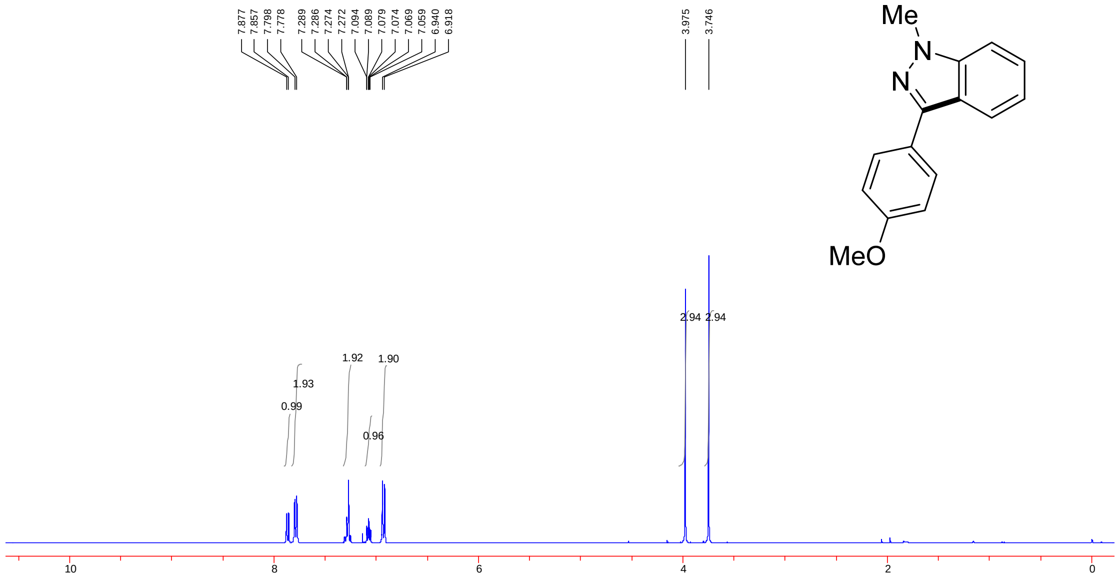

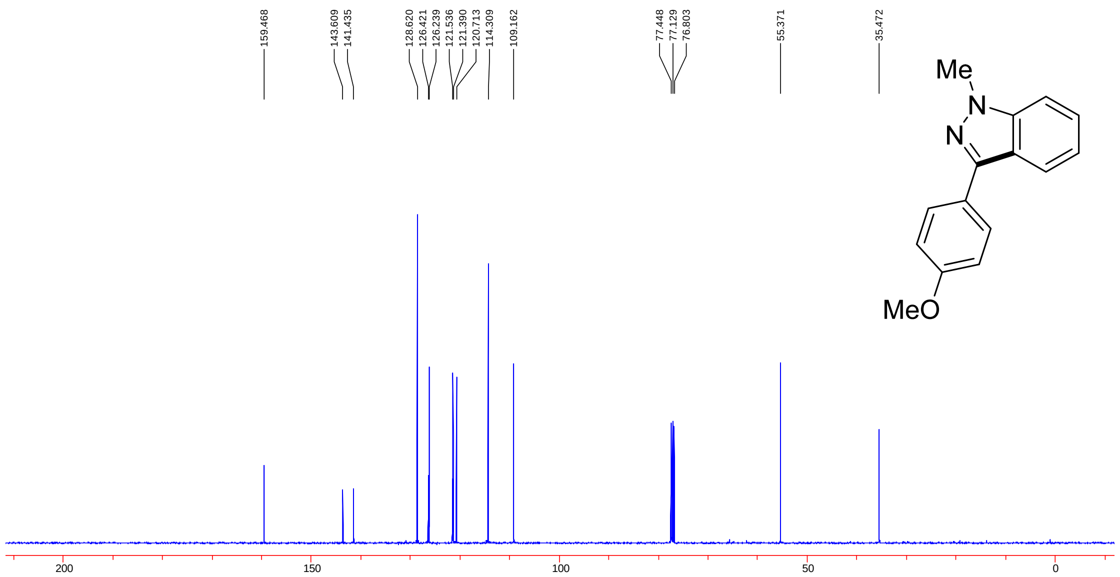

NMR spectra of **2i**

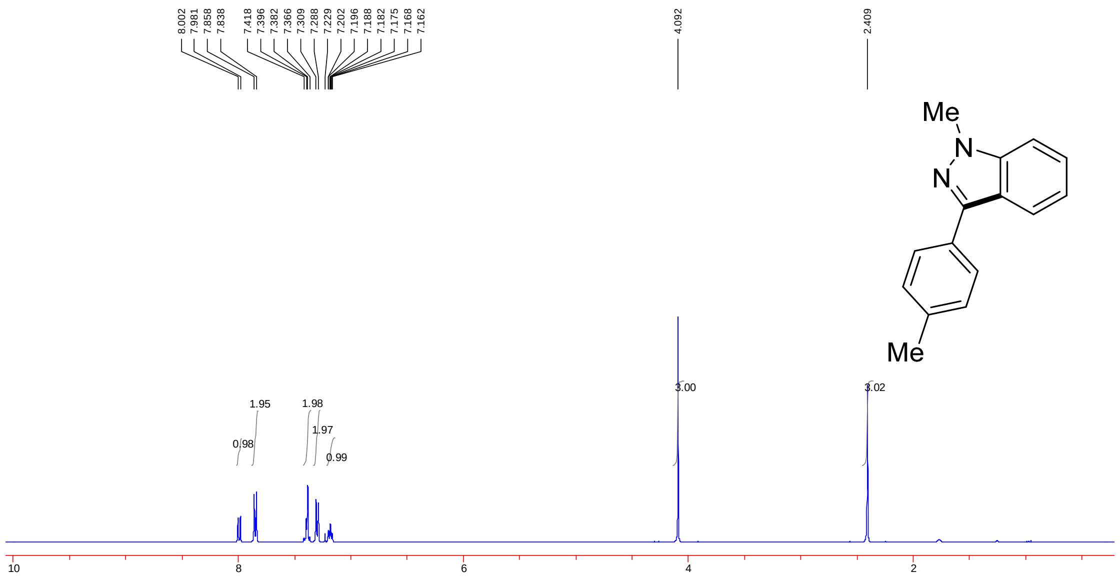

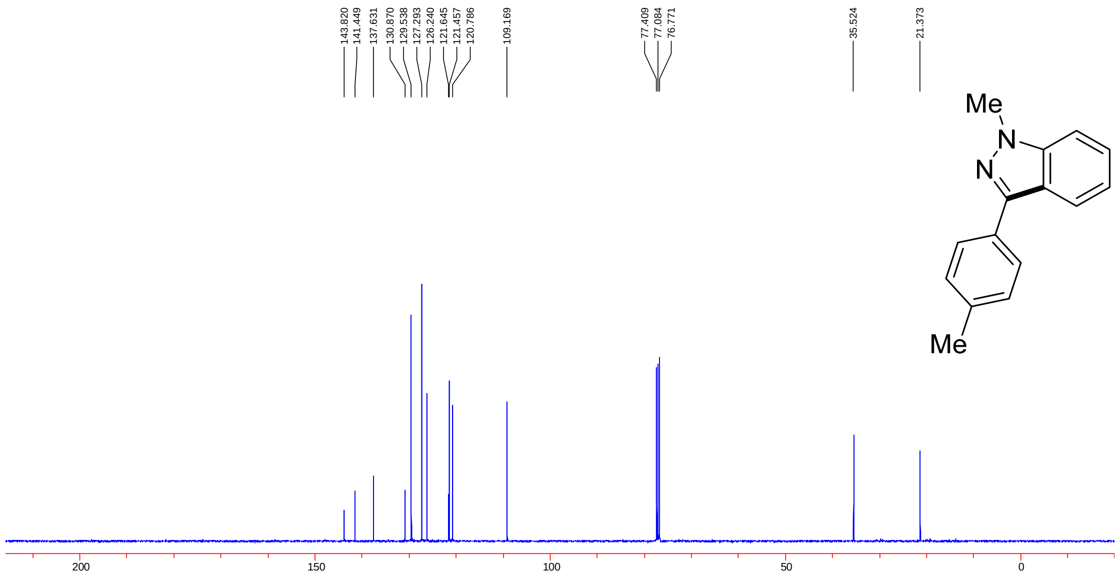

NMR spectra of **2j**

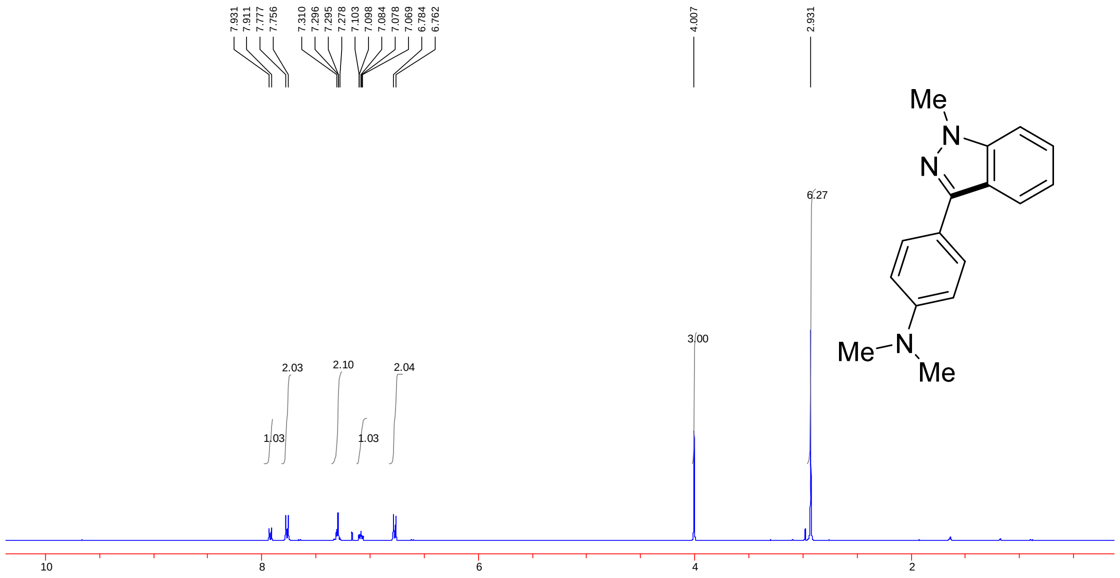

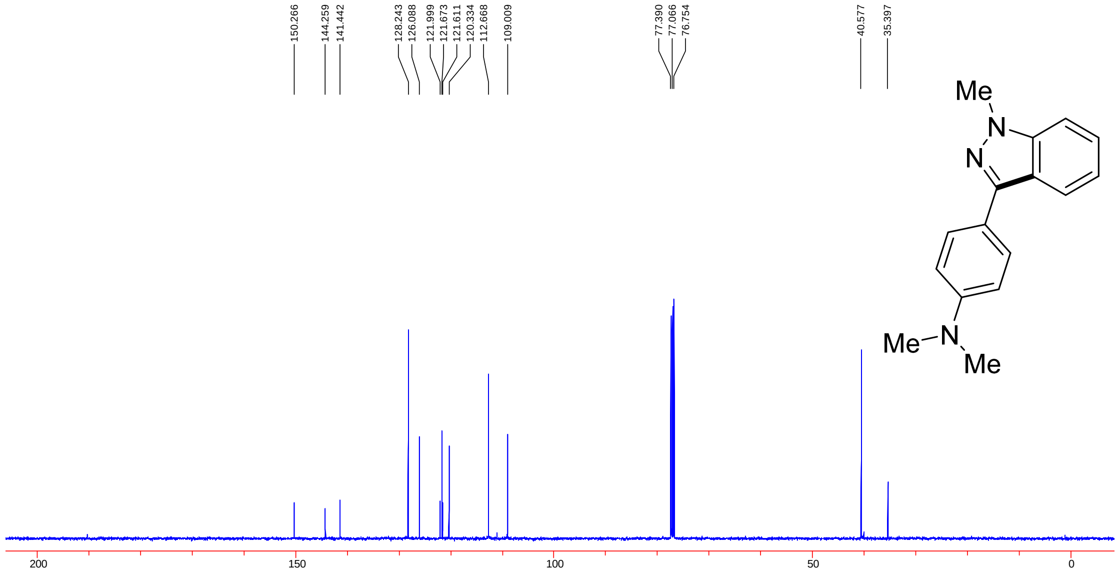

NMR spectra of **2k**

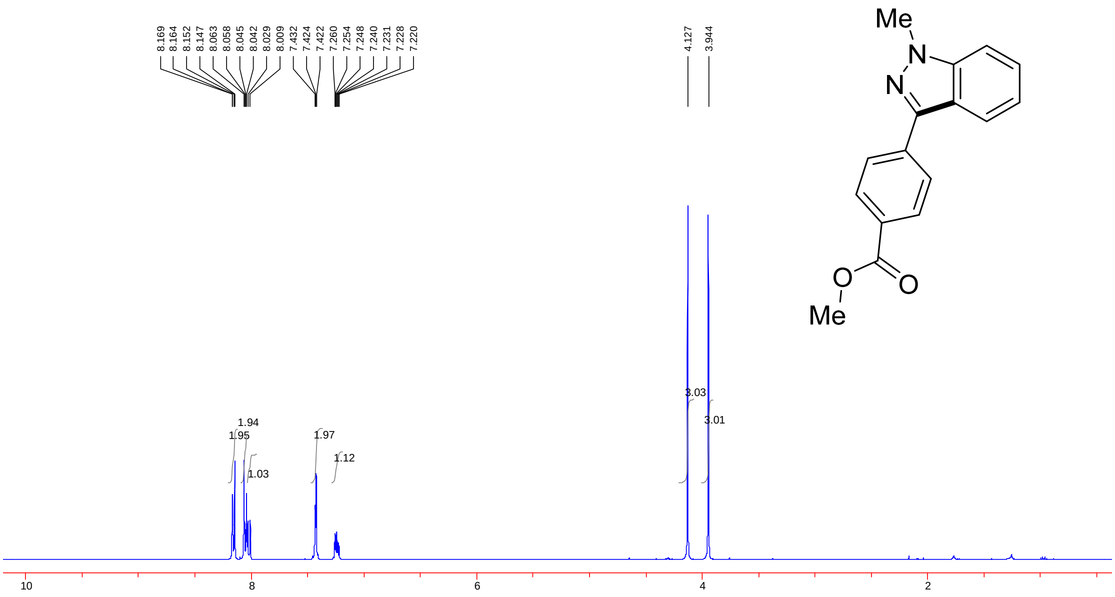

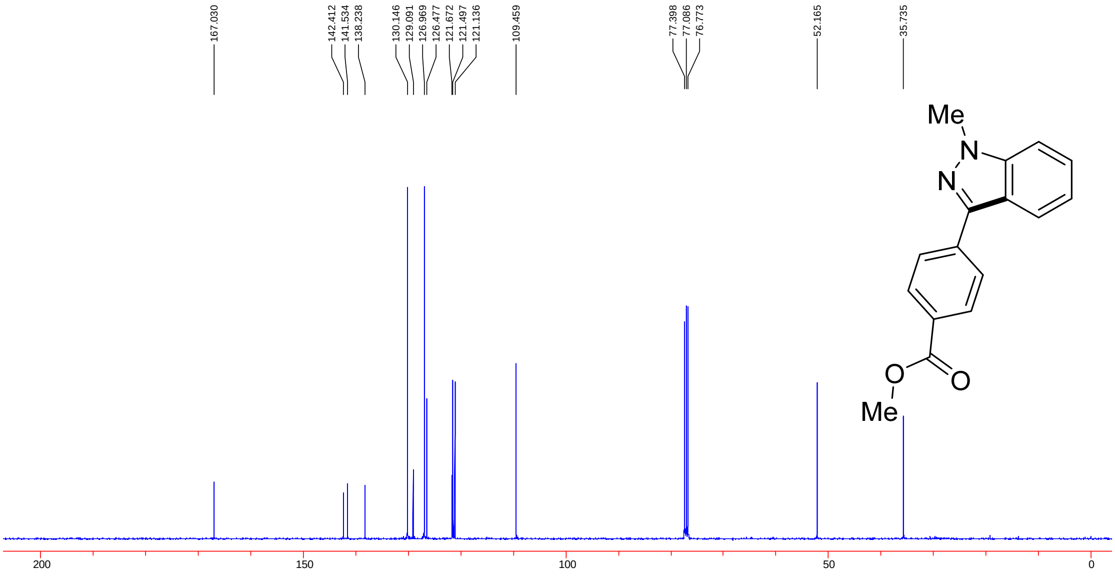

NMR spectra of **2l**

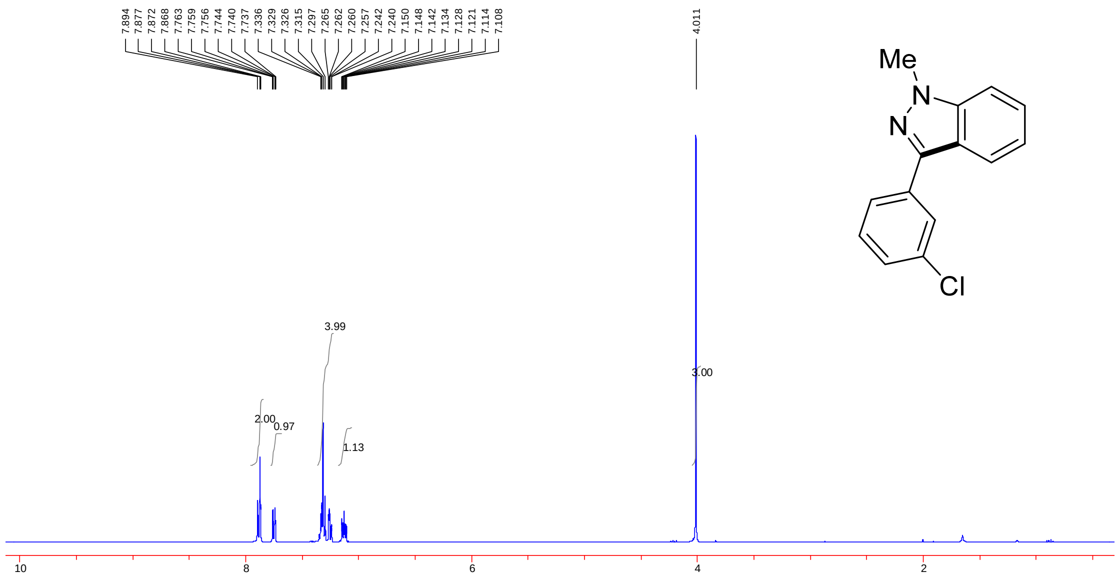

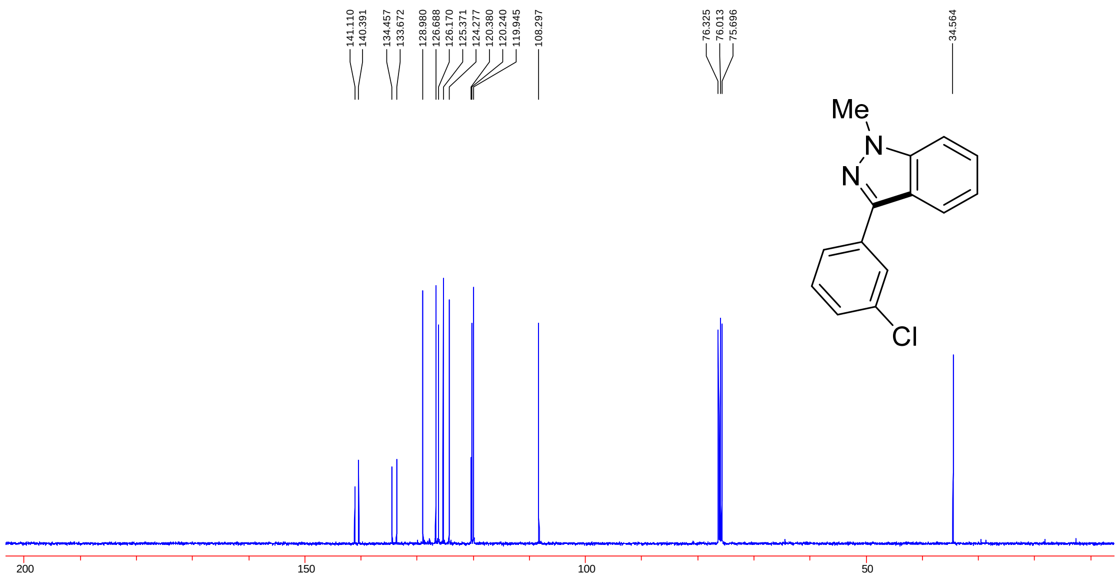

NMR spectra of **2m**

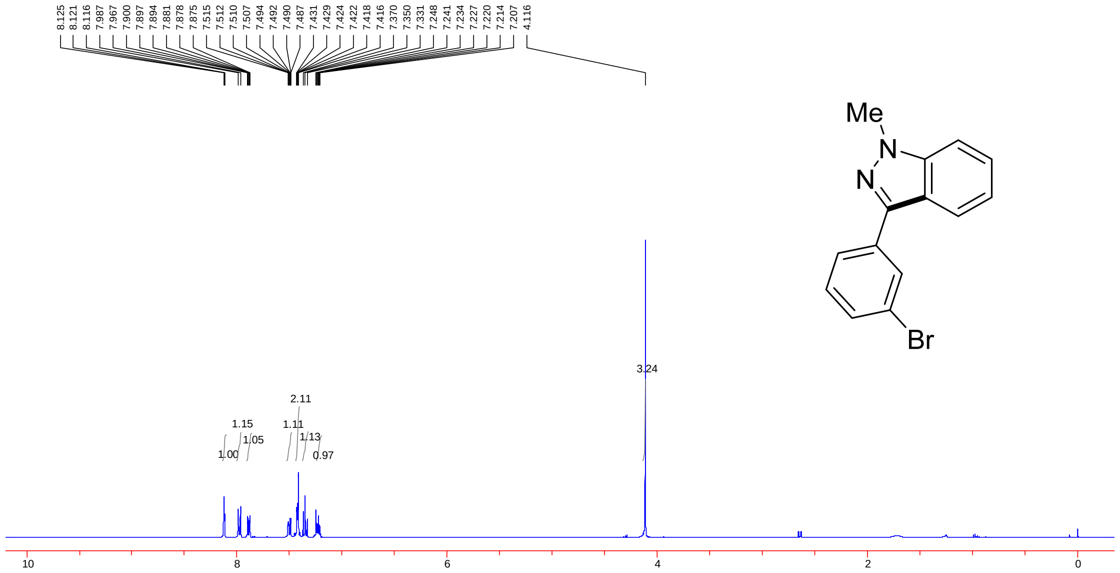

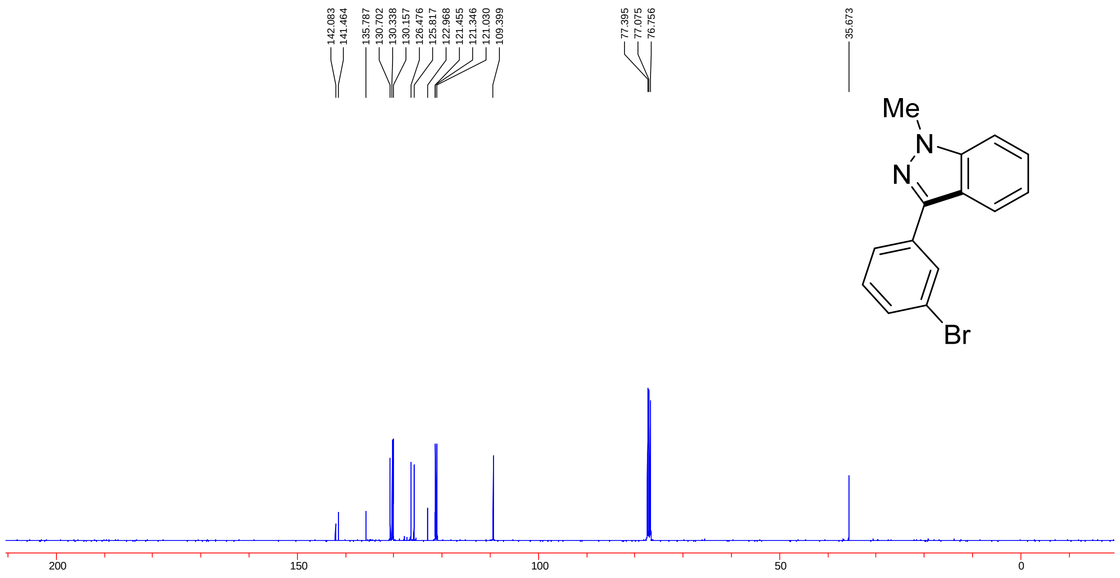

NMR spectra of **2n**

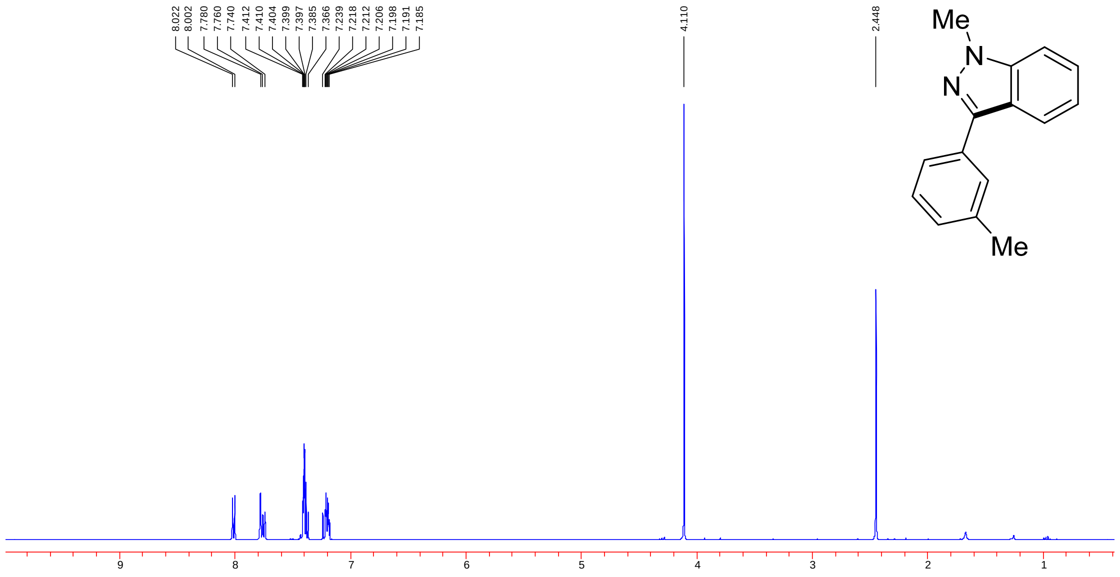

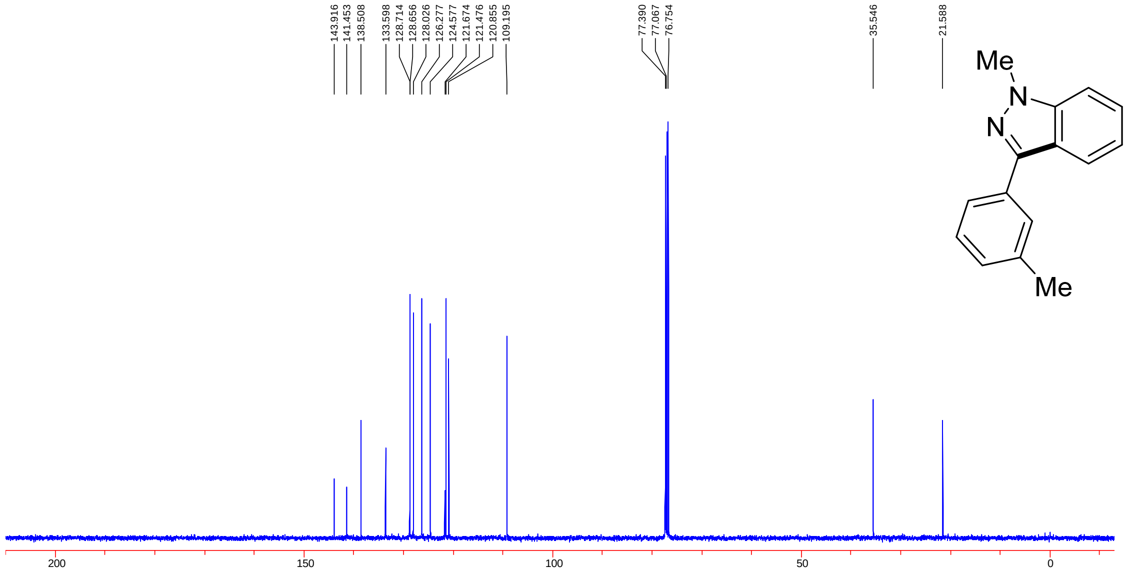

NMR spectra of **2o**

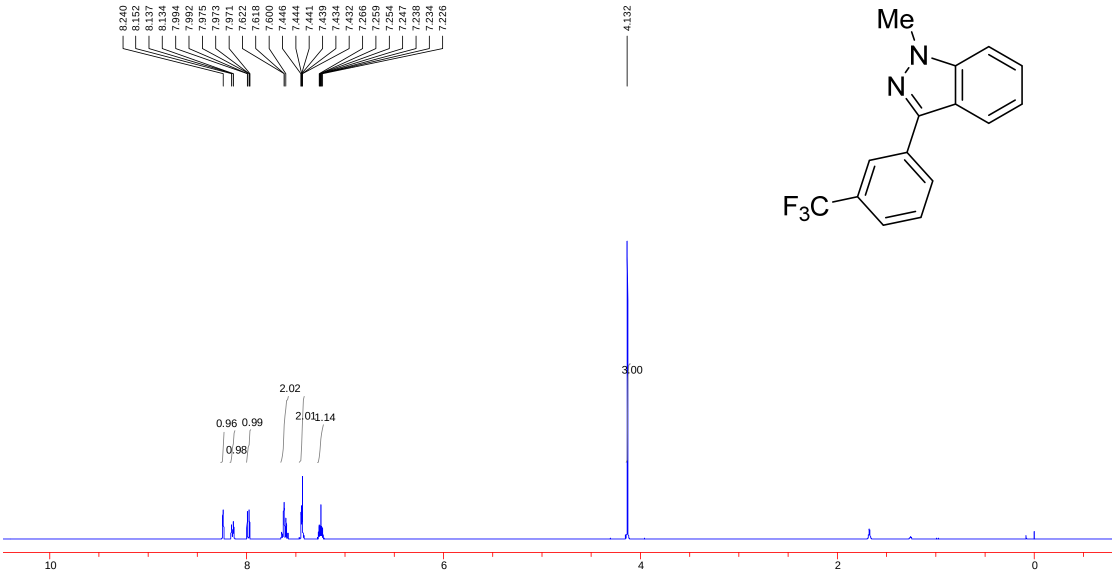

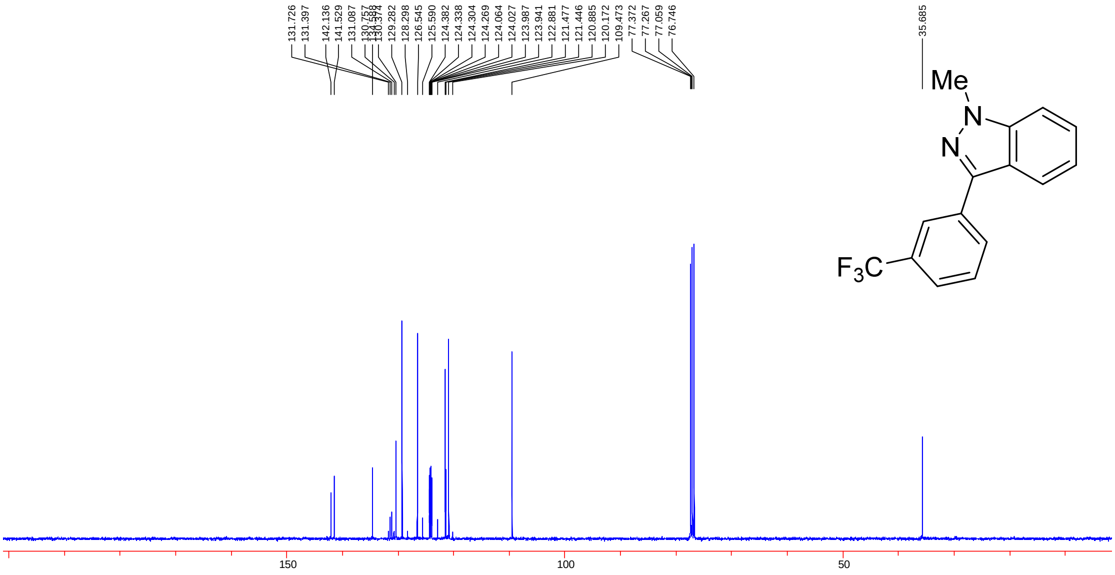

NMR spectra of **2p**

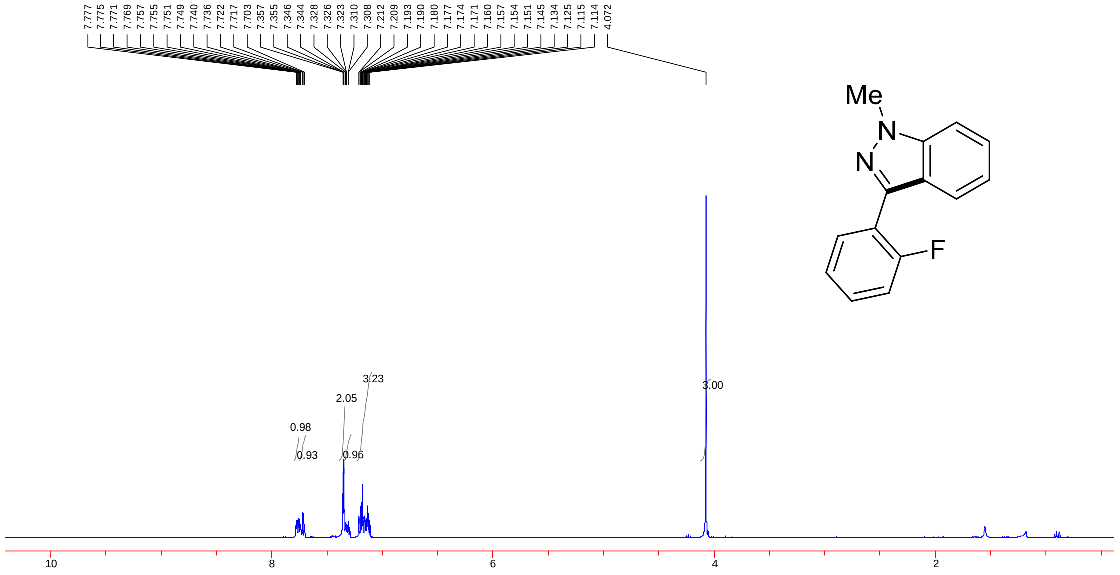

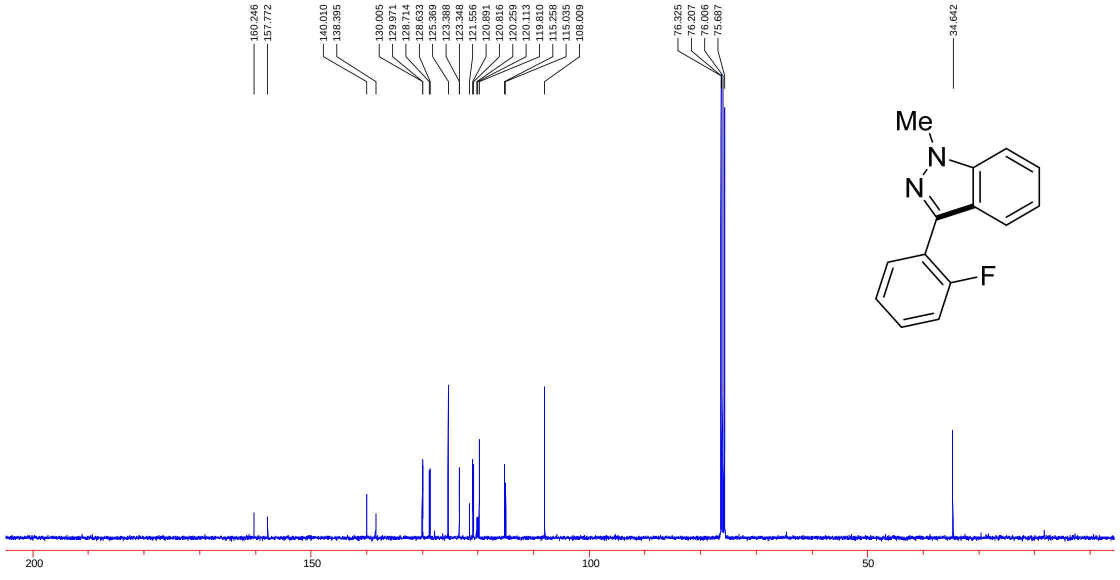

NMR spectra of **2q**

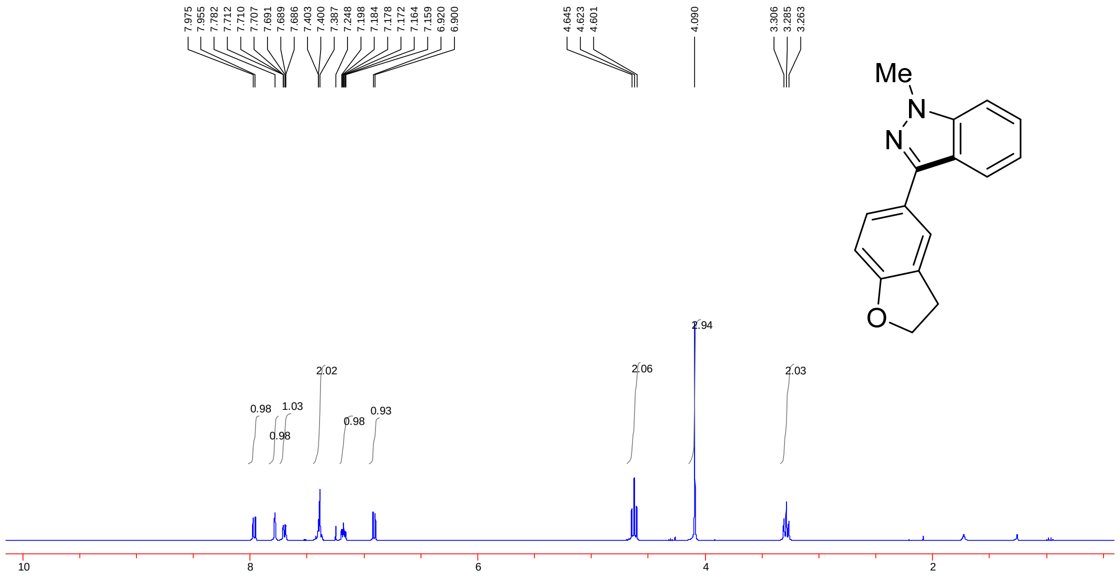

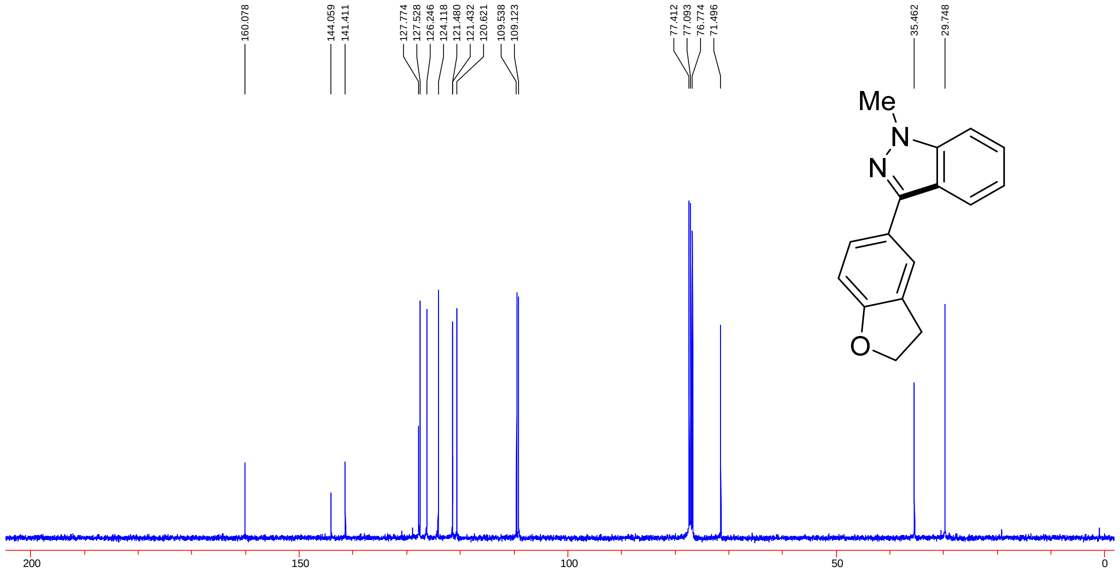

NMR spectra of **2r**

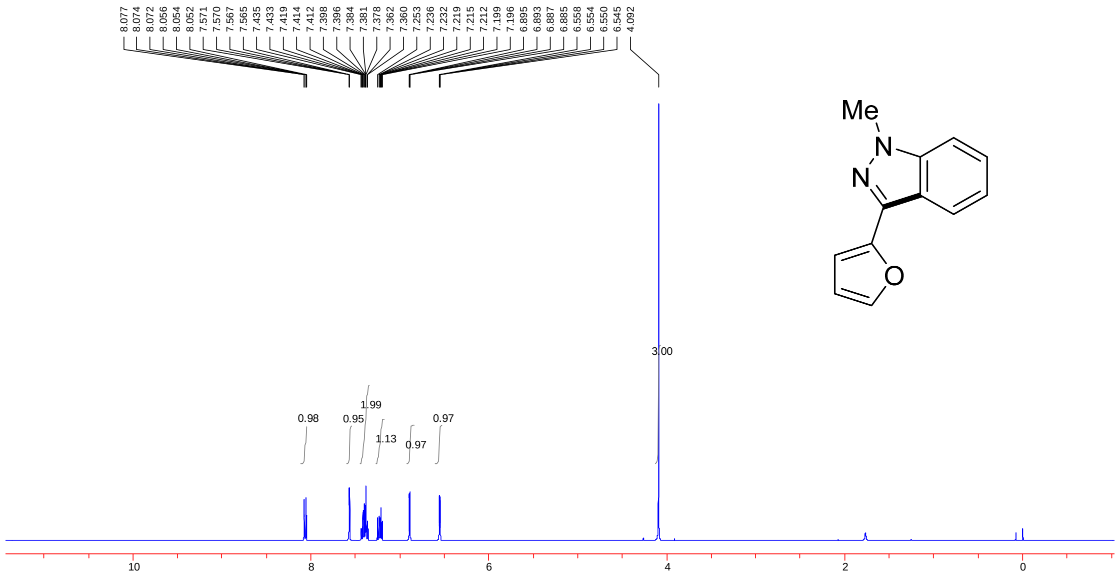

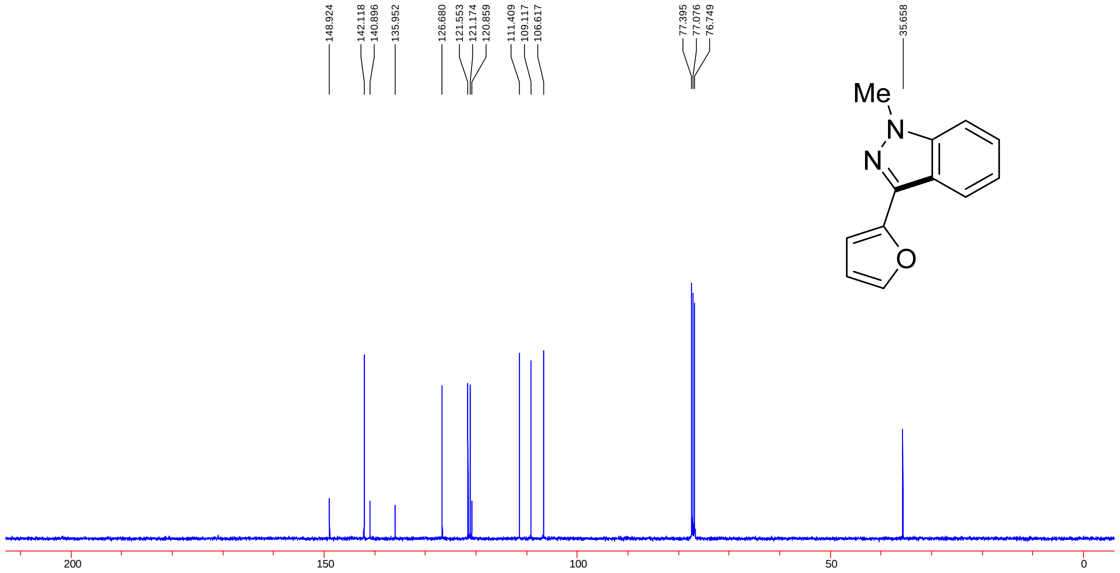

NMR spectra of **2s**

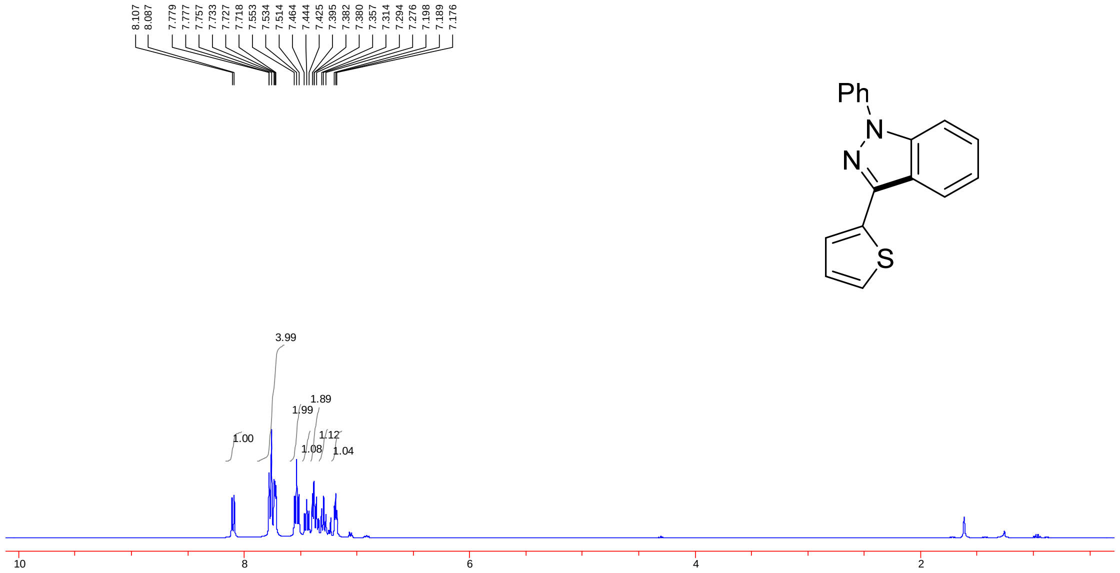

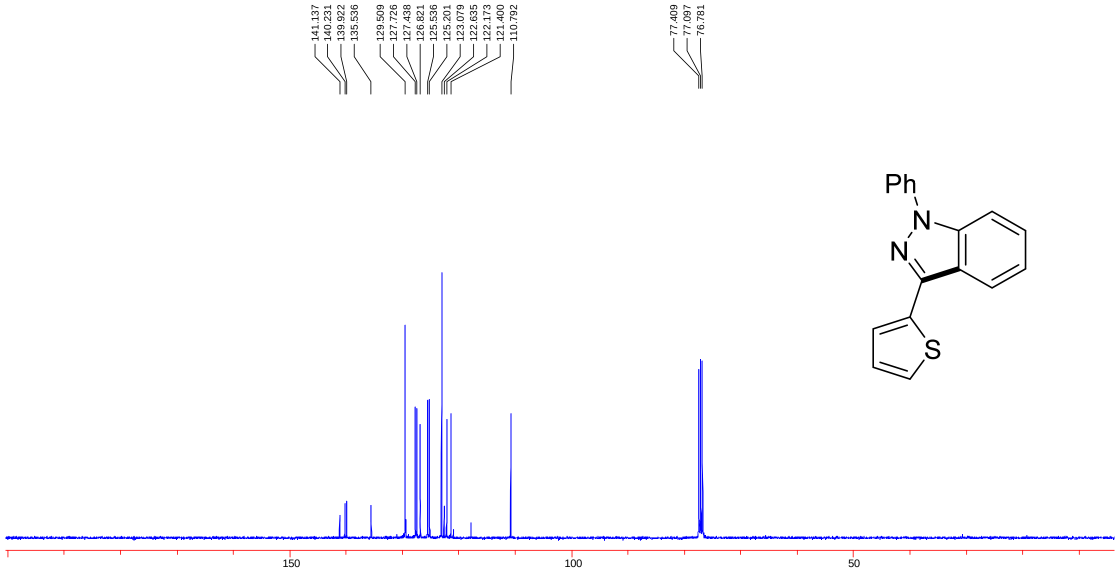

NMR spectra of **2t**

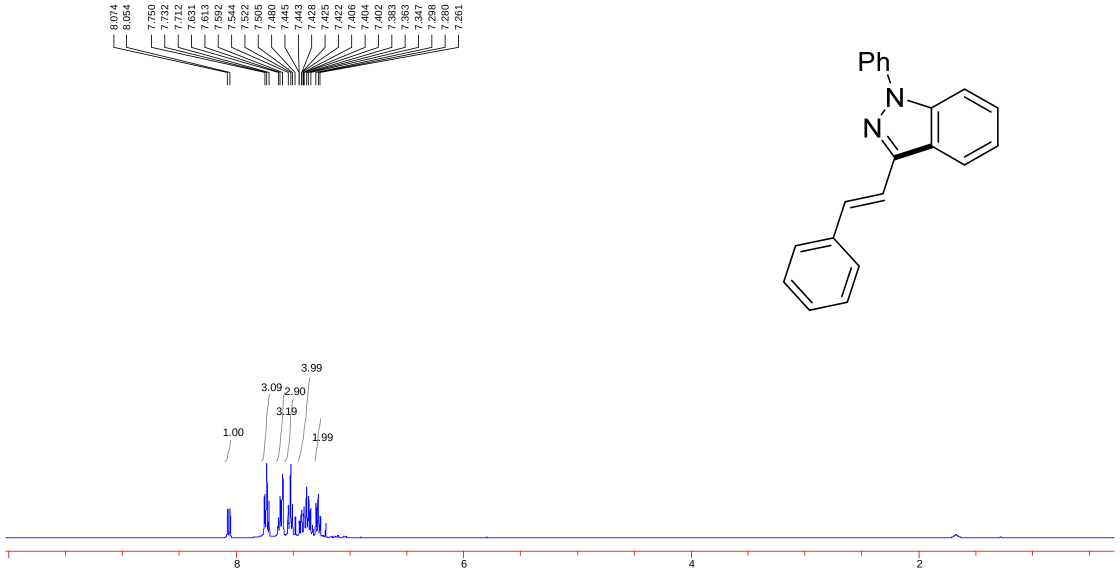

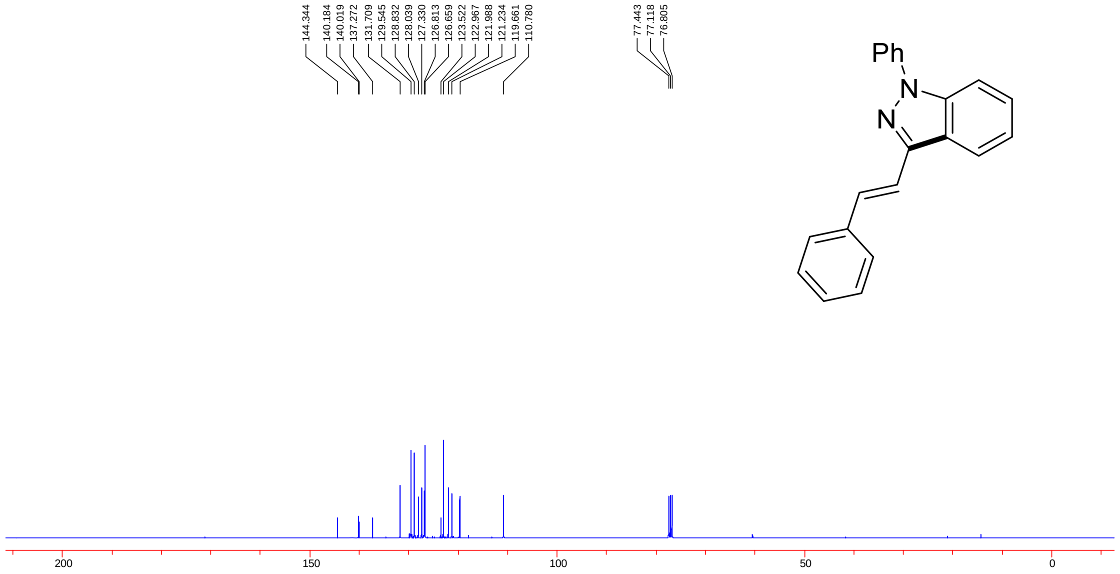

NMR spectra of **2u** and **2u'**

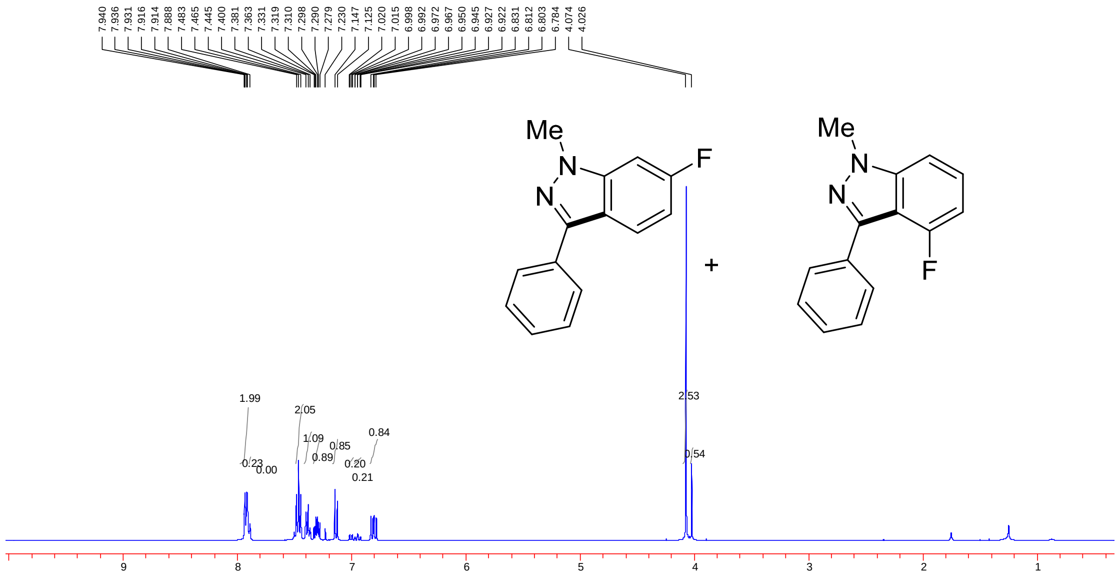

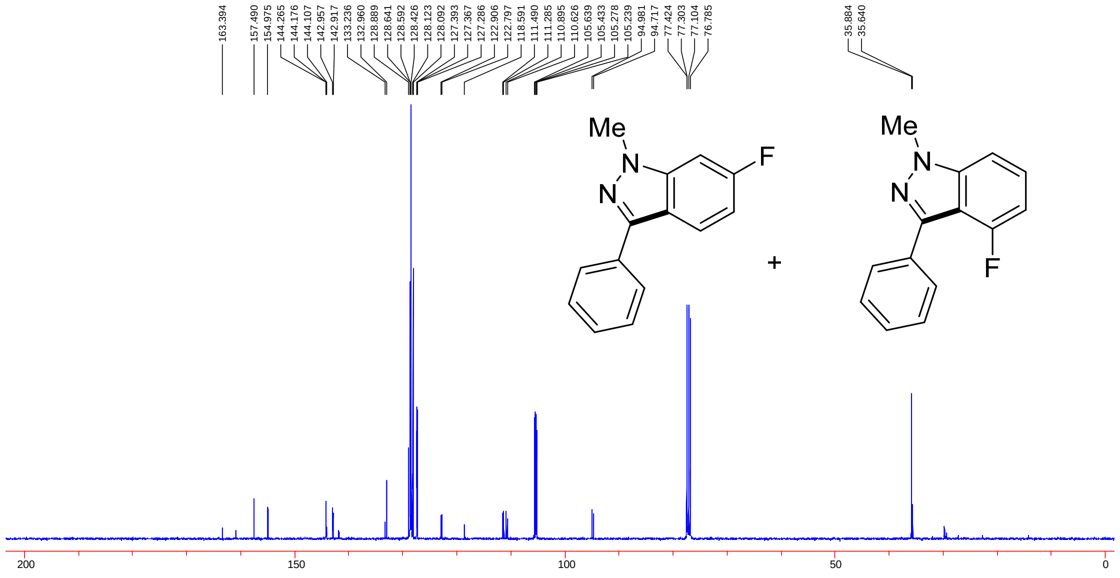

NMR spectra of **2v** and **2v’**

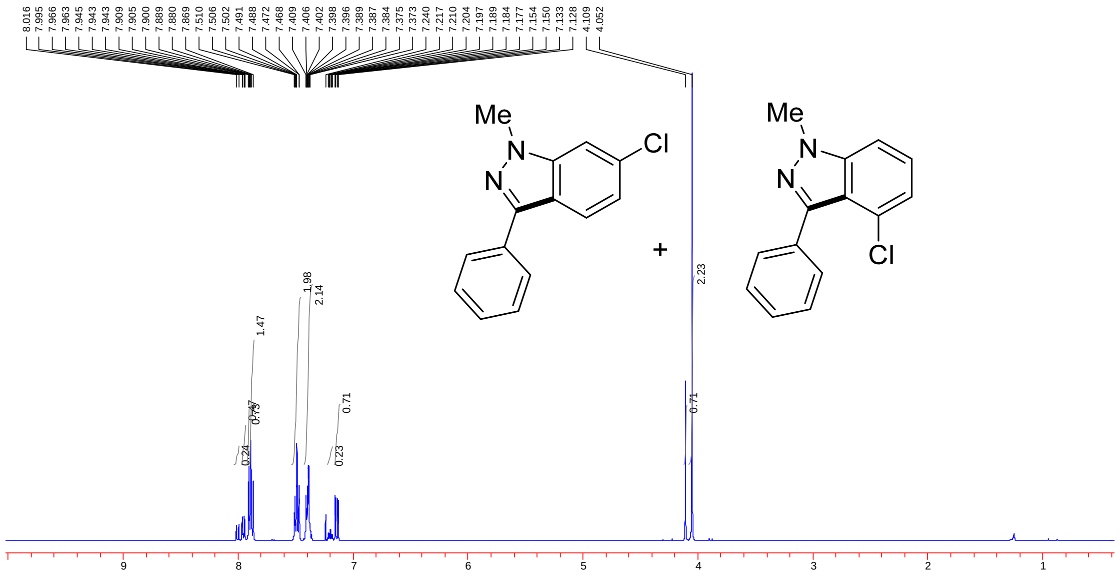

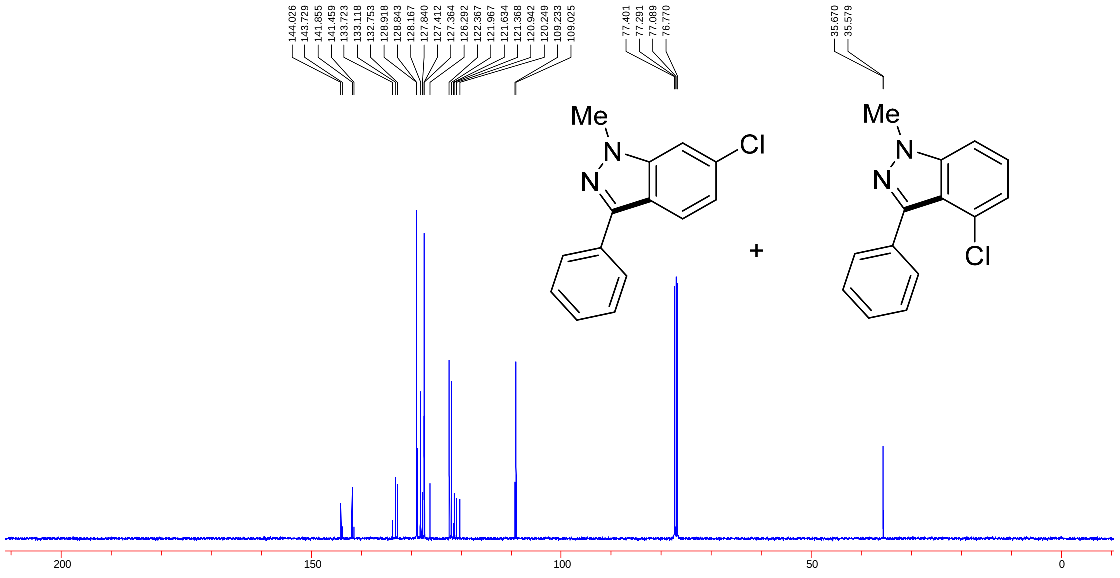

NMR spectra of **2w**

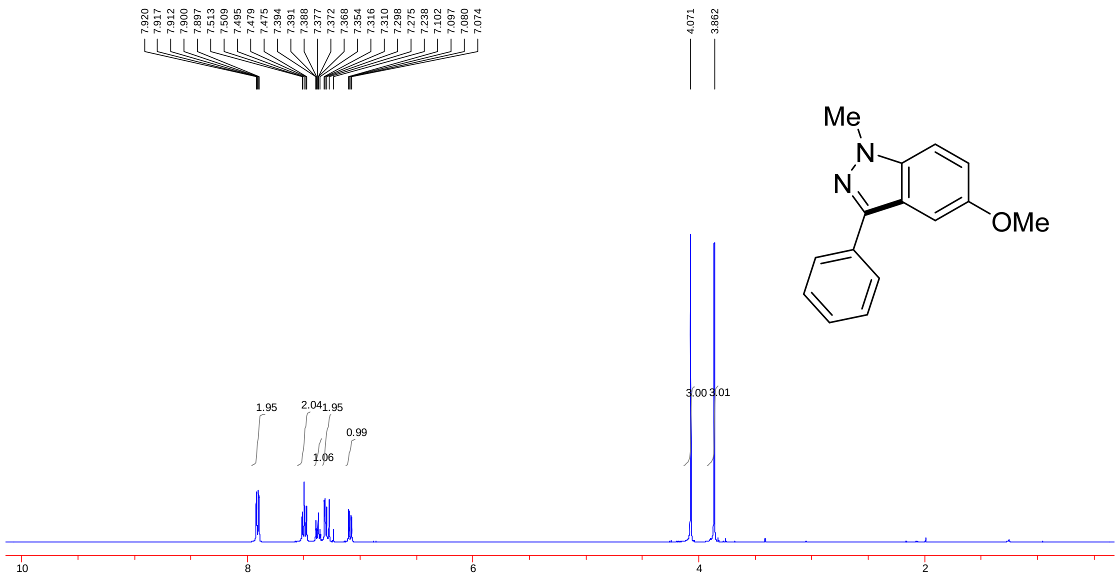

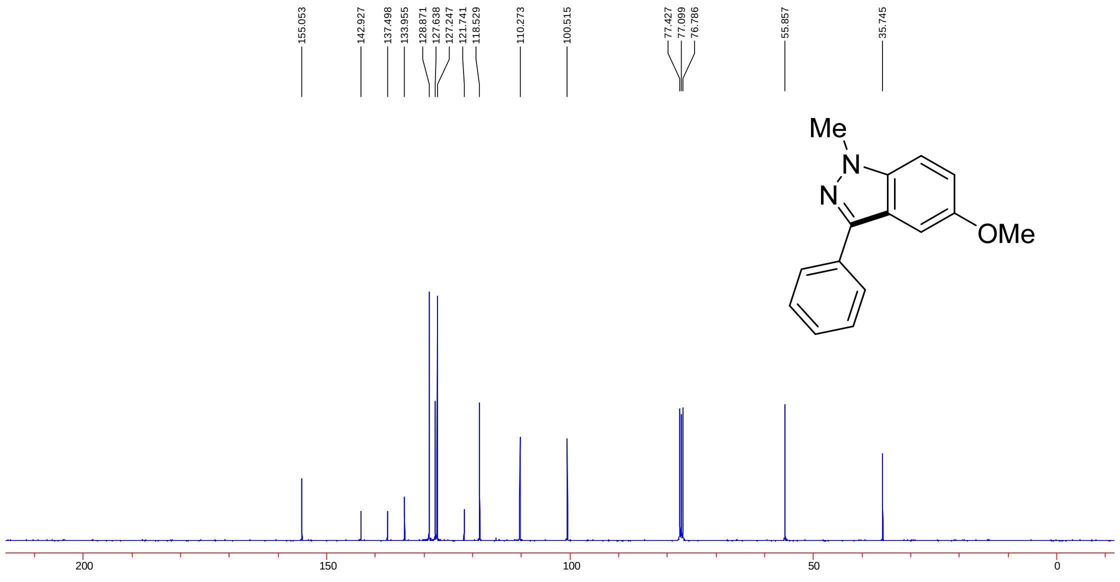

NMR spectra of **2x**

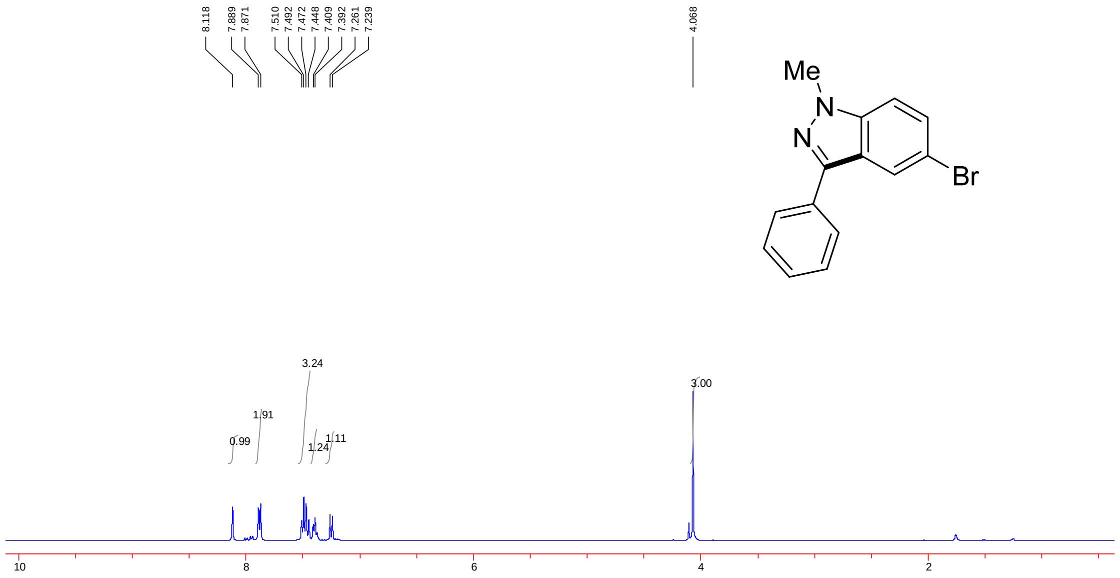

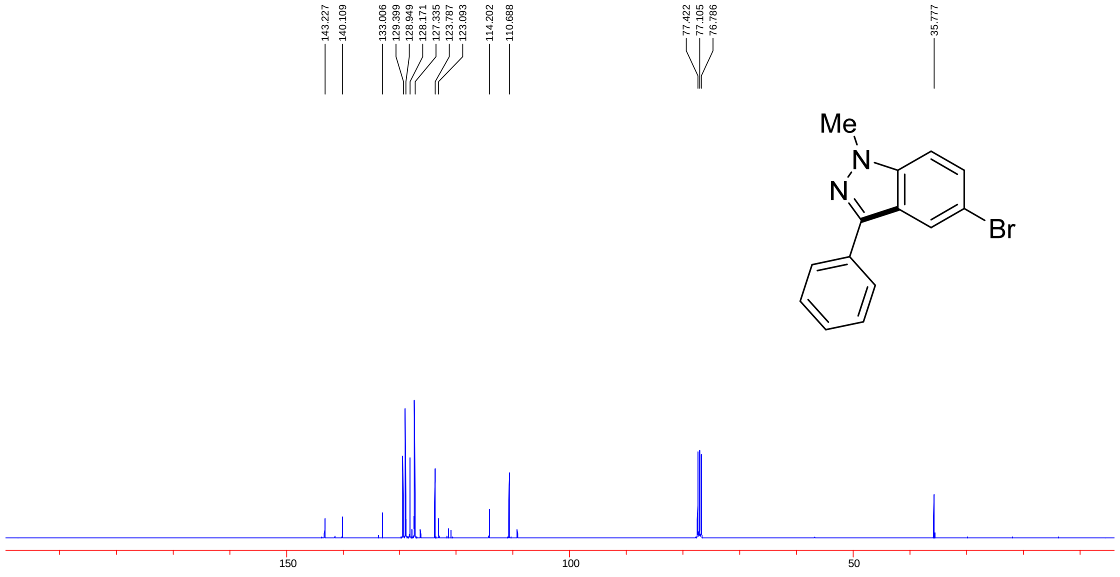

NMR spectra of **4a**

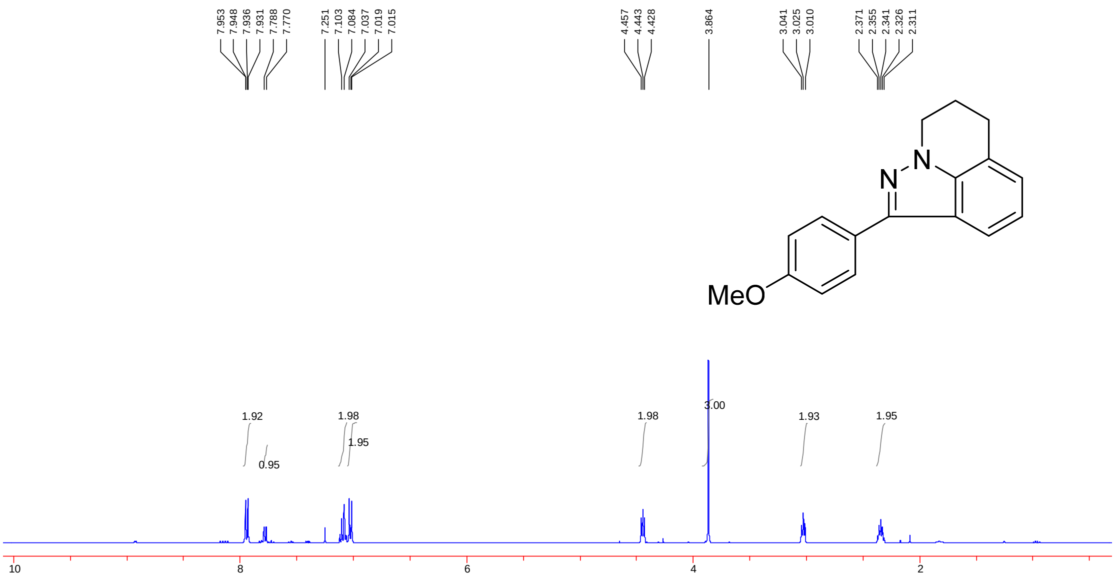

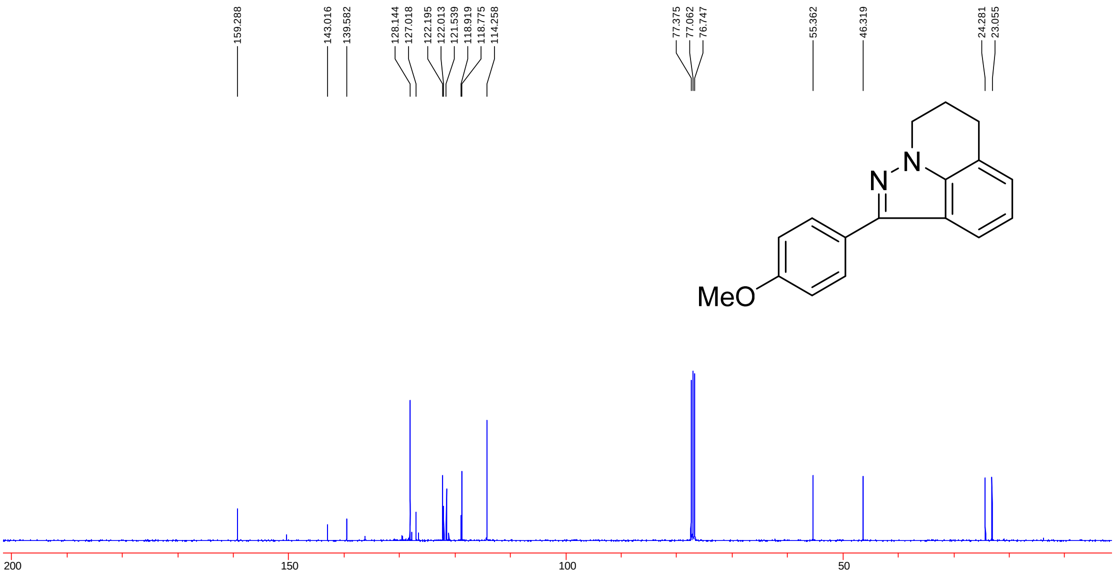

NMR spectra of **4b**

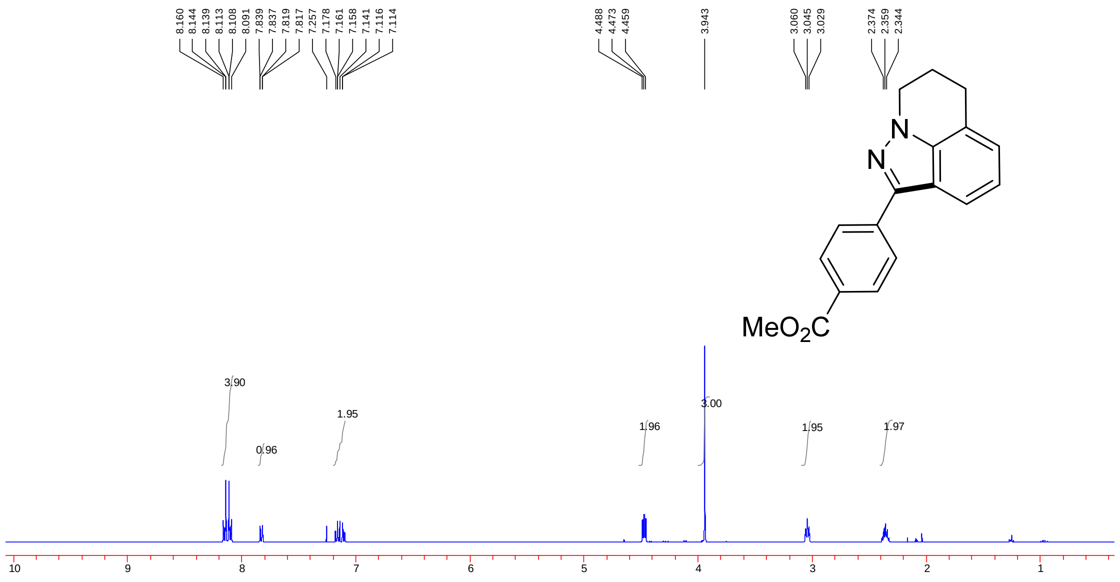

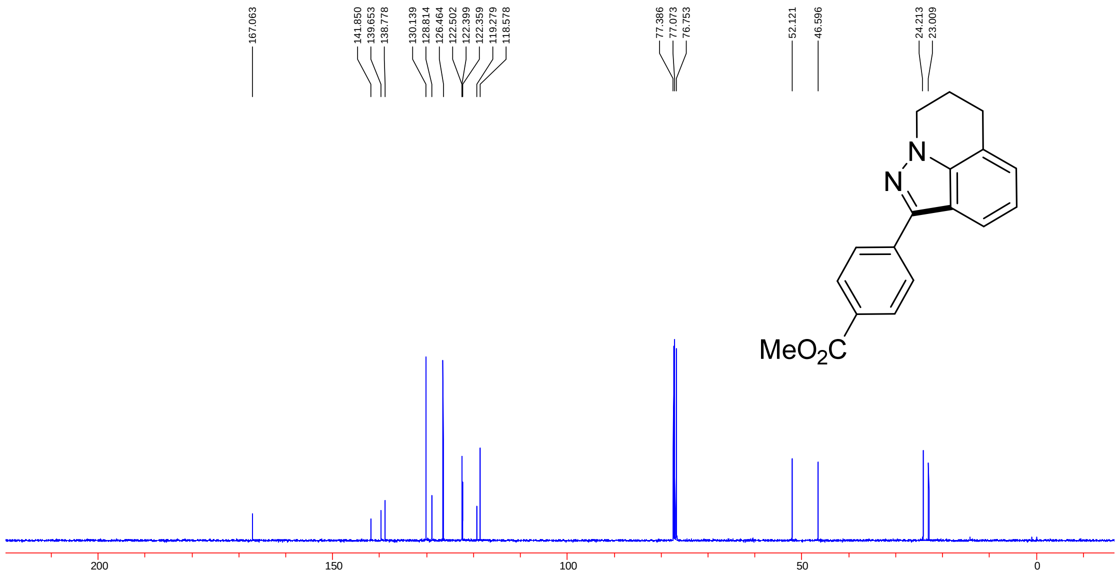

NMR spectra of **4c**

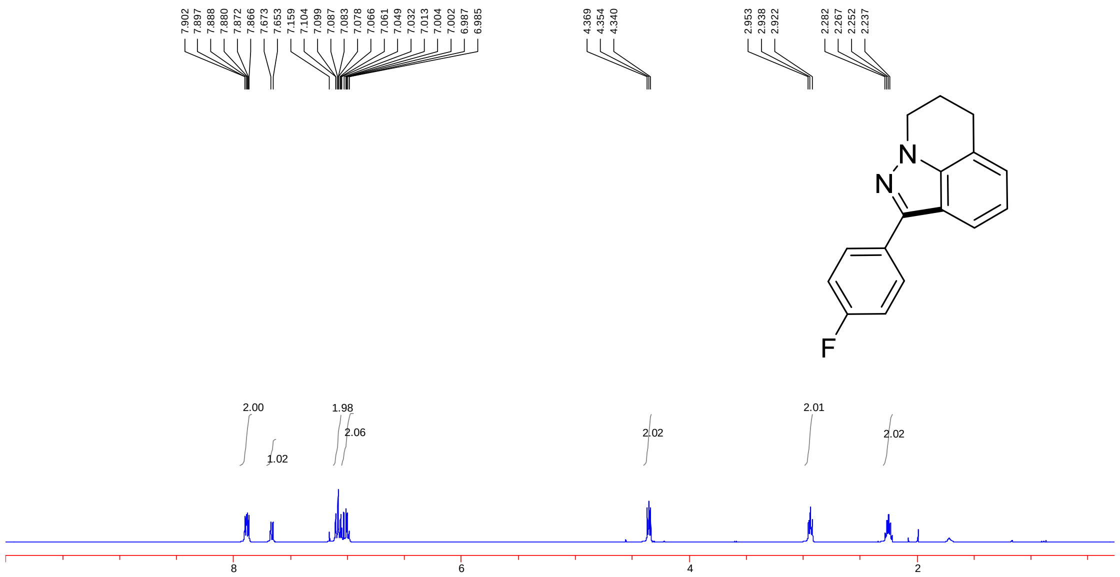

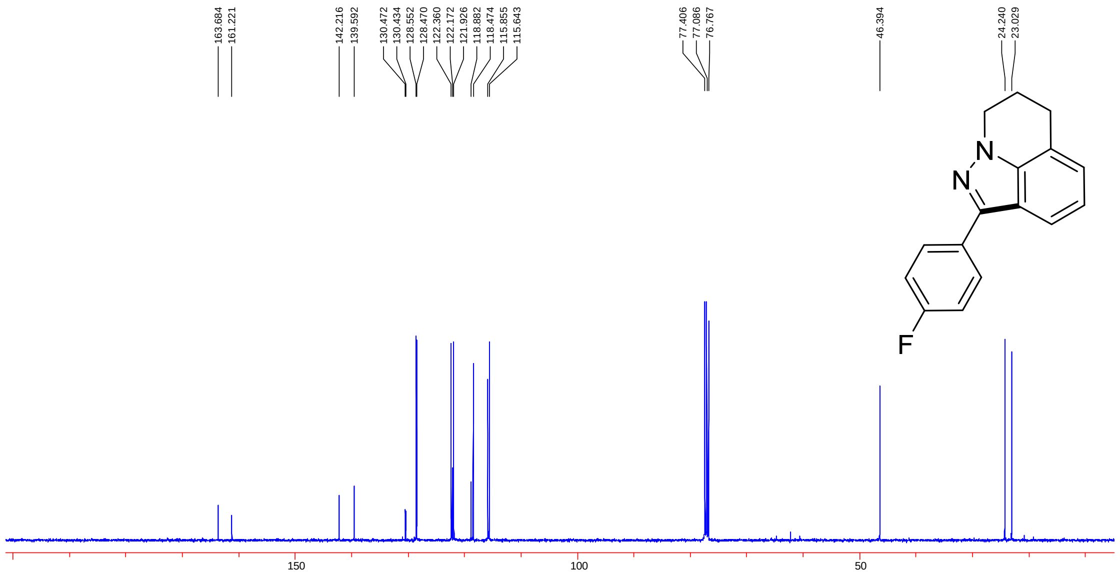

NMR spectra of **4d**

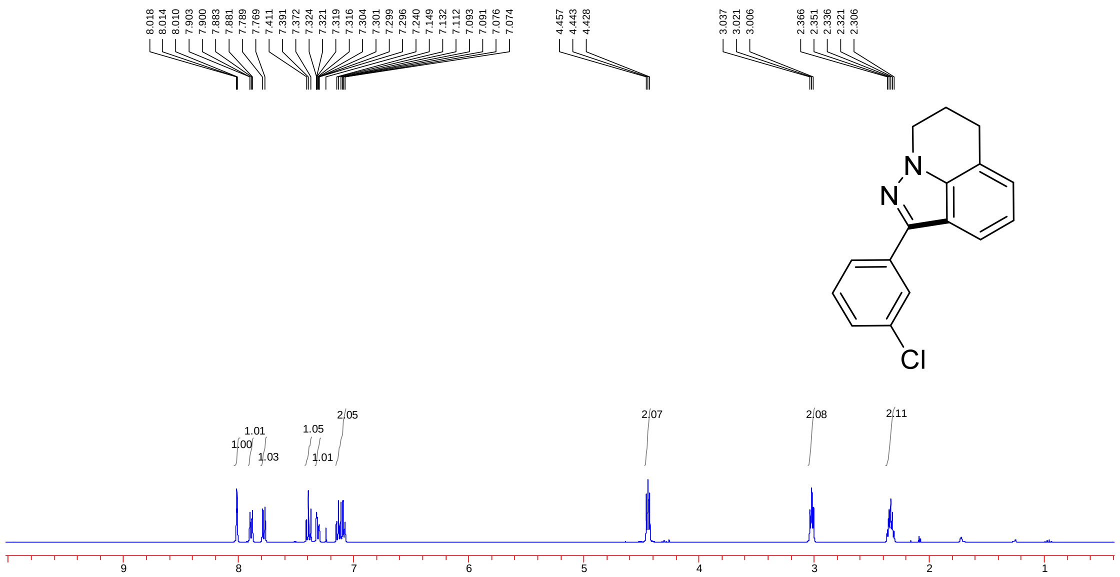

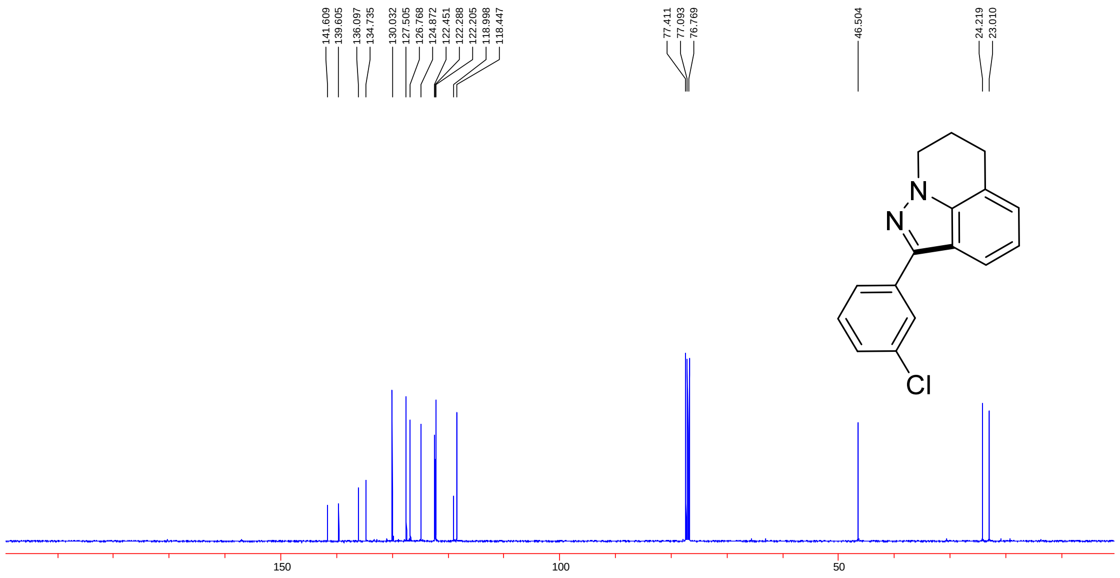

NMR spectra of **4e**

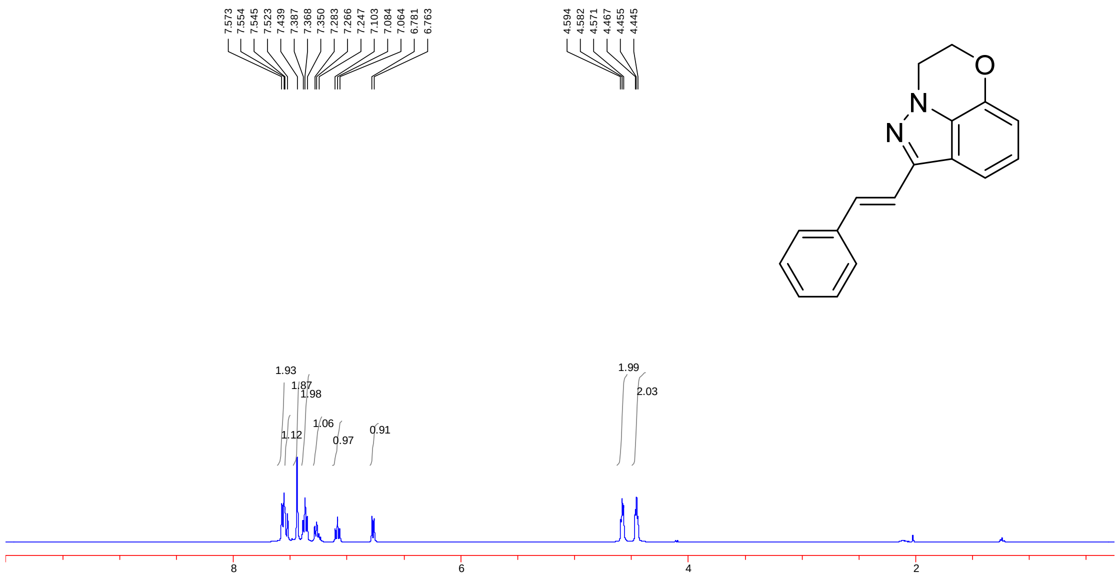

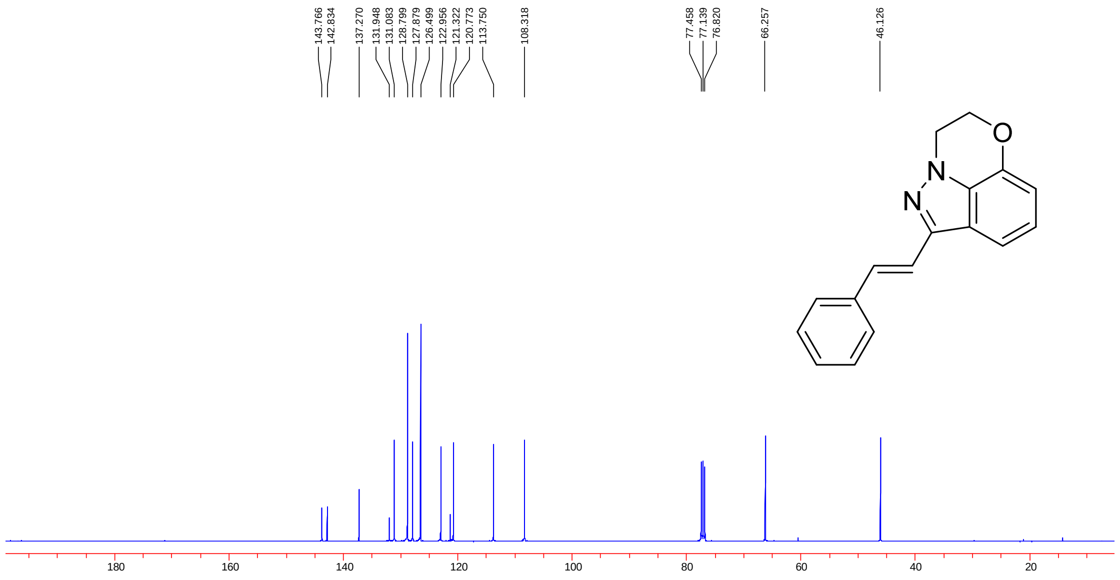

NMR spectra of **4f**

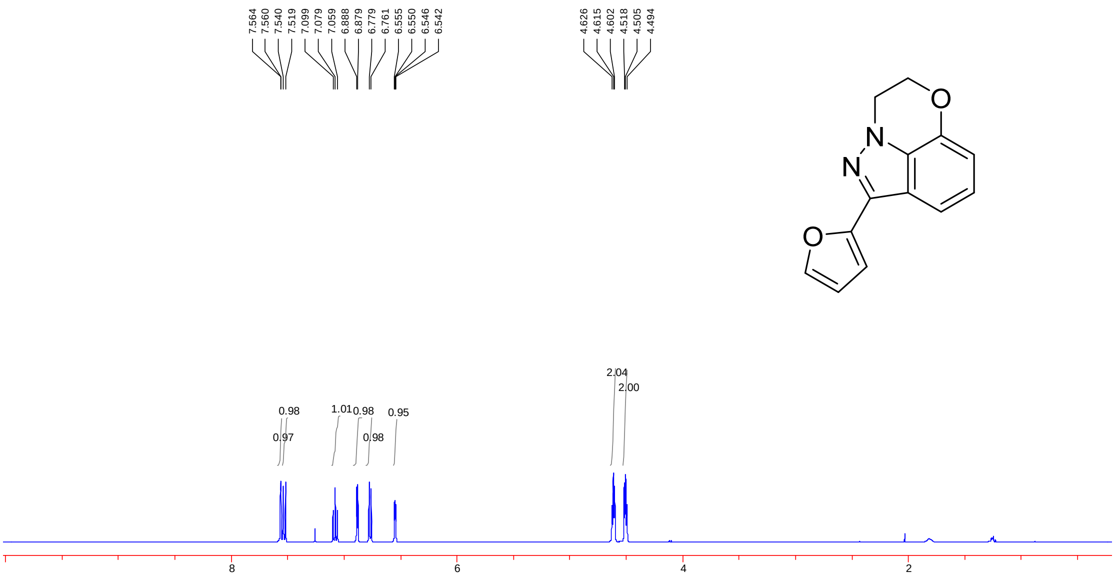

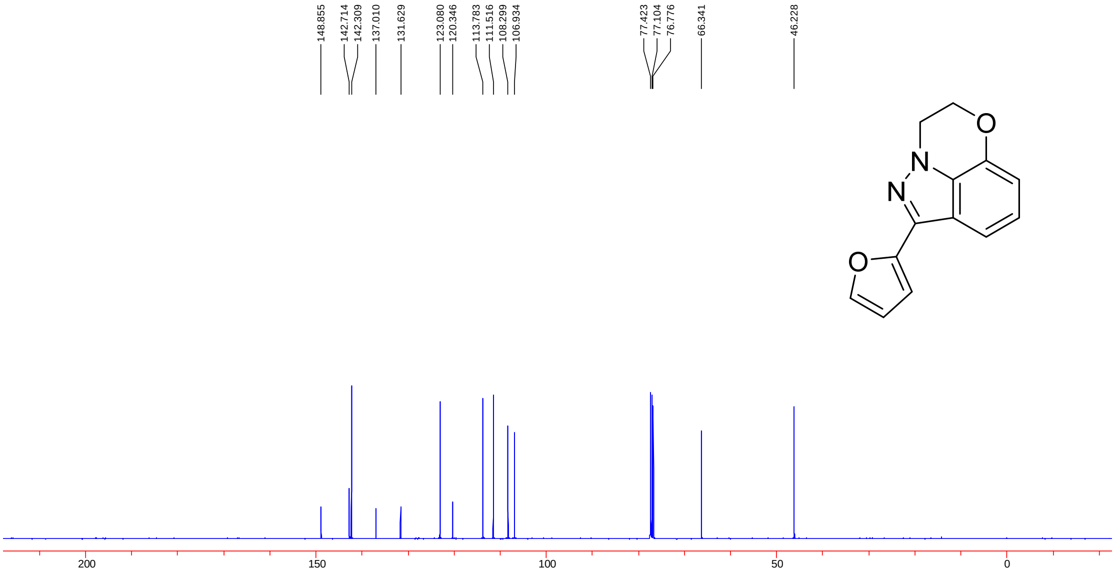

Supplement: Supplementary file 1 [file SC-008-C6SC03888C-s001.pdf]
